# Genome-Wide Study of the Defective Sucrose Fermenter Strain of *Vibrio cholerae* from the Latin American Cholera Epidemic

**DOI:** 10.1371/journal.pone.0037283

**Published:** 2012-05-25

**Authors:** Daniel Rios Garza, Cristiane C. Thompson, Edvaldo Carlos Brito Loureiro, Bas E. Dutilh, Davi Toshio Inada, Edivaldo Costa Sousa Junior, Jedson Ferreira Cardoso, Márcio Roberto T. Nunes, Clayton Pereira Silva de Lima, Rodrigo Vellasco Duarte Silvestre, Keley Nascimento Barbosa Nunes, Elisabeth C. O. Santos, Robert A. Edwards, Ana Carolina P. Vicente, Lena Lillian Canto de Sá Morais

**Affiliations:** 1 Laboratory of Environmental Microbiology, Evandro Chagas Institute, Ananindeua, Pará, Brazil; 2 Laboratory of Molecular Genetics of Microorganisms, Oswaldo Cruz Institute, Rio de Janeiro, Brazil; 3 Bacteriology Section of the Evandro Chagas Institute, Ananindeua, Pará, Brazil; 4 Centre for Molecular and Biomolecular Informatics, Radboud University, Nijmegen Medical Centre, Nijmegen, The Netherlands; 5 Centre for Molecular Life Sciences, Radboud University, Nijmegen Medical Centre, Nijmegen, The Netherlands; 6 Department of Computer Science, San Diego State University, San Diego, California, United States of America; 7 Department of Biology, San Diego State University, San Diego, California, United States of America; 8 Center for Technological Innovation, Evandro Chagas Institute, Ananindeua, Pará, Brazil; 9 Evandro Chagas Institute, Ananindeua, Pará, Brazil; 10 Department of Computer Science, San Diego State University, San Diego, California, United States of America; 11 Mathematics and Computer Science Division, Argonne National Laboratory, Argonne, Illinois, United States of America; Charité-University Medicine Berlin, Germany

## Abstract

The 7th cholera pandemic reached Latin America in 1991, spreading from Peru to virtually all Latin American countries. During the late epidemic period, a strain that failed to ferment sucrose dominated cholera outbreaks in the Northern Brazilian Amazon region. In order to understand the genomic characteristics and the determinants of this altered sucrose fermenting phenotype, the genome of the strain IEC224 was sequenced. This paper reports a broad genomic study of this strain, showing its correlation with the major epidemic lineage. The potentially mobile genomic regions are shown to possess GC content deviation, and harbor the main *V. cholera* virulence genes. A novel bioinformatic approach was applied in order to identify the putative functions of hypothetical proteins, and was compared with the automatic annotation by RAST. The genome of a large bacteriophage was found to be integrated to the IEC224's alanine aminopeptidase gene. The presence of this phage is shown to be a common characteristic of the El Tor strains from the Latin American epidemic, as well as its putative ancestor from Angola. The defective sucrose fermenting phenotype is shown to be due to a single nucleotide insertion in the *V. cholerae* sucrose-specific transportation gene. This frame-shift mutation truncated a membrane protein, altering its structural pore-like conformation. Further, the identification of a common bacteriophage reinforces both the monophyletic and African-Origin hypotheses for the main causative agent of the 1991 Latin America cholera epidemics.

## Introduction

*Vibrio cholerae* strains harboring the O1 or O139 surface antigen are the etiological agents of epidemic cholera. The choleragenic O1 lineages comprise the classical and El Tor biotypes, responsible for the sixth and seventh cholera pandemics, respectively [1]. They originated independently [2] and the estimated divergence date of their most recent common ancestor (MRCA) is the end of the nineteenth century [3]. During the twentieth century, the El Tor biotype became epidemic in Indonesia and spread to Asia, Africa, Europe and reached Latin America in 1991 [4].

The emergence of the *V. cholerae* El Tor biotype, as well as its successful spread to all continents, is associated with progressive genomic diversification, acquisition of mobile genomic elements, and adaptation to different environments from a monophyletic start point [5]. Thirty years after its pandemic expansion in the 1960s, it reached Latin America at the harbor city of Chimbote/Peru where it caused a major outbreak that quickly spread throughout the continent [6]. Prior to that, Latin America had not experienced a cholera epidemic since the fifth pandemic (1881–96) [7].

Many studies suggest that the Latin American epidemic was caused by a distinguishable monophyletic strain [8]–[13]. Phylogenomic analysis of 136 El Tor isolates clustered the Latin American epidemic strains with an Angolan strain isolated in 1989 [13]. In this period, an increased circulation of people between Africa and Latin America was reported, due to the Cuban Angolan intervention (1975–1991).

**Figure 1:**
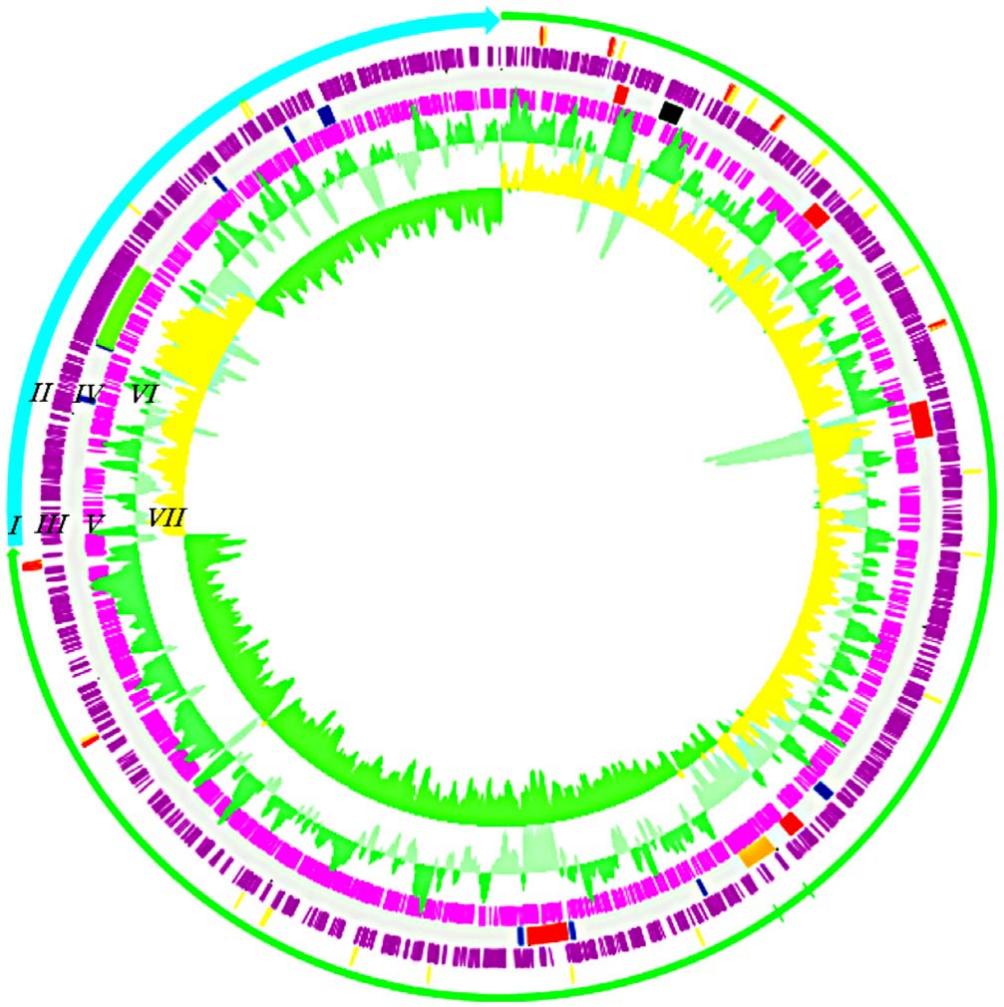
Circular plot of the IEC224 genome. The green arrow of ring I represents chromosome I, while the blue arrow represents chromosome II. Red and yellow traces on ring II represent, respectively rRNAs and tRNAs. Rings III and V show the predicted CDS, respectively clockwise and counter-clockwise. Ring IV represents putative mobile regions – elements associated with virulence and pandemic strains are colored red: *Vibrio* pathogenicity island I and II (VPI-1 and VPI-2); cholera toxin phage (CTX), *Vibrio* seventh pandemic islands I and II (VSP-1, and VSP2). Genomic islands predicted by the study in reference 38 (GI I-X) are blue, the O antigen gene cluster is black, the Latin American marker phage is orange, and the super integron is green. The Ring VI contains the GC plot, where areas with GC above range are dark green, while GC below range is light green. Ring VII contains the GC skew (G−C/G+C), in which regions above average are colored yellow, while below average are colored green. (Image generated in the DNA Plotter software [38])

**Table 1:** General genome features of the *V. cholerae* strain IEC224.

	Chromosome I	Chromosome II	Total
**Size (bp)**	3,007,450	1,072,136	4,079,586
**GC percentage (%)**	47.66	46.92	47.5
**CDS**	2,834	1,067	3,901
**Average CDS size (bp)**	945	849	919
**Hypothetical proteins**	566	346	912
**rRNA**	25	0	25
**tRNA**	94	4	98
**Coding sequences (bp)**	2,708,064 (90.05%)	911,994 (85.06%)	3,620,058 (88.74%)
**Non-coding sequences (bp)**	299,386 (9.95%)	160,142 (14.94%)	459,528 (11.26%)

**Figure 2:**
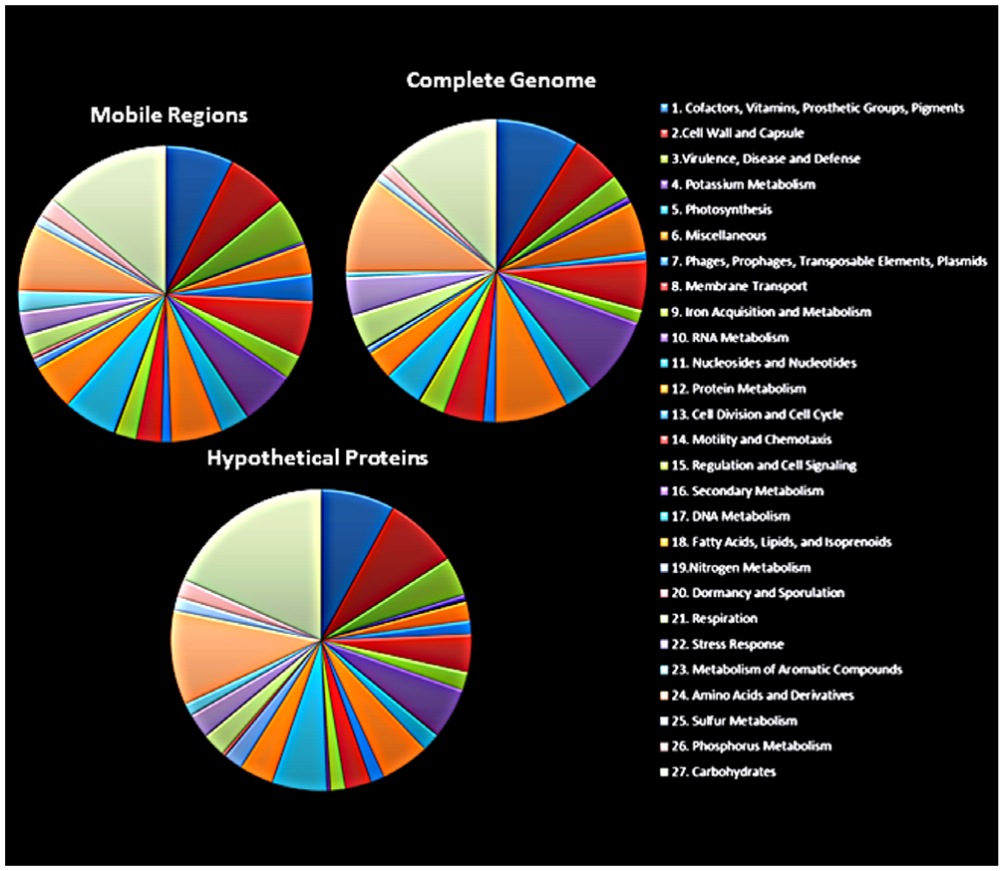
Distribution of the IEC224 genes in Clusters of Orthologous Groups (COG). Proportional distribution of genes in COGs as assessed by the RAST annotation pipeline [21]. Each color corresponds to a level 1 subsystem (subsystems are described in reference [32]), starting at Cofactors, Vitamins, Prosthetic groups, Pigments (dark blue) and ending with the Carbohydrates subsystem (light gray). In the central pie all of the IEC224 genes were considered, while the upper left pie contains only the genes from mobile genomic regions, and the lower left pie contains the genes from hypothetical proteins that had their function assigned by the Real-Time Metagenomics Portal (see the main text and reference [33]).

**Figure 3:**
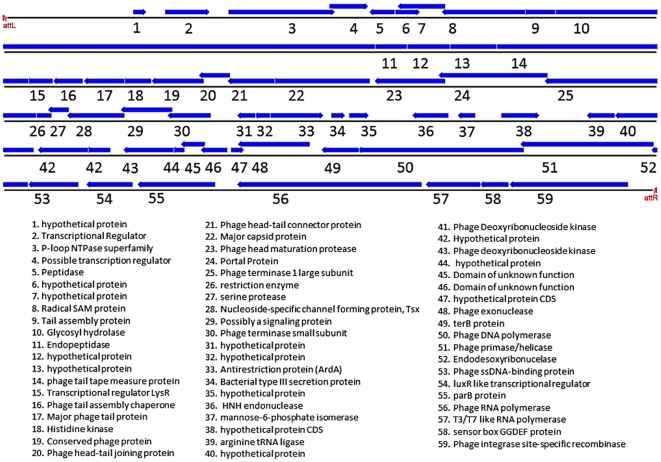
Genome of the Latin American epidemic *Vibrio cholerae* marker phage. Graphical representation of the genes that correspond to the genome of a bacteriophage that is present in all the Latin American epidemic strains tested in this study. The CDS are the blue arrows that are pointed towards the direction they are coded in the genome. The putative protein functions are listed below, with a corresponding number to its localization in the image. (Images generated in the Geneious software – reference [24])

**Figure 4:**
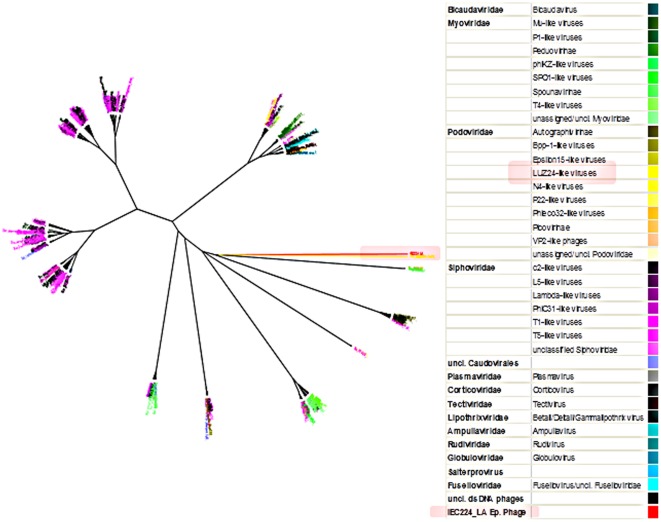
Classification of the *V. cholerae* Latin American epidemic phage in the dsDNA Phage Proteomic Tree. Neighbor Joining comparison of all the proteins of 733 dsDNA phages grouped the Latin American epidemic phage with the genus of LUZ24-Like viruses. These are phages from the Podoviridae family that conform a separate cluster, which is different from all other phages classified in the tree. The group containing these phages is highlighted pink. On the right is a table with the color keys of the phages that are colored in the tree according to their ICTV classification [36].

**Figure 5:**
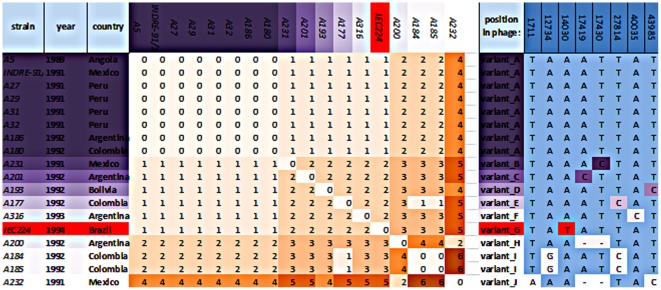
Variants of the Latin American epidemic phage in sequenced V. cholerae genomes. The assembly of sequencing reads from 16 other El Tor *V. cholerae* genomes from Latin America, and their putative ancestor strain from Angola, revealed the presence of the Latin American epidemic phage in all strains. The genomic variations were in 8 sites numbered from bases 0 through 49,291 (right). Collectively these strains formed 10 variants of the phage, with the variant A being the most abundant. This variant is shared with the putative ancestor strain. All strains accumulated at least one SNP after 1992.

**Figure 6:**
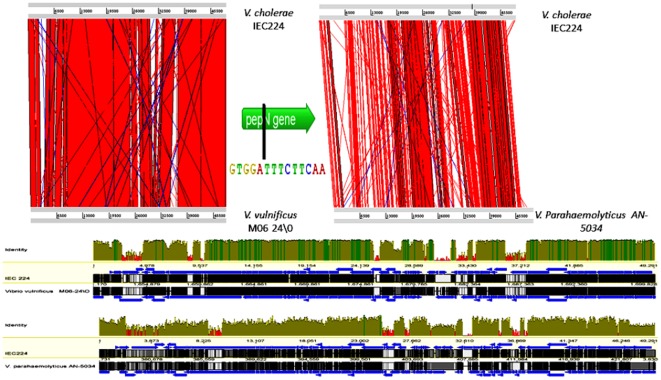
A similar bacteriophage is present in strains of two other Vibrio species. The *Vibrio vulnificus* strain MO6 24/O and the *Vibrio parahaemolyticus* AN-503 both contain a similar bacteriophage inserted in the same repeat sequence of the membrane alanine aminopeptidase (pepN) gene which is shown in the center of the top figure. The Artemis Comparison Tool (ACT) alignment shows nucleotide matches as red lines, inverted repeats as blue lines, and gaps as white spaces. The lower image shows alignments were the corresponding genes are represented as blue arrows. Above these alignments, there is a pair-wise similarity representation, showing identical matches as dark vivid green and similarity grades down into lighter green tones. Mismatches are red. (Images generated in ACT [39], and Geneious [24] softwares)

**Figure 7:**
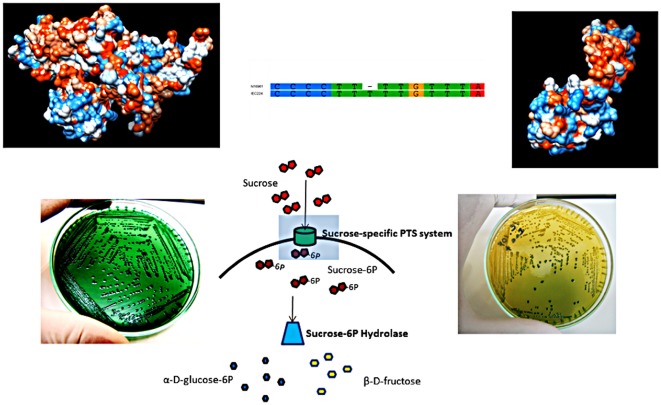
The altered sucrose phenotype of the strain IEC224 is due to a frame-shift mutation. The characteristic phenotype of *Vibrio cholerae* is to produce shiny gold colonies after 48 h incubation on TCBS agar (as pictured on the right side). The strain IEC224 fails to ferment sucrose and colonies remain green (pictured on the left). The only genomic difference between a functioning sucrose fermenter and the IEC224 strain is an insertion in the gene coding for the sucrose-specific IIB domain of the PTS system, which is shown above in the aligned fragments of the mutated IEC224 gene and the functioning N16961 gene. A diagram with the metabolic role of this protein is illustrated in the center, showing that it is a carrier that selectively transports sucrose into the cell and phosphorylates it to signal downstream reactions. A model of the functioning protein structure is shown on the top left, as well as a model for the altered structure can be seen in the top right. (Model generated by PHYRE^2^, following the pipeline of reference [25])

**Figure 8:**
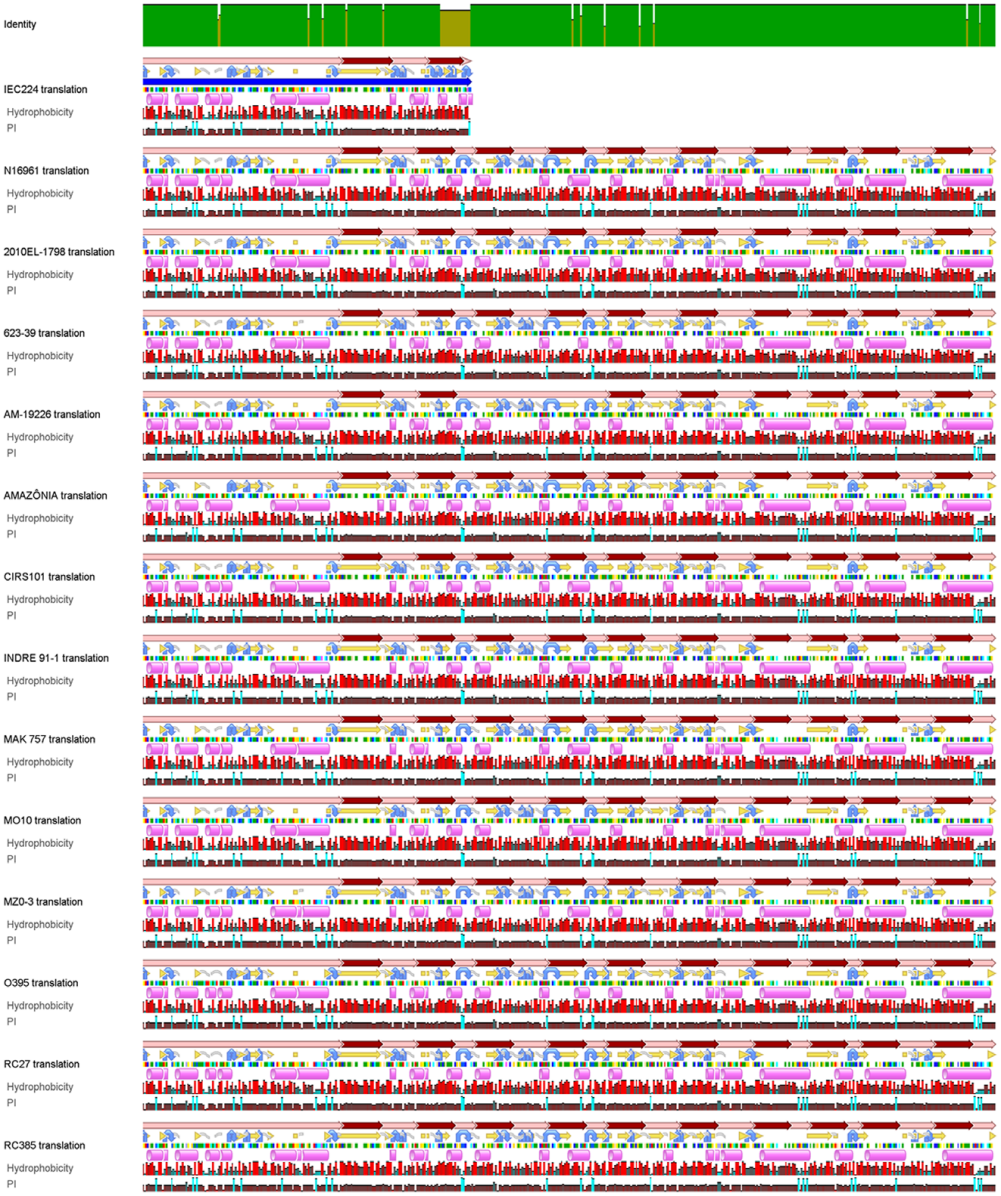
Protein alignment reveals a conserved structure of the sucrose-specific IIB domain of the PTS system gene in *Vibrio cholerae* strains. Comparative amino-acid alignment of the sucrose-specific IIB domain of the PTS system gene in all the of Vibrio cholerae strains that have their genome available in the NCBI portal. Because of space only 13 strains are shown, but 42 were actually aligned, exhibiting a similar profile. A highly conserved structure is observed in the transmembrane domains (alterning pink for cytoplasmic and extracellular domains, and dark red for transmenbrane domains), as well as in the secondary structure (purple cilinder – represent alpha helix; yellow arrows – beta strands; gray coil – coils; blue curved arrows – turns), hydrophobicity, and isoelectric point (PI) (both shown as bar graphs). This profile is the same in virtually all V. cholerae strains, but is deeply altered in the truncated protein of the IEC224 strain, which has frame-shift mutation in amino-acid residue 185 (Image generated in the Geneious software [33])

During the spread of cholera through Latin America, genetic and phenotypic diversification of the causative agent was reported [14], [15]. In this context, in the year of 1994, a variant that failed to ferment sucrose when plated on thiosulphate-citrate-bile-salt-sucrose agar (TCBS-agar) [16] began to be isolated from cholera cases in the Northern Brazilian Amazon region. This variant drove local cholera outbreaks until 1996. Defective sucrose fermenting *V. cholerae* strains have been reported in other continents, although the genetic determinants for this phenotype are unknown [17], [18].

Genomic data of strains from the Latin American cholera epidemic is scarce. Among the *V. cholerae* genomes available in GenBank, only one draft genome sequence (INDRE 91/1, ADAK00000000) is a representative of this epidemic. Raw data from 15 other strains reported [13] are also available through the European Nucleotide Archive (ENA).

Here, the complete genome of the *V. cholerae* strain IEC224 is analyzed. This strain is shown to harbor the unique features of the main Latin American epidemic El Tor strains. Nevertheless, it exhibits a defective sucrose phenotype as a distinguishing characteristic. Supporting text S1 contains a brief review of the local epidemic context in which the IEC224 strain was isolated.

This study also provides a broad comprehension of the Latin American seventh pandemic lineage. The unique genomic features are summarized and compared with other genomes that were available. The novel genomic island denoted WASA1 [13] is demonstrated to be a phage genome, and ubiquitous in the Latin American strains so far analyzed. Moreover, the genetic determinant of the non-sucrose-fermenting phenotype of this strain is proposed.

## Methods

### Purification of genomic DNA

Total genomic DNA was purified as described previously [19]. Briefly, harvested cells of overnight LB broth cultures were subjected to: alkaline lyses, proteinase K and RNase P digestion, phenol – chloroform – isoamyl alcohol purification, and two ethanol precipitations. The final product was washed three times in 70% ethanol, air-dried and re-suspended in TE buffer. Quantity and quality were assayed by the Nanodrop 2000c spectrophotometer (Thermo Scientific).

### Sequencing, assembling, and genome annotation

Two Next Generation Sequencing platforms were used to generate the whole genome sequence of the *V. cholerae* IEC224 strain. A hybrid strategy was used to assemble the sequencing reads: first, a *de novo* method was used to assemble long reads from the GS FLX 454 system (Roche, Applied Science); then a resequencing methodology was used to assemble short reads from the SOLiD 3 Plus platform (Life Technologies). In order to close the genome, deep sequencing strategies were used, including reads on a mated-paired library.

The data generated by the GS FLX 454 was assembled *de novo* with the Newbler software (version 2.6, Roche). Ninety-two contigs were generated from 319,825 reads with an average size of 400 nucleotides. The sequencing depth was of 35.5×, which covered approximately 95% of the genome in comparison to the *V. cholerae* strain N16961 (chromosome I: NC_002505.1 and chromosome II: NC_002506.1 [20]).

Furthermore, in order to close the genome, a mated-paired library was sequenced in 23 million short reads by the SOLiD 3 Plus platform, yielding a genome coverage of 268× in comparison to the reference. We used the SOLiD™ Bioscope software (Applied Biosystems) for resequencing and the N16961 strain as a reference. A final consensus sequence was generated with the scaffolds of both sequencers. The hybrid sequencing strategy, using short reads of SOLiD for re-sequencing, allowed us to close the gaps of the *de novo* sequence generated from the assembly of the long pyrosequencing reads. Through this pipeline we were able to close difficult sequencing regions such as the class 4 integron cassette array called *V. cholerae* superintegron (SI). This was also achieved because a phylogenetically close reference was available to support the re-sequencing assembly.

The final sequence was submitted to automatic annotation by the Rapid Annotation using Subsystem Technology (RAST) pipeline [21]. The resulting annotation was manually curated based in the *V. cholerae* Genome Blast (NCBI). Both chromosome sequences were deposited in the GenBank database under the accession numbers CP003330 and CP003331, respectively for chromosome I and II.

### Assembly of genomes from the European Nucleotide Archive

The genomes of Latin American El Tor *V. cholerae* strains were downloaded from the European Nucleotide Archive and assembled using Velvet [22] (Supporting Table S1 shows the assembly statistics). The reference with the highest Megablast bitscore hit was selected out of the five completely closed *V. cholerae* genomes (N16961, O395, MJ-1236, M66-2, and LMA 3984-4), and we scaffolded the contigs against this reference using *scaffold builder* (source code available at: http://sourceforge.net/projects/scaffold-b/files/) with default parameters.

### The Phage Proteomic Tree

The phage proteomic tree was constructed as described in reference [23] and online in the webpage http://phantome.org/Tree/construction.html

### Proteomic analyses

Secondary structures, transmembrane regions, hydrophobicity, and other physical properties of proteins were predicted from the corresponding amino-acid residues using mainly the Geneious software [24] and were further confirmed by tools that are available through the Uniprot/Swiss-Prot portal at the web-site: http://www.expasy.org/tools/. Quaternary models were generated from the sequence of amino-acid residues through the pipeline described in reference [25], using the Phyre2 server.

### Polymerase chain reaction (PCR)

Specific primers to regions of the genomic island WASA1 were designed with the primer3 software [26]. Platinum TAQ kit (Invitrogen) was used for DNA amplification. Manufacturer's instructions were followed, using primer-specific annealing temperatures. Results were assessed by electrophoresis and confirmed by sequencing on the ABI3130 sequencer (Applied Biosystems).

## Results and Discussion

### General genome features

Two high-throughput Next Generation sequencing technologies were combined to determine the complete genome sequence of the IEC224 strain. The genome was 4,079,586 base pairs (bp) in length: 3,007,450 bp on chromosome I, 1,072,136 bp on chromosome II. We predicted 3,901 coding sequences (CDS), of which 912 were hypothetical proteins. The remaining 2,989 CDS had a function predicted by homology. 25 rRNAs (all on chromosome I) and 98 tRNAs (94 on chromosome I and 4 in chromosome II) were predicted (Figure 1). The general chromosome features are summarized in Table 1.

### GC signature and the function of the mobile genomic regions

The GC content is shown on ring VI of the genome plot (Figure 1) and the GC skew ((C−G)/(C+G)) on ring VII. Both are strong predictors of potentially mobile regions, in particular the ones linked with the *V. cholerae* virulence (red traces in the ring 4). GC skew measures strand-specific mutational bias [27]. Together these measures can indicate recent genetic recombination events. In this genome assembly, as with previously published sequences [3], [20], [28], [29], a backbone contains the core genes that are common to all *V. cholerae* genomes. This core genome is distinguished by a low GC deviation (0.003) and a high GC content (47.8%), while the putative mobile regions have a comparatively high GC deviation (0.005) and low GC content (43.2%).

Based on the GC content, and on previous comparative genomic studies of the *V. cholerae* species, we grouped genome fragments of the IEC224 genome in potentially mobile and low GC content regions. These regions were concatenated, and a summary of their gene content is shown in the Supporting Table S2. For this comparison we also considered the gene cluster that is responsible for the O antigen phenotype because this region contains a low GC content (Figure 1) and an extensive recombinational signal [29], [30].

Here, we show that out of the 332 genes that were located in low GC content/potentially mobile regions, only 55 have been directly implied in virulence, such as the A and B subunits of the cholera toxin (CTX) and the toxin co-regulated pilus cluster (TCP) (Supporting Table S2). The 277 remaining genes include functions for structural components of the cell wall and capsule, RNA and DNA metabolism, sugar amino-acid, aromatic compounds, and sulfur metabolism. In fact, new metabolic opportunities are present in predicted mobile regions, such as a system for the transport of fructose and nitrogen in Genomic Island IV (Figure 1, Supporting Table S2). There are also many genes for environmental sensing or interactions with other cells, such as methyl-accepting chemotaxis protein, outer membrane protective antigen OMA87 and paraquat inducible protein.

### COG distribution and assessment of a putative function for hypothetical proteins

The proteins of the IEC224 strain were distributed by homology in clusters of orthologous groups (COG) [31], [32] through the RAST annotation pipeline [21]. The Carbohydrate subsystem was the most abundant category with 408 orthologous genes, followed by the Amino Acids and Derivatives that contained 324 genes. The Dormancy and Sporulation group, as well as the group composed of Secondary Metabolism orthologous were represented only by 2 and 3 genes (Dormancy and Sporulation: cell inhibitor and peptidyl-tRNA hydrolase; Secondary Metabolism: 3 genes of the paerucumarin biosynthesis pathway). From 1,536 genes that were not assigned to any category, 798 were hypothetical proteins.

In order to understand the potential functions that hypothetical proteins could represent in this genome, they were submitted to the Real-Time Metagenomics Portal (RTMP) using default parameters [33]. Our objective was to test if this approach could also find the possible function of proteins that RAST could not identify in this bacterial genome. Since it was primarily developed for metagenomic reads, its efficiency was first compared to the RAST results for the non-hypothetical proteins. Very similar results were achieved for the proteins that RAST already identified. Because it uses a different approach to match potentially homologous genes, a broader range of putative functions were also found (Supporting Table S3). This is the first time to our knowledge that this approach was used to identify a putative gene function for proteins in a bacterial genome. It successfully identified homologous functions for 816 of the 912 hypothetical proteins. Here, the probable functions found for the hypothetical proteins are presented as a broad picture of the gene functions that have not been described in the El Tor strains. A detailed table with all the gene functions found and assigned to subsystems is provided in Supporting Table S4.

The most representative category of genes was related to the Carbohydrate subsystem. Interestingly, it contains four genes for the utilization of chitin, which is an important energy source for this species, as well as a natural competence activator [34]. The novel genes identified for this metabolism were a beta hexosaminidase, a chitinase, N-acetyl-D-glucosamine ABC transport system, permease protein 1, and N-acetylglucosamine-6-phosphate deacetylase. Other 147 genes were assigned to the Carbohydrate subsystem, 83 to the Amino Acids and derivatives. It was also identified 12 genes for Phages and Transposable Elements, including phage coat proteins, and plasmid elements. Thirty-four hypothetical proteins were identified with functions linked to the subsystem of Virulence, Disease and Defense, which included genes for resistance mechanisms such as multidrug resistance efflux pumps; cobalt, zinc, cadmium, cooper, and arsenic resistance; colonization factors; antibacterial peptides; invasion and intracellular resistance proteins. Figure 2 shows the proportional distribution of genes in level 1 subsystems [32]. The pies compare the complete genome with the potentially mobile regions and the hypothetical proteins.

### Clonality of the Latin American epidemic strains

In order to determine the genetic relationship between the IEC224 and the Latin American epidemic lineage, an allelic comparison of the complete genomes of four *V. cholerae* strains was performed.

First the IEC224 genes were compared with the N16961 strain, because this is a complete genomic scaffold that is considered the prototypical seventh pandemic clone (20). Small differences in the DNA composition of 122 genes (1 to 5 nucleotides) were detected. These genes were further compared with the genes from two draft genomic sequences, MO10 (India, 1992) and INDRE 91/1 (Mexico, 1991). The INDRE 91/1 strain was isolated from a cholera outbreak in Mexico, and represents an early epidemic isolate. Supporting table S5 presents the comparison of alleles and shows a pattern consistent with the clonal relation of the INDRE 91/11 and IEC224 strains. The Latin American strains shared 92 of the 122 alleles (75%), IEC224 and M010 shared 41 (33%), INDRE and M010 57 (46%), INDRE and N16961 23 (18%). Previously described Single Nucleotide Polymorphism (SNP) profiles that clustered 15 Latin American strains in a phylogenetic study of 136 El Tor strains [13] were compared with the IEC224 genome. The Latin American epidemic strains are characterized by 48 SNPs, and INDRE 91/1 and IEC224 shared this same SNP profile. They also shared 37 SNPs with the putative ancestral strain A5 from Angola. This SNP profile is described in detail in the supporting text of the reference 14, and the supporting table S5 contains a list of all SNPs that are exclusive of the Latin American El Tor strains of reference 15, the Angolan putative ancestor, INDRE 91/1, and IEC224.

### WASA1 bacteriophage

The genome alignment of the IEC224 and the N16961 strains revealed a large insertion of 49,277 base pairs within the gene coding for a membrane alanine aminopeptidase (VC1494 locus). This region integrated in a 14 nucleotide *att* site contains 59 predicted genes, many are conserved phage proteins (Figure 3), such as proteins for DNA replication and transcription, with at least 4 transcriptional regulators.

Besides common phage machinery proteins, this phage also contains genes that can be correlated with bacterial fitness and survival in the environment, such as the large protein from the SAM radical family. This family of proteins is implied in the cleavage of unreactive C-H bonds that could be involved in a great range of metabolic processes. Its previous identification in a lineage of phages that target the food-borne pathogen *Campylobacter* has been suggested to favor the host through the enhancement of its metabolism [35]. It also contains proteins that are related to diverse cellular mechanisms such as cell signaling and quorum sensing.

In order to identify this phage, we used the whole-genome based taxonomy system [23], called the phage proteomic tree. This comparison is based on a BLASTP distance matrix, and CLUSTALW and PROTDIST distance scores. As shown in the reference 23, it is efficient in reproducing much of the biological information from the traditional classification by the International Committee on Taxonomy of Viruses (ICTV) and even clusters phages that are known to be related but were previously classified in different groups due to inconsistencies of phenetic and biological data.

A complete phage proteomic tree containing 733 double-stranded DNA phages can be seen in Figure 4. The taxons are colored according to their ICTV classification. A polar tree layout where taxon names can be seen is also available in Supporting Figure S1. In the phage proteomic tree, this phage showed similarity with the Podoviridae genus called the LUZ24-like viruses [36]. This genus is composed of two species of phages known to infect Pseudomonas: LUZ24 and PaP3. The pair-wise identity of the two species that belong to the LUZ24-like genus is 72.4%; meanwhile the identity of these with the IEC224 phage is 50.0%. This is consistent with the average identity of phages from the Podoviridae genus [36].

A genomic BLAST search in the NCBI revealed the genome of this phage in the INDRE91/1 strain with 99% of identity. In order to determine if this phage is prevalent in the *V. cholerae* Latin America epidemic strains, we performed a PCR targeting three genes of the phage's genome: glycosyl hydrolase, phage RNA polymerase, and phage deoxyribonucleoside kinase. Strains from Guyana, Peru, and nine States of Brazil, isolated from 1991 to 2004, were positive for the phages genes. Conversely, strains from Asia and Africa, as well environmental NAGs, were negative (Supporting Text S2).

The homologous genes found and the fact that this phage was inserted in the alanine aminopeptidase gene and present in all the Latin American strains so far tested, lead us to the conclusion that it is same genomic island identified as WASA1 by Mutreja et al. (2011) [13]. To confirm whether it was indeed present in the Latin American sequences reported by that study, we assembled their genomes that are available as raw data at the ENA databank. By this, we could confirm the presence of the same phage in all of these strains. We then aligned all these regions and identified ten different variants that differed with SNPs in only 7 positions (figure 5). The most abundant was variant A, present in strain A5 (Angola 1989). Interestingly, variant A was not found in any strain after 1992, suggesting that all these strains accumulated at least one SNP during the first year that the epidemic spread across Latin American (Figure 5).

Our results so far-in accordance to the previous report of the WASA1 element (13)-suggest that this phage is linked with the Latin American epidemic lineage, and that it was a feature present in the ancestor of the epidemic. Both facts strengthen the already suggested monophyletic and African origin hypothesis for the 1991 Latin American cholera epidemic [11]–[13].

All of the strains that we analyzed so far, harbor this phage and an altered VSP-II that contains two subunits of a transposase. These two elements characterize the Latin American EL Tor lineage.

A BLAST search also revealed that a similar phage was present in two other *Vibrio* species-*V. vulnificus*, strain MO6 24/O, was isolated from a patient that had eaten sea-food from the Atlantic Ocean, and *V. parahaemolyticus*, strain AN-5034 from South-East-Asia. In both of these species, the phage was also inserted in the alanine aminopeptidase gene, with a very similar *att* site. The comparison of their phages genome with the one found in the IEC224 strain can be seen in figure 6.

### Sucrose fermenting phenotype

Because this strain fails to ferment sucrose, we screened all of the 20 *V. cholerae* genes related to the starch and sucrose metabolism as annotated by the Kyoto Encyclopedia of Genes and Genomes (KEGG). *V. mimicus*, which does not ferment sucrose, has a deletion of two genes, corresponding to the sucrose operon repressor (*srcR*) and the sucrose-6-phosphate dehydrogenase [37]. All the 20 genes are intact in the IEC224 genome, and the only difference is a single nucleotide insertion in the gene encoding the sucrose-specific second domain (IIB) of the phosphoenolpyruvate-dependent sugar transport system. This protein is an essential component for the sucrose metabolism because it selectively transports sucrose to the intracellular medium and phosphorylates it, so it can be further metabolized by the sucrose-6-hydrolase enzyme into α-D-glucose-6p and β-D-fructose. This insertion was confirmed by over 40 reads of both third generation sequencing technologies used.

This insertion truncates the protein sequence by introducing a stop-codon at amino-acid residue 185. Through the amino-acid sequence of the functioning N16961 protein it was possible to determine a most probable model for the protein structure and its transmembrane domains, based on fragments of proteins that have been solved by crystallography [25]. According to this *in silico* structure prediction, the sucrose-specific domain consists of a protein with ten predicted transmembrane domains, formed by a structure that contains 23 α-helices (Figure 7). This structure forms a pore that crosses the cell membrane and has an intracellular active site on cysteine residue 26. Because this is a metabolically essential membrane protein, its molecular structure, size, transmembrane motifs, amino-acid charge, isoelectric point and hydrophobicity distribution are conserved among virtually all *V. cholera*e strains, with the exception of a small amino-acid variation illustrated by the amino-acid alignment (Figure 8). This insertion changes the protein size, structure and transmembrane motifs, as well as the the loss of the pore conformation (as shown in Figure 7) and are the probable cause for the non-sucrose-fermenting phenotype in IEC224.

### Concluding Remarks

This paper is the first genome-wide study of the defective sucrose fermenting strain of *V. cholerae* O1 from the Brazilian Cholera Epidemic. In this, we compared the putative mobile regions of *V. cholerae* strains to the non-mobile regions, proposing potentially novel functional roles for these regions and describing their gene composition and distribution in functional subsystems. Moreover, we suggested a novel approach to determine the possible role of hypothetical proteins. With this approach, many possible functions were suggested for the nearly 20% of the genome that commonly used approaches fail to find any homology. The suggested distribution of gene functions opens new research possibilities for the understanding of genomic components that have not yet been described in this species.

Our results clarify the genomic singularities of the *V. cholerae* O1 from Latin American Cholera epidemic, and reinforce the monophyletic hypothesis of this epidemic. One of the genomic island, previously denoted WASA1, was revealed to be the genome of a bacteriophage that harbors a diversity of genes common to phage machinery, and genes that can be correlated with bacterial fitness and survival in the environment, and present in all Latin American strains. Finally, it was possible to show the genetic mechanism behind the altered sucrose phenotype that predominated in the two last seasonal outbreaks of the Brazilian Amazon cholera epidemic. A frame-shift mutation in gene that codes for the sucrose transporter altered the transmembrane-pore-like structure of this protein and, as a consequence, led to the loss of function.

## Supporting Information

Text S1 **Epidemiological context in which the IEC224 strain was isolated.**
(PDF)

Text S2 **Identification of the Vibrio cholerae Latin American epidemic phage in a global scale by PCR.**
(PDF)

Table S1 **Assembly statistics of the V. cholerae genomes from the European Nucleotide Archive.**
(PDF)

Table S2 **Predicted protein function of mobile genomic elements.**
(PDF)

Table S3 **Annotation of protein functions by the Real Time Metagenomics Portal.**
(PDF)

Table S4 **Predicted functions of the hypothetical proteins of the IEC224** ***V. cholerae*** **strain.**
(PDF)

Table S5 **Comparison of the allelic composition between the strains IEC224, INDRE 91/11, MO10, and N16961.**
(PDF)

Figure S1 **Phage Proteomic Tree with the IEC224's phage highlighted.**
(PDF)
