# Supplementary material for: Genome-Wide Study of the Defective Sucrose Fermenter Strain of Vibrio cholerae from the Latin American Cholera Epidemic
Source: PLoS One. 2012 May 25;7(5):e37283. doi: 10.1371/journal.pone.0037283 (PMC3360680; doi:10.1371/journal.pone.0037283)
Supplement: Text S1 — Epidemiological context in which the IEC224 strain was isolated. (PDF) [file pone.0037283.s001.pdf]

Genome-Wide Study of the Defective Sucrose Fermenter Strain of *Vibrio cholerae* from the Latin American Cholera Epidemic

(Garza DR, Thompson CC, Loureiro ECB, Dutilh BE, Inada DT, et al.)

Text S1

Epidemiological Context in which the IEC224 Strain was Isolated

The 7th cholera pandemic started in 1961, and reached Latin America in 1991. It was first reported in Peru on January 29, and a further investigation pointed the city of Chimbote as its most probable starting-point (1). No clear evidence of importation was identified, and the exact introductory event remains a mystery (2).

Within one month after the first cases had been reported in Peru, sixty six patients died from cholera and 1,859 persons were hospitalized with gastroenteritis (3). The initial spread was through the Peruvian Pacific coast, reaching the city of Tumaco (Colombia) in March of the same year (4). It also spread inland through the Andes, as well as through the Peruvian Amazon, and by April it had reached most of Peru. Meanwhile in Colombia 16,800 cases, with 291 deaths were reported in 1991(5).

By April of 1991 the first Brazilian patients with cholera were reported at the city of Leticia (Colombia), border with the Brazilian city of Benjamin Costant, at the Western Amazon Region (6). Benjamin Costant also shares a border with the Peruvian city of Íquitos that was also afflicted by the cholera epidemic. From this double border (Colombia and Peru) the epidemic reached Brazil, spreading quickly through the travel routes of the Amazon River. During the years of 1991-2001 Brazil officially reported 168.598 cases, and 2.037 deaths. By 1996 cholera had already reached most of the Brazilian regions. The majority of cases occurred in the Northeast (7, 8).

In the Amazon, birth place of the Brazilian epidemic, cholerae exhibited a seasonal pattern, with outbreaks in the rainy season - December through March. The highest number of cases was reported in 1992, with the proportion of 40.7 cases per 100.000 inhabitants. The State of Amapá was the most afflicted in the region, exhibiting a rate of 111.3 cases per 100.000 inhabitants (9). After 1992 the epidemic declined, and by 1996 cholera was only observed in the western region of the Amazon. At the late epidemic period (1994), in the state of Amapá, a cholera isolate that failed to ferment sucrose was observed, possibly coming from the French Guiana. This strain quickly spread, and was first thought to be a *Vibrio mimicus* that was causing epidemic cholera. Nevertheless it was later confirmed to be an O1 El Tor *Vibrio cholerae*, with an altered sucrose-fermenting phenotype (10). Multi Locus Enzyme Electrophoresis (MLEE) later showed that this strain belonged to the same zymovar as the Latin American epidemic lineage, suggesting that this variation was the consequence of a mutation in the sucrose operon (11).

This variant remained restricted to the North of Brazil, predominating the cholera cases in the last seasonal outbreaks of cholera during the years of 1994 and 1995. Six hundred and forty four cases were reported as caused by this variant, accounting for 80.4% of the late cholera cases. The IEC224 strain was isolated from one of these confirmed cases in the city of Belém, in the year of 1994.

Bibliography

1) Tauxe R, Seminario L, Tapia R, Libel M (1994). The Latin American Epidemic. In Wachsmuth, Blake, Olsvik, Editors. *Vibrio cholerae* and cholera. Washington D.C.: American Society of Microbiology. pp. 321-344.

2) Seas C, Miranda J, Gil, Leon-Barua R, Patz J, *et al.* (2000). New insights on the emergence of cholera in Latin America during 1991: the Peruvian experience. American Journal of Tropical Medicine and Hygiene, 62(4), 513-517.

3) CDC (1991). International notes – Peru, 1991. Morbidity and Mortality Weekly Report. Available: http://www.cdc.gov/mmwr/preview/mmwrhtml/00001912.htm via the internet. Accessed 15 Nov 2011.

4) Tamayo M, Koblavi S, Grimont F, Castañeda E, Grimont PA (1997). Molecular epidemiology of *Vibrio cholerae* O1 isolates from Colombia. Journal of Medical Microbiology, 46(7), 611-6116.

5) WHO (1997). Epidemiological Bulletin – Cholera situation in the Americas, 1996. Pan American Health Organization, 18 (1). Available: http://www.paho.org/english/sha/epibul_95-98/be971cho.htm#colombia via the internet. Accessed 15 Nov 2011.

6) Silverstein K (1991). Brazil faces cholera threat. The Telegraph. Available: http://news.google.com/newspapers?nid=2209&dat=19910418&id=AYVKAAAAIBAJ&sjid=55MMAAAAIBAJ&pg=5295,3486150 via the internet. Accessed 15 nov 2011.

7) Gerolomo M, Penna MLF (1999). The seventh pandemy of cholera in Brazil – the first five years. Informe Epidemiológico do SUS, 8(3), 49-58. Available: http://bvsms.saude.gov.br/bvs/periodicos/informe_epi_sus_v08_n3.pdf via the internet. Accessed 15 Nov 2011.

8) Vicente ACP, Coelho AM (2005). 1990s *Vibrio cholerae* epidemic, Brazil. Emerging Infectious Diseases. 11 (1), 171-172.

9) Waldman EA, Antunes JLF, Nichiata LYI, Takahashi RF, Cacavallo RC (2002). Cholera in Brazil during 1991-1998: socioeconomic characterization of affected areas. Journal of Health, Population and Nutrition, 20(1), 85-92.

10) Ramos F, Lins-Lainson ZC, Silva EL, Poetti A, Mareco M, *et al.* (1997). Cholera in North Brazil: on the Occurence of Strains of *Vibrio cholerae* O1 which Fail to Ferement Sucrose During Routine Plating on Thiosulphate - Citrate - Bile Salt - Sucrose Agar (TCBS). A New Problem in Diagnosis and Control?. Latin American Microbiology, 39, 141-144.

11) Freitas FS, Momen H, Salles CA (2002). The Zymovars of *Vibrio cholerae*: Multilocus Enzyme Electrophoresis of *Vibrio cholerae*. Memórias do Instituto Oswaldo Cruz, 97(4), 511-516.
