# Supplementary material for: Genome-Wide Study of the Defective Sucrose Fermenter Strain of Vibrio cholerae from the Latin American Cholera Epidemic
Source: PLoS One. 2012 May 25;7(5):e37283. doi: 10.1371/journal.pone.0037283 (PMC3360680; doi:10.1371/journal.pone.0037283)
Supplement: Text S2 — Identification of the Vibrio cholerae Latin American epidemic phage in a global scale by PCR. (PDF) [file pone.0037283.s002.pdf]

Genome-Wide Study of the Defective Sucrose Fermenter Strain of *Vibrio cholerae* from the Latin American Cholera Epidemic

(Garza DR, Thompson CC, Loureiro ECB, Dutilh BE, Inada DT, et al.)

Text S2

Identification of the *Vibrio cholerae* Latin American epidemic Phage in a Global Scale by PCR

A wide screening of strains that were available in our collections show that the Latin American epidemic phage described in the main text is indeed a distinctive marker of the epidemic lineage. El Tor strains from non-Latin American countries did not contain this phage, nor did the NAGs that we tested so far. On the other hand, all the Latin American epidemic isolates are positive for the four primers that were tested. Results considered positive were confirmed by sequencing in ABI3130 (Applied Biosystems). The results are shown in table 1 and the primers used are shown in table 2. Figure 1 contains the electrophoretic profiles of the primers tested.

Detection of the Latin American epidemic phage in strains of *V. cholerae*

Strain	Serotype	Biotype	Source	Country/State/City	Year	Latin American Epidemic Phage[a]
IEC224	O1	El Tor	Clinical	Brazil /Pará/Belém	1994	present
IEC1201	O1	El Tor	Clinical	Brazil/Amapá/Macapá	1994	present
IEC241	O1	El Tor	Clinical	Brazil/Pará/Almeirim	1994	present
IEC200	O1	El Tor	Clinical	Brazil/Pará/Óbidos	1995	present
IEC1133	O1	El Tor	Clinical	Brazil/Pará/Bagre	1994	present
IEC1120	O1	El Tor	Clinical	Brazil/Pará/Igarapé Mirim	1994	present
IEC1082	O1	El Tor	Clinical	Brazil/Amapá/Macapá	1995	present
IEC183	O1	El Tor	Clinical	Brazil		present
IEC1127	O1	El Tor	Clinical	Brazil/Pará/Breves	1994	present
IEC20	O1	El Tor	Clinical	Brazil/Pará/Breves	1994	present
IEC1153	O1	El Tor	Clinical	Brazil/Amapá/Macapá	1994	present
IEC186	O1	El Tor	Clinical	Brazil/Amapá/Macapá	1994	present
IEC28	O1	El Tor	Clinical	Brazil/Pará/Bagre	1994	present
IEC1123	O1	El Tor	Clinical	Brazil/Pará/Cametá	1994	present
IEC21	O1	El Tor	Clinical	Brazil/Pará/Igarapé Mirim	1994	present
IEC1148	O1	El Tor	Clinical	Brazil/Amapá/Macapá	1994	present
IEC1136	O1	El Tor	Clinical	Brazil/Pará/Almeirim	1994	present
IEC1114	O1	El Tor	Clinical	Brazil/Amapá/Macapá	1995	present
IEC1126	O1	El Tor	Clinical	Brazil/Amapá/Vigia	1994	present
IEC1205	O1	El Tor	Clinical	Brazil/Amapá/Macapá	1994	present
VC20	O1	El Tor	Clinical	Brazil/Ceará	1993	Present

VC135	O1	El Tor	Clinical	Brazil/Rio Grande do Sul	1993	Present
VC153	O1	El Tor	Clinical	Brazil/Amazonas	1991	Present
VC153	O1	El Tor	Clinical	Brazil/Amazonas	1994	Present
VC592	O1	El Tor	Clinical	Brazil/Paraná	1999	Present
	O1	El Tor	Clinical	Brazil/Paraná	1999	Present
	O1	El Tor	Clinical	Brazil/Rio de Janeiro	1993	present
	O1	El Tor	Clinical	Brazil/Rio de Janeiro	1994	Present
	O1	El Tor	Clinical	Brazil/Bahia	1995	Present
VC504	O1	El Tor	Clinical	Guyana	1994	Present
VC508	O1	El Tor	Clinical	Guyana	1994	Present
VC625	O1	El Tor	Clinical	Peru	1998	Present
VC627	O1	El Tor	Clinical	Peru	1998	Present
IOC17810	01	El Tor	Superficial water	Brazil/Pernambuco/Machadeiro	2004	Present
IOC17859	O1	El Tor	Superficial water	Brazil/Pernambuco/São Bento	2004	Present
IOC17866	O1	El Tor	Superficial water	Brazil/Alagoas	2002	Present
IOC17935	O1	El Tor	Superficial water	Brazil/Pernambuco	2004	Present
V.621	O1	El Tor	Superficial water	Brazil/Pará/Barcarena	1991	Present
V.412	O1	El Tor	Wastewater	Brazil/Pará/Barcarena	1993	Present
V.780	O1	El Tor	Superficial water	Brazil/Acre/Rio Branco	1993	Present
V.778	O1	El Tor	Superficial water	Brazil/Acre/Santa Rosa	1993	Present
V.482	O1	El Tor	Wastewater	Brazil/Pará/Belém	1994	Present
V.1125	O1	El Tor	Wastewater	Brazil/Amapá/Macapá	1991	Present
V.733	O1	El Tor	Superficial water	Brazil/Amazonas/Tabatinga	1992	Present
V.736	O1	El Tor	Superficial water	Brazil/Amazonas/Tabatinga	1992	Present
V.794	O1	El Tor	Superficial water	Brazil/Amapá/Oiapoque	1994	Present
V.737	O1	El Tor	Superficial water	Brazil/Amazonas/Tabatinga	1992	Present
V.613	O1	El Tor	Superficial water	Brazil/Amapá/Macapá	1991	Present
V.548	O1	El Tor	Wastewater	Brazil/Pará/Belém	1994	Present
V.725	O1	El Tor	Superficial water	Brazil/Pará/Belém	1992	Present
V.485	O1	El Tor	Wastewater	Brazil/Pará/Belém	1994	Present
V.734	O1	El Tor	Superficial water	Brazil/Amazonas/Tabatinga	1992	Present
V.1124	O1	El Tor	Superficial water	Brazil/Amapá/Macapá	1991	Present
LMA1053	NAG		Superficial water	Brazil/Pará/Belém	1999	Absent
LMA1043	NAG		Superficial water	Brazil/Pará/Belém	1999	Absent
LMA1032	NAG		Superficial water	Brazil/Pará/Belém	1999	Absent
LMA1018	NAG		Superficial water	Brazil/Pará/Belém	1999	Absent
LMA1026	NAG		Superficial water	Brazil/Pará/Belém	1999	Absent
LMA620-4	NAG		Superficial water	Brazil/Pará/Belém	1999	Absent
LMA1056	NAG		Superficial water	Brazil/Pará/Belém	1999	Absent
LMA1051	NAG		Superficial water	Brazil/Pará/Belém	1999	Absent
LMA1027	NAG		Superficial water	Brazil/Pará/Belém	1999	Absent
LMA1040	NAG		Superficial water	Brazil/Pará/Belém	1999	Absent
LMA1083	NAG		Superficial water	Brazil/Pará/Belém	1999	Absent
LMA1041	NAG		Superficial water	Brazil/Pará/Belém	1999	Absent
LMA1065	NAG		Superficial water	Brazil/Pará/Belém	1999	Absent
LMA1027	NAG		Superficial water	Brazil/Pará/Belém	1999	Absent
LMA1030	NAG		Superficial water	Brazil/Pará/Belém	1999	Absent
LMA1013	NAG		Superficial water	Brazil/Pará/Belém	1999	Absent
LMA1042	NAG		Superficial water	Brazil/Pará/Belém	1999	Absent
LMA1080	NAG		Superficial water	Brazil/Pará/Belém	1999	Absent
LMA1022	NAG		Superficial water	Brazil/Pará/Belém	1999	Absent
LMA1066	NAG		Superficial water	Brazil/Pará/Belém	1999	Absent
LMA1069	NAG		Superficial water	Brazil/Pará/Belém	1999	Absent
LMA1029	NAG		Superficial water	Brazil/Pará/Belém	1999	Absent
LMA1024	NAG		Superficial water	Brazil/Pará/Belém	1999	Absent
LMA1028	NAG		Superficial water	Brazil/Pará/Belém	1999	Absent
LMA541-1	NAG		Superficial water	Brazil/Pará/Belém	2000	Absent
LMA519-1	NAG		Superficial water	Brazil/Pará/Belém	2000	Absent
LMA620-4	NAG		Superficial water	Brazil/Pará/Belém	2000	Absent
LMA519-4	NAG		Superficial water	Brazil/Pará/Belém	2000	Absent
LMA407-2	NAG		Superficial water	Brazil/Pará/Belém	2000	Absent
LMA408-2	NAG		Superficial water	Brazil/Pará/Belém	2000	Absent
LMA518-3	NAG		Superficial water	Brazil/Pará/Belém	2000	Absent
LMA407-4	NAG		Superficial water	Brazil/Pará/Belém	2000	Absent
LMA634-1	NAG		Superficial water	Brazil/Pará/Belém	2000	Absent
LMA407-5	NAG		Superficial water	Brazil/Pará/Belém	2000	Absent

LMA408-3	NAG		Superficial water	Brazil/Pará/Belém	2000	Absent
LMA519-5	NAG		Superficial water	Brazil/Pará/Belém	2000	Absent
LMA8865-4	NAG		Superficial water	Brazil/Pará/Belém		Absent
LMA4411	NAG		Superficial water	Brazil/Pará/Belém		Absent
LMA7766-4	NAG		Superficial water	Brazil/Pará/Belém		Absent
LMA3984-3	NAG		Superficial water	Brazil/Pará/Belém	2007	Absent
LMA7766-3	NAG		Superficial water	Brazil/Pará/Belém		Absent
8865-2	NAG		Superficial water	Brazil/Pará/Belém		Absent
VC201	O1	classical	Clinical	Pakistan	1986	Absent
VC200	01	classical	Clinical	Pakistan	1986	Absent
VC216	O1	classical	Clinical	Bangladesh	Unk.	Absent
VC04	O1	classical	Clinical	India	Unk.	Absent
VC27	O1	classical	Clinical	India	1971	Absent
VC174	01	classical	Clinical	India	Unk.	Absent
VC33	O1	El Tor	Clinical	Tanzania	1979	Absent
VC79	O1	El Tor	Clinical	Nigeria	1971	Absent
VC104	O1	El Tor	Clinical	Algeria	1972	Absent
VC107	O1	El Tor	Clinical	Ghana	1980	Absent
VC500	O1	El Tor	Clinical	Rwanda	1993	Absent
VC998	O1	El Tor	Clinical	Nigeria	2006	Absent

[a]All positive results, showed amplification for the three primers that were tested with the expected molecular weight, and were further confirmed by sequencing. All negative results, exhibited no amplification.

Primers used in this study

Primer	Amplified Gene	Forward Sequence (5´-3´)	Reverse Sequence (5´-3´)	Expected Band
laep I	Glycosyl hydrolase	CTTCCGGCGTCATTGGGCGT	CGGTGTAGCGGGTGACGTGG	853
laep II	RNA polymerase	AGCGTGTTCACGGTCAGCCT	GCGCGTCAACGCTCGGTAGA	781
laep III	Phage deoxyribonucleoside kinase	CCGCTTGAATCAACCACGGTCACA	CGAGGGAAACATTGGGGCGGG	486

Electrophoretic profile of the PCR reactions for the detection of the Latin American epidemic phage

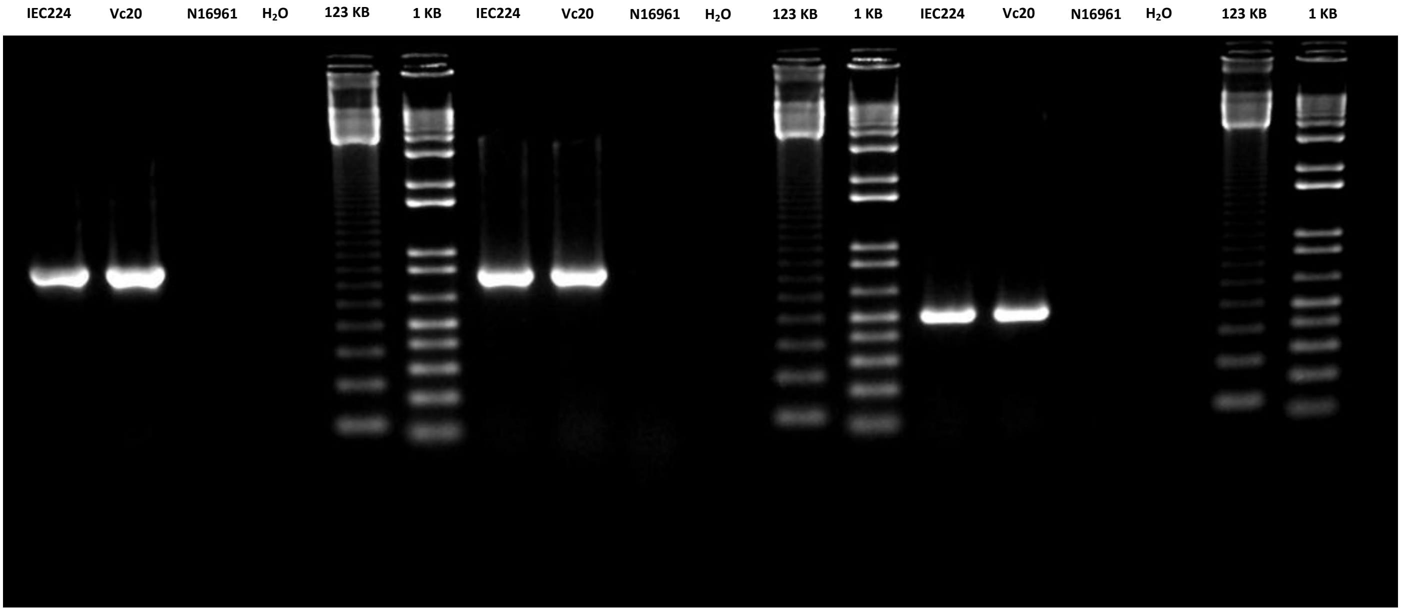

Comparison of PCR results for the three primers used in the detection of the Latin American epidemic phage – from left to right: LAEP I, LAEP II, and LAEP III (see table above). As examples of positive amplifications, we used the IEC224 strain and the VC20 strain that´s a clinical epidemic isolate from the state of Ceará (Brazil). As examples of negative results we used the N16961 strain from Bangladesh, and a reaction with water instead of DNA. All DNA concentrations used were the same (200 ng per reaction), as well as all the other components of the PCR reaction.
