# Supplementary material for: Genome-Wide Study of the Defective Sucrose Fermenter Strain of Vibrio cholerae from the Latin American Cholera Epidemic
Source: PLoS One. 2012 May 25;7(5):e37283. doi: 10.1371/journal.pone.0037283 (PMC3360680; doi:10.1371/journal.pone.0037283)
Supplement: Table S1 — Assembly statistics of the V. cholerae genomes from the European Nucleotide Archive. (PDF) [file pone.0037283.s003.pdf]

Genome-Wide Study of the Defective Sucrose Fermenter Strain of *Vibrio cholerae* from the Latin American Cholera Epidemic

(Garza DR, Thompson CC, Loureiro ECB, Dutilh BE, Inada DT, et al.)

Table S1

Assembly Statistics of V. cholerae Genomes from the European Nucleotide Archive

Accession	Strain	Year	Country	N_reads	N_contigs	N50_contigs	Longest_contig	Scaffolding_reference	N_scaffolds	N50_scaffolds	Longest_scaffold	Genome_length
**ERS013174**	A184	1992	Colombia	1833652	2952	2602	14222	MJ-1236	291	2980010	2980010	4165955
**ERS013175**	A185	1992	Colombia	2072516	2534	3212	16530	MJ-1236	325	2972139	2972139	4191262
**ERS013182**	A177	1992	Colombia	7167324	1855	6741	39181	M66-2	685	2915601	2915601	4153428
**ERS013183**	A180	1992	Colombia	1710970	3447	2208	11680	MJ-1236	334	2992124	2992124	4206204
**ERS013184**	A186	1992	Argentina	2973452	3004	2657	20635	MJ-1236	479	2977904	2977904	4176053
**ERS013185**	A193	1992	Bolivia	4001314	2710	3181	35999	MJ-1236	574	2975112	2975112	4168318
**ERS013186**	A232	1991	Mexico	1701326	5677	1199	8413	M66-2	546	2921270	2921270	4157983
**ERS013188**	A200	1992	Argentina	4238722	2722	3341	37443	M66-2	719	2910734	2910734	4150422
**ERS013189**	A201	1992	Argentina	2578524	3231	2302	15350	M66-2	540	2892135	2892135	4117826
**ERS013195**	A231	1991	Mexico	2693380	3222	2384	12381	MJ-1236	446	2981777	2981777	4173103
**ERS013200**	A316	1993	Argentina	2156650	6320	1079	7141	MJ-1236	623	2989262	2989262	4338837
**ERS013251**	A27	1991	Peru	6144342	700	33872	108325	MJ-1236	187	3018148	3018148	4269236
**ERS013274**	A29	1991	Peru	7414754	724	32731	108326	MJ-1236	213	3004715	3004715	4270042
**ERS013275**	A31	1991	Peru	13285864	762	25330	106346	MJ-1236	202	2994622	2994622	4155495
**ERS013276**	A32	1991	Peru	8286426	724	30292	108590	N16961	169	3009718	3009718	4234232
**ERS013245**	A5	1989	Angola	7244590	749	27905	108320	MJ-1236	215	2999588	2999588	4208358
