# Supplementary material for: Genome-Wide Study of the Defective Sucrose Fermenter Strain of Vibrio cholerae from the Latin American Cholera Epidemic
Source: PLoS One. 2012 May 25;7(5):e37283. doi: 10.1371/journal.pone.0037283 (PMC3360680; doi:10.1371/journal.pone.0037283)
Supplement: Table S2 — Predicted protein function of mobile genomic elements. (PDF) [file pone.0037283.s004.pdf]

Genome-Wide Study of the Defective Sucrose Fermenter Strain of *Vibrio cholerae* from the Latin American Cholera Epidemic

(Garza DR, Thompson CC, Loureiro ECB, Dutilh BE, Inada DT, et al.)

Table S2

Predicted Protein Functions of Putative Mobile Genomic Regions		
#	Function	Mobile Element
1	Quaternary ammonium compound-resistance protein SugE	GI -I
2	COG0840: Methyl-accepting chemotaxis protein	GI –I
3	Chemotaxis regulator - transmits chemoreceptor signals to flagelllar motor components CheY	GI –I
4	FIG01200770: hypothetical protein	GI –I
5	Signal transduction histidine kinase CheA (EC 2.7.3.-)	GI –I
6	Chemotaxis regulator - transmits chemoreceptor signals to flagelllar motor components CheY	GI –I
7	Chemotaxis protein methyltransferase CheR (EC 2.1.1.80)	GI –I
8	FOG: HEAT repeat	GI –I
9	Chemotaxis response regulator protein-glutamate methylesterase CheB (EC 3.1.1.61)	GI –I
10	Positive regulator of CheA protein activity (CheW)	GI –I
11	COG0840: Methyl-accepting chemotaxis protein	GI –I
12	hypothetical protein	GI –I
13	methyl-accepting chemotaxis protein	GI -I
14	Membrane-fusion protein	GI – 2
15	Type I secretion outer membrane protein, TolC precursor	GI – 2
16	ABC-type antimicrobial peptide transport system, permease component	GI – 2
17	ABC-type antimicrobial peptide transport system, permease component	GI – 2
18	Macrolide export ATP-binding/permease protein MacB (EC 3.6.3.-)	GI – 2
19	hypothetical protein	GI – 3
20	FIG01201212: hypothetical protein	GI – 3
21	Outer membrane protein/protective antigen OMA87	GI – 3
22	corresponds to STY3950 from Accession AL513382: Salmonella typhi CT18	GI – 3
23	Small-conductance mechanosensitive channel	GI – 3
24	Paraquat-inducible protein A	GI – 3
25	Aldehyde dehydrogenase (EC 1.2.1.3)	GI – 4
26	PTS system, fructose-specific IIA component (EC 2.7.1.69) / PTS system, fructose-specific IIB component (EC 2.7.1.69) / PTS system, fructose-specific IIC component (EC 2.7.1.69)	GI – 4
27	PTS system, fructose-specific IIA component (EC 2.7.1.69) / PTS system, fructose-specific IIB component (EC 2.7.1.69) / PTS system, fructose-specific IIC component (EC 2.7.1.69)	GI – 4

**28**	PTS system, fructose-specific IIB component (EC 2.7.1.69)	GI – 4
**29**	Putative PTS system, nitrogen regulatory IIA component	GI – 4
**30**	Transcriptional regulator, AraC family	GI – 4
**31**	GMP reductase (EC 1.7.1.7)	GI - 5
**32**	hypothetical protein	GI – 5
**33**	DNA-cytosine methyltransferase (EC 2.1.1.37)	GI – 5
**34**	FIG01202881: hypothetical protein	GI – 5
**35**	hypothetical protein	GI – 5
**36**	FIG01200375: hypothetical protein	GI – 5
**37**	IS1004 transposase	GI – 5
**38**	ATP-dependent RNA helicase RhlE	GI – 5
**39**	Aminomethyltransferase (glycine cleavage system T protein) (EC 2.1.2.10)	GI - 6
**40**	Phage Integrase	GI – 6
**41**	IS5 transposase	GI – 6
**42**	hypothetical protein	GI – 6
**43**	Uropathogenic specific protein	GI – 6
**44**	FIG01203297: hypothetical protein	GI – 6
**45**	hypothetical protein	GI – 6
**46**	Threonyl-tRNA synthetase (EC 6.1.1.3)	GI – 6
**47**	L-serine dehydratase (EC 4.3.1.17)	GI - 7
**48**	Pantothenate:Na+ symporter (TC 2.A.21.1.1)	GI – 7
**49**	4-hydroxybenzoate transporter	GI – 7
**50**	FIG01200201: hypothetical protein	GI – 7
**51**	Transcriptional regulator, LacI family	GI – 7
**52**	Nitrate/nitrite response regulator protein	GI – 7
**53**	FIG01199690: hypothetical protein	GI – 8
**54**	Putative sodium-glucose/galactose cotransporter	GI – 8
**55**	COG0582: Integrase	GI – 8
**56**	transposase OrfAB, subunit B	GI – 8
**57**	Transposase	GI – 8
**58**	hypothetical protein	GI – 8

**59**	hypothetical protein	GI – 8
**60**	resolvase, putative	GI – 8
**61**	RTX toxins and related Ca2+-binding proteins	GI – 9
**62**	Response regulator	GI – 9
**63**	hypothetical protein	GI – 9
**64**	Autolysin sensor kinase (EC 2.7.3.-)	GI – 9
**65**	FIG01201672: hypothetical protein	GI – 9
**66**	FIG01201095: hypothetical protein	GI – 9
**67**	Lipoprotein releasing system transmembrane protein LolC	GI – 9
**68**	ABC transporter, ATP-binding protein	GI – 9
**69**	FIG01200001: hypothetical protein	GI – 9
**70**	FIG01202943: hypothetical protein	GI – 9
**71**	FIG01201764: hypothetical protein	GI – 9
**72**	Oxidoreductase, aldo/keto reductase family	GI – 9
**73**	Choloylglycine hydrolase (EC 3.5.1.24)	GI - 10
**74**	phage integrase	GI – 10
**75**	FIG01201351: hypothetical protein	GI – 10
**76**	conserved hypothetical protein	GI – 10
**77**	FIG01201312: hypothetical protein	GI – 10
**78**	Non-hemolytic enterotoxin lytic component L1	GI – 10
**79**	Non-hemolytic enterotoxin lytic component L1	GI – 10
**80**	L-threonine 3-dehydrogenase (EC 1.1.1.103)	GI – 10
**81**	hypothetical protein	Latin American epidemic marker phage
**82**	Transcriptional Regulator	Latin American epidemic marker phage
**83**	P-loop NTPase superfamily	Latin American epidemic marker phage
**84**	Possible transcription regulator	Latin American epidemic marker phage
**85**	Peptidase	Latin American epidemic marker phage
**86**	hypotetical protein	Latin American epidemic marker phage
**87**	hypothetical protein	Latin American epidemic marker phage
**88**	Radical SAM protein	Latin American epidemic marker phage
**89**	Tail assembly protein	Latin American epidemic marker phage

**90**	Glycosyl hydrolase	Latin American epidemic marker phage
**91**	Endopeptidase	Latin American epidemic marker phage
**92**	hypotetical protein	Latin American epidemic marker phage
**93**	hypothetical protein	Latin American epidemic marker phage
**94**	phage tail tape meausure protein	Latin American epidemic marker phage
**95**	Transcriptional regulator LysR	Latin American epidemic marker phage
**96**	Phage tail assembly chaperone	Latin American epidemic marker phage
**97**	Major phage tail protein	Latin American epidemic marker phage
**98**	Histidine kinase	Latin American epidemic marker phage
**99**	Conserved phage protein	Latin American epidemic marker phage
**100**	Phage head-tail joining protein	Latin American epidemic marker phage
**101**	Phage head-tail connector protein	Latin American epidemic marker phage
**102**	Major capsid protein	Latin American epidemic marker phage
**103**	Phage head maturation protease	Latin American epidemic marker phage
**104**	Portal Protein	Latin American epidemic marker phage
**105**	Phage terminase 1 large subunit	Latin American epidemic marker phage
**106**	Possibly a restriction enzyme	Latin American epidemic marker phage
**107**	Possibly a serine protease	Latin American epidemic marker phage
**108**	Nucleoside-specific channel forming protein, Tsx	Latin American epidemic marker phage
**109**	Possibly a signaling protein	Latin American epidemic marker phage
**110**	Phage terminase small subunit	Latin American epidemic marker phage
**111**	hypothetical protein	Latin American epidemic marker phage
**112**	hypotetical protein	Latin American epidemic marker phage
**113**	Antirestriction protein (ArdA)	Latin American epidemic marker phage
**114**	Bacterial type III secretion protein	Latin American epidemic marker phage
**115**	hypothetical protein	Latin American epidemic marker phage
**116**	HNH endonuclease	Latin American epidemic marker phage
**117**	Possibly mannose-6-phosphate isomerase	Latin American epidemic marker phage
**118**	hypothetical protein CDS	Latin American epidemic marker phage
**119**	Possibly arginine tRNA ligase	Latin American epidemic marker phage
**120**	hypothetical protein	Latin American epidemic marker phage

121	Phage Deoxyribonucleoside kinase	Latin American epidemic marker phage
122	Hypothetical protein	Latin American epidemic marker phage
123	Phage deoxyribonucleoside kinase	Latin American epidemic marker phage
124	hypothetical protein	Latin American epidemic marker phage
125	Domain of unknown function	Latin American epidemic marker phage
126	Domain of unknown function	Latin American epidemic marker phage
127	hypothetical protein CDS	Latin American epidemic marker phage
128	Phage exonuclease	Latin American epidemic marker phage
129	terB protein	Latin American epidemic marker phage
130	Phage DNA polymerase	Latin American epidemic marker phage
131	Phage primase/helicase	Latin American epidemic marker phage
132	Endodesoxyribonucelase	Latin American epidemic marker phage
133	Phage ssDNA-binding protein	Latin American epidemic marker phage
134	luxR like transcriptional regulator	Latin American epidemic marker phage
135	parB protein	Latin American epidemic marker phage
136	Phage RNA polymerase	Latin American epidemic marker phage
137	T3/T7 like RNA polymerase	Latin American epidemic marker phage
138	Possibly a sensor box GGDEF protein	Latin American epidemic marker phage
139	Phage integrase site-specific recombinase	Latin American epidemic marker phage
140	FIG01200619: hypothetical protein	TLC + CTXphi
141	RTX toxin activating lysine-acyltransferase (EC 2.3.1.-)	TLC + CTXphi
142	RTX toxins determinant A and related Ca2+-binding proteins	TLC + CTXphi
143	RstC phage-related antirepressor	TLC + CTXphi
144	RstB phage-related integrase	TLC + CTXphi
145	RstA phage-related replication protein	TLC + CTXphi
146	RstR phage-related transcriptional repressor	TLC + CTXphi
147	Enterotoxin, B subunit	TLC + CTXphi
148	Enterotoxin, A subunit (NAD(+)--diphthamide ADP- ribosyltransferase) (EC 2.4.2.36)	TLC + CTXphi
149	Zona occludens toxin	TLC + CTXphi
150	Accessory cholera enterotoxin	TLC + CTXphi
151	FIG01201937: hypothetical protein	TLC + CTXphi

152	hypothetical protein	VPI-1
153	FIG01202538: hypothetical protein	VPI-1
154	hypothetical protein	VPI-1
155	FIG01199680: hypothetical protein	VPI-1
156	FIG01199680: hypothetical protein	VPI-1
157	Helicase-related protein	VPI-1
158	Tellurite resistance protein-related protein	VPI-1
159	Predicted transcriptional regulator	VPI-1
160	FIG01200656: hypothetical protein	VPI-1
161	hypothetical protein	VPI-1
162	Transposase	VPI-1
163	Transposase	VPI-1
164	Transposase	VPI-1
165	Aldehyde dehydrogenase (EC 1.2.1.3)	VPI-1
166	hypothetical protein	VPI-1
167	Lipoprotein, ToxR-activated gene, TagA	VPI-1
168	inner membrane protein, putative	VPI-1
169	hypothetical protein	VPI-1
170	Thiol peroxidase, Tpx-type (EC 1.11.1.15)	VPI-1
171	Toxin co-regulated pilus biosynthesis protein I, chemoreceptor, negative regulator of TcpA	VPI-1
172	Toxin co-regulated pilus biosynthesis protein P, transcriptional activator of ToxT promoter	VPI-1
173	Toxin co-regulated pilus biosynthesis protein H, transcriptional activator of ToxT promoter	VPI-1
174	Toxin co-regulated pilin A	VPI-1
175	Toxin co-regulated pilus biosynthesis protein B	VPI-1
176	Toxin co-regulated pilus biosynthesis protein Q	VPI-1
177	Toxin co-regulated pilus biosynthesis protein C, outer membrane protein	VPI-1
178	Toxin co-regulated pilus biosynthesis protein R	VPI-1
179	Toxin co-regulated pilus biosynthesis protein D	VPI-1
180	Toxin co-regulated pilus biosynthesis protein S	VPI-1
181	Toxin co-regulated pilus biosynthesis protein T, putative ATP-binding translocase of TcpA	VPI-1
182	Toxin co-regulated pilus biosynthesis protein E, anchors TcpT to membrane	VPI-1

183	Toxin co-regulated pilus biosynthesis protein F, putative outer membrane channel for TcpA extrusion	VPI-1
184	TCP pilus virulence regulatory protein ToxT, transcription activator	VPI-1
185	TCP pilin signal peptidase, TcpA processing	VPI-1
186	Accessory colonization factor AcfB	VPI-1
187	Accessory colonization factor AcfC	VPI-1
188	hypothetical protein	VPI-1
189	COG0739: Membrane proteins related to metalloendopeptidases	VPI-1
190	Accessory colonization factor AcfA	VPI-1
191	Accessory colonization factor AcfD precursor	VPI-1
192	Phage integrase	VPI-1
193	hypothetical protein	VPI-1
194	Phage integrase	VPI-1
195	hypothetical protein	VPI-1
196	Prophage CP4-57 integrase	VPI-1
197	tmRNA-binding protein SmpB	VPI-1
198	Acriflavin resistance protein	VPI-2
199	tRNA-Ser-GGA	VPI-2
200	phage integrase	VPI-2
201	Prophage CP4-57 integrase	VPI-2
202	helicase, putative	VPI-2
203	FIG01200559: hypothetical protein	VPI-2
204	chemotaxis protein MotB-related protein	VPI-2
205	putative membrane protein	VPI-2
206	Type I restriction-modification system, restriction subunit R (EC 3.1.21.3)	VPI-2
207	FIG01200742: hypothetical protein	VPI-2
208	FIG01200429: hypothetical protein	VPI-2
209	Type I restriction-modification system, specificity subunit S (EC 3.1.21.3)	VPI-2
210	Type I restriction-modification system, DNA-methyltransferase subunit M (EC 2.1.1.72)	VPI-2
211	FIG01202070: hypothetical protein	VPI-2
212	FIG01201906: hypothetical protein	VPI-2

213	FIG01201150: hypothetical protein	VPI-2
214	Sialic acid-induced transmembrane protein YjhT(NanM), possible mutarotase	VPI-2
215	Sialic acid-induced transmembrane protein YjhT(NanM), possible mutarotase	VPI-2
216	Sialic acid utilization regulator, RpiR family	VPI-2
217	N-acetylneuraminate lyase (EC 4.1.3.3)	VPI-2
218	TRAP-type transport system, large permease component, predicted N-acetylneuraminate transporter	VPI-2
219	TRAP-type transport system, small permease component, predicted N-acetylneuraminate transporter	VPI-2
220	TRAP-type transport system, periplasmic component, predicted N-acetylneuraminate-binding protein	VPI-2
221	N-acetylmannosamine-6-phosphate 2-epimerase (EC 5.1.3.9)	VPI-2
222	N-acetylmannosamine kinase (EC 2.7.1.60)	VPI-2
223	N-acetylglucosamine-6-phosphate deacetylase (EC 3.5.1.25)	VPI-2
224	Sialidase (EC 3.2.1.18)	VPI-2
225	Predicted transcriptional regulator	VPI-2
226	DNA repair protein RadC	VPI-2
227	FIG01201713: hypothetical protein	VPI-2
228	FIG01203000: hypothetical protein	VPI-2
229	transposase OrfAB, subunit B	VPI-2
230	Transposase	VPI-2
231	Mu-like prophage FluMu protein gp42	VPI-2
232	hypothetical protein	VPI-2
233	Mu-like prophage FluMu protein gp41	VPI-2
234	FIG01199767: hypothetical protein	VPI-2
235	FIG01202257: hypothetical protein	VPI-2
236	transcriptional regulator, putative	VPI-2
237	hypothetical protein	VPI-2
238	hypothetical protein	VPI-2
239	FIG01200588: hypothetical protein	VPI-2
240	General secretion pathway protein A	VPI-2
241	hypothetical protein	VPI-2

242	FIG01200781: hypothetical protein	VPI-2
243	FIG01205904: hypothetical protein	VPI-2
244	FIG01200234: hypothetical protein	VPI-2
245	hypothetical protein	VPI-2
246	conserved hypothetical protein	VPI-2
247	FIG01201713: hypothetical protein	VPI-2
248	FIG01201770: hypothetical protein	VPI-2
249	FIG01200074: hypothetical protein	VPI-2
250	hypothetical protein	VPI-2
251	FIG01202565: hypothetical protein	VPI-2
252	FIG01201490: hypothetical protein	VPI-2
253	Transcriptional regulator, AlpA like	VPI-2
254	FIG01200010: hypothetical protein	VPI-2
255	FIG01203377: hypothetical protein	VSP - I
256	hypothetical protein	VSP – I
257	Putative transcriptional regulator	VSP – I
258	Response regulator	VSP – I
259	hypothetical protein	VSP – I
260	patatin-related protein	VSP – I
261	FIG01202724: hypothetical protein	VSP – I
262	FIG01200176: hypothetical protein	VSP – I
263	FIG01200099: hypothetical protein	VSP – I
264	Phage Integrase	VSP – I
265	FIG01202926: hypothetical protein	VSP – I
266	transposase, putative	VSP – I
267	Glutathione reductase (EC 1.8.1.7)	VSP – I
268	hypothetical protein	VSP – I
269	hypothetical protein	VSP – I
270	Putative hemolysin	VSP - II
271	hypothetical protein	VSP – II
272	hypothetical protein	VSP – II

**273**	hypothetical protein	VSP – II
**274**	FIG01202833: hypothetical protein	VSP – II
**275**	FIG01202149: hypothetical protein	VSP – II
**276**	FIG01199737: hypothetical protein	VSP - II
**277**	FIG01199737: hypothetical protein	VSP – II
**278**	FIG01203010: hypothetical protein	VSP – II
**279**	predicted transcriptional regulator	VSP – II
**280**	Ribonuclease HI, Vibrio paralog	VSP – II
**281**	transposase OrfAB, subunit B	VSP – II
**282**	Transposase	VSP – II
**283**	transposase OrfAB, subunit A	VSP – II
**284**	Transposase	VSP – II
**285**	methylation site containing protein	VSP – II
**286**	high-affinity zinc uptake system protein ZnuA precursor	VSP – II
**287**	Cell wall endopeptidase, family M23/M37	VSP – II
**288**	FIG01201073: hypothetical protein	VSP – II
**289**	FIG01199973: hypothetical protein	VSP – II
**290**	FIG01200607: hypothetical protein	VSP – II
**291**	FIG01202639: hypothetical protein	VSP – II
**292**	FIG01200390: hypothetical protein	VSP – II
**293**	FIG01201167: hypothetical protein	VSP – II
**294**	DNA repair protein RadC	VSP – II
**295**	FIG00938826: hypothetical protein	VSP – II
**296**	hypothetical protein	VSP – II
**297**	FIG00828579: hypothetical protein	VSP – II
**298**	Transposase	VSP – II
**299**	transposase OrfAB, subunit B	VSP – II
**300**	Prophage CP4-57 integrase	VSP – II
**301**	hypothetical protein	VSP – II
**302**	tRNA-Met-CAT	VSP – II
**303**	RNA polymerase sigma factor RpoD	VSP – II

304	Phosphomannomutase (EC 5.4.2.8)	O1 Antigen cluster
305	GDP-mannose 4,6-dehydratase (EC 4.2.1.47)	O1 Antigen cluster
306	Bacillosamine/Legionaminic acid biosynthesis aminotransferase PglE; 4-keto-6-deoxy-N-Acetyl-D-hexosaminyl-(Lipid carrier) aminotransferase	O1 Antigen cluster
307	O-antigen export system, polymerase or transferase	O1 Antigen cluster
308	O-antigen export system, permease protein	O1 Antigen cluster
309	Methionine ABC transporter ATP-binding protein	O1 Antigen cluster
310	acyl carrier protein, putative	O1 Antigen cluster
311	Long-chain-fatty-acid--CoA ligase (EC 6.2.1.3)	O1 Antigen cluster
312	Alcohol dehydrogenase (EC 1.1.1.1); Acetaldehyde dehydrogenase (EC 1.2.1.10)	O1 Antigen cluster
313	acyl protein synthase/acyl-CoA reductase RfbN	O1 Antigen cluster
314	acetyltransferase RfbO, CysE/LacA/LpxA/NodL family	O1 Antigen cluster
315	RfbT protein	O1 Antigen cluster
316	Transposase	O1 Antigen cluster
317	transposase OrfAB, subunit B	O1 Antigen cluster
318	RfbT protein	O1 Antigen cluster
319	Glycosyltransferase	O1 Antigen cluster
320	Nucleoside-diphosphate sugar epimerase/dehydratase	O1 Antigen cluster
321	hypothetical protein	O1 Antigen cluster
322	UDP-glucose 4-epimerase (EC 5.1.3.2)	O1 Antigen cluster
323	Lipid carrier : UDP-N-acetylgalactosaminyltransferase (EC 2.4.1.-)	O1 Antigen cluster
324	FIG01201221: hypothetical protein	O1 Antigen cluster
325	Metallo-beta-lactamase family protein, RNA-specific	O1 Antigen cluster
326	Small-conductance mechanosensitive channel	O1 Antigen cluster
327	Hypothetical protein VC0266 (sugar utilization related?)	O1 Antigen cluster
328	D-3-phosphoglycerate dehydrogenase (EC 1.1.1.95)	O1 Antigen cluster
329	FIG01200173: hypothetical protein	O1 Antigen cluster
330	Mannose-6-phosphate isomerase (EC 5.3.1.8)	O1 Antigen cluster
331	ROK family Glucokinase with ambiguous substrate specificity	O1 Antigen cluster
332	COG1253: Hemolysins and related proteins containing CBS domains	O1 Antigen cluster
