# Supplementary material for: Genome-Wide Study of the Defective Sucrose Fermenter Strain of Vibrio cholerae from the Latin American Cholera Epidemic
Source: PLoS One. 2012 May 25;7(5):e37283. doi: 10.1371/journal.pone.0037283 (PMC3360680; doi:10.1371/journal.pone.0037283)
Supplement: Table S3 — Annotation of protein functions by the Real Time Metagenomics Portal. (PDF) [file pone.0037283.s005.pdf]

Genome-Wide Study of the Defective Sucrose Fermenter Strain of *Vibrio cholerae* from the Latin American Cholera Epidemic

(Garza DR, Thompson CC, Loureiro ECB, Dutilh BE, Inada DT, et al.)

Table S3

Annotation of Protein Functions by the Real-Time Metagenomics Portal

**Amino Acids and Derivatives**	**374**
**Alanine, serine, and glycine**	**33**
Alanine biosynthesis	7
Alanine racemase (EC 5.1.1.1)	1
Cysteine desulfurase (EC 2.8.1.7), IscS subfamily	1
Cysteine desulfurase (EC 2.8.1.7), SufS subfamily	1
Glutamate-pyruvate aminotransferase (EC 2.6.1.2)	1
HTH-type transcriptional regulator IlvY	1
Valine--pyruvate aminotransferase (EC 2.6.1.66)	2
Glycine and Serine Utilization	16
Cystathionine beta-synthase (EC 4.2.1.22)	1
D-3-phosphoglycerate dehydrogenase (EC 1.1.1.95)	1
D-serine dehydratase transcriptional activator	1
D-serine/D-alanine/glycine transporter	1
Glycerate kinase (EC 2.7.1.31)	1
Glycine cleavage system H protein	1
Glycine dehydrogenase [decarboxylating] (glycine cleavage system P protein) (EC 1.4.4.2)	1
Glycine dehydrogenase [decarboxylating] (glycine cleavage system P1 protein) (EC 1.4.4.2)	1
Glycine dehydrogenase [decarboxylating] (glycine cleavage system P2 protein) (EC 1.4.4.2)	1
L-serine dehydratase (EC 4.3.1.17)	1
L-serine dehydratase, alpha subunit (EC 4.3.1.17)	1
L-serine dehydratase, beta subunit (EC 4.3.1.17)	1
Phosphoserine phosphatase (EC 3.1.3.3)	1
Serine transporter	1

Seryl-tRNA synthetase (EC 6.1.1.11)	1
Threonine dehydratase, catabolic (EC 4.3.1.19)	1
Glycine Biosynthesis	1
Low-specificity L-threonine aldolase (EC 4.1.2.5)	1
Glycine cleavage system	6
Glycine cleavage system H protein	1
Glycine cleavage system transcriptional activator GcvA	1
Glycine dehydrogenase [decarboxylating] (glycine cleavage system P protein) (EC 1.4.4.2)	1
Glycine dehydrogenase [decarboxylating] (glycine cleavage system P1 protein) (EC 1.4.4.2)	1
Glycine dehydrogenase [decarboxylating] (glycine cleavage system P2 protein) (EC 1.4.4.2)	1
Sodium/glycine symporter GlyP	1
Serine Biosynthesis	3
D-3-phosphoglycerate dehydrogenase (EC 1.1.1.95)	1
Phosphoserine phosphatase (EC 3.1.3.3)	2
**Arginine; urea cycle, polyamines**	**77**
Anaerobic Oxidative Degradation of L-Ornithine	1
D-Ornithine 4,5-aminomutase S subunit (EC 5.4.3.5)	1
Arginine and Ornithine Degradation	23
Agmatine deiminase (EC 3.5.3.12)	1
Arginine ABC transporter, periplasmic arginine-binding protein ArtI	1
Arginine ABC transporter, permease protein ArtM	1
Arginine N-succinyltransferase (EC 2.3.1.109)	1
Arginine pathway regulatory protein ArgR, repressor of arg regulon	1
Arginine permease RocE	1
Arginine utilization protein RocB	1
Arginine/ornithine ABC transporter, permease protein AotQ	1
Arginine/ornithine antiporter ArcD	1
Carbamate kinase (EC 2.7.2.2)	1
Delta-1-pyrroline-5-carboxylate dehydrogenase (EC 1.5.1.12)	1
Histidine ABC transporter, ATP-binding protein HisP (TC 3.A.1.3.1)	1
Histidine ABC transporter, permease protein HisM (TC 3.A.1.3.1)	1
Histidine ABC transporter, permease protein HisQ (TC 3.A.1.3.1)	1
Lysine-arginine-ornithine-binding periplasmic protein precursor (TC 3.A.1.3.1)	1
NADP-specific glutamate dehydrogenase (EC 1.4.1.4)	1
Ornithine aminotransferase (EC 2.6.1.13)	1
Ornithine carbamoyltransferase (EC 2.1.3.3)	1
Ornithine cyclodeaminase (EC 4.3.1.12)	1

Ornithine decarboxylase (EC 4.1.1.17)	1
Succinylglutamate desuccinylase (EC 3.5.1.96)	1
Succinylglutamic semialdehyde dehydrogenase (EC 1.2.1.71)	1
Transcriptional regulator of catabolic arginine decarboxylase (adiA)	1
Arginine Biosynthesis extended	8
Acetylglutamate kinase (EC 2.7.2.8)	1
Acetylornithine aminotransferase (EC 2.6.1.11)	1
Arginine pathway regulatory protein ArgR, repressor of arg regulon	1
Argininosuccinate lyase (EC 4.3.2.1)	1
Argininosuccinate synthase (EC 6.3.4.5)	1
N-acetyl-gamma-glutamyl-phosphate reductase (EC 1.2.1.38)	1
N-acetylglutamate synthase (EC 2.3.1.1)	1
Ornithine carbamoyltransferase (EC 2.1.3.3)	1
Arginine Deiminase Pathway	4
Arginine pathway regulatory protein ArgR, repressor of arg regulon	1
Arginine/ornithine antiporter ArcD	1
Carbamate kinase (EC 2.7.2.2)	1
Ornithine carbamoyltransferase (EC 2.1.3.3)	1
Cyanophycin Metabolism	2
Asparagine synthetase [glutamine-hydrolyzing] (EC 6.3.5.4)	1
Cyanophycin synthase (EC 6.3.2.29)(EC 6.3.2.30)	1
Polyamine Metabolism	17
ABC transporter, periplasmic spermidine putrescine-binding protein PotD (TC 3.A.1.11.1)	1
Agmatine deiminase (EC 3.5.3.12)	1
Arginine/ornithine antiporter ArcD	1
Carbamate kinase (EC 2.7.2.2)	1
Ornithine decarboxylase (EC 4.1.1.17)	1
Predicted polyamine sensor NspS, involved in biofilm formation	1
Putrescine ABC transporter putrescine-binding protein PotF (TC 3.A.1.11.2)	1
Putrescine transport ATP-binding protein PotA (TC 3.A.1.11.1)	1
Putrescine transport ATP-binding protein PotG (TC 3.A.1.11.2)	1
Putrescine transport system permease protein PotI (TC 3.A.1.11.2)	1
Putrescine/proton symporter, putrescine/ornithine antiporter PotE	1
Putrescine-ornithine antiporter	1
S-adenosylmethionine decarboxylase proenzyme (EC 4.1.1.50), prokaryotic class 1B	1
Spermidine N1-acetyltransferase (EC 2.3.1.57)	1
Spermidine Putrescine ABC transporter permease component PotB (TC 3.A.1.11.1)	1

Spermidine Putrescine ABC transporter permease component potC (TC_3.A.1.11.1)	1
Transcriptional regulator of catabolic arginine decarboxylase (adiA)	1
Putrescine utilization pathways	3
Gamma-glutamyl-putrescine synthetase (EC 6.3.1.11)	1
Glycine/D-amino acid oxidase (deaminating) in putrescine utilization cluster	1
Putrescine importer	1
Urea carboxylase and Allophanate hydrolase cluster	3
Allophanate hydrolase 2 subunit 1 (EC 3.5.1.54)	1
Allophanate hydrolase 2 subunit 2 (EC 3.5.1.54)	1
Urea carboxylase (EC 6.3.4.6)	1
Urea decomposition	11
Nickel transporter UreH	1
Urea ABC transporter, permease protein UrtC	1
Urea ABC transporter, urea binding protein	1
Urea carboxylase (EC 6.3.4.6)	1
Urea carboxylase-related ABC transporter, permease protein	1
Urea carboxylase-related amino acid permease	1
Urease accessory protein UreD	1
Urease accessory protein UreE	1
Urease accessory protein UreF	1
Urease accessory protein UreG	1
Urease alpha subunit (EC 3.5.1.5)	1
Urease subunits	5
Urease accessory protein UreD	1
Urease accessory protein UreE	1
Urease accessory protein UreF	1
Urease accessory protein UreG	1
Urease alpha subunit (EC 3.5.1.5)	1
**Aromatic amino acids and derivatives**	**54**
Aromatic amino acid degradation	9
2,4-dihydroxyhept-2-ene-1,7-dioic acid aldolase (EC 4.1.2.-)	1
5-carboxymethyl-2-hydroxymuconate delta-isomerase (EC 5.3.3.10)	1
5-oxopent-3-ene-1,2,5-tricarboxylate decarboxylase.	1
Aromatic amino acid transport protein AroP	1
Aromatic-L-amino-acid decarboxylase (EC 4.1.1.28)	1
Indoleacetamide hydrolase (EC 3.5.1.-)	1
Kynurenine formamidase, bacterial (EC 3.5.1.9)	1

Phenylalanine-4-hydroxylase (EC 1.14.16.1)	1
Tryptophanase (EC 4.1.99.1)	1
Aromatic amino acid interconversions with aryl acids	1
Indolepyruvate ferredoxin oxidoreductase, alpha and beta subunits	1
Chorismate Synthesis	11
2-keto-3-deoxy-D-arabino-heptulosonate-7-phosphate synthase I alpha (EC 2.5.1.54)	1
3-dehydroquinate dehydratase II (EC 4.2.1.10)	1
3-dehydroquinate synthase (EC 4.2.3.4)	1
5-Enolpyruvylshikimate-3-phosphate synthase (EC 2.5.1.19)	1
Chorismate mutase II (EC 5.4.99.5)	1
Chorismate synthase (EC 4.2.3.5)	1
Cyclohexadienyl dehydrogenase (EC 1.3.1.12)(EC 1.3.1.43)	1
Prephenate dehydratase (EC 4.2.1.51)	1
Shikimate 5-dehydrogenase I alpha (EC 1.1.1.25)	1
Shikimate kinase I (EC 2.7.1.71)	1
Shikimate kinase III (EC 2.7.1.71)	1
Chorismate: Intermediate for synthesis of PAPA antibiotics, PABA, anthranilate, 3-hydroxyanthranilate and more.	6
Anthranilate synthase, amidotransferase component (EC 4.1.3.27)	1
Anthranilate synthase, aminase component (EC 4.1.3.27)	1
Isochorismate synthase (EC 5.4.4.2)	1
Isochorismate synthase (EC 5.4.4.2) of siderophore biosynthesis	1
Para-aminobenzoate synthase, amidotransferase component (EC 2.6.1.85)	1
Para-aminobenzoate synthase, aminase component (EC 2.6.1.85)	1
Cinnamic Acid Degradation	3
2-keto-4-pentenoate hydratase (EC 4.2.1.-)	1
4-hydroxybenzoate transporter	1
Probable 3-phenylpropionic acid transporter	1
Common Pathway For Synthesis of Aromatic Compounds (DAHP synthase to chorismate)	8
2-keto-3-deoxy-D-arabino-heptulosonate-7-phosphate synthase I alpha (EC 2.5.1.54)	1
3-dehydroquinate dehydratase II (EC 4.2.1.10)	1
3-dehydroquinate synthase (EC 4.2.3.4)	1
5-Enolpyruvylshikimate-3-phosphate synthase (EC 2.5.1.19)	1
Chorismate synthase (EC 4.2.3.5)	1
Shikimate 5-dehydrogenase I alpha (EC 1.1.1.25)	1
Shikimate kinase I (EC 2.7.1.71)	1
Shikimate kinase III (EC 2.7.1.71)	1
Phenylalanine and Tyrosine Branches from Chorismate	5

Biosynthetic Aromatic amino acid aminotransferase alpha (EC 2.6.1.57)	1
Chorismate mutase II (EC 5.4.99.5)	1
Cyclohexadienyl dehydratase (EC 4.2.1.51)(EC 4.2.1.91)	1
Cyclohexadienyl dehydrogenase (EC 1.3.1.12)(EC 1.3.1.43)	1
Prephenate dehydratase (EC 4.2.1.51)	1
Tryptophan catabolism	3
2-keto-4-pentenoate hydratase (EC 4.2.1.-)	1
Kynureninase (EC 3.7.1.3)	1
Kynurenine formamidase, bacterial (EC 3.5.1.9)	1
Tryptophan synthesis	8
Anthranilate phosphoribosyltransferase (EC 2.4.2.18)	1
Anthranilate synthase, amidotransferase component (EC 4.1.3.27)	1
Anthranilate synthase, aminase component (EC 4.1.3.27)	1
Indole-3-glycerol phosphate synthase (EC 4.1.1.48)	1
Para-aminobenzoate synthase, amidotransferase component (EC 2.6.1.85)	1
Phosphoribosylanthranilate isomerase (EC 5.3.1.24)	1
Tryptophan synthase alpha chain (EC 4.2.1.20)	1
Tryptophan synthase beta chain (EC 4.2.1.20)	1
**Branched-chain amino acids**	**85**
Branched chain amino acid degradation regulons	17
3-hydroxyisobutyrate dehydrogenase (EC 1.1.1.31)	1
3-hydroxyisobutyryl-CoA hydrolase (EC 3.1.2.4)	1
Acetoacetyl-CoA synthetase (EC 6.2.1.16)	1
Branched-chain alpha-keto acid dehydrogenase, E1 component, alpha subunit (EC 1.2.4.4)	1
Branched-chain alpha-keto acid dehydrogenase, E1 component, beta subunit (EC 1.2.4.4)	1
Dihydrolipoamide acyltransferase component of branched-chain alpha-keto acid dehydrogenase complex (EC 2.3.1.168)	1
Electron transfer flavoprotein, alpha subunit	1
Electron transfer flavoprotein-ubiquinone oxidoreductase (EC 1.5.5.1)	1
Hydroxymethylglutaryl-CoA lyase (EC 4.1.3.4)	1
Isovaleryl-CoA dehydrogenase (EC 1.3.99.10)	1
Methylcrotonyl-CoA carboxylase biotin-containing subunit (EC 6.4.1.4)	1
Methylcrotonyl-CoA carboxylase carboxyl transferase subunit (EC 6.4.1.4)	1
Methylglutaconyl-CoA hydratase (EC 4.2.1.18)	1
Methylmalonate-semialdehyde dehydrogenase (EC 1.2.1.27)	1
Predicted transcriptional regulator LiuR of leucine degradation pathway, MerR family	1
Succinyl-CoA:3-ketoacid-coenzyme A transferase subunit B (EC 2.8.3.5)	1
Transcriptional regulator BkdR of isoleucine and valine catabolism operon	1

Branched-Chain Amino Acid Biosynthesis	11
(R)-citramalate synthase (EC 2.3.1.182)	1
2-isopropylmalate synthase (EC 2.3.3.13)	1
3-isopropylmalate dehydrogenase (EC 1.1.1.85)	1
Acetolactate synthase large subunit (EC 2.2.1.6)	1
Acetolactate synthase small subunit (EC 2.2.1.6)	1
Dihydroxy-acid dehydratase (EC 4.2.1.9)	1
Leucine-responsive regulatory protein, regulator for leucine (or lrp) regulon and high-affinity branched-chain amino acid transport system	1
Threonine dehydratase (EC 4.3.1.19)	1
Threonine dehydratase biosynthetic (EC 4.3.1.19)	1
Threonine dehydratase, catabolic (EC 4.3.1.19)	1
Valine--pyruvate aminotransferase (EC 2.6.1.66)	1
HMG CoA Synthesis	7
Acetoacetyl-CoA synthetase (EC 6.2.1.16)	1
Hydroxymethylglutaryl-CoA lyase (EC 4.1.3.4)	1
Isovaleryl-CoA dehydrogenase (EC 1.3.99.10)	1
Methylcrotonyl-CoA carboxylase biotin-containing subunit (EC 6.4.1.4)	1
Methylcrotonyl-CoA carboxylase carboxyl transferase subunit (EC 6.4.1.4)	1
Methylglutaconyl-CoA hydratase (EC 4.2.1.18)	1
Predicted transcriptional regulator LiuR of leucine degradation pathway, MerR family	1
HMG-CoA	12
Acetoacetyl-CoA synthetase (EC 6.2.1.16)	1
Branched-chain alpha-keto acid dehydrogenase, E1 component, alpha subunit (EC 1.2.4.4)	1
Branched-chain alpha-keto acid dehydrogenase, E1 component, beta subunit (EC 1.2.4.4)	1
Dihydrolipoamide acyltransferase component of branched-chain alpha-keto acid dehydrogenase complex (EC 2.3.1.168)	1
Dihydrolipoamide dehydrogenase of branched-chain alpha-keto acid dehydrogenase (EC 1.8.1.4)	1
Hydroxymethylglutaryl-CoA lyase (EC 4.1.3.4)	1
Hydroxymethylglutaryl-CoA synthase (EC 2.3.3.10)	1
Isovaleryl-CoA dehydrogenase (EC 1.3.99.10)	1
Methylcrotonyl-CoA carboxylase biotin-containing subunit (EC 6.4.1.4)	1
Methylcrotonyl-CoA carboxylase carboxyl transferase subunit (EC 6.4.1.4)	1
Methylglutaconyl-CoA hydratase (EC 4.2.1.18)	1
Succinyl-CoA:3-ketoacid-coenzyme A transferase subunit B (EC 2.8.3.5)	1
Isoleucine degradation	9
3-hydroxyacyl-CoA dehydrogenase (EC 1.1.1.35)	1
Acyl-CoA dehydrogenase, short-chain specific (EC 1.3.99.2)	1
Branched-chain alpha-keto acid dehydrogenase, E1 component, alpha subunit (EC 1.2.4.4)	1

Branched-chain alpha-keto acid dehydrogenase, E1 component, beta subunit (EC 1.2.4.4)	1
Dihydrolipoamide acyltransferase component of branched-chain alpha-keto acid dehydrogenase complex (EC 2.3.1.168)	1
Dihydrolipoamide dehydrogenase of branched-chain alpha-keto acid dehydrogenase (EC 1.8.1.4)	1
Enoyl-CoA hydratase (EC 4.2.1.17)	1
Probable acyl-CoA dehydrogenase (EC 1.3.99.3)	1
Transcriptional regulator BkdR of isoleucine and valine catabolism operon	1
Ketoisovalerate oxidoreductase	3
Acetyl-coenzyme A synthetase (EC 6.2.1.1)	1
Hydroxymethylglutaryl-CoA synthase (EC 2.3.3.10)	1
Pyruvate:ferredoxin oxidoreductase, gamma subunit (EC 1.2.7.1)	1
Leucine Biosynthesis	3
2-isopropylmalate synthase (EC 2.3.3.13)	1
3-isopropylmalate dehydrogenase (EC 1.1.1.85)	1
Probable transcriptional activator for leuABCD operon	1
Leucine Degradation and HMG-CoA Metabolism	12
Acetoacetyl-CoA synthetase (EC 6.2.1.16)	1
Branched-chain alpha-keto acid dehydrogenase, E1 component, alpha subunit (EC 1.2.4.4)	1
Branched-chain alpha-keto acid dehydrogenase, E1 component, beta subunit (EC 1.2.4.4)	1
Dihydrolipoamide acyltransferase component of branched-chain alpha-keto acid dehydrogenase complex (EC 2.3.1.168)	1
Dihydrolipoamide dehydrogenase of branched-chain alpha-keto acid dehydrogenase (EC 1.8.1.4)	1
Hydroxymethylglutaryl-CoA lyase (EC 4.1.3.4)	1
Hydroxymethylglutaryl-CoA synthase (EC 2.3.3.10)	1
Isovaleryl-CoA dehydrogenase (EC 1.3.99.10)	1
Methylcrotonyl-CoA carboxylase biotin-containing subunit (EC 6.4.1.4)	1
Methylcrotonyl-CoA carboxylase carboxyl transferase subunit (EC 6.4.1.4)	1
Methylglutaconyl-CoA hydratase (EC 4.2.1.18)	1
Succinyl-CoA:3-ketoacid-coenzyme A transferase subunit B (EC 2.8.3.5)	1
Valine degradation	11
3-hydroxyacyl-CoA dehydrogenase (EC 1.1.1.35)	1
3-hydroxyisobutyrate dehydrogenase (EC 1.1.1.31)	1
3-hydroxyisobutyryl-CoA hydrolase (EC 3.1.2.4)	1
Branched-chain alpha-keto acid dehydrogenase, E1 component, alpha subunit (EC 1.2.4.4)	1
Branched-chain alpha-keto acid dehydrogenase, E1 component, beta subunit (EC 1.2.4.4)	1
Dihydrolipoamide acyltransferase component of branched-chain alpha-keto acid dehydrogenase complex (EC 2.3.1.168)	1
Dihydrolipoamide dehydrogenase of branched-chain alpha-keto acid dehydrogenase (EC 1.8.1.4)	1
Enoyl-CoA hydratase (EC 4.2.1.17)	1
Methylmalonate-semialdehyde dehydrogenase (EC 1.2.1.27)	1

Probable acyl-CoA dehydrogenase (EC 1.3.99.3)	1
Transcriptional regulator BkdR of isoleucine and valine catabolism operon	1
**Creatine and Creatinine Degradation**	**2**
Creatine and Creatinine Degradation	2
Cytosine deaminase (EC 3.5.4.1)	1
N-methylhydantoinase A (EC 3.5.2.14)	1
**Glutamine, glutamate, aspartate, asparagine; ammonia assimilation**	**22**
Glutamate and Aspartate uptake in Bacteria	2
Asparagine synthetase [glutamine-hydrolyzing] (EC 6.3.5.4)	1
Glutamate Aspartate transport system permease protein GltK (TC 3.A.1.3.4)	1
Glutamate dehydrogenases	3
NADP-specific glutamate dehydrogenase (EC 1.4.1.4)	1
NAD-specific glutamate dehydrogenase (EC 1.4.1.2)	1
NAD-specific glutamate dehydrogenase (EC 1.4.1.2), large form	1
Glutamine synthetases	2
Glutamine synthetase type III, GlnN (EC 6.3.1.2)	1
Glutamine synthetase, clostridia type (EC 6.3.1.2)	1
Glutamine, Glutamate, Aspartate and Asparagine Biosynthesis	14
Asparagine synthetase [glutamine-hydrolyzing] (EC 6.3.5.4)	1
Asparagine synthetase [glutamine-hydrolyzing] (EC 6.3.5.4) AsnH	1
Aspartate aminotransferase (EC 2.6.1.1)	1
Ferredoxin-dependent glutamate synthase (EC 1.4.7.1)	1
Glutamate synthase [NADPH] large chain (EC 1.4.1.13)	1
Glutaminase (EC 3.5.1.2)	1
glutamine synthetase family protein	1
Glutamine synthetase type III, GlnN (EC 6.3.1.2)	1
Glutamine synthetase, clostridia type (EC 6.3.1.2)	1
L-asparaginase (EC 3.5.1.1)	1
Leucine-responsive regulatory protein, regulator for leucine (or lrp) regulon and high-affinity branched-chain amino acid transport system	1
NADP-specific glutamate dehydrogenase (EC 1.4.1.4)	1
NAD-specific glutamate dehydrogenase (EC 1.4.1.2)	1
NAD-specific glutamate dehydrogenase (EC 1.4.1.2), large form	1
Poly-gamma-glutamate biosynthesis	1
Gamma-glutamyltranspeptidase (EC 2.3.2.2)	1
**Histidine Metabolism**	**13**
Histidine Biosynthesis	9
ATP phosphoribosyltransferase (EC 2.4.2.17)	1

ATP phosphoribosyltransferase catalytic subunit (EC 2.4.2.17)	1
ATP phosphoribosyltransferase regulatory subunit (EC 2.4.2.17)	1
Histidinol dehydrogenase (EC 1.1.1.23)	1
Histidinol-phosphatase [alternative form] (EC 3.1.3.15)	1
Histidinol-phosphate aminotransferase (EC 2.6.1.9)	1
Imidazole glycerol phosphate synthase amidotransferase subunit (EC 2.4.2.-)	1
Imidazole glycerol phosphate synthase cyclase subunit (EC 4.1.3.-)	1
Phosphoribosylformimino-5-aminoimidazole carboxamide ribotide isomerase (EC 5.3.1.16)	1
Histidine Degradation	4
Formiminoglutamase (EC 3.5.3.8)	1
Formiminoglutamic iminohydrolase (EC 3.5.3.13)	1
Histidine ammonia-lyase (EC 4.3.1.3)	1
N-formylglutamate deformylase (EC 3.5.1.68)	1
**Lysine, threonine, methionine, and cysteine**	**72**
Cysteine Biosynthesis	16
Cys regulon transcriptional activator CysB	1
Cystathionine beta-synthase (EC 4.2.1.22)	1
Cysteine synthase (EC 2.5.1.47)	1
Cysteine synthase B (EC 2.5.1.47)	1
Phosphoadenylyl-sulfate reductase [thioredoxin] (EC 1.8.4.8)	1
Serine acetyltransferase (EC 2.3.1.30)	1
Sulfate adenylyltransferase subunit 1 (EC 2.7.7.4)	1
Sulfate adenylyltransferase subunit 2 (EC 2.7.7.4)	1
Sulfate and thiosulfate binding protein CysP	1
Sulfate and thiosulfate import ATP-binding protein CysA (EC 3.6.3.25)	1
Sulfate permease	1
Sulfate transport system permease protein CysT	1
Sulfate transport system permease protein CysW	1
Sulfate transporter, CysZ-type	1
Sulfite reductase [NADPH] flavoprotein alpha-component (EC 1.8.1.2)	1
Sulfite reductase [NADPH] hemoprotein beta-component (EC 1.8.1.2)	1
Lysine biosynthesis AAA pathway 2	1
Lysine biosynthesis protein LysX	1
Lysine Biosynthesis DAP Pathway	7
2,3,4,5-tetrahydropyridine-2,6-dicarboxylate N-acetyltransferase (EC 2.3.1.89)	1
Aspartate-semialdehyde dehydrogenase (EC 1.2.1.11)	1
Aspartokinase (EC 2.7.2.4)	1

Diaminopimelate epimerase (EC 5.1.1.7)	1
Dihydrodipicolinate synthase (EC 4.2.1.52)	1
N-acetyl-L,L-diaminopimelate deacetylase (EC 3.5.1.47)	1
N-succinyl-L,L-diaminopimelate aminotransferase alternative (EC 2.6.1.17)	1
Lysine degradation	5
5-aminopentanamidase (EC 3.5.1.30)	1
L-lysine permease	1
Lysine decarboxylase, inducible (EC 4.1.1.18)	1
Lysine/cadaverine antiporter membrane protein CadB	1
Transcriptional activator of cad operon	1
Lysine fermentation	1
Electron transfer flavoprotein, alpha subunit	1
Methionine Biosynthesis	16
5,10-methylenetetrahydrofolate reductase (EC 1.5.1.20)	1
5-methyltetrahydropteroyltriglutamate--homocysteine methyltransferase (EC 2.1.1.14)	1
Adenosylhomocysteinase (EC 3.3.1.1)	1
Cystathionine beta-synthase (EC 4.2.1.22)	1
Cystathionine gamma-synthase (EC 2.5.1.48)	1
Cysteine synthase (EC 2.5.1.47)	1
Duplicated ATPase component MtsB of energizing module of methionine-regulated ECF transporter	1
Homoserine dehydrogenase (EC 1.1.1.3)	1
Homoserine kinase (EC 2.7.1.39)	1
Homoserine O-acetyltransferase (EC 2.3.1.31)	1
Homoserine O-succinyltransferase (EC 2.3.1.46)	1
Methionine ABC transporter permease protein	1
Methionine ABC transporter substrate-binding protein	1
Methionine repressor MetJ	1
S-adenosylmethionine synthetase (EC 2.5.1.6)	1
Serine acetyltransferase (EC 2.3.1.30)	1
Methionine Degradation	8
2-Oxobutyrate oxidase, putative	1
Adenosylhomocysteinase (EC 3.3.1.1)	1
Cystathionine beta-synthase (EC 4.2.1.22)	1
Methionine ABC transporter permease protein	1
Methionine ABC transporter substrate-binding protein	1
Methionine gamma-lyase (EC 4.4.1.11)	1
Pyruvate dehydrogenase E1 component (EC 1.2.4.1)	1

S-adenosylmethionine synthetase (EC 2.5.1.6)	1
Methionine Salvage	2
2-hydroxy-3-keto-5-methylthiopentenyl-1-phosphate phosphatase	1
5-methylthioribose kinase (EC 2.7.1.100)	1
Threonine anaerobic catabolism gene cluster	4
L-threonine transporter, anaerobically inducible	1
Phosphate acetyltransferase (EC 2.3.1.8)	1
Serine transporter	1
Threonine dehydratase, catabolic (EC 4.3.1.19)	1
Threonine and Homoserine Biosynthesis	6
Aspartate aminotransferase (EC 2.6.1.1)	1
Aspartate-semialdehyde dehydrogenase (EC 1.2.1.11)	1
Aspartokinase (EC 2.7.2.4)	1
Homoserine dehydrogenase (EC 1.1.1.3)	1
Homoserine kinase (EC 2.7.1.39)	1
Threonine synthase (EC 4.2.3.1)	1
Threonine degradation	6
FIG003492: Threonine dehydrogenase and related Zn-dependent dehydrogenases	1
low-specificity D-threonine aldolase	1
Low-specificity L-threonine aldolase (EC 4.1.2.5)	1
L-threonine transporter, anaerobically inducible	1
Threonine dehydratase (EC 4.3.1.19)	1
Threonine dehydratase, catabolic (EC 4.3.1.19)	1
**Proline and 4-hydroxyproline**	**16**
A Hypothetical Protein Related to Proline Metabolism	2
Hypothetical protein YggS, proline synthase co-transcribed bacterial homolog PROSC	1
Pyrroline-5-carboxylate reductase (EC 1.5.1.2)	1
Proline Synthesis	4
Gamma-glutamyl phosphate reductase (EC 1.2.1.41)	1
Glutamate 5-kinase (EC 2.7.2.11)	1
NADP-specific glutamate dehydrogenase (EC 1.4.1.4)	1
Pyrroline-5-carboxylate reductase (EC 1.5.1.2)	1
Proline, 4-hydroxyproline uptake and utilization	10
1-pyrroline-4-hydroxy-2-carboxylate deaminase (EC 3.5.4.22)	1
4-hydroxyproline epimerase (EC 5.1.1.8)	1
D-amino-acid oxidase (EC 1.4.3.3)	1
Delta-1-pyrroline-5-carboxylate dehydrogenase (EC 1.5.1.12)	1

L-Proline/Glycine betaine transporter ProP	1
Microbial collagenase, secreted (EC 3.4.24.3)	1
Not a Proline racemase, nor 4-hydroxyproline epimerase [missing catalytic residues]	1
Proline dehydrogenase (EC 1.5.99.8) (Proline oxidase)	1
ProQ: influences osmotic activation of compatible solute ProP	1
Ureidoglycolate/malate/sulfolactate dehydrogenase family (EC 1.1.1.-)	1
**Arabinose Sensor and transport module**	**2**
**Arabinose**	**2**
An Arabinose Sensor	2
L-arabinose transport system permease protein (TC 3.A.1.2.2)	1
L-arabinose-binding periplasmic protein precursor AraF (TC 3.A.1.2.2)	1
**Carbohydrates**	**611**
**Aminosugars**	**22**
(GlcNAc)2 Catabolic Operon	7
(GlcNAc)2 ABC transporter, ATP-binding component 1	1
(GlcNAc)2 ABC transporter, ATP-binding component 2	1
(GlcNAc)2 ABC transporter, permease component 1	1
(GlcNAc)2 ABC transporter, permease component 2	1
Chitin catabolic cascade sensor histidine kinase ChiS	1
Glucosamine kinase GpsK (EC 2.7.1.8)	1
Glucosamine-link cellobiase (EC 3.2.1.21)	1
Chitin and N-acetylglucosamine utilization	10
Beta-hexosaminidase (EC 3.2.1.52)	1
Chitinase (EC 3.2.1.14)	1
Glucosamine-6-phosphate deaminase (EC 3.5.99.6)	1
N-Acetyl-D-glucosamine ABC transport system, permease protein 1	1
N-Acetyl-D-glucosamine ABC transport system, permease protein 2	1
N-Acetyl-D-glucosamine ABC transport system, sugar-binding protein	1
N-acetylglucosamine regulated methyl-accepting chemotaxis protein	1
N-acetylglucosamine-6P-responsive transcriptional repressor NagC, ROK family	1
Predicted transcriptional regulator of N-Acetylglucosamine utilization, GntR family	1
PTS system, N-acetylglucosamine-specific IIA component (EC 2.7.1.69)	1
N-Acetyl-Galactosamine and Galactosamine Utilization	5
Beta-hexosaminidase (EC 3.2.1.52)	1
Beta-phosphoglucomutase (EC 5.4.2.6)	1
PTS system, N-acetylgalactosamine-specific IIC component (EC 2.7.1.69)	1
Tagatose 1,6-bisphosphate aldolase (EC 4.1.2.40)	1

Transcriptional repressor of aga operon	1
**Carbon storage regulator**	**3**
Carbon storage regulator	3
Carbon storage regulator	1
Flagellar assembly factor FliW	1
Flagellar hook-associated protein FlgK	1
**Central carbohydrate metabolism**	**148**
Dehydrogenase complexes	13
2-oxoglutarate dehydrogenase E1 component (EC 1.2.4.2)	1
Acetoin dehydrogenase E1 component alpha-subunit (EC 1.2.4.-)	1
Acetoin dehydrogenase E1 component beta-subunit (EC 1.2.4.-)	1
Branched-chain alpha-keto acid dehydrogenase, E1 component, alpha subunit (EC 1.2.4.4)	1
Branched-chain alpha-keto acid dehydrogenase, E1 component, beta subunit (EC 1.2.4.4)	1
Dihydrolipoamide acetyltransferase component of pyruvate dehydrogenase complex (EC 2.3.1.12)	1
Dihydrolipoamide acyltransferase component of branched-chain alpha-keto acid dehydrogenase complex (EC 2.3.1.168)	1
Dihydrolipoamide dehydrogenase of 2-oxoglutarate dehydrogenase (EC 1.8.1.4)	1
Dihydrolipoamide dehydrogenase of branched-chain alpha-keto acid dehydrogenase (EC 1.8.1.4)	1
Enoyl-CoA hydratase [branched-chain amino acid degradation] (EC 4.2.1.17)	1
Leucine-, isoleucine-, valine-, threonine-, and alanine-binding protein	1
Pyruvate dehydrogenase E1 component (EC 1.2.4.1)	1
Pyruvate dehydrogenase E1 component beta subunit (EC 1.2.4.1)	1
Dihydroxyacetone kinases	3
Dihydroxyacetone kinase, ATP-dependent (EC 2.7.1.29)	1
Phosphoenolpyruvate-dihydroxyacetone phosphotransferase (EC 2.7.1.121), ADP-binding subunit DhaL	1
Putative dihydroxyacetone kinase (EC 2.7.1.29), ADP-binding subunit	1
Entner-Doudoroff Pathway	15
2,3-bisphosphoglycerate-independent phosphoglycerate mutase (EC 5.4.2.1)	1
2,3-bisphosphoglycerate-independent phosphoglycerate mutase, archaeal type (EC 5.4.2.1)	1
2-dehydro-3-deoxygluconate kinase (EC 2.7.1.45)	1
6-phosphogluconolactonase (EC 3.1.1.31)	1
6-phosphogluconolactonase (EC 3.1.1.31), eukaryotic type	1
D-glycerate 2-kinase (EC 2.7.1.-)	1
Enolase (EC 4.2.1.11)	1
Glucokinase (EC 2.7.1.2)	1
Gluconokinase (EC 2.7.1.12)	1
Glucose dehydrogenase, PQQ-dependent (EC 1.1.5.2)	1
Glucose-6-phosphate 1-dehydrogenase (EC 1.1.1.49)	1

NAD-dependent glyceraldehyde-3-phosphate dehydrogenase (EC 1.2.1.12)	1
Phosphoglycerate kinase (EC 2.7.2.3)	1
Phosphoglycerate mutase (EC 5.4.2.1)	1
Pyruvate kinase (EC 2.7.1.40)	1
Ethylmalonyl-CoA pathway of C2 assimilation	1
Methylsuccinyl-CoA dehydrogenase, predicted by (Erb et al, 2007)	1
Ethylmalonyl-CoA pathway of C2 assimilation, GJO	4
Acetoacetyl-CoA reductase (EC 1.1.1.36)	1
Isocitrate lyase (EC 4.1.3.1)	1
Malate synthase (EC 2.3.3.9)	1
Methylsuccinyl-CoA dehydrogenase, predicted by (Erb et al, 2007)	1
Glycolate, glyoxylate interconversions	4
D-Lactate dehydrogenase, cytochrome c-dependent (EC 1.1.2.4)	1
Glycolate dehydrogenase (EC 1.1.99.14), iron-sulfur subunit GlcF	1
Glycolate permease	1
Glycolate utilization operon transcriptional activator GlcC	1
Glycolysis and Gluconeogenesis	18
2,3-bisphosphoglycerate-independent phosphoglycerate mutase (EC 5.4.2.1)	1
Enolase (EC 4.2.1.11)	1
Fructose-1,6-bisphosphatase, Bacillus type (EC 3.1.3.11)	1
Fructose-1,6-bisphosphatase, GlpX type (EC 3.1.3.11)	1
Fructose-1,6-bisphosphatase, type I (EC 3.1.3.11)	1
Fructose-bisphosphate aldolase class I (EC 4.1.2.13)	1
Glucokinase (EC 2.7.1.2)	1
Glucose-6-phosphate isomerase (EC 5.3.1.9)	1
NAD-dependent glyceraldehyde-3-phosphate dehydrogenase (EC 1.2.1.12)	1
NADPH-dependent glyceraldehyde-3-phosphate dehydrogenase (EC 1.2.1.13)	1
Phosphoenolpyruvate synthase (EC 2.7.9.2)	1
Phosphoglycerate kinase (EC 2.7.2.3)	1
Phosphoglycerate mutase (EC 5.4.2.1)	1
Predicted Fructose-bisphosphate aldolase (EC 4.1.2.13) in Geobacter	1
Pyrophosphate--fructose 6-phosphate 1-phosphotransferase, alpha subunit (EC 2.7.1.90)	1
Pyruvate kinase (EC 2.7.1.40)	1
Pyruvate,phosphate dikinase (EC 2.7.9.1)	1
Triosephosphate isomerase (EC 5.3.1.1)	1
Glycolysis and Gluconeogenesis, including Archaeal enzymes	13
2,3-bisphosphoglycerate-independent phosphoglycerate mutase (EC 5.4.2.1)	1

2,3-bisphosphoglycerate-independent phosphoglycerate mutase, archaeal type (EC 5.4.2.1)	1
Enolase (EC 4.2.1.11)	1
Fructose-1,6-bisphosphatase, GlpX type (EC 3.1.3.11)	1
Fructose-1,6-bisphosphatase, type I (EC 3.1.3.11)	1
Glucose-6-phosphate isomerase (EC 5.3.1.9)	1
Phosphoenolpyruvate synthase (EC 2.7.9.2)	1
Phosphoglycerate kinase (EC 2.7.2.3)	1
Phosphoglycerate mutase (EC 5.4.2.1)	1
Pyrophosphate--fructose 6-phosphate 1-phosphotransferase, alpha subunit (EC 2.7.1.90)	1
Pyruvate kinase (EC 2.7.1.40)	1
Pyruvate,phosphate dikinase (EC 2.7.9.1)	1
Triosephosphate isomerase (EC 5.3.1.1)	1
Glyoxylate bypass	7
Aconitate hydratase (EC 4.2.1.3)	1
Aconitate hydratase 2 (EC 4.2.1.3)	1
Citrate synthase (si) (EC 2.3.3.1)	1
Isocitrate lyase (EC 4.1.3.1)	1
Malate dehydrogenase (EC 1.1.1.37)	1
Malate synthase (EC 2.3.3.9)	1
Malate synthase G (EC 2.3.3.9)	1
Methylglyoxal Metabolism	2
Aldehyde dehydrogenase B (EC 1.2.1.22)	1
Methylglyoxal synthase (EC 4.2.3.3)	1
Pentose phosphate pathway	9
6-phosphogluconate dehydrogenase, decarboxylating (EC 1.1.1.44)	1
6-phosphogluconolactonase (EC 3.1.1.31)	1
6-phosphogluconolactonase (EC 3.1.1.31), eukaryotic type	1
Glucose-6-phosphate 1-dehydrogenase (EC 1.1.1.49)	1
Ribose 5-phosphate isomerase A (EC 5.3.1.6)	1
Ribulose-phosphate 3-epimerase (EC 5.1.3.1)	1
Transaldolase (EC 2.2.1.2)	1
Transketolase (EC 2.2.1.1)	1
Transketolase, C-terminal section (EC 2.2.1.1)	1
Peripheral Glucose Catabolism Pathways	2
Glucokinase (EC 2.7.1.2)	1
Integral membrane sensor signal transduction histidine kinase (EC 2.7.13.3), glucose catabolism cluster	1
Pyruvate Alanine Serine Interconversions	11

Alanine dehydrogenase (EC 1.4.1.1)	1
Alanine racemase (EC 5.1.1.1)	1
D-alanine aminotransferase (EC 2.6.1.21)	1
D-amino acid dehydrogenase small subunit (EC 1.4.99.1)	1
D-serine/D-alanine/glycine transporter	1
L-serine dehydratase (EC 4.3.1.17)	1
L-serine dehydratase, alpha subunit (EC 4.3.1.17)	1
L-serine dehydratase, beta subunit (EC 4.3.1.17)	1
Omega-amino acid--pyruvate aminotransferase (EC 2.6.1.18)	1
Serine transporter	1
Valine--pyruvate aminotransferase (EC 2.6.1.66)	1
Pyruvate metabolism I: anaplerotic reactions, PEP	17
Aerobic C4-dicarboxylate transporter for fumarate, L-malate, D-malate, succunate	1
Malate Na(+) symporter	1
Malate permease	1
Malolactic enzyme (EC 1.-.-.-)	1
NAD-dependent malic enzyme (EC 1.1.1.38)	1
NADP-dependent malic enzyme (EC 1.1.1.40)	1
Oxaloacetate decarboxylase alpha chain (EC 4.1.1.3)	1
Oxaloacetate decarboxylase gamma chain (EC 4.1.1.3)	1
Phosphoenolpyruvate carboxykinase [ATP] (EC 4.1.1.49)	1
Phosphoenolpyruvate carboxykinase [GTP] (EC 4.1.1.32)	1
Phosphoenolpyruvate carboxylase (EC 4.1.1.31)	1
Phosphoenolpyruvate synthase (EC 2.7.9.2)	1
Positive regulator of Tartrate dehydrogenase/decarboxylase/D-malic enzyme	1
Pyruvate carboxyl transferase (EC 6.4.1.1)	1
Pyruvate kinase (EC 2.7.1.40)	1
Pyruvate,phosphate dikinase (EC 2.7.9.1)	1
Two-component sensor histidine kinase, malate (EC 2.7.3.-)	1
Pyruvate metabolism II: acetyl-CoA, acetogenesis from pyruvate	12
Acetate permease ActP (cation/acetate symporter)	1
Acetyl-CoA synthetase (ADP-forming) alpha and beta chains, putative	1
Acetyl-coenzyme A synthetase (EC 6.2.1.1)	1
Acylphosphate phosphohydrolase (EC 3.6.1.7), putative	1
Dihydrolipoamide acetyltransferase component of pyruvate dehydrogenase complex (EC 2.3.1.12)	1
Lactate 2-monooxygenase (EC 1.13.12.4)	1
NAD-independent protein deacetylase AcuC	1

Phosphate acetyltransferase (EC 2.3.1.8)	1
Protein acetyltransferase	1
Putative membrane protein, clustering with ActP	1
Pyruvate dehydrogenase E1 component (EC 1.2.4.1)	1
Pyruvate dehydrogenase E1 component beta subunit (EC 1.2.4.1)	1
Pyruvate:ferredoxin oxidoreductase	2
Pyruvate:ferredoxin oxidoreductase, beta subunit (EC 1.2.7.1)	1
Pyruvate:ferredoxin oxidoreductase, gamma subunit (EC 1.2.7.1)	1
TCA Cycle	15
2-oxoglutarate dehydrogenase E1 component (EC 1.2.4.2)	1
Aconitate hydratase (EC 4.2.1.3)	1
Aconitate hydratase 2 (EC 4.2.1.3)	1
Citrate synthase (si) (EC 2.3.3.1)	1
Dihydrolipoamide dehydrogenase of 2-oxoglutarate dehydrogenase (EC 1.8.1.4)	1
Fumarate hydratase class I, aerobic (EC 4.2.1.2)	1
Fumarate hydratase class II (EC 4.2.1.2)	1
hypothetical protein that often co-occurs with aconitase	1
Isocitrate dehydrogenase [NAD] (EC 1.1.1.41)	1
Malate dehydrogenase (EC 1.1.1.37)	1
Malate:quinone oxidoreductase (EC 1.1.5.4)	1
Succinate dehydrogenase flavoprotein subunit (EC 1.3.99.1)	1
Succinate dehydrogenase iron-sulfur protein (EC 1.3.99.1)	1
Succinyl-CoA ligase [ADP-forming] alpha chain (EC 6.2.1.5)	1
Succinyl-CoA ligase [ADP-forming] beta chain (EC 6.2.1.5)	1
**CO2 fixation**	**32**
Acetyl-CoA pathway of CO2 fixation	1
Phosphate acetyltransferase (EC 2.3.1.8)	1
Calvin-Benson cycle	12
Fructose-1,6-bisphosphatase, GlpX type (EC 3.1.3.11)	1
Fructose-1,6-bisphosphatase, type I (EC 3.1.3.11)	1
Fructose-bisphosphate aldolase class I (EC 4.1.2.13)	1
NAD-dependent glyceraldehyde-3-phosphate dehydrogenase (EC 1.2.1.12)	1
NADPH-dependent glyceraldehyde-3-phosphate dehydrogenase (EC 1.2.1.13)	1
Phosphoglycerate kinase (EC 2.7.2.3)	1
Phosphoribulokinase (EC 2.7.1.19) homolog, function unknown	1
Ribose 5-phosphate isomerase A (EC 5.3.1.6)	1
Ribulose-phosphate 3-epimerase (EC 5.1.3.1)	1

Transketolase (EC 2.2.1.1)	1
Transketolase, C-terminal section (EC 2.2.1.1)	1
Triosephosphate isomerase (EC 5.3.1.1)	1
Carboxysome	3
Carbonic anhydrase (EC 4.2.1.1)	1
High-affnity carbon uptake protein Hat/HatR	1
NADH dehydrogenase (EC 1.6.99.3)	1
CO2 uptake, carboxysome	5
Carbon dioxide concentrating mechanism protein CcmO	1
High-affnity carbon uptake protein Hat/HatR	1
NADH dehydrogenase subunit 4, Involved in CO2 fixation	1
NADH dehydrogenase subunit 5, Involved in CO2 fixation	1
Sensory subunit of low CO2-induced protein complex, putative	1
Photorespiration (oxidative C2 cycle)	11
Catalase (EC 1.11.1.6)	1
D-Lactate dehydrogenase, cytochrome c-dependent (EC 1.1.2.4)	1
Glycerate kinase (EC 2.7.1.31)	1
Glycine cleavage system H protein	1
Glycine dehydrogenase [decarboxylating] (glycine cleavage system P protein) (EC 1.4.4.2)	1
Glycine dehydrogenase [decarboxylating] (glycine cleavage system P1 protein) (EC 1.4.4.2)	1
Glycine dehydrogenase [decarboxylating] (glycine cleavage system P2 protein) (EC 1.4.4.2)	1
Glycolate dehydrogenase (EC 1.1.99.14), iron-sulfur subunit GlcF	1
Glycolate permease	1
Malate synthase (EC 2.3.3.9)	1
Malate synthase G (EC 2.3.3.9)	1
**Di- and oligosaccharides**	**74**
Beta-Glucoside Metabolism	7
6-phospho-beta-glucosidase (EC 3.2.1.86)	1
Beta-glucanase precursor (EC 3.2.1.73)	1
Cellobiose phosphotransferase system YdjC-like protein	1
Outer surface protein of unknown function, cellobiose operon	1
PTS system, cellobiose-specific IIA component (EC 2.7.1.69)	1
PTS system, cellobiose-specific IIB component (EC 2.7.1.69)	1
PTS system, cellobiose-specific IIC component (EC 2.7.1.69)	1
Fructooligosaccharides(FOS) and Raffinose Utilization	5
Alpha-galactosidase (EC 3.2.1.22)	1
Alpha-mannosidase (EC 3.2.1.2	1

Multiple sugar ABC transporter, membrane-spanning permease protein MsmF	1
Raffinose operon transcriptional regulatory protein RafR	1
Sucrose phosphorylase (EC 2.4.1.7)	1
Lactose and Galactose Uptake and Utilization	10
Aldose 1-epimerase (EC 5.1.3.3)	1
Alpha-galactosidase (EC 3.2.1.22)	1
Beta-galactosidase (EC 3.2.1.23)	1
Galactose operon repressor, GalR-LacI family of transcriptional regulators	1
Galactose/methyl galactoside ABC transport system, ATP-binding protein MglA (EC 3.6.3.17)	1
Galactose/methyl galactoside ABC transport system, D-galactose-binding periplasmic protein MglB (TC 3.A.1.2.3)	1
Galactose/methyl galactoside ABC transport system, permease protein MglC (TC 3.A.1.2.3)	1
Lactose and galactose permease, GPH translocator family	1
Tagatose 1,6-bisphosphate aldolase (EC 4.1.2.40)	1
UDP-glucose 4-epimerase (EC 5.1.3.2)	1
Lactose utilization	3
Beta-galactosidase (EC 3.2.1.23)	1
Galactoside O-acetyltransferase (EC 2.3.1.18)	1
Lactose and galactose permease, GPH translocator family	1
Maltose and Maltodextrin Utilization	26
ABC-type sugar transport system, periplasmic binding protein YcjN	1
Aldose 1-epimerase (EC 5.1.3.3)	1
Alpha-amylase (EC 3.2.1.1)	1
Alpha-glucosidase (EC 3.2.1.20)	1
Beta-phosphoglucomutase (EC 5.4.2.6)	1
Glucoamylase (EC 3.2.1.3)	1
Glycogen phosphorylase (EC 2.4.1.1)	1
Maltodextrin glucosidase (EC 3.2.1.20)	1
Maltodextrin phosphorylase (EC 2.4.1.1)	1
Malto-oligosyltrehalose synthase (EC 5.4.99.15)	1
Maltoporin (maltose/maltodextrin high-affinity receptor, phage lambda receptor protein)	1
Maltose O-acetyltransferase (EC 2.3.1.79)	1
Maltose operon periplasmic protein MalM	1
Maltose operon transcriptional repressor MalR, LacI family	1
Maltose/maltodextrin ABC transporter, permease protein MalF	1
Maltose/maltodextrin ABC transporter, permease protein MalG	1
Maltose/maltodextrin ABC transporter, substrate binding periplasmic protein MalE	1
Maltose/maltodextrin transport ATP-binding protein MalK (EC 3.6.3.19)	1

Mlc, transcriptional repressor of MalT (the transcriptional activator of maltose regulon) and manXYZ operon	1
Neopullulanase (EC 3.2.1.135)	1
Periplasmic alpha-amylase (EC 3.2.1.1)	1
Predicted maltose transporter MalT	1
Predicted maltose-specific TonB-dependent receptor	1
Pullulanase (EC 3.2.1.41)	1
Transcriptional activator of maltose regulon, MalT	1
Transcriptional regulator of maltose utilization, LacI family	1
Melibiose Utilization	2
Alpha-galactosidase (EC 3.2.1.22)	1
Melibiose carrier protein, Na+/melibiose symporter	1
Sucrose utilization	3
Fructokinase (EC 2.7.1.4)	1
Sucrose operon repressor ScrR, LacI family	1
Sucrose phosphorylase (EC 2.4.1.7)	1
Sucrose utilization Shewanella	2
Fructokinase (EC 2.7.1.4)	1
Sucrose phosphorylase (EC 2.4.1.7)	1
Trehalose Biosynthesis	8
1,4-alpha-glucan (glycogen) branching enzyme, GH-13-type (EC 2.4.1.18)	1
Alpha,alpha-trehalose-phosphate synthase [UDP-forming] (EC 2.4.1.15)	1
Alpha-amylase (EC 3.2.1.1)	1
Glucoamylase (EC 3.2.1.3)	1
Glycogen debranching enzyme (EC 3.2.1.-)	1
Malto-oligosyltrehalose synthase (EC 5.4.99.15)	1
Malto-oligosyltrehalose trehalohydrolase (EC 3.2.1.141)	1
Trehalose synthase (EC 5.4.99.16)	1
Trehalose Uptake and Utilization	8
Beta-phosphoglucomutase (EC 5.4.2.6)	1
Glucose/mannose:H+ symporter GlcP	1
Maltoporin (maltose/maltodextrin high-affinity receptor, phage lambda receptor protein)	1
PTS system, glucose-specific IIA component (EC 2.7.1.69)	1
Transcriptional regulator of trehalose utilization, LacI family	1
Trehalose operon transcriptional repressor	1
Trehalose-6-phosphate hydrolase (EC 3.2.1.93)	1
Trehalose-regulated TonB-dependent outer membrane receptor	1
**Fermentation**	**25**

Acetoin, butanediol metabolism	6
Acetoin dehydrogenase E1 component alpha-subunit (EC 1.2.4.-)	1
Acetoin dehydrogenase E1 component beta-subunit (EC 1.2.4.-)	1
Acetolactate synthase large subunit (EC 2.2.1.6)	1
Acetolactate synthase small subunit (EC 2.2.1.6)	1
Acetolactate synthase, catabolic (EC 2.2.1.6)	1
Transcriptional activator of acetoin dehydrogenase operon AcoR	1
Acetone Butanol Ethanol Synthesis	3
Alcohol dehydrogenase (EC 1.1.1.1)	1
Electron transfer flavoprotein, alpha subunit	1
NADH-dependent butanol dehydrogenase A (EC 1.1.1.-)	1
Acetyl-CoA fermentation to Butyrate	6
3-hydroxyacyl-CoA dehydrogenase (EC 1.1.1.35)	1
Acetoacetyl-CoA reductase (EC 1.1.1.36)	1
Electron transfer flavoprotein, alpha subunit	1
Electron transfer flavoprotein-ubiquinone oxidoreductase (EC 1.5.5.1)	1
Enoyl-CoA hydratase (EC 4.2.1.17)	1
Probable electron transfer flavoprotein-quinone oxidoreductase FixC (EC 1.5.5.-)	1
Butanol Biosynthesis	3
Enoyl-CoA hydratase (EC 4.2.1.17)	1
NADH-dependent butanol dehydrogenase A (EC 1.1.1.-)	1
Pyruvate formate-lyase (EC 2.3.1.54)	1
Fermentations: Lactate	1
Phosphate acetyltransferase (EC 2.3.1.8)	1
Fermentations: Mixed acid	6
Alcohol dehydrogenase (EC 1.1.1.1)	1
Formate efflux transporter (TC 2.A.44 family)	1
Phosphate acetyltransferase (EC 2.3.1.8)	1
Phosphoenolpyruvate carboxylase (EC 4.1.1.31)	1
Pyruvate formate-lyase (EC 2.3.1.54)	1
Sugar/maltose fermentation stimulation protein homolog	1
**Glycoside hydrolases**	**3**
Predicted carbohydrate hydrolases	3
COG2152 predicted glycoside hydrolase	1
Peptidoglycan N-acetylglucosamine deacetylase (EC 3.5.1.-)	1
Polysaccharide deacetylase	1
**Lacto-N-Biose I and Galacto-N-Biose Metabolic Pathway**	**1**

Lacto-N-Biose I and Galacto-N-Biose Metabolic Pathway	1
UDP-glucose 4-epimerase (EC 5.1.3.2)	1
**Monosaccharides**	**104**
2-Ketogluconate Utilization	2
Epimerase KguE	1
HTH-type transcriptional regulator PtxR	1
Deoxyribose and Deoxynucleoside Catabolism	10
Deoxyribose operon repressor, DeoR family	1
Deoxyribose-phosphate aldolase (EC 4.1.2.4)	1
Nucleoside permease NupC	1
Phosphopentomutase (EC 5.4.2.7)	1
Predicted nucleoside ABC transporter, substrate-binding component	1
Purine nucleoside phosphorylase (EC 2.4.2.1)	1
Putative deoxyribonuclease YjjV	1
Putative deoxyribose-specific ABC transporter, permease protein	1
Pyrimidine-nucleoside phosphorylase (EC 2.4.2.2)	1
Ribokinase (EC 2.7.1.15)	1
D-galactarate, D-glucarate and D-glycerate catabolism	7
5-dehydro-4-deoxyglucarate dehydratase (EC 4.2.1.41)	1
D-galactarate dehydratase (EC 4.2.1.42)	1
D-galactarate permease	1
D-glucarate permease	1
D-glycerate 2-kinase (EC 2.7.1.-)	1
Glycerate kinase (EC 2.7.1.31)	1
Sugar diacid utilization regulator SdaR	1
D-galactonate catabolism	3
2-dehydro-3-deoxygalactonokinase (EC 2.7.1.58)	1
2-dehydro-3-deoxyphosphogalactonate aldolase (EC 4.1.2.21)	1
D-galactonate transporter	1
D-Galacturonate and D-Glucuronate Utilization	11
2-dehydro-3-deoxygluconate kinase (EC 2.7.1.45)	1
5-dehydro-4-deoxyglucarate dehydratase (EC 4.2.1.41)	1
Alpha-glucosidase (EC 3.2.1.20)	1
Altronate hydrolase (EC 4.2.1.7)	1
D-galactarate dehydratase (EC 4.2.1.42)	1
D-mannonate oxidoreductase (EC 1.1.1.57)	1
Endo-1,4-beta-xylanase A precursor (EC 3.2.1.8)	1

Hexuronate utilization operon transcriptional repressor ExuR	1
Novel D-mannonate-D-gluconate epimerase	1
Polygalacturonase (EC 3.2.1.15)	1
Uronate isomerase (EC 5.3.1.12)	1
D-gluconate and ketogluconates metabolism	11
2-dehydro-3-deoxygluconate kinase (EC 2.7.1.45)	1
2-Keto-D-gluconate dehydrogenase (EC 1.1.99.4), membrane-bound, cytochrome c	1
5-keto-D-gluconate 5-reductase (EC 1.1.1.69)	1
Gluconate 2-dehydrogenase (EC 1.1.99.3), membrane-bound, gamma subunit	1
Gluconate permease	1
Gluconate permease, Bsu4004 homolog	1
Gluconate transporter family protein	1
Gluconate utilization system Gnt-I transcriptional repressor	1
Gluconokinase (EC 2.7.1.12)	1
Glucose dehydrogenase, PQQ-dependent (EC 1.1.5.2)	1
Low-affinity gluconate/H+ symporter GntU	1
D-ribose utilization	3
Predicted nucleoside ABC transporter, substrate-binding component	1
Ribokinase (EC 2.7.1.15)	1
Ribose 5-phosphate isomerase A (EC 5.3.1.6)	1
D-Sorbitol(D-Glucitol) and L-Sorbose Utilization	2
Putative transcriptional regulator of sorbose uptake and utilization genes	1
Sorbitol operon transcription regulator	1
D-Tagatose and Galactitol Utilization	1
Tagatose 1,6-bisphosphate aldolase (EC 4.1.2.40)	1
Fructose utilization	11
1-phosphofructokinase (EC 2.7.1.56)	1
Fructokinase (EC 2.7.1.4)	2
Fructose ABC transporter, permease component FrcC	1
Fructose ABC transporter, substrate-binding component FrcB	1
Phosphoenolpyruvate-protein phosphotransferase of PTS system (EC 2.7.3.9)	1
Phosphotransferase system, phosphocarrier protein HPr	1
PTS system, fructose-specific IIB component (EC 2.7.1.69)	1
Transaldolase (EC 2.2.1.2)	1
Transcriptional regulator FrcR for fructose utilization, ROK family	1
Transcriptional repressor of the fructose operon, DeoR family	1
Hexose Phosphate Uptake System	4

Hexose phosphate uptake regulatory protein UhpC	1
Homolog of fucose/glucose/galactose permeases	1
Sensor histidine protein kinase UhpB, glucose-6-phosphate specific (EC 2.7.13.3)	1
Transcriptional regulatory protein UhpA	1
L-Arabinose utilization	8
Alpha-L-arabinofuranosidase II precursor (EC 3.2.1.55)	1
Alpha-N-arabinofuranosidase (EC 3.2.1.55)	1
Alpha-N-arabinofuranosidase 2 (EC 3.2.1.55)	1
Arabinose operon protein AraL	1
Arabinose operon regulatory protein	1
L-arabinose transport system permease protein (TC 3.A.1.2.2)	1
L-arabinose-binding periplasmic protein precursor AraF (TC 3.A.1.2.2)	1
L-ribulose-5-phosphate 4-epimerase (EC 5.1.3.4)	1
L-ascorbate utilization (and related gene clusters)	5
3-keto-L-gulonate 6-phosphate decarboxylase	1
L-ribulose-5-phosphate 4-epimerase (EC 5.1.3.4)	1
L-xylulose 5-phosphate 3-epimerase (EC 5.1.3.-)	1
Probable L-ascorbate-6-phosphate lactonase UlaG (EC 3.1.1.-) (L-ascorbate utilization protein G)	1
Putative 2-keto-3-deoxygluconate kinase (EC 2.7.1.45)	1
L-fucose utilization	2
Alpha-L-fucosidase (EC 3.2.1.51)	1
Lactaldehyde reductase (EC 1.1.1.77)	1
L-fucose utilization temp	2
Alpha-L-fucosidase (EC 3.2.1.51)	1
L-fuco-beta-pyranose dehydrogenase	1
L-rhamnose utilization	6
Alfa-L-rhamnosidase (EC 3.2.1.40)	1
Lactaldehyde reductase (EC 1.1.1.77)	1
L-lactate dehydrogenase (EC 1.1.2.3)	1
L-rhamnose operon transcriptional activator RhaR	1
Predicted L-lactate dehydrogenase, Iron-sulfur cluster-binding subunit YkgF	1
Transcriptional regulator of rhamnose utilization, AraC family	1
Mannose Metabolism	8
Alpha-1,2-mannosidase	1
Beta-mannosidase (EC 3.2.1.25)	1
Endo-1,4-beta-mannosidase	1
Mannoside ABC transport system, sugar-binding protein	1

Phosphomannomutase (EC 5.4.2.8)	1
PTS system, mannose-specific IIA component (EC 2.7.1.69)	1
PTS system, mannose-specific IIC component (EC 2.7.1.69)	1
Transcriptional regulator of mannoside utilization, LacI family	1
Unknown pentose utilization	3
Ribokinase (EC 2.7.1.15)	1
Transketolase (EC 2.2.1.1)	1
Transketolase, C-terminal section (EC 2.2.1.1)	1
Xylose utilization	5
Alpha-xylosidase (EC 3.2.1.-)	1
Beta-xylosidase (EC 3.2.1.37)	1
Endo-1,4-beta-xylanase A precursor (EC 3.2.1.8)	1
Xylose ABC transporter, substrate-binding component	1
Xylose-responsive transcription regulator, ROK family	1
**One-carbon Metabolism**	**34**
Formaldehyde assimilation: Ribulose monophosphate pathway	1
6-phospho-3-hexuloisomerase	1
Methanogenesis	4
CoB--CoM heterodisulfide reductase subunit A (EC 1.8.98.1)	1
Formylmethanofuran--tetrahydromethanopterin N-formyltransferase (EC 2.3.1.101)	1
N(5),N(10)-methenyltetrahydromethanopterin cyclohydrolase (EC 3.5.4.27)	1
N5-methyltetrahydromethanopterin:coenzyme M methyltransferase subunit C (EC 2.1.1.86)	1
One-carbon metabolism by tetrahydropterines	5
5,10-methylenetetrahydrofolate reductase (EC 1.5.1.20)	1
5-formyltetrahydrofolate cyclo-ligase (EC 6.3.3.2)	1
Formate--tetrahydrofolate ligase (EC 6.3.4.3)	1
Formyltetrahydrofolate deformylase (EC 3.5.1.10)	1
N(5),N(10)-methenyltetrahydromethanopterin cyclohydrolase (EC 3.5.4.27)	1
Serine-glyoxylate cycle	24
5,10-methylenetetrahydrofolate reductase (EC 1.5.1.20)	1
5-formyltetrahydrofolate cyclo-ligase (EC 6.3.3.2)	1
Acetoacetyl-CoA reductase (EC 1.1.1.36)	1
Aconitate hydratase (EC 4.2.1.3)	1
Citrate synthase (si) (EC 2.3.3.1)	1
cytosolic long-chain acyl-CoA thioester hydrolase family protein	1
Enolase (EC 4.2.1.11)	1
Formate--tetrahydrofolate ligase (EC 6.3.4.3)	1

Fumarate hydratase class I, aerobic (EC 4.2.1.2)	1
Glycerate kinase (EC 2.7.1.31)	1
Isocitrate lyase (EC 4.1.3.1)	1
low-specificity D-threonine aldolase	1
Malate dehydrogenase (EC 1.1.1.37)	1
Methylcrotonyl-CoA carboxylase biotin-containing subunit (EC 6.4.1.4)	1
Methylcrotonyl-CoA carboxylase carboxyl transferase subunit (EC 6.4.1.4)	1
Methylmalonyl-CoA mutase (EC 5.4.99.2)	1
N(5),N(10)-methenyltetrahydromethanopterin cyclohydrolase (EC 3.5.4.27)	1
Phosphoenolpyruvate carboxykinase [ATP] (EC 4.1.1.49)	1
Propionyl-CoA carboxylase beta chain (EC 6.4.1.3)	1
Succinate dehydrogenase flavoprotein subunit (EC 1.3.99.1)	1
Succinate dehydrogenase iron-sulfur protein (EC 1.3.99.1)	1
Succinyl-CoA ligase [ADP-forming] alpha chain (EC 6.2.1.5)	1
Succinyl-CoA ligase [ADP-forming] beta chain (EC 6.2.1.5)	1
Succinyl-CoA:3-ketoacid-coenzyme A transferase subunit B (EC 2.8.3.5)	1
**Organic acids**	**41**
Alpha-acetolactate operon	1
Acetolactate synthase, catabolic (EC 2.2.1.6)	1
CitAB	2
Sensor kinase CitA, DpiB (EC 2.7.3.-)	1
Transcriptional regulatory protein CitB, DpiA	1
Citrate Metabolism, Transport, and Regulation	10
[Citrate [pro-3S]-lyase] ligase (EC 6.2.1.22)	1
2-(5''-triphosphoribosyl)-3'-dephosphocoenzyme-A synthase (EC 2.7.8.25)	1
Anaerobic C4-dicarboxylate transporter DcuC	1
Apo-citrate lyase phosphoribosyl-dephospho-CoA transferase (EC 2.7.7.61)	1
Citrate lyase alpha chain (EC 4.1.3.6)	1
Citrate lyase beta chain (EC 4.1.3.6)	1
Citrate Succinate antiporter (TC 2.A.47.3.2)	1
Na(+)Citrate OH(-) antiporter	1
Sensor kinase CitA, DpiB (EC 2.7.3.-)	1
Transcriptional regulatory protein CitB, DpiA	1
Glycerate metabolism	3
D-glycerate 2-kinase (EC 2.7.1.-)	1
Glycerate kinase (EC 2.7.1.31)	1
Pyruvate kinase (EC 2.7.1.40)	1

Isobutyryl-CoA to Propionyl-CoA Module	3
3-hydroxyisobutyrate dehydrogenase (EC 1.1.1.31)	1
3-hydroxyisobutyryl-CoA hydrolase (EC 3.1.2.4)	1
Methylmalonate-semialdehyde dehydrogenase (EC 1.2.1.27)	1
Lactate utilization	6
D-Lactate dehydrogenase (EC 1.1.2.5)	1
Lactate-responsive regulator LldR in Enterobacteria, GntR family	1
L-lactate dehydrogenase (EC 1.1.2.3)	1
L-lactate permease	1
Predicted Lactate-responsive regulator, LysR family	1
Predicted L-lactate dehydrogenase, Iron-sulfur cluster-binding subunit YkgF	1
Malonate decarboxylase	2
Malonate decarboxylase beta subunit	1
Malonate utilization transcriptional regulator	1
Methylcitrate cycle	4
2-methylcitrate dehydratase (EC 4.2.1.79)	1
2-methylcitrate dehydratase FeS dependent (EC 4.2.1.79)	1
2-methylcitrate synthase (EC 2.3.3.5)	1
Methylisocitrate lyase (EC 4.1.3.30)	1
Propionate-CoA to Succinate Module	6
2-methylcitrate dehydratase (EC 4.2.1.79)	1
2-methylcitrate dehydratase FeS dependent (EC 4.2.1.79)	1
2-methylcitrate synthase (EC 2.3.3.5)	1
Aconitate hydratase (EC 4.2.1.3)	1
Aconitate hydratase 2 (EC 4.2.1.3)	1
Methylisocitrate lyase (EC 4.1.3.30)	1
Propionyl-CoA to Succinyl-CoA Module	3
Methylmalonyl-CoA mutase (EC 5.4.99.2)	1
Na+/H+-dicarboxylate symporters	1
Propionyl-CoA carboxylase biotin-containing subunit (EC 6.4.1.3)	1
Tricarballylate Utilization	1
TcuB: works with TcuA to oxidize tricarballylate to cis-aconitate	1
**Polysaccharides**	**11**
Alpha-Amylase locus in Streptocococcus	2
Maltose/maltodextrin ABC transporter, substrate binding periplasmic protein MalE	1
putative esterase	1
Cellulosome	2

Alpha-glucosidase SusB (EC 3.2.1.20)	1
SusC, outer membrane protein involved in starch binding	1
Glycogen metabolism	6
1,4-alpha-glucan (glycogen) branching enzyme, GH-13-type (EC 2.4.1.18)	1
Glucose-1-phosphate adenylyltransferase (EC 2.7.7.27)	1
Glycogen branching enzyme, GH-57-type, archaeal (EC 2.4.1.18)	1
Glycogen debranching enzyme (EC 3.2.1.-)	1
Glycogen phosphorylase (EC 2.4.1.1)	1
Maltodextrin phosphorylase (EC 2.4.1.1)	1
Unknown carbohydrate utilization containing Fructose-bisphosphate aldolase	1
Putative carbohydrate PTS system, IIC component (EC 2.7.1.69)	1
**Sugar alcohols**	**58**
Di-Inositol-Phosphate biosynthesis	1
Inositol-1-monophosphatase (EC 3.1.3.25)	1
Erythritol utilization	1
Possible D-erythrulose 4-phosphate dehydrogenase EryC (EC 1.1.1.-)	1
Ethanolamine utilization	4
Ethanolamine permease	1
Ethanolamine utilization protein EutA	1
Ethanolamine utilization protein EutQ	1
Phosphate acetyltransferase (EC 2.3.1.8)	1
Glycerol and Glycerol-3-phosphate Uptake and Utilization	13
Aerobic glycerol-3-phosphate dehydrogenase (EC 1.1.5.3)	1
Anaerobic glycerol-3-phosphate dehydrogenase subunit A (EC 1.1.5.3)	1
Anaerobic glycerol-3-phosphate dehydrogenase subunit B (EC 1.1.5.3)	1
GlpG protein (membrane protein of glp regulon)	1
Glycerol kinase (EC 2.7.1.30)	1
Glycerol uptake facilitator protein	1
Glycerol-3-phosphate ABC transporter, ATP-binding protein UgpC (TC 3.A.1.1.3)	1
Glycerol-3-phosphate ABC transporter, periplasmic glycerol-3-phosphate-binding protein (TC 3.A.1.1.3)	1
Glycerol-3-phosphate ABC transporter, permease protein UgpA (TC 3.A.1.1.3)	1
Glycerol-3-phosphate dehydrogenase (EC 1.1.5.3)	1
Glycerophosphoryl diester phosphodiesterase (EC 3.1.4.46)	1
Glycerophosphoryl diester phosphodiesterase, periplasmic (EC 3.1.4.46)	1
NADH peroxidase (EC 1.11.1.1)	1
Glycerol fermenation to 1,3-propanediol	2
Cob(I)alamin adenosyltransferase PduO (EC 2.5.1.17)	1

Glycerol uptake facilitator protein	1
Inositol catabolism	8
5-deoxy-glucuronate isomerase (EC 5.3.1.-)	1
5-keto-2-deoxy-D-gluconate-6 phosphate aldolase (EC 4.1.2.29)	1
Epi-inositol hydrolase (EC 3.7.1.-)	1
Inositol transport system ATP-binding protein	1
Major myo-inositol transporter IolT	1
Myo-inositol 2-dehydrogenase (EC 1.1.1.18)	1
Myo-inositol 2-dehydrogenase 1 (EC 1.1.1.18)	1
Transcriptional repressor of the myo-inositol catabolic operon DeoR family	1
Inositol utilization	10
5-deoxy-glucuronate isomerase (EC 5.3.1.-)	1
5-keto-2-deoxy-D-gluconate-6 phosphate aldolase (EC 4.1.2.29)	1
Epi-inositol hydrolase (EC 3.7.1.-)	1
Inositol transport system ATP-binding protein	1
Inositol-1-monophosphatase (EC 3.1.3.25)	1
Major myo-inositol transporter IolT	1
Methylmalonate-semialdehyde dehydrogenase (EC 1.2.1.27)	1
Myo-inositol 2-dehydrogenase (EC 1.1.1.18)	1
Myo-inositol 2-dehydrogenase 1 (EC 1.1.1.18)	1
Transcriptional repressor of the myo-inositol catabolic operon DeoR family	1
Mannitol Utilization	5
Fructokinase (EC 2.7.1.4)	1
Mannitol operon activator, BglG family	1
Mannitol operon repressor	1
Phosphoenolpyruvate-protein phosphotransferase of PTS system (EC 2.7.3.9)	1
Transcriptional regulator of mannitol utilization, DeoR family protein	1
Propanediol utilization	4
Cob(I)alamin adenosyltransferase PduO (EC 2.5.1.17)	1
Phosphate acetyltransferase (EC 2.3.1.8)	1
Propanediol utilization polyhedral body protein PduB	1
Putative iron-containing NADPH-dependent propanol dehydrogenase	1
Ribitol, Xylitol, Arabitol, Mannitol and Sorbitol utilization	10
Fructokinase (EC 2.7.1.4)	1
Oxidoreductase, short chain dehydrogenase/reductase family	1
Ribulose-phosphate 3-epimerase (EC 5.1.3.1)	1
Sorbitol dehydrogenase (EC 1.1.1.14)	1

Sorbitol operon transcription regulator	1
Transcriptional regulator of mannitol utilization, DeoR family protein	1
Transcriptional regulator of various polyols utilization, AraC family	1
Various polyols ABC transporter, periplasmic substrate-binding protein	1
Various polyols ABC transporter, permease component 2	1
Xylitol dehydrogenase (EC 1.1.1.9)	1
**Sugar utilization in Thermotogales**	**49**
Sugar utilization in Thermotogales	49
1-phosphofructokinase (EC 2.7.1.56)	1
2-dehydro-3-deoxygluconate kinase (EC 2.7.1.45)	1
6-phosphogluconate dehydrogenase, decarboxylating (EC 1.1.1.44)	1
6-phosphogluconolactonase (EC 3.1.1.31), eukaryotic type	1
Alpha-galactosidase (EC 3.2.1.22)	1
Alpha-N-arabinofuranosidase (EC 3.2.1.55)	1
Beta-galactosidase (EC 3.2.1.23)	1
Beta-hexosaminidase (EC 3.2.1.52)	1
Beta-xylosidase (EC 3.2.1.37)	1
D-glycerate 2-kinase (EC 2.7.1.-)	1
D-mannonate oxidoreductase (EC 1.1.1.57)	1
Endo-1,4-beta-mannosidase	1
Endo-1,4-beta-xylanase A precursor (EC 3.2.1.8)	1
Enolase (EC 4.2.1.11)	1
Fructokinase (EC 2.7.1.4)	1
Glucokinase (EC 2.7.1.2)	1
Gluconokinase (EC 2.7.1.12)	1
Glucose-6-phosphate 1-dehydrogenase (EC 1.1.1.49)	1
Glucose-6-phosphate isomerase (EC 5.3.1.9)	1
Glycerol kinase (EC 2.7.1.30)	1
Glycerol uptake facilitator protein	1
Glycerol-3-phosphate ABC transporter, periplasmic glycerol-3-phosphate-binding protein (TC 3.A.1.1.3)	1
Glycerol-3-phosphate ABC transporter, permease protein UgpA (TC 3.A.1.1.3)	1
Glycerol-3-phosphate dehydrogenase (EC 1.1.5.3)	1
L-ribulose-5-phosphate 4-epimerase (EC 5.1.3.4)	1
Maltodextrin glucosidase (EC 3.2.1.20)	1
Mannoside ABC transport system, sugar-binding protein	1
Myo-inositol 2-dehydrogenase 1 (EC 1.1.1.18)	1
NAD-dependent glyceraldehyde-3-phosphate dehydrogenase (EC 1.2.1.12)	1

Neopullulanase (EC 3.2.1.135)	1
Novel D-mannonate-D-gluconate epimerase	1
Phosphoenolpyruvate-protein phosphotransferase of PTS system (EC 2.7.3.9)	1
Phosphoglycerate kinase (EC 2.7.2.3)	1
Phosphoglycerate mutase (EC 5.4.2.1)	1
Phosphotransferase system, phosphocarrier protein HPr	1
Polygalacturonase (EC 3.2.1.15)	1
PTS system, fructose-specific IIB component (EC 2.7.1.69)	1
Pullulanase (EC 3.2.1.41)	1
Pyruvate kinase (EC 2.7.1.40)	1
Ribokinase (EC 2.7.1.15)	1
Ribulose-phosphate 3-epimerase (EC 5.1.3.1)	1
Transaldolase (EC 2.2.1.2)	1
Transketolase (EC 2.2.1.1)	1
Transketolase, C-terminal section (EC 2.2.1.1)	1
Triosephosphate isomerase (EC 5.3.1.1)	1
UDP-glucose 4-epimerase (EC 5.1.3.2)	1
Uronate isomerase (EC 5.3.1.12)	1
Xylose ABC transporter, substrate-binding component	1
Xylose-responsive transcription regulator, ROK family	1
**Unknown carbohydrate utilization (cluster Ydj)**	**4**
Unknown carbohydrate utilization (cluster Ydj)	4
Peptide methionine sulfoxide reductase MsrB (EC 1.8.4.12)	1
Putative two-component response regulator and GGDEF family protein YeaJ	1
Uncharacterized protein YeaC	1
UPF0229 protein YeaH	1
**Unknown carbohydrate utilization (cluster Yeg)**	**1**
Unknown carbohydrate utilization (cluster Yeg)	1
Fructose-bisphosphate aldolase class I (EC 4.1.2.13)	1
**VC0266**	**1**
VC0266	1
Hypothetical protein VC0266 (sugar utilization related?)	1
**Cell Division and Cell Cycle**	**40**
**Bacterial Cytoskeleton**	**18**
Bacterial Cytoskeleton	18
Cell division protein FtsA	1
Cell division protein FtsB	1

Cell division protein FtsI [Peptidoglycan synthetase] (EC 2.4.1.129)	1
Cell division protein FtsK	1
Cell division protein FtsL	1
Cell division protein FtsQ	1
Cell division protein FtsW	1
Cell division protein ZipA	1
Cell division topological specificity factor MinE	1
Chromosome (plasmid) partitioning protein ParB	1
Rod shape-determining protein MreB	1
Rod shape-determining protein MreC	1
Rod shape-determining protein MreD	1
Rod shape-determining protein RodA	1
Septum formation protein Maf	1
Septum site-determining protein MinC	1
Septum site-determining protein MinD	1
Z-ring-associated protein ZapA	1
**Control of cell elongation - division cycle in Bacilli**	**2**
Control of cell elongation - division cycle in Bacilli	2
Endonuclease III (EC 4.2.99.18)	1
FIG001721: Predicted N6-adenine-specific DNA methylase	1
**Cyanobacterial Circadian Clock**	**1**
Cyanobacterial Circadian Clock	1
Circadian input kinase A	1
**Intracellular septation in Enterobacteria**	**3**
Intracellular septation in Enterobacteria	3
Acyl-CoA thioesterase YciA, involved in membrane biogenesis	1
Intracellular septation protein IspA	1
Membrane protein YciC, linked to IspA	1
**Macromolecular synthesis operon**	**6**
Macromolecular synthesis operon	6
Carbamoyl-phosphate synthase large chain (EC 6.3.5.5)	1
Carbamoyl-phosphate synthase small chain (EC 6.3.5.5)	1
DNA primase (EC 2.7.7.-)	1
SSU ribosomal protein S21p	1
Transamidase GatB domain protein	1
YgjD/Kae1/Qri7 family, required for threonylcarbamoyladenosine (t(6)A) formation in tRNA	1
**MukBEF Chromosome Condensation**	**4**

MukBEF Chromosome Condensation	4
Chromosome partition protein MukB	1
Chromosome partition protein MukE	1
Membrane Protein Functionally coupled to the MukBEF Chromosome Partitioning Mechanism	1
S-adenosylmethionine-dependent methyltransferase Functionally Coupled to the MukBEF Chromosome Partitioning Mechanism	1
**Two cell division clusters relating to chromosome partitioning**	**5**
Two cell division clusters relating to chromosome partitioning	5
Chromosome (plasmid) partitioning protein ParB	1
Chromosome partition protein smc	1
Ribonuclease III (EC 3.1.26.3)	1
Segregation and condensation protein A	1
Signal recognition particle receptor protein FtsY (=alpha subunit) (TC 3.A.5.1.1)	1
**YgjD and YeaZ**	**1**
YgjD and YeaZ	1
YgjD/Kae1/Qri7 family, required for threonylcarbamoyladenosine (t(6)A) formation in tRNA	1
**Cell Wall and Capsule**	**255**
**Capsular and extracellular polysacchrides**	**79**
Alginate metabolism	1
Phosphomannomutase (EC 5.4.2.8)	1
Capsular heptose biosynthesis	4
D-glycero-D-manno-heptose 1,7-bisphosphate phosphatase (EC 3.1.1.-)	1
dTDP-4-dehydrorhamnose 3,5-epimerase (EC 5.1.3.13)	1
GDP-mannose 4,6-dehydratase (EC 4.2.1.47)	1
Phosphoheptose isomerase (EC 5.3.1.-)	1
Capsular Polysaccharide (CPS) of Campylobacter	1
Capsular polysaccharide export system inner membrane protein KpsE	1
Capsular Polysaccharides Biosynthesis and Assembly	4
Capsular polysaccharide biosynthesis protein WcbQ	1
Capsular polysaccharide export system inner membrane protein KpsE	1
Oxidoreductase, short-chain dehydrogenase/reductase family (EC 1.1.1.-)	1
Tyrosine-protein kinase Wzc (EC 2.7.10.2)	1
CMP-N-acetylneuraminate Biosynthesis	1
N-Acetylneuraminate cytidylyltransferase (EC 2.7.7.43)	1
Colanic acid biosynthesis	4
Colanic acid biosynthesis acetyltransferase WcaF (EC 2.3.1.-)	1
Colanic acid biosynthesis glycosyl transferase WcaE	1
GDP-mannose 4,6-dehydratase (EC 4.2.1.47)	1

Tyrosine-protein kinase Wzc (EC 2.7.10.2)	1
dTDP-rhamnose synthesis	5
dTDP-4-dehydrorhamnose 3,5-epimerase (EC 5.1.3.13)	1
dTDP-4-dehydrorhamnose reductase (EC 1.1.1.133)	1
dTDP-glucose 4,6-dehydratase (EC 4.2.1.46)	1
dTDP-Rha:A-D-GlcNAc-diphosphoryl polyprenol, A-3-L-rhamnosyl transferase WbbL	1
Glucose-1-phosphate thymidylyltransferase (EC 2.7.7.24)	1
Exopolysaccharide Biosynthesis	6
Capsular polysaccharide synthesis enzyme CpsA, sugar transferase	1
Capsular polysaccharide synthesis enzyme CpsB	1
Capsular polysaccharide synthesis enzyme CpsD, exopolysaccharide synthesis	1
Glycosyl transferase, group 2 family protein	1
Manganese-dependent protein-tyrosine phosphatase (EC 3.1.3.48)	1
Tyrosine-protein kinase transmembrane modulator EpsC	1
Extracellular Polysaccharide Biosynthesis of Streptococci	2
Membrane protein CarB involved in the export of O-antigen and teichoic acid, Streptococci specific	1
Tyrosine-protein kinase transmembrane modulator EpsC	1
Legionaminic Acid Biosynthesis	1
UDP-N-acetylglucosamine 4,6-dehydratase (EC 4.2.1.-)	1
Phosphorylcholine incorporation in LPS	1
Lipopolysaccharide cholinephosphotransferase LicD1 (EC 2.7.8.-)	1
Polysaccharide deacetylases	2
Peptidoglycan N-acetylglucosamine deacetylase (EC 3.5.1.-)	1
Polysaccharide deacetylase	1
Rhamnose containing glycans	9
Alpha-L-Rha alpha-1,3-L-rhamnosyltransferase (EC 2.4.1.-)	1
capsular polysaccharide biosynthesis protein	1
DNA for glycosyltransferase, lytic transglycosylase, dTDP-4-rhamnose reductase	1
dTDP-4-dehydrorhamnose 3,5-epimerase (EC 5.1.3.13)	1
dTDP-4-dehydrorhamnose reductase (EC 1.1.1.133)	1
dTDP-glucose 4,6-dehydratase (EC 4.2.1.46)	1
Glucose-1-phosphate thymidylyltransferase (EC 2.7.7.24)	1
Glycerol-3-phosphate cytidylyltransferase (EC 2.7.7.39)	1
UDP-glucose 4-epimerase (EC 5.1.3.2)	1
Sialic Acid Metabolism	14
Glucosamine-6-phosphate deaminase (EC 3.5.99.6)	1
Glucosamine--fructose-6-phosphate aminotransferase [isomerizing] (EC 2.6.1.16)	1

N-acetylmannosamine kinase (EC 2.7.1.60)	1
N-Acetylneuraminate cytidylyltransferase (EC 2.7.7.43)	1
N-acetylneuraminate lyase (EC 4.1.3.3)	1
Phosphoglucosamine mutase (EC 5.4.2.10)	1
Predicted sialic acid transporter	1
PTS system, mannose-specific IIA component (EC 2.7.1.69)	1
PTS system, mannose-specific IIC component (EC 2.7.1.69)	1
PTS system, N-acetylglucosamine-specific IIA component (EC 2.7.1.69)	1
Putative sugar isomerase involved in processing of exogenous sialic acid	1
Sialic acid utilization regulator, RpiR family	1
TRAP-type transport system, large permease component, predicted N-acetylneuraminate transporter	1
TRAP-type transport system, small permease component, predicted N-acetylneuraminate transporter	1
Streptococcal Hyaluronic Acid Capsule	2
UDP-glucose dehydrogenase (EC 1.1.1.22)	1
UTP--glucose-1-phosphate uridylyltransferase (EC 2.7.7.9)	1
Vibrio Polysaccharide (VPS) Biosynthesis	22
CapK protein, putative	1
Capsular polysaccharide synthesis enzyme CpsA, sugar transferase	1
Capsular polysaccharide synthesis enzyme CpsB	1
Capsular polysaccharide synthesis enzyme CpsD, exopolysaccharide synthesis	1
exopolysaccharide biosynthesis protein EpsF, putative	1
Hemolysin-related protein Vcp	1
Hypothetical protein RbmB	1
Hypothetical protein VpsF	1
Hypothetical protein VpsJ	1
Hypothetical protein VpsP	1
Hypothetical protein VpsQ	1
Low molecular weight protein tyrosine phosphatase (EC 3.1.3.48)	1
N-acetylmannosaminyltransferase (EC 2.4.1.187)	1
polysaccharide biosynthesis protein, putative	1
polysaccharide export protein, putative	1
Quorum-sensing regulator of virulence HapR	1
RbmA protein	1
RbmD, similar to Lipid A core - O-antigen ligase and related enzymes	1
Serine acetyltransferase (EC 2.3.1.30)	1
Transcriptional regulator CdgA	1
Transcriptional regulator VpsR	1

Transcriptional regulator VpsT	1
**Cell wall of Mycobacteria**	**10**
linker unit-arabinogalactan synthesis	6
dTDP-4-dehydrorhamnose 3,5-epimerase (EC 5.1.3.13)	1
dTDP-4-dehydrorhamnose reductase (EC 1.1.1.133)	1
dTDP-glucose 4,6-dehydratase (EC 4.2.1.46)	1
Glucose-1-phosphate thymidylyltransferase (EC 2.7.7.24)	1
UDP-galactopyranose mutase (EC 5.4.99.9)	1
UDP-glucose 4-epimerase (EC 5.1.3.2)	1
mycolic acid synthesis	4
3-oxoacyl-[acyl-carrier protein] reductase (EC 1.1.1.100)	1
3-oxoacyl-[acyl-carrier-protein] synthase, KASII (EC 2.3.1.41)	1
Acyl carrier protein	1
Enoyl-[acyl-carrier-protein] reductase [NADH] (EC 1.3.1.9)	1
**Gram-Negative cell wall components**	**94**
A cluster in Borrelia probably related to lipoprotein export	1
Lipoprotein signal peptidase (EC 3.4.23.36)	1
Inner membrane protein YhjD and conserved cluster involved in LPS biosynthesis	3
Inner membrane metabolite transport protein YhjE	1
Protein YhjJ, putative peptidase	1
Uncharacterized protein YhjG	1
KDO2-Lipid A biosynthesis	17
3-deoxy-D-manno-octulosonate 8-phosphate phosphatase (EC 3.1.3.45)	1
3-deoxy-D-manno-octulosonic-acid transferase (EC 2.-.-.-)	1
3-deoxy-manno-octulosonate cytidylyltransferase (EC 2.7.7.38)	1
Acyl-[acyl-carrier-protein]--UDP-N-acetylglucosamine O-acyltransferase (EC 2.3.1.129)	1
Arabinose 5-phosphate isomerase (EC 5.3.1.13)	1
Lipid A biosynthesis (KDO) 2-(lauroyl)-lipid IVA acyltransferase (EC 2.3.1.-)	1
Lipid A biosynthesis lauroyl acyltransferase (EC 2.3.1.-)	1
Lipid A export ATP-binding/permease protein MsbA (EC 3.6.3.25)	1
Lipid-A-disaccharide synthase (EC 2.4.1.182)	1
Lipopolysaccharide ABC transporter, ATP-binding protein LptB	1
LptA, protein essential for LPS transport across the periplasm	1
Predicted hydrolase of the metallo-beta-lactamase superfamily, clustered with KDO2-Lipid A biosynthesis genes	1
regulator of length of O-antigen component of lipopolysaccharide chains	1
Tetraacyldisaccharide 4'-kinase (EC 2.7.1.130)	1
UDP-2,3-diacylglucosamine hydrolase (EC 3.6.1.-)	1

UDP-3-O-[3-hydroxymyristoyl] glucosamine N-acyltransferase (EC 2.3.1.-)	1
UDP-3-O-[3-hydroxymyristoyl] N-acetylglucosamine deacetylase (EC 3.5.1.-)	1
Lipid A modifications	3
Phosphoethanolamine transferase EptA specific for the 1 phosphate group of core-lipid A	1
Sensor protein basS/pmrB (EC 2.7.3.-)	1
Transcriptional regulatory protein PhoP	1
Lipid A-Ara4N pathway (Polymyxin resistance)	3
Polymyxin resistance protein ArnC, glycosyl transferase (EC 2.4.-.-)	1
UDP-4-amino-4-deoxy-L-arabinose--oxoglutarate aminotransferase (EC 2.6.1.-)	1
UDP-glucose dehydrogenase (EC 1.1.1.22)	1
Lipopolysaccharide assembly	14
HtrA protease/chaperone protein	1
Lipopolysaccharide ABC transporter, ATP-binding protein LptB	1
Lipoprotein releasing system transmembrane protein LolC	1
LptA, protein essential for LPS transport across the periplasm	1
Outer membrane lipoprotein carrier protein LolA	1
Outer membrane lipoprotein LolB precursor	1
Outer membrane protein assembly factor YaeT precursor	1
Outer membrane protein YfgL, lipoprotein component of the protein assembly complex (forms a complex with YaeT, YfiO, and NlpB)	1
Probable component of the lipoprotein assembly complex (forms a complex with YaeT, YfgL, and NlpB)	1
Survival protein SurA precursor (Peptidyl-prolyl cis-trans isomerase SurA) (EC 5.2.1.8)	1
Uncharacterized ABC transporter, ATP-binding protein YrbF	1
Uncharacterized ABC transporter, auxiliary component YrbC	1
Uncharacterized ABC transporter, periplasmic component YrbD	1
Uncharacterized protein YrbK clustered with lipopolysaccharide transporters	1
Lipopolysaccharide-related cluster in Alphaproteobacteria	6
3-deoxy-D-manno-octulosonic-acid transferase (EC 2.-.-.-)	1
FIG081201: hypothetical protein	1
Lipid A export ATP-binding/permease protein MsbA (EC 3.6.3.25)	1
Protein of unknown function DUF374	1
Tetraacyldisaccharide 4'-kinase (EC 2.7.1.130)	1
TldE/PmbA protein, part of proposed TldE/TldD proteolytic complex (PMID 12029038)	1
Lipoprotein sorting system	3
Lipoprotein releasing system transmembrane protein LolC	1
Outer membrane lipoprotein carrier protein LolA	1
Outer membrane lipoprotein LolB precursor	1
LOS core oligosaccharide biosynthesis	11

3-deoxy-D-manno-octulosonic-acid transferase (EC 2.-.-.-)	1
ADP-heptose--lipooligosaccharide heptosyltransferase II (EC 2.4.1.-)	1
Beta-1,3-glucosyltransferase	1
D-glycero-D-manno-heptose 1,7-bisphosphate phosphatase (EC 3.1.1.-)	1
Lipopolysaccharide core biosynthesis protein RfaZ	1
Lipopolysaccharide core biosynthesis protein WaaP (EC 2.7.-.-), heptosyl-I-kinase	1
Lipopolysaccharide heptosyltransferase I (EC 2.4.1.-)	1
Lipopolysaccharide heptosyltransferase III (EC 2.4.1.-)	1
O-antigen ligase	1
Phosphoheptose isomerase (EC 5.3.1.-)	1
UDP-galactopyranose mutase (EC 5.4.99.9)	1
Major Outer Membrane Proteins	10
Maltoporin (maltose/maltodextrin high-affinity receptor, phage lambda receptor protein)	1
Osmolarity sensory histidine kinase EnvZ	1
Outer membrane protein A precursor	1
Outer membrane protein C precursor	1
Outer membrane protein H precursor	1
Outer membrane protein N precursor	1
Outer membrane protein OmpK	1
Outer membrane protein OmpT	1
Outer membrane protein OmpV	1
Two-component system response regulator OmpR	1
Outer membrane	4
Outer membrane protein A precursor	1
Outer membrane protein assembly factor YaeT precursor	1
Outer membrane protein C precursor	1
Outer membrane protein H precursor	1
Perosamine Synthesis Vibrio	3
GDP-mannose 4,6-dehydratase (EC 4.2.1.47)	1
O-antigen export system, permease protein	1
Phosphomannomutase (EC 5.4.2.8)	1
Vibrio Core Oligosaccharide Biosynthesis	16
3-deoxy-D-manno-octulosonic acid kinase (EC 2.7.1.-)	1
3-deoxy-D-manno-octulosonic-acid transferase (EC 2.-.-.-)	1
ADP-heptose--lipooligosaccharide heptosyltransferase II (EC 2.4.1.-)	1
ADP-heptose--lipooligosaccharide heptosyltransferase, putative	1
capsular polysaccharide biosynthesis protein	1

capsular polysaccharide biosynthesis protein, putative	1
Lipopolysaccharide biosynthesis glycosyltransferase	1
lipopolysaccharide biosynthesis protein, putative	1
Lipopolysaccharide heptosyltransferase I (EC 2.4.1.-)	1
Lipopolysaccharide synthesis protein WavD	1
Lipopolysaccharide synthesis protein WavE	1
Mannosyltransferase OCH1 and related enzymes	1
Nucleoside-diphosphate-sugar pyrophosphorylase involved in lipopolysaccharide biosynthesis/translation initiation factor 2B, gamma/epsilon subunits (eIF-2Bgamma/eIF-2Bepsilon)	1
O-antigen ligase	1
Polysaccharide deacetylase	1
transferase, hexapeptide repeat family	1
**Gram-Positive cell wall components**	**20**
D-Alanyl Lipoteichoic Acid Biosynthesis	3
D-alanine--poly(phosphoribitol) ligase subunit 1 (EC 6.1.1.13)	1
D-alanyl transfer protein DltB	1
Poly(glycerophosphate chain) D-alanine transfer protein DltD	1
Polyglycerolphosphate lipoteichoic acid biosynthesis	2
Lipoteichoic acid synthase LtaS Type IIb	1
Lipoteichoic acid synthase LtaS Type IIc	1
Sortase	2
NPQTN specific sortase B	1
Sortase A, LPXTG specific	1
Teichoic and lipoteichoic acids biosynthesis	11
2-C-methyl-D-erythritol 4-phosphate cytidylyltransferase (EC 2.7.7.60)	1
CDP-glycerol: N-acetyl-beta-D-mannosaminyl-1,4-N-acetyl-D-glucosaminyldiphosphoundecaprenyl glycerophosphotransferase	1
COG1887: Putative glycosyl/glycerophosphate transferases involved in teichoic acid biosynthesis TagF/TagB/EpsJ/RodC	1
D-alanine--poly(phosphoribitol) ligase subunit 1 (EC 6.1.1.13)	1
D-alanyl transfer protein DltB	1
Membrane protein CarB involved in the export of O-antigen and teichoic acid, Streptococci specific	1
N-acetylmannosaminyltransferase (EC 2.4.1.187)	1
Poly(glycerophosphate chain) D-alanine transfer protein DltD	1
Regulation of D-alanyl-lipoteichoic acid biosynthesis, DltR	1
Teichoic acid biosynthesis protein	1
Undecaprenyl-phosphate N-acetylglucosaminyl 1-phosphate transferase (EC 2.7.8.-)	1
Teichuronic acid biosynthesis	2
Putative N-acetylgalactosaminyl-diphosphoundecaprenol glucuronosyltransferase	1
UDP-glucose dehydrogenase (EC 1.1.1.22)	1

**Murein Hydrolases**	**8**
Murein Hydrolases	8
Beta N-acetyl-glucosaminidase (EC 3.2.1.52)	1
Membrane-bound lytic murein transglycosylase B (EC 3.2.1.-)	1
Membrane-bound lytic murein transglycosylase B precursor (EC 3.2.1.-)	1
Membrane-bound lytic murein transglycosylase C precursor (EC 3.2.1.-)	1
Membrane-bound lytic murein transglycosylase D precursor (EC 3.2.1.-)	1
Membrane-bound lytic murein transglycosylase E (EC 3.2.1.-)	1
Muramoyltetrapeptide carboxypeptidase (EC 3.4.17.13)	1
N-acetylmuramoyl-L-alanine amidase (EC 3.5.1.28)	1
**Peptidoglycan Biosynthesis**	**19**
Peptidoglycan Biosynthesis	19
Cell division protein FtsI [Peptidoglycan synthetase] (EC 2.4.1.129)	1
D-alanine--D-alanine ligase (EC 6.3.2.4)	1
Glutamine synthetase type III, GlnN (EC 6.3.1.2)	1
Glutamine synthetase, clostridia type (EC 6.3.1.2)	1
Membrane-bound lytic murein transglycosylase B (EC 3.2.1.-)	1
Membrane-bound lytic murein transglycosylase B precursor (EC 3.2.1.-)	1
Monofunctional biosynthetic peptidoglycan transglycosylase (EC 2.4.2.-)	1
Multimodular transpeptidase-transglycosylase (EC 2.4.1.129) (EC 3.4.-.-)	1
Murein-DD-endopeptidase (EC 3.4.99.-)	1
N-acetylglucosamine-1-phosphate uridyltransferase eukaryotic (EC 2.7.7.23)	1
Penicillin-binding protein 2 (PBP-2)	1
Penicillin-insensitive transglycosylase (EC 2.4.2.-) & transpeptidase PBP-1C	1
Phospho-N-acetylmuramoyl-pentapeptide-transferase (EC 2.7.8.13)	1
Rod shape-determining protein RodA	1
UDP-N-acetylenolpyruvoylglucosamine reductase (EC 1.1.1.158)	1
UDP-N-acetylglucosamine 1-carboxyvinyltransferase (EC 2.5.1.7)	1
UDP-N-acetylmuramate--alanine ligase (EC 6.3.2.8)	1
UDP-N-acetylmuramoylalanine--D-glutamate ligase (EC 6.3.2.9)	1
UDP-N-acetylmuramoylalanyl-D-glutamate--2,6-diaminopimelate ligase (EC 6.3.2.13)	1
**Peptidoglycan biosynthesis--gjo**	**6**
Peptidoglycan biosynthesis--gjo	6
D-alanine--D-alanine ligase (EC 6.3.2.4)	1
UDP-N-acetylmuramate:L-alanyl-gamma-D-glutamyl-meso-diaminopimelate ligase (EC 6.3.2.-)	1
UDP-N-acetylmuramate--alanine ligase (EC 6.3.2.8)	1
UDP-N-acetylmuramoylalanine--D-glutamate ligase (EC 6.3.2.9)	1

UDP-N-acetylmuramoylalanyl-D-glutamate--2,6-diaminopimelate ligase (EC 6.3.2.13)	1
UDP-N-acetylmuramoylalanyl-D-glutamate--L-ornithine ligase	1
**Peptidoglycan Crosslinking of Peptide Stems**	**1**
Peptidoglycan Crosslinking of Peptide Stems	1
L,D-transpeptidase YcbB	1
**Recycling of Peptidoglycan Amino Acids**	**6**
Recycling of Peptidoglycan Amino Acids	6
Aminoacyl-histidine dipeptidase (Peptidase D) (EC 3.4.13.3)	1
Gamma-D-Glutamyl-meso-Diaminopimelate Amidase	1
Muramoyltetrapeptide carboxypeptidase (EC 3.4.17.13)	1
N-acetylmuramoyl-L-alanine amidase (EC 3.5.1.28)	1
N-acetylmuramoyl-L-alanine amidase (EC 3.5.1.28) AmpD	1
UDP-N-acetylmuramate:L-alanyl-gamma-D-glutamyl-meso-diaminopimelate ligase (EC 6.3.2.-)	1
**Recycling of Peptidoglycan Amino Sugars**	**3**
Recycling of Peptidoglycan Amino Sugars	3
Anhydro-N-acetylmuramic acid kinase (EC 2.7.1.-)	1
Beta N-acetyl-glucosaminidase (EC 3.2.1.52)	1
N-acetylmuramic acid 6-phosphate etherase (EC 4.2.-.-)	1
**UDP-N-acetylmuramate from Fructose-6-phosphate Biosynthesis**	**7**
UDP-N-acetylmuramate from Fructose-6-phosphate Biosynthesis	7
Glucosamine-6-phosphate deaminase (EC 3.5.99.6)	1
Glucosamine--fructose-6-phosphate aminotransferase [isomerizing] (EC 2.6.1.16)	1
N-acetylglucosamine-1-phosphate uridyltransferase eukaryotic (EC 2.7.7.23)	1
N-acetylmuramic acid 6-phosphate etherase (EC 4.2.-.-)	1
Phosphoglucosamine mutase (EC 5.4.2.10)	1
UDP-N-acetylenolpyruvoylglucosamine reductase (EC 1.1.1.158)	1
UDP-N-acetylglucosamine 1-carboxyvinyltransferase (EC 2.5.1.7)	1
**YjeE**	**2**
YjeE	2
COG3178: Predicted phosphotransferase related to Ser/Thr protein kinases	1
YjeF protein, function unknown	1
**Cofactors, Vitamins, Prosthetic Groups, Pigments**	**327**
**Biotin**	**7**
Biotin biosynthesis	7
Biotin synthase (EC 2.8.1.6)	1
Biotin synthesis protein bioC	1
Biotin synthesis protein bioH	1

Dethiobiotin synthetase (EC 6.3.3.3)	1
Long-chain-fatty-acid--CoA ligase (EC 6.2.1.3)	1
Substrate-specific component BioY of biotin ECF transporter	1
Transmembrane component BioN of energizing module of biotin ECF transporter	1
**Coenzyme A**	**9**
Coenzyme A Biosynthesis	9
2-dehydropantoate 2-reductase (EC 1.1.1.169)	1
3-methyl-2-oxobutanoate hydroxymethyltransferase (EC 2.1.2.11)	1
Dephospho-CoA kinase (EC 2.7.1.24)	1
Pantoate--beta-alanine ligase (EC 6.3.2.1)	1
Pantothenate kinase (EC 2.7.1.33)	1
Pantothenate kinase type III, CoaX-like (EC 2.7.1.33)	1
Pantothenate:Na+ symporter (TC 2.A.21.1.1)	1
Phosphopantetheine adenylyltransferase (EC 2.7.7.3)	1
Substrate-specific component PanT of predicted pantothenate ECF transporter	1
**Coenzyme M**	**1**
coenzyme M biosynthesis	1
2-phosphosulfolactate phosphatase (EC 3.1.3.71)	1
**Fe-S clusters**	**11**
Fe-S cluster assembly	11
Chaperone protein HscB	1
Cysteine desulfurase (EC 2.8.1.7), IscS subfamily	1
Cysteine desulfurase (EC 2.8.1.7), SufS subfamily	1
Iron binding protein IscA for iron-sulfur cluster assembly	1
Iron-sulfur cluster assembly protein SufB	1
Iron-sulfur cluster assembly protein SufD	1
Iron-sulfur cluster assembly scaffold protein IscU	1
Iron-sulfur cluster assembly scaffold protein NifU	1
PaaD-like protein (DUF59) involved in Fe-S cluster assembly	1
probable iron binding protein from the HesB_IscA_SufA family	1
tRNA S(4)U 4-thiouridine synthase (former ThiI)	1
**Folate and pterines**	**120**
5-FCL-like protein	14
5,10-methylenetetrahydrofolate reductase (EC 1.5.1.20)	1
5-formyltetrahydrofolate cyclo-ligase (EC 6.3.3.2)	1
Alcohol dehydrogenase (EC 1.1.1.1)	1
Dihydrofolate reductase (EC 1.5.1.3)	1

Dihydrolipoamide acetyltransferase component of pyruvate dehydrogenase complex (EC 2.3.1.12)	1
Formate--tetrahydrofolate ligase (EC 6.3.4.3)	1
Formyltetrahydrofolate deformylase (EC 3.5.1.10)	1
Methylenetetrahydrofolate dehydrogenase (NADP+) (EC 1.5.1.5) / Methenyltetrahydrofolate cyclohydrolase (EC 3.5.4.9)	1
Phosphoribosylglycinamide formyltransferase (EC 2.1.2.2)	1
Pyruvate dehydrogenase E1 component (EC 1.2.4.1)	1
Substrate-specific component ThiW of predicted thiazole ECF transporter	1
Succinate dehydrogenase iron-sulfur protein (EC 1.3.99.1)	1
Thiaminase II (EC 3.5.99.2)	1
Thiamin-phosphate pyrophosphorylase (EC 2.5.1.3)	1
Folate Biosynthesis	19
2-amino-4-hydroxy-6-hydroxymethyldihydropteridine pyrophosphokinase (EC 2.7.6.3)	1
5-formyltetrahydrofolate cyclo-ligase (EC 6.3.3.2)	1
5-nucleotidase SurE (EC 3.1.3.5)	1
alternate gene name: yzbB	1
ATPase component of general energizing module of ECF transporters	1
COG0488: ATPase components of ABC transporters with duplicated ATPase domains	1
Dihydrofolate reductase (EC 1.5.1.3)	1
Dihydroneopterin aldolase (EC 4.1.2.25)	1
Folate transporter 3	1
FolM Alternative dihydrofolate reductase 1	1
Formate--tetrahydrofolate ligase (EC 6.3.4.3)	1
GTP cyclohydrolase I (EC 3.5.4.16) type 1	1
GTP cyclohydrolase I (EC 3.5.4.16) type 2	1
Para-aminobenzoate synthase, amidotransferase component (EC 2.6.1.85)	1
Para-aminobenzoate synthase, aminase component (EC 2.6.1.85)	1
Thymidylate synthase (EC 2.1.1.45)	1
Thymidylate synthase thyX (EC 2.1.1.-)	1
Transaldolase (EC 2.2.1.2)	1
Transmembrane component of general energizing module of ECF transporters	1
Methanopterin biosynthesis2	5
2-amino-4-hydroxy-6-hydroxymethyldihydropteridine pyrophosphokinase (EC 2.7.6.3)	1
Dihydroneopterin aldolase (EC 4.1.2.25)	1
GTP cyclohydrolase I (EC 3.5.4.16) type 1	1
GTP cyclohydrolase I (EC 3.5.4.16) type 2	1
Queuosine biosynthesis QueD, PTPS-I	1
Molybdenum cofactor biosynthesis	13

GTP cyclohydrolase I (EC 3.5.4.16) type 1	1
Molybdate-binding domain of ModE	1
Molybdenum ABC transporter, periplasmic molybdenum-binding protein ModA (TC 3.A.1.8.1)	1
Molybdenum cofactor biosynthesis protein MoaA	1
Molybdenum cofactor biosynthesis protein MoaC	1
Molybdenum cofactor biosynthesis protein MoaD	1
Molybdenum cofactor biosynthesis protein MoaE	1
Molybdenum transport ATP-binding protein ModC (TC 3.A.1.8.1)	1
Molybdenum transport system permease protein ModB (TC 3.A.1.8.1)	1
Molybdopterin biosynthesis enzyme	1
Molybdopterin biosynthesis protein MoeA	1
Molybdopterin biosynthesis protein MoeB	1
Molybdopterin-guanine dinucleotide biosynthesis protein MobA	1
Pterin biosynthesis	3
GTP cyclohydrolase I (EC 3.5.4.16) type 1	1
Phenylalanine-4-hydroxylase (EC 1.14.16.1)	1
Pterin-4-alpha-carbinolamine dehydratase (EC 4.2.1.96)	1
Pterin carbinolamine dehydratase	5
Aromatic-amino-acid aminotransferase (EC 2.6.1.57)	1
Fumarylacetoacetase (EC 3.7.1.2)	1
Fumarylacetoacetate hydrolase family protein	1
Phenylalanine-4-hydroxylase (EC 1.14.16.1)	1
Pterin-4-alpha-carbinolamine dehydratase (EC 4.2.1.96)	1
Pterin metabolism	3
GTP cyclohydrolase I (EC 3.5.4.16) type 1	1
Phenylalanine-4-hydroxylase (EC 1.14.16.1)	1
Pterin-4-alpha-carbinolamine dehydratase (EC 4.2.1.96)	1
Pterin metabolism 3	13
2-amino-4-hydroxy-6-hydroxymethyldihydropteridine pyrophosphokinase (EC 2.7.6.3)	1
Acetoacetyl-CoA synthetase (EC 6.2.1.16)	1
Aromatic-amino-acid aminotransferase (EC 2.6.1.57)	1
COG1565: Uncharacterized conserved protein	1
COGs COG3146	1
Dihydroneopterin aldolase (EC 4.1.2.25)	1
FolM Alternative dihydrofolate reductase 1	1
Fumarylacetoacetase (EC 3.7.1.2)	1
Fumarylacetoacetate hydrolase family protein	1

GTP cyclohydrolase I (EC 3.5.4.16) type 1	1
GTP cyclohydrolase I (EC 3.5.4.16) type 2	1
Phenylalanine-4-hydroxylase (EC 1.14.16.1)	1
Pterin-4-alpha-carbinolamine dehydratase (EC 4.2.1.96)	1
YgfZ	37
2-amino-4-hydroxy-6-hydroxymethyldihydropteridine pyrophosphokinase (EC 2.7.6.3)	1
5,10-methylenetetrahydrofolate reductase (EC 1.5.1.20)	1
Aconitate hydratase (EC 4.2.1.3)	1
Aconitate hydratase 2 (EC 4.2.1.3)	1
Adenosylcobinamide-phosphate synthase	1
Biotin synthase (EC 2.8.1.6)	1
Chorismate synthase (EC 4.2.3.5)	1
Coproporphyrinogen III oxidase, oxygen-independent (EC 1.3.99.22)	1
Cystathionine gamma-synthase (EC 2.5.1.48)	1
Cysteine desulfurase (EC 2.8.1.7), IscS subfamily	1
Cysteine desulfurase (EC 2.8.1.7), SufS subfamily	1
Dihydroorotase (EC 3.5.2.3)	1
Dihydroorotate dehydrogenase (EC 1.3.3.1)	1
Dihydroorotate dehydrogenase, catalytic subunit (EC 1.3.3.1)	1
Ferredoxin	1
Ferric uptake regulation protein FUR	1
Flavodoxin reductases (ferredoxin-NADPH reductases) family 1	1
Folate-dependent protein for Fe/S cluster synthesis/repair in oxidative stress	1
Formate dehydrogenase-O, major subunit (EC 1.2.1.2)	1
Fumarate hydratase class I, aerobic (EC 4.2.1.2)	1
Gamma-glutamyl phosphate reductase (EC 1.2.1.41)	1
Glutamate--cysteine ligase (EC 6.3.2.2)	1
Iron binding protein IscA for iron-sulfur cluster assembly	1
L-aspartate oxidase (EC 1.4.3.16)	1
Lipoate synthase	1
Molybdenum cofactor biosynthesis protein MoaA	1
NADH-ubiquinone oxidoreductase chain F (EC 1.6.5.3)	1
Phosphoglycerate kinase (EC 2.7.2.3)	1
Phosphoribosylformylglycinamidine cyclo-ligase (EC 6.3.3.1)	1
Queuosine Biosynthesis QueC ATPase	1
Quinolinate synthetase (EC 2.5.1.72)	1
Rhodanese-related sulfurtransferase	1

Succinate dehydrogenase iron-sulfur protein (EC 1.3.99.1)	1
Thiazole biosynthesis protein ThiH	1
tRNA-i(6)A37 methylthiotransferase	1
Twin-arginine translocation protein TatA	1
YgfY COG2938	1
YgfZ-Iron	8
COG1272: Predicted membrane protein hemolysin III homolog	1
Cytochrome c heme lyase subunit CcmF	1
Dihydroorotase (EC 3.5.2.3)	1
Ferric uptake regulation protein FUR	1
Folate-dependent protein for Fe/S cluster synthesis/repair in oxidative stress	1
Heme O synthase, protoheme IX farnesyltransferase (EC 2.5.1.-) COX10-CtaB	1
TonB-dependent receptor; Outer membrane receptor for ferrienterochelin and colicins	1
Tricarboxylate transport membrane protein TctA	1
**Lipoic acid**	**4**
Lipoic acid metabolism	4
Lipoate synthase	1
Lipoate-protein ligase A	1
Octanoate-[acyl-carrier-protein]-protein-N-octanoyltransferase	1
Proposed lipoate regulatory protein YbeD	1
**Molybdopterin cytosine dinucleotide**	**3**
Molybdopterin cytosine dinucleotide	3
CTP:molybdopterin cytidylyltransferase	1
Molybdopterin biosynthesis enzyme	1
Periplasmic aromatic aldehyde oxidoreductase, FAD binding subunit YagS	1
**NAD and NADP**	**23**
NAD and NADP cofactor biosynthesis global	12
ADP-ribose pyrophosphatase (EC 3.6.1.13)	1
Kynureninase (EC 3.7.1.3)	1
Kynurenine formamidase, bacterial (EC 3.5.1.9)	1
L-aspartate oxidase (EC 1.4.3.16)	1
Niacin transporter NiaP	1
Nicotinamidase (EC 3.5.1.19)	1
Nicotinate phosphoribosyltransferase (EC 2.4.2.11)	1
Nicotinate-nucleotide adenylyltransferase (EC 2.7.7.18)	1
Predicted N-ribosylNicotinamide CRP-like regulator	1
Quinolinate phosphoribosyltransferase [decarboxylating] (EC 2.4.2.19)	1

Quinolinate synthetase (EC 2.5.1.72)	1
Ribosyl nicotinamide transporter, PnuC-like	1
NAD regulation	8
L-aspartate oxidase (EC 1.4.3.16)	1
Niacin transporter NiaP	1
Nicotinamidase (EC 3.5.1.19)	1
Nicotinate phosphoribosyltransferase (EC 2.4.2.11)	1
Nicotinate-nucleotide adenylyltransferase (EC 2.7.7.18)	1
Quinolinate phosphoribosyltransferase [decarboxylating] (EC 2.4.2.19)	1
Quinolinate synthetase (EC 2.5.1.72)	1
Ribosyl nicotinamide transporter, PnuC-like	1
PnuC-like transporters	3
Predicted N-ribosylNicotinamide CRP-like regulator	1
Ribosyl nicotinamide transporter, PnuC-like	1
Thiamin pyrophosphokinase (EC 2.7.6.2)	1
**Pyridoxine**	**10**
Pyridoxin (Vitamin B6) Biosynthesis	10
1-deoxy-D-xylulose 5-phosphate synthase (EC 2.2.1.7)	1
4-hydroxythreonine-4-phosphate dehydrogenase (EC 1.1.1.262)	1
D-3-phosphoglycerate dehydrogenase (EC 1.1.1.95)	1
Erythronate-4-phosphate dehydrogenase (EC 1.1.1.290)	1
NAD-dependent glyceraldehyde-3-phosphate dehydrogenase (EC 1.2.1.12)	1
Predicted transcriptional regulator of pyridoxine metabolism	1
Pyridoxal kinase (EC 2.7.1.35)	1
Pyridoxamine 5'-phosphate oxidase (EC 1.4.3.5)	1
Pyridoxine 5'-phosphate synthase (EC 2.6.99.2)	1
Pyridoxine biosynthesis glutamine amidotransferase, glutaminase subunit (EC 2.4.2.-)	1
**Quinone cofactors**	**24**
Coenzyme PQQ synthesis	1
Coenzyme PQQ synthesis protein F (EC 3.4.99.-)	1
Menaquinone and Phylloquinone Biosynthesis	7
1,4-dihydroxy-2-naphthoate octaprenyltransferase (EC 2.5.1.74)	1
2-succinyl-5-enolpyruvyl-6-hydroxy-3-cyclohexene-1-carboxylic-acid synthase (EC 2.2.1.9)	1
2-succinyl-6-hydroxy-2,4-cyclohexadiene-1-carboxylate synthase (EC 4.2.99.20)	1
Menaquinone-specific isochorismate synthase (EC 5.4.4.2)	1
Naphthoate synthase (EC 4.1.3.36)	1
O-succinylbenzoate-CoA synthase (EC 4.2.1.-)	1

Ubiquinone/menaquinone biosynthesis methyltransferase UbiE (EC 2.1.1.-)	1
Menaquinone Biosynthesis via Futalosine	2
Menaquinone via futalosine step 4	1
Ubiquinone/menaquinone biosynthesis methyltransferase UbiE (EC 2.1.1.-)	1
Menaquinone Biosynthesis via Futalosine -- gjo	3
4-hydroxybenzoate polyprenyltransferase (EC 2.5.1.-)	1
Menaquinone via futalosine step 4	1
Ubiquinone/menaquinone biosynthesis methyltransferase UbiE (EC 2.1.1.-)	1
Plastoquinone Biosynthesis	1
Homogentisate prenyltransferase	1
Pyrroloquinoline Quinone biosynthesis	1
Coenzyme PQQ synthesis protein F (EC 3.4.99.-)	1
Tocopherol Biosynthesis	1
Homogentisate prenyltransferase	1
Ubiquinone Biosynthesis	8
2-octaprenyl-3-methyl-6-methoxy-1,4-benzoquinol hydroxylase (EC 1.14.13.-)	1
2-octaprenyl-6-methoxyphenol hydroxylase (EC 1.14.13.-)	1
3-demethylubiquinone-9 3-methyltransferase (EC 2.1.1.64)	1
4-hydroxybenzoate polyprenyltransferase (EC 2.5.1.-)	1
Chorismate--pyruvate lyase (EC 4.1.3.40)	1
NAD(P)H-flavin reductase (EC 1.5.1.29) (EC 1.16.1.3)	1
Ubiquinone biosynthesis monooxygenase UbiB	1
Ubiquinone/menaquinone biosynthesis methyltransferase UbiE (EC 2.1.1.-)	1
**Riboflavin, FMN, FAD**	**7**
Flavodoxin	3
Flavodoxin 1	1
Flavodoxin 2	1
Flavoprotein MioC	1
riboflavin to FAD	1
Riboflavin kinase (EC 2.7.1.26)	1
Riboflavin, FMN and FAD metabolism	3
6,7-dimethyl-8-ribityllumazine synthase (EC 2.5.1.78)	1
Pyrimidine deaminase archaeal predicted (EC 3.5.4.26)	1
Riboflavin kinase (EC 2.7.1.26)	1
**Tetrapyrroles**	**108**
Chlorophyll Biosynthesis	8
2-desacetyl-2-hydroxyethyl bacteriochlorophyllide A dehydrogenase BchC	1

2-vinyl bacteriochlorophyllide hydratase BchF (EC 4.2.1.-)	1
Chlorophyllide reductase subunit BchY (EC 1.18.-.-)	1
Light-dependent protochlorophyllide reductase (EC 1.3.1.33)	1
Light-independent protochlorophyllide reductase subunit N (EC 1.18.-.-)	1
Mg-protoporphyrin IX monomethyl ester oxidative cyclase (anaerobic) (EC 1.14.13.81)	1
Protoporphyrin IX Mg-chelatase subunit D (EC 6.6.1.1)	1
Putative chaperon-like protein Ycf39 for quinone binding in Photosystem II	1
Chlorophyll Degradation	1
Pheophorbide a oxygenase (EC 1.14.-.-)	1
Cobalamin synthesis	13
Adenosylcobinamide-phosphate guanylyltransferase (EC 2.7.7.62)	1
Adenosylcobinamide-phosphate synthase	1
Alpha-ribazole-5'-phosphate phosphatase (EC 3.1.3.73)	1
Cob(I)alamin adenosyltransferase (EC 2.5.1.17)	1
Cobalamin biosynthesis protein CbiG	1
Cobalamin synthase	1
Cobalt-precorrin-2 C20-methyltransferase (EC 2.1.1.130)	1
Cobalt-precorrin-4 C11-methyltransferase (EC 2.1.1.133)	1
Cobalt-precorrin-8x methylmutase (EC 5.4.1.2)	1
Cobyric acid synthase	1
Cobyrinic acid A,C-diamide synthase	1
Nicotinate-nucleotide--dimethylbenzimidazole phosphoribosyltransferase (EC 2.4.2.21)	1
Sirohydrochlorin cobaltochelatase CbiK (EC 4.99.1.3)	1
Coenzyme B12 biosynthesis	25
Adenosylcobinamide-phosphate guanylyltransferase (EC 2.7.7.62)	1
Adenosylcobinamide-phosphate synthase	1
Aerobic cobaltochelatase CobS subunit (EC 6.6.1.2)	1
Alpha-ribazole-5'-phosphate phosphatase (EC 3.1.3.73)	1
ATPase component CbiO of energizing module of cobalt ECF transporter	1
CblX, a non-orthologous displasment for Alpha-ribazole-5'-phosphate phosphatase	1
CblZ, a non-orthologous displasment for Alpha-ribazole-5'-phosphate phosphatase	1
Cob(I)alamin adenosyltransferase (EC 2.5.1.17)	1
Cob(I)alamin adenosyltransferase PduO (EC 2.5.1.17)	1
Cobalamin biosynthesis protein CbiG	1
Cobalamin synthase	1
Cobalt-precorrin-2 C20-methyltransferase (EC 2.1.1.130)	1
Cobalt-precorrin-4 C11-methyltransferase (EC 2.1.1.133)	1

Cobalt-precorrin-8x methylmutase (EC 5.4.1.2)	1
CobW GTPase involved in cobalt insertion for B12 biosynthesis	1
Cobyric acid synthase	1
Cobyrinic acid A,C-diamide synthase	1
Nicotinate-nucleotide--dimethylbenzimidazole phosphoribosyltransferase (EC 2.4.2.21)	1
Outer membrane vitamin B12 receptor BtuB	1
Sirohydrochlorin cobaltochelatase CbiK (EC 4.99.1.3)	1
Transmembrane component CbiQ of energizing module of cobalt ECF transporter	1
Transmembrane component CbrV of energizing module of predicted cobalamin ECF transporter	1
Uroporphyrinogen-III methyltransferase (EC 2.1.1.107)	1
Vitamin B12 ABC transporter, B12-binding component BtuF	1
Vitamin B12 ABC transporter, permease component BtuC	1
CPO analysis	21
5-aminolevulinate synthase (EC 2.3.1.37)	1
Coproporphyrinogen III oxidase, aerobic (EC 1.3.3.3)	1
Coproporphyrinogen III oxidase, oxygen-independent (EC 1.3.99.22)	1
DUF1801 domain-containing protein	1
Glutamate-1-semialdehyde aminotransferase (EC 5.4.3.8)	1
Glutamyl-tRNA reductase (EC 1.2.1.70)	1
Hypothetical protein, ydbT homolog	1
Hypothetical radical SAM family enzyme, NOT coproporphyrinogen III oxidase, oxygen-independent	1
Membrane protein, distant similarity to thiosulphate:quinone oxidoreductase DoxD	1
Periplasmic/membrane protein associated with DUF414	1
Porphobilinogen deaminase (EC 2.5.1.61)	1
Porphobilinogen synthase (EC 4.2.1.24)	1
Possibly related to ydbT	1
Protein of unknown function DUF414	1
Protoporphyrinogen IX oxidase, aerobic (EC 1.3.3.4)	1
Protoporphyrinogen IX oxidase, oxygen-independent, HemG (EC 1.3.-.-)	1
Putative coproporphyrinogen III oxidase of BS HemN-type, oxygen-independent (EC 1.3.99.22), in heat shock gene cluster	1
Uroporphyrinogen III decarboxylase (EC 4.1.1.37)	1
Uroporphyrinogen-III methyltransferase (EC 2.1.1.107)	1
Uroporphyrinogen-III synthase (EC 4.2.1.75)	1
YjbH-like, GTP pyrophosphokinase domain	1
Experimental tye	23
5-aminolevulinate synthase (EC 2.3.1.37)	1
Acyl-coenzyme A synthetases/AMP-(fatty) acid ligases, YtcI homolog	1

Coproporphyrinogen III oxidase, aerobic (EC 1.3.3.3)	1
Coproporphyrinogen III oxidase, oxygen-independent (EC 1.3.99.22)	1
Cytochrome c-type biogenesis protein CcdA (DsbD analog)	1
Cytochrome c-type biogenesis protein CcsA/ResC	1
DUF1801 domain-containing protein	1
Glutamate-1-semialdehyde aminotransferase (EC 5.4.3.8)	1
Glutamyl-tRNA reductase (EC 1.2.1.70)	1
Homolog of E. coli HemY protein	1
Hypothetical protein, ydbT homolog	1
Periplasmic/membrane protein associated with DUF414	1
Porphobilinogen deaminase (EC 2.5.1.61)	1
Porphobilinogen synthase (EC 4.2.1.24)	1
Possibly related to ydbT	1
Protein of unknown function DUF414	1
Protoporphyrinogen IX oxidase, aerobic (EC 1.3.3.4)	1
Protoporphyrinogen IX oxidase, oxygen-independent, HemG (EC 1.3.-.-)	1
Ribonuclease D (EC 3.1.26.3)	1
Sua5 YciO YrdC YwlC family protein	1
Uroporphyrinogen III decarboxylase (EC 4.1.1.37)	1
Uroporphyrinogen-III methyltransferase (EC 2.1.1.107)	1
Uroporphyrinogen-III synthase (EC 4.2.1.75)	1
Heme and Siroheme Biosynthesis	17
5-aminolevulinate synthase (EC 2.3.1.37)	1
Coproporphyrinogen III oxidase, aerobic (EC 1.3.3.3)	1
Coproporphyrinogen III oxidase, oxygen-independent (EC 1.3.99.22)	1
Distant homolog of E. coli HemX protein in Xanthomonadaceae	1
Glutamate-1-semialdehyde aminotransferase (EC 5.4.3.8)	1
Glutamyl-tRNA reductase (EC 1.2.1.70)	1
Glutamyl-tRNA synthetase (EC 6.1.1.17)	1
Hypothetical radical SAM family enzyme, NOT coproporphyrinogen III oxidase, oxygen-independent	1
Porphobilinogen deaminase (EC 2.5.1.61)	1
Porphobilinogen synthase (EC 4.2.1.24)	1
Protoporphyrinogen IX oxidase, aerobic (EC 1.3.3.4)	1
Protoporphyrinogen IX oxidase, oxygen-independent, HemG (EC 1.3.-.-)	1
Putative coproporphyrinogen III oxidase of BS HemN-type, oxygen-independent (EC 1.3.99.22), in heat shock gene cluster	1
Radical SAM family protein HutW, similar to coproporphyrinogen III oxidase, oxygen-independent, associated with heme uptake	1
Uroporphyrinogen III decarboxylase (EC 4.1.1.37)	1

Uroporphyrinogen-III methyltransferase (EC 2.1.1.107)	1
Uroporphyrinogen-III synthase (EC 4.2.1.75)	1
**DNA Metabolism**	**169**
**CRISPs**	**8**
CRISPR-associated cluster	2
CRISPR-associated protein, Csm1 family	1
DUF324 domain-containing protein	1
CRISPRs	6
CRISPR-associated helicase Cas3	1
CRISPR-associated helicase Cas3, protein	1
CRISPR-associated protein Cas2	1
CRISPR-associated protein, CT1133 family	1
CRISPR-associated protein, CT1975 family	1
CRISPR-associated protein, TM1801 family	1
**DNA phosphorothioation**	**1**
DNA phosphorothioation	1
DNA sulfur modification protein DndD	1
**DNA recombination**	**4**
RuvABC plus a hypothetical	4
Crossover junction endodeoxyribonuclease RuvC (EC 3.1.22.4)	1
FIG000859: hypothetical protein	1
Holliday junction DNA helicase RuvA	1
Holliday junction DNA helicase RuvB	1
**DNA repair**	**83**
2-phosphoglycolate salvage	4
2-deoxyglucose-6-phosphate hydrolase YniC	1
Putative phosphatase YieH	1
Putative phosphatase YqaB	1
Similar to phosphoglycolate phosphatase, clustered with ribosomal large subunit pseudouridine synthase C	1
ATP-dependent Nuclease	1
ATP-dependent nuclease, subunit A	1
DNA Repair Base Excision	11
ATP-dependent DNA ligase (EC 6.5.1.1)	1
ATP-dependent DNA ligase (EC 6.5.1.1) clustered with Ku protein, LigD	1
ATP-dependent DNA ligase (EC 6.5.1.1) LigC	1
DNA polymerase I (EC 2.7.7.7)	1
DNA-3-methyladenine glycosylase (EC 3.2.2.20)	1

Endonuclease III (EC 4.2.99.18)	1
Exodeoxyribonuclease I (EC 3.1.11.1)	1
Formamidopyrimidine-DNA glycosylase (EC 3.2.2.23)	1
Ku domain protein	1
Single-stranded-DNA-specific exonuclease RecJ (EC 3.1.-.-)	1
Uracil-DNA glycosylase, family 1	1
DNA repair system including RecA, MutS and a hypothetical protein	3
DNA mismatch repair protein MutS	1
RecA protein	1
Regulatory protein RecX	1
DNA repair, bacterial	21
ADA regulatory protein	1
Alkylated DNA repair protein AlkB	1
DNA mismatch repair endonuclease MutH	1
DNA polymerase IV (EC 2.7.7.7)	1
DNA recombination protein RmuC	1
DNA recombination-dependent growth factor C	1
DNA repair protein RadA	1
DNA repair protein RecN	1
DNA-damage-inducible protein I	1
DNA-damage-inducible protein J	1
Endonuclease IV (EC 3.1.21.2)	1
Exodeoxyribonuclease VII large subunit (EC 3.1.11.6)	1
Exodeoxyribonuclease VII small subunit (EC 3.1.11.6)	1
Exonuclease SbcD	1
G:T/U mismatch-specific uracil/thymine DNA-glycosylase	1
Methylated-DNA--protein-cysteine methyltransferase (EC 2.1.1.63)	1
Methyl-directed repair DNA adenine methylase (EC 2.1.1.72)	1
RecA protein	1
Single-stranded DNA-binding protein	1
SOS-response repressor and protease LexA (EC 3.4.21.88)	1
Very-short-patch mismatch repair endonuclease (G-T specific)	1
DNA repair, bacterial DinG and relatives	3
ATP-dependent helicase DinG/Rad3	1
DinG family ATP-dependent helicase CPE1197	1
DinG family ATP-dependent helicase YoaA	1
DNA repair, bacterial MutL-MutS system	4

DNA mismatch repair protein MutL	1
DNA mismatch repair protein MutS	1
MutS-related protein, family 1	1
Recombination inhibitory protein MutS2	1
DNA repair, bacterial photolyase	3
Cryptochrome	1
Deoxyribodipyrimidine photolyase (EC 4.1.99.3)	1
Deoxyribodipyrimidine photolyase, single-strand-specific	1
DNA repair, bacterial RecBCD pathway	4
Exodeoxyribonuclease V beta chain (EC 3.1.11.5)	1
Exodeoxyribonuclease V gamma chain (EC 3.1.11.5)	1
Protease III precursor (EC 3.4.24.55)	1
RecD-like DNA helicase YrrC	1
DNA repair, bacterial RecFOR pathway	6
DNA recombination and repair protein RecF	1
DNA recombination and repair protein RecO	1
RecA protein	1
Recombination protein RecR	1
Single-stranded DNA-binding protein	1
Single-stranded-DNA-specific exonuclease RecJ (EC 3.1.-.-)	1
DNA repair, bacterial UmuCD system	4
Error-prone repair protein UmuD	1
Error-prone, lesion bypass DNA polymerase V (UmuC)	1
RecA protein	1
SOS-response repressor and protease LexA (EC 3.4.21.88)	1
DNA repair, bacterial UvrD and related helicases	6
ATP-dependent DNA helicase pcrA (EC 3.6.1.-)	1
ATP-dependent DNA helicase Rep	1
ATP-dependent DNA helicase UvrD/PcrA	1
ATP-dependent DNA helicase UvrD/PcrA, actinomycete paralog	1
ATP-dependent DNA helicase UvrD/PcrA, proteobacterial paralog	1
DNA helicase IV	1
DNA repair, UvrABC system	4
Excinuclease ABC subunit A	1
Excinuclease ABC subunit A paralog of unknown function	1
Excinuclease ABC subunit B	1
Excinuclease ABC subunit C	1

Nonhomologous End-Joining in Bacteria	3
ATP-dependent DNA ligase (EC 6.5.1.1) clustered with Ku protein, LigD	1
ATP-dependent DNA ligase (EC 6.5.1.1) LigC	1
Ku domain protein	1
RecA and RecX	2
RecA protein	1
Regulatory protein RecX	1
Uracil-DNA glycosylase	4
G:T/U mismatch-specific uracil/thymine DNA-glycosylase	1
Uracil-DNA glycosylase, family 1	1
Uracil-DNA glycosylase, family 4	1
Uracil-DNA glycosylase, putative family 6	1
**DNA replication**	**37**
DNA replication, archaeal	3
Archaeal DNA polymerase I (EC 2.7.7.7)	1
ATP-dependent DNA ligase (EC 6.5.1.1)	1
Replication factor C small subunit	1
DNA topoisomerases, Type I, ATP-independent	3
DNA topoisomerase I (EC 5.99.1.2)	1
DNA topoisomerase III (EC 5.99.1.2)	1
Similar to C-terminal Zn-finger domain of DNA topoisomerase I	1
DNA topoisomerases, Type II, ATP-dependent	5
DNA gyrase subunit A (EC 5.99.1.3)	1
DNA gyrase subunit B (EC 5.99.1.3)	1
DNA topoisomerase II (EC 5.99.1.3)	1
DNA topoisomerase VI subunit B (EC 5.99.1.3)	1
Topoisomerase IV subunit A (EC 5.99.1.-)	1
DNA-replication	24
ATP-dependent DNA helicase RecG (EC 3.6.1.-)	1
Chromosomal replication initiator protein DnaA	1
Crossover junction endodeoxyribonuclease RuvC (EC 3.1.22.4)	1
DNA polymerase I (EC 2.7.7.7)	1
DNA polymerase III alpha subunit (EC 2.7.7.7)	1
DNA polymerase III beta subunit (EC 2.7.7.7)	1
DNA polymerase III chi subunit (EC 2.7.7.7)	1
DNA polymerase III delta subunit (EC 2.7.7.7)	1
DNA polymerase III epsilon subunit (EC 2.7.7.7)	1

DNA polymerase III subunits gamma and tau (EC 2.7.7.7)	1
DNA primase (EC 2.7.7.-)	1
DNA repair protein RecN	1
Exodeoxyribonuclease V beta chain (EC 3.1.11.5)	1
Exodeoxyribonuclease V gamma chain (EC 3.1.11.5)	1
Helicase PriA essential for oriC/DnaA-independent DNA replication	1
Holliday junction DNA helicase RuvA	1
Holliday junction DNA helicase RuvB	1
Primosomal replication protein N	1
Primosomal replication protein N prime prime	1
RecA protein	1
Recombination protein RecR	1
Replicative DNA helicase (EC 3.6.1.-)	1
Single-stranded-DNA-specific exonuclease RecJ (EC 3.1.-.-)	1
Transcription-repair coupling factor	1
Plasmid replication	2
Chromosome (plasmid) partitioning protein ParB	1
Plasmid replication protein RepA	1
**DNA structural proteins, bacterial**	**5**
DNA structural proteins, bacterial	5
Chromosome partition protein MukB	1
Chromosome partition protein MukE	1
DNA-binding protein HU-alpha	1
DNA-binding protein HU-beta	1
Integration host factor beta subunit	1
**DNA uptake, competence**	**17**
Competence in Streptococci	2
Competence-stimulating peptide ABC transporter permease protein ComB	1
Transcriptional regulator SpxA1	1
DNA processing cluster	3
DNA polymerase III subunits gamma and tau (EC 2.7.7.7)	1
DNA topoisomerase III (EC 5.99.1.2)	1
Recombination protein RecR	1
Gram Positive Competence	6
ComF operon protein A, DNA transporter ATPase	1
Competence protein CoiA	1
DNA-entry nuclease (Competence-specific nuclease) (EC 3.1.30.-)	1

Late competence protein ComEA, DNA receptor	1
Late competence protein ComEC, DNA transport	1
Late competence protein ComGA, access of DNA to ComEA	1
Late competence	4
ComF operon protein A, DNA transporter ATPase	1
Late competence protein ComEA, DNA receptor	1
Late competence protein ComEC, DNA transport	1
Late competence protein ComGA, access of DNA to ComEA	1
Natural DNA Transformation in Vibrio	2
Extracellular deoxyribonuclease Xds	1
Positive regulator of competence TfoX	1
**Nucleoid-associated proteins in Bacteria**	**1**
Nucleoid-associated proteins in Bacteria	1
DNA-binding protein H-NS	1
**Restriction-Modification System**	**7**
Restriction-Modification System	7
Putative DNA-binding protein in cluster with Type I restriction-modification system	1
Putative predicted metal-dependent hydrolase	1
Type I restriction-modification system, DNA-methyltransferase subunit M (EC 2.1.1.72)	1
Type I restriction-modification system, restriction subunit R (EC 3.1.21.3)	1
Type I restriction-modification system, specificity subunit S (EC 3.1.21.3)	1
Type III restriction-modification system DNA endonuclease res (EC 3.1.21.5)	1
Type III restriction-modification system methylation subunit (EC 2.1.1.72)	1
**Type I Restriction-Modification**	**3**
Type I Restriction-Modification	3
Type I restriction-modification system, DNA-methyltransferase subunit M (EC 2.1.1.72)	1
Type I restriction-modification system, restriction subunit R (EC 3.1.21.3)	1
Type I restriction-modification system, specificity subunit S (EC 3.1.21.3)	1
**YcfH**	**3**
YcfH	3
Putative deoxyribonuclease similar to YcfH, type 4	1
Putative deoxyribonuclease YcfH	1
Putative deoxyribonuclease YjjV	1
**Dormancy and Sporulation**	**31**
**Bsub-Spore-Coat**	**2**
Bsub-Spore-Coat	2
Manganese superoxide dismutase (EC 1.15.1.1)	1

Stage IV sporulation protein A	1
**Persister Cells**	**1**
Persister Cells	1
Ribosome modulation factor	1
**Spore Core Dehydration**	**1**
Spore Core Dehydration	1
Spore maturation protein B	1
**Spore germination**	**6**
Spore germination	6
Spore germination protein GerHA/GerIA	1
Spore germination protein GerKA	1
Spore germination protein GerKB	1
Spore germination protein GerKC	1
Spore germination protein GerLB	1
Spore germination protein GerQB	1
**Sporulation Cluster**	**3**
Sporulation Cluster	3
Spore cortex biosynthesis protein	1
Stage II sporulation serine phosphatase for sigma-F activation (SpoIIE)	1
Transcription-repair coupling factor	1
**Sporulation Cluster III A**	**4**
Sporulation Cluster III A	4
Stage III sporulation protein AE	1
Stage III sporulation protein AF	1
Stage III sporulation protein AG	1
Stage III sporulation protein AH	1
**Sporulation draft**	**1**
Sporulation draft	1
Sporulation kinase B homolog 2	1
**Sporulation gene orphans**	**10**
Sporulation gene orphans	10
Stage II sporulation protein related to metaloproteases (SpoIIQ)	1
Stage II sporulation serine phosphatase for sigma-F activation (SpoIIE)	1
Stage III sporulation protein AE	1
Stage III sporulation protein AF	1
Stage III sporulation protein AG	1
Stage III sporulation protein AH	1

Stage IV sporulation protein A	1
Stage V sporulation protein AD (SpoVAD)	1
Stage V sporulation protein AF (SpoVAF)	1
Stage V sporulation protein involved in spore cortex synthesis (SpoVR)	1
**Sporulation-associated proteins with broader functions**	**2**
Sporulation-associated proteins with broader functions	2
Peptidyl-tRNA hydrolase (EC 3.1.1.29)	1
Stage III sporulation protein AE	1
**SpoVS protein family**	**1**
SpoVS protein family	1
Stage III sporulation protein AE	1
**Fatty Acids, Lipids, and Isoprenoids**	**108**
**Fatty acids**	**37**
Acyl-CoA thioesterase II	3
Acyl-CoA thioesterase II (EC 3.1.2.-)	1
TesB-like acyl-CoA thioesterase 2	1
TesB-like acyl-CoA thioesterase 3	1
Fatty Acid Biosynthesis FASII	15
(3R)-hydroxymyristoyl-[acyl carrier protein] dehydratase (EC 4.2.1.-)	1
3-oxoacyl-[acyl-carrier protein] reductase (EC 1.1.1.100)	1
3-oxoacyl-[acyl-carrier-protein] synthase, KASI (EC 2.3.1.41)	1
3-oxoacyl-[acyl-carrier-protein] synthase, KASII (EC 2.3.1.41)	1
Acetyl-coenzyme A carboxyl transferase alpha chain (EC 6.4.1.2)	1
Acetyl-coenzyme A carboxyl transferase beta chain (EC 6.4.1.2)	1
Acyl carrier protein	1
Acyl carrier protein phosphodiesterase (EC 3.1.4.14)	1
Biotin carboxyl carrier protein of acetyl-CoA carboxylase	1
Biotin carboxylase of acetyl-CoA carboxylase (EC 6.3.4.14)	1
Enoyl-[acyl-carrier-protein] reductase [FMN] (EC 1.3.1.9)	1
Enoyl-[acyl-carrier-protein] reductase [NADH] (EC 1.3.1.9)	1
Enoyl-[acyl-carrier-protein] reductase [NADPH] (EC 1.3.1.10)	1
Holo-[acyl-carrier protein] synthase (EC 2.7.8.7)	1
Transcriptional regulator of fatty acid biosynthesis FabT	1
Fatty acid degradation regulons	6
2,4-dienoyl-CoA reductase [NADPH] (EC 1.3.1.34)	1
Enoyl-CoA hydratase (EC 4.2.1.17)	1
Long-chain fatty acid transport protein	1

Long-chain-fatty-acid--CoA ligase (EC 6.2.1.3)	1
Predicted transcriptional regulator for fatty acid degradation FadQ, TetR family	1
Transcriptional regulator for fatty acid degradation FadR, GntR family	1
Phospholipid and Fatty acid biosynthesis related cluster	5
3-oxoacyl-[ACP] synthase	1
3-oxoacyl-[ACP] synthase (EC 2.3.1.41) FabV like	1
FIG022199: FAD-binding protein	1
FIG027190: Putative transmembrane protein	1
FIG138576: 3-oxoacyl-[ACP] synthase (EC 2.3.1.41)	1
Polyunsaturated Fatty Acids synthesis	3
omega-3 polyunsaturated fatty acid synthase subunit, PfaA	1
omega-3 polyunsaturated fatty acid synthase subunit, PfaB	1
omega-3 polyunsaturated fatty acid synthase subunit, PfaC	1
Unsaturated Fatty Acid Metabolism	5
3-oxoacyl-[acyl-carrier-protein] synthase, KASI (EC 2.3.1.41)	1
3-oxoacyl-[acyl-carrier-protein] synthase, KASII (EC 2.3.1.41)	1
Fatty acid desaturase (EC 1.14.19.3)	1
Long-chain-fatty-acid--CoA ligase (EC 6.2.1.3)	1
Unsaturated fatty acid biosythesis repressor FabR, TetR family	1
**Isoprenoids**	**45**
Acyclic terpenes utilization	1
Geranyl-CoA carboxylase biotin-containing subunit	1
Archaeal lipids	6
(S)-2,3-di-O-geranylgeranylglyceryl phosphate synthase	1
Diphosphomevalonate decarboxylase (EC 4.1.1.33)	1
Geranyltranstransferase (farnesyldiphosphate synthase) (EC 2.5.1.10)	1
Glycerol-1-phosphate dehydrogenase [NAD(P)] (EC 1.1.1.261)	1
Hydroxymethylglutaryl-CoA synthase (EC 2.3.3.10)	1
Isopentenyl-diphosphate delta-isomerase (EC 5.3.3.2)	1
Carotenoids	10
2-vinyl bacteriochlorophyllide hydratase BchF (EC 4.2.1.-)	1
Beta-carotene hydroxylase	1
CrtV-methyltransferase-like protein	1
Geranyltranstransferase (farnesyldiphosphate synthase) (EC 2.5.1.10)	1
Methoxyneurosporene dehydrogenase (EC 1.14.99.-)	1
Phytoene dehydrogenase and related proteins	1
Phytoene desaturase, neurosporene or lycopene producing (EC 1.3.-.-)	1

Phytoene desaturase, pro-zeta-carotene producing (EC 1.-.-.-)	1
Phytoene synthase (EC 2.5.1.32)	1
Pro-zeta-carotene desaturase, prolycopene producing (EC 1.-.-.-)	1
Isoprenoid Biosynthesis	10
1-deoxy-D-xylulose 5-phosphate reductoisomerase (EC 1.1.1.267)	1
1-deoxy-D-xylulose 5-phosphate synthase (EC 2.2.1.7)	1
2-C-methyl-D-erythritol 4-phosphate cytidylyltransferase (EC 2.7.7.60)	1
4-diphosphocytidyl-2-C-methyl-D-erythritol kinase (EC 2.7.1.148)	1
4-hydroxy-3-methylbut-2-enyl diphosphate reductase (EC 1.17.1.2)	1
Diphosphomevalonate decarboxylase (EC 4.1.1.33)	1
Geranyltranstransferase (farnesyldiphosphate synthase) (EC 2.5.1.10)	1
Hydroxymethylglutaryl-CoA synthase (EC 2.3.3.10)	1
Isopentenyl-diphosphate delta-isomerase (EC 5.3.3.2)	1
Undecaprenyl pyrophosphate synthetase (EC 2.5.1.31)	1
Isoprenoid Biosynthesis: Interconversions	2
Geranyltranstransferase (farnesyldiphosphate synthase) (EC 2.5.1.10)	1
Isopentenyl-diphosphate delta-isomerase (EC 5.3.3.2)	1
Mevalonate Branch of Isoprenoid Biosynthesis	2
Diphosphomevalonate decarboxylase (EC 4.1.1.33)	1
Hydroxymethylglutaryl-CoA synthase (EC 2.3.3.10)	1
Myxoxanthophyll biosynthesis in Cyanobacteria	1
Beta-carotene hydroxylase	1
Nonmevalonate Branch of Isoprenoid Biosynthesis	5
1-deoxy-D-xylulose 5-phosphate reductoisomerase (EC 1.1.1.267)	1
1-deoxy-D-xylulose 5-phosphate synthase (EC 2.2.1.7)	1
2-C-methyl-D-erythritol 4-phosphate cytidylyltransferase (EC 2.7.7.60)	1
4-diphosphocytidyl-2-C-methyl-D-erythritol kinase (EC 2.7.1.148)	1
4-hydroxy-3-methylbut-2-enyl diphosphate reductase (EC 1.17.1.2)	1
Polyprenyl Diphosphate Biosynthesis	3
Geranyltranstransferase (farnesyldiphosphate synthase) (EC 2.5.1.10)	1
Solanesyl diphosphate synthase (EC 2.5.1.11)	1
Undecaprenyl pyrophosphate synthetase (EC 2.5.1.31)	1
polyprenyl synthesis	5
1-deoxy-D-xylulose 5-phosphate reductoisomerase (EC 1.1.1.267)	1
2-C-methyl-D-erythritol 4-phosphate cytidylyltransferase (EC 2.7.7.60)	1
4-diphosphocytidyl-2-C-methyl-D-erythritol kinase (EC 2.7.1.148)	1
4-hydroxy-3-methylbut-2-enyl diphosphate reductase (EC 1.17.1.2)	1

Isopentenyl-diphosphate delta-isomerase (EC 5.3.3.2)	1
**Phospholipids**	**18**
Glycerolipid and Glycerophospholipid Metabolism in Bacteria	18
1-acyl-sn-glycerol-3-phosphate acyltransferase (EC 2.3.1.51)	1
Acyl carrier protein	1
Acyl-phosphate:glycerol-3-phosphate O-acyltransferase PlsY	1
Aerobic glycerol-3-phosphate dehydrogenase (EC 1.1.5.3)	1
Alcohol dehydrogenase (EC 1.1.1.1)	1
Aldehyde dehydrogenase B (EC 1.2.1.22)	1
Anaerobic glycerol-3-phosphate dehydrogenase subunit A (EC 1.1.5.3)	1
Anaerobic glycerol-3-phosphate dehydrogenase subunit B (EC 1.1.5.3)	1
CDP-diacylglycerol--glycerol-3-phosphate 3-phosphatidyltransferase (EC 2.7.8.5)	1
CDP-diacylglycerol--serine O-phosphatidyltransferase (EC 2.7.8.8)	1
Glycerate kinase (EC 2.7.1.31)	1
Glycerol kinase (EC 2.7.1.30)	1
Glycerol-1-phosphate dehydrogenase [NAD(P)] (EC 1.1.1.261)	1
Glycerol-3-phosphate dehydrogenase (EC 1.1.5.3)	1
Phosphate:acyl-ACP acyltransferase PlsX	1
Phosphatidate cytidylyltransferase (EC 2.7.7.41)	1
Phosphatidylglycerophosphatase A (EC 3.1.3.27)	1
Phosphatidylserine decarboxylase (EC 4.1.1.65)	1
**Polyhydroxybutyrate metabolism**	**8**
Polyhydroxybutyrate metabolism	8
3-hydroxyacyl-CoA dehydrogenase (EC 1.1.1.35)	1
Acetoacetyl-CoA reductase (EC 1.1.1.36)	1
Acetoacetyl-CoA synthetase (EC 6.2.1.16)	1
D(-)-3-hydroxybutyrate oligomer hydrolase (EC 3.1.1.22)	1
D-beta-hydroxybutyrate dehydrogenase (EC 1.1.1.30)	1
D-beta-hydroxybutyrate permease	1
Enoyl-CoA hydratase (EC 4.2.1.17)	1
Polyhydroxyalkanoic acid synthase	1
**Iron acquisition and metabolism**	**106**
**ABC transporter [iron.B12.siderophore.hemin]**	**2**
ABC transporter [iron.B12.siderophore.hemin]	2
ABC transporter (iron.B12.siderophore.hemin) , permease component	1
Outer membrane (iron.B12.siderophore.hemin) receptor	1
**Campylobacter Iron Metabolism**	**5**

Campylobacter Iron Metabolism	5
Ferric iron ABC transporter, iron-binding protein	1
Ferric iron ABC transporter, permease protein	1
Ferric siderophore transport system, periplasmic binding protein TonB	1
Ferrous iron transport protein B	1
Haemin uptake system outer membrane receptor	1
**Ferrous iron transporter EfeUOB, low-pH-induced**	**1**
Ferrous iron transporter EfeUOB, low-pH-induced	1
Ferrous iron transport peroxidase EfeB	1
**Heme, hemin uptake and utilization systems in GramPositives**	**12**
Heme, hemin uptake and utilization systems in GramPositives	12
Heme ABC type transporter HtsABC, permease protein HtsC	1
Heme oxygenase HemO, associated with heme uptake	1
Heme transporter IsdDEF, permease component IsdF	1
Hemin transport protein HmuS	1
Hypothetical protein DUF454	1
Iron compound ABC uptake transporter permease protein	1
NPQTN specific sortase B	1
Sensor histidine kinase colocalized with HrtAB transporter	1
Sortase A, LPXTG specific	1
Two-component response regulator colocalized with HrtAB transporter	1
Two-component response regulator SA14-24	1
Uncharacterized iron compound ABC uptake transporter, substrate-binding protein	1
**Hemin transport system**	**12**
Hemin transport system	12
ABC-type hemin transport system, ATPase component	1
Biopolymer transport protein ExbD1	1
Ferric siderophore transport system, periplasmic binding protein TonB	1
Haemin uptake system outer membrane receptor	1
Heme oxygenase HemO, associated with heme uptake	1
Hemin ABC transporter, permease protein	1
Hemin transport protein HmuS	1
Hemin uptake protein	1
Outer membrane receptor proteins, mostly Fe transport	1
Periplasmic hemin-binding protein	1
Pyridoxamine 5'-phosphate oxidase-related putative heme iron utilization protein	1
TonB-dependent hemin , ferrichrome receptor	1

**Iron acquisition in Streptococcus**	**4**
Iron acquisition in Streptococcus	4
Ferric iron ABC transporter, ATP-binding protein	1
Ferric iron ABC transporter, iron-binding protein	1
Ferric iron ABC transporter, permease protein	1
Iron compound ABC uptake transporter permease protein	1
**Iron acquisition in Vibrio**	**37**
Iron acquisition in Vibrio	37
2,3-dihydro-2,3-dihydroxybenzoate dehydrogenase (EC 1.3.1.28)	1
2,3-dihydroxybenzoate-AMP ligase (EC 2.7.7.58)	1
Amide synthase component of siderophore synthetase	1
Bacterioferritin	1
Bacterioferritin-associated ferredoxin	1
Enterobactin receptor VctA	1
Ferric hydroxamate ABC transporter (TC 3.A.1.14.3), ATP-binding protein FhuC	1
Ferric hydroxamate ABC transporter (TC 3.A.1.14.3), permease component FhuB	1
Ferric iron ABC transporter, ATP-binding protein	1
Ferric iron ABC transporter, iron-binding protein	1
Ferric iron ABC transporter, permease protein	1
Ferric uptake regulation protein FUR	1
Ferric vibriobactin, enterobactin transport system, ATP-binding protein (TC 3.A.1.14.6)	1
Ferric vibriobactin, enterobactin transport system, ATP-binding protein ViuC (TC 3.A.1.14.6)	1
Ferric vibriobactin, enterobactin transport system, permease protein VctD (TC 3.A.1.14.6)	1
Ferric vibriobactin, enterobactin transport system, permease protein ViuD (TC 3.A.1.14.6)	1
Ferric vibriobactin, enterobactin transport system, permease protein ViuG (TC 3.A.1.14.6)	1
Ferric vibriobactin, enterobactin transport system, substrate-binding protein VctP (TC 3.A.1.14.6)	1
Ferric vibriobactin, enterobactin transport system, substrate-binding protein ViuP (TC 3.A.1.14.6)	1
Ferric vulnibactin receptor VuuA	1
Ferrichrome transport system permease protein FhuB (TC 3.A.1.14.3)	1
Ferrichrome-binding periplasmic protein precursor (TC 3.A.1.14.3)	1
Ferrous iron transport protein B	1
Ferrous iron transport protein C	1
Hemin ABC transporter, permease protein	1
Hypothetical protein colocalized with Enterobactin receptor VctA	1
Iron-regulated virulence regulatory protein irgB	1
Isochorismate synthase (EC 5.4.4.2) of siderophore biosynthesis	1
Non-ribosomal peptide synthetase modules, siderophore biosynthesis	1

Phosphopantetheinyl transferase component of siderophore synthetase (EC 2.7.8.-)	1
Putative heme iron utilization protein	1
Putative TonB-dependent heme receptor HasR	1
Pyridoxamine 5'-phosphate oxidase-related putative heme iron utilization protein	1
TonB-dependent heme receptor HutR	1
TonB-dependent receptor	1
Transcriptional regulator near Vibriobactin biosynthetic gene custer	1
Transcriptional regulator, VCA0231 ortholog	1
**Iron Scavenging cluster in Thermus**	**2**
Iron Scavenging cluster in Thermus	2
Ferrichrome-binding periplasmic protein precursor (TC 3.A.1.14.3)	1
Probable thiol oxidoreductase with 2 cytochrome c heme-binding sites	1
**Siderophores**	**24**
Siderophore Achromobactin	2
Putative siderophore biosynthesis protein, related to 2-demethylmenaquinone methyltransferase	1
Siderophore achromobactin ABC transporter, substrate-binding protein	1
Siderophore Aerobactin	2
Ferric hydroxamate ABC transporter (TC 3.A.1.14.3), ATP-binding protein FhuC	1
Ferric hydroxamate ABC transporter (TC 3.A.1.14.3), permease component FhuB	1
Siderophore assembly kit	11
ABC-type Fe3+-siderophore transport system, permease component	1
ABC-type hemin transport system, ATPase component	1
Ferric hydroxamate ABC transporter (TC 3.A.1.14.3), ATP-binding protein FhuC	1
Ferric hydroxamate ABC transporter (TC 3.A.1.14.3), permease component FhuB	1
Ferric reductase (1.6.99.14)	1
Hemin ABC transporter, permease protein	1
Hemin transport protein HmuS	1
Isochorismate synthase (EC 5.4.4.2) of siderophore biosynthesis	1
Periplasmic hemin-binding protein	1
TonB-dependent hemin , ferrichrome receptor	1
TonB-dependent siderophore receptor	1
Siderophore Enterobactin	4
Enterobactin esterase	1
Enterobactin synthetase component F, serine activating enzyme (EC 2.7.7.-)	1
Ferric enterobactin transport ATP-binding protein FepC (TC 3.A.1.14.2)	1
Ferric enterobactin-binding periplasmic protein FepB (TC 3.A.1.14.2)	1
Siderophore Pyoverdine	3

Non-ribosomal peptide synthetase modules, pyoverdine	1
Non-ribosomal peptide synthetase modules, pyoverdine??	1
Putative dipeptidase, pyoverdin biosynthesis PvdM	1
Siderophore Yersiniabactin Biosynthesis	1
iron aquisition regulator (YbtA,AraC-like,required for transcription of FyuA/psn,Irp2)	1
Vibrioferrin synthesis	1
Vibrioferrin decarboxylase protein PvsE	1
**Transport of Iron**	**7**
Transport of Iron	7
Ferric iron ABC transporter, ATP-binding protein	1
Ferric iron ABC transporter, iron-binding protein	1
Ferric iron ABC transporter, permease protein	1
Ferric uptake regulation protein FUR	1
Ferrous iron transport protein B	1
Iron-regulated protein A precursor	1
Iron-uptake factor PiuC	1
**Membrane Transport**	**232**
**ABC transporters**	**26**
ABC transporter alkylphosphonate (TC 3.A.1.9.1)	3
Phosphonate ABC transporter ATP-binding protein (TC 3.A.1.9.1)	1
Phosphonate ABC transporter permease protein phnE (TC 3.A.1.9.1)	1
Phosphonate ABC transporter permease protein phnE2 (TC 3.A.1.9.1)	1
ABC transporter branched-chain amino acid (TC 3.A.1.4.1)	2
Branched-chain amino acid transport ATP-binding protein LivG (TC 3.A.1.4.1)	1
Branched-chain amino acid transport system permease protein LivM (TC 3.A.1.4.1)	1
ABC transporter dipeptide (TC 3.A.1.5.2)	5
Dipeptide transport ATP-binding protein DppD (TC 3.A.1.5.2)	1
Dipeptide transport ATP-binding protein DppF (TC 3.A.1.5.2)	1
Dipeptide transport system permease protein DppB (TC 3.A.1.5.2)	1
Dipeptide transport system permease protein DppC (TC 3.A.1.5.2)	1
Dipeptide-binding ABC transporter, periplasmic substrate-binding component (TC 3.A.1.5.2)	1
ABC transporter oligopeptide (TC 3.A.1.5.1)	2
Oligopeptide transport ATP-binding protein OppD (TC 3.A.1.5.1)	1
Oligopeptide transport system permease protein OppB (TC 3.A.1.5.1)	1
ABC transporter peptide (TC 3.A.1.5.5)	4
Peptide transport periplasmic protein sapA (TC 3.A.1.5.5)	1
Peptide transport system ATP-binding protein sapF (TC 3.A.1.5.5)	1

Peptide transport system permease protein sapB (TC 3.A.1.5.5)	1
Peptide transport system permease protein sapC (TC 3.A.1.5.5)	1
ABC transporter tungstate (TC 3.A.1.6.2)	3
ABC-type tungstate transport system, ATP-binding protein	1
ABC-type tungstate transport system, periplasmic binding protein	1
ABC-type tungstate transport system, permease protein	1
ATP-dependent efflux pump transporter Ybh	3
ABC transport system, permease component YbhR	1
ABC transporter multidrug efflux pump, fused ATP-binding domains	1
Transcriptional regulator YbiH, TetR family	1
AttEFGH ABC Transport System	2
AttE component of AttEFGH ABC transport system	1
AttH component of AttEFGH ABC transport system	1
Periplasmic-Binding-Protein-Dependent Transport System for α-Glucosides	2
Alpha-glucosides-binding periplasmic protein AglE precursor	1
Transcriptional regulator AglR, LacI family	1
**Choline Transport**	**1**
Choline Transport	1
Sodium-Choline Symporter	1
**ECF class transporters**	**20**
ECF class transporters	20
ATPase component CbiO of energizing module of cobalt ECF transporter	1
ATPase component of general energizing module of ECF transporters	1
ATPase component STY3232 of energizing module of queuosine-regulated ECF transporter	1
Duplicated ATPase component BL0693 of energizing module of predicted ECF transporter	1
Duplicated ATPase component MtsB of energizing module of methionine-regulated ECF transporter	1
Duplicated ATPase component YkoD of energizing module of thiamin-regulated ECF transporter for HydroxyMethylPyrimidine	1
Substrate-specific component BioY of biotin ECF transporter	1
Substrate-specific component PanT of predicted pantothenate ECF transporter	1
Substrate-specific component QueT (COG4708) of predicted queuosine-regulated ECF transporter	1
Substrate-specific component STY3230 of queuosine-regulated ECF transporter	1
Substrate-specific component ThiT of thiamin ECF transporter	1
Substrate-specific component ThiW of predicted thiazole ECF transporter	1
Transmembrane component BioN of energizing module of biotin ECF transporter	1
Transmembrane component BL0694 of energizing module of predicted ECF transporter	1
Transmembrane component CbiQ of energizing module of cobalt ECF transporter	1
Transmembrane component CbrV of energizing module of predicted cobalamin ECF transporter	1

Transmembrane component NikQ of energizing module of nickel ECF transporter	1
Transmembrane component of general energizing module of ECF transporters	1
Transmembrane component STY3231 of energizing module of queuosine-regulated ECF transporter	1
Transmembrane component YkoC of energizing module of thiamin-regulated ECF transporter for HydroxyMethylPyrimidine	1
**Folate transporters**	**6**
Folate transporters	6
5-formyltetrahydrofolate cyclo-ligase (EC 6.3.3.2)	1
ATPase component of general energizing module of ECF transporters	1
Dihydrofolate synthase (EC 6.3.2.12) / Folylpolyglutamate synthase (EC 6.3.2.17)	1
Folate transporter 3	1
Formyltetrahydrofolate deformylase (EC 3.5.1.10)	1
Transmembrane component of general energizing module of ECF transporters	1
**Phosphoglycerate transport system**	**2**
Phosphoglycerate transport system	2
Phosphoglycerate transport system sensor protein PgtB (EC 2.7.3.-)	1
Phosphoglycerate transporter protein PgtP	1
**Protein and nucleoprotein secretion system, Type IV**	**48**
Dot-Icm type IV secretion system	1
DotA protein	1
Mannose-sensitive hemagglutinin type 4 pilus	12
MSHA biogenesis protein MshG	1
MSHA biogenesis protein MshH	1
MSHA biogenesis protein MshI	1
MSHA biogenesis protein MshJ	1
MSHA biogenesis protein MshK	1
MSHA biogenesis protein MshL	1
MSHA biogenesis protein MshN	1
MSHA biogenesis protein MshO	1
MSHA biogenesis protein MshP	1
MSHA biogenesis protein MshQ	1
MSHA pilin protein MshC	1
MSHA pilin protein MshD	1
pVir Plasmid of Campylobacter	3
ATPase provides energy for both assembly of type IV secretion complex and secretion of T-DNA complex (VirB4)	1
DNA topoisomerase I (EC 5.99.1.2)	1
Single-stranded DNA-binding protein	1
Toxin co-regulated pilus	12

TCP pilin signal peptidase, TcpA processing	1
Toxin co-regulated pilin A	1
Toxin co-regulated pilus biosynthesis protein C, outer membrane protein	1
Toxin co-regulated pilus biosynthesis protein D	1
Toxin co-regulated pilus biosynthesis protein E, anchors TcpT to membrane	1
Toxin co-regulated pilus biosynthesis protein F, putative outer membrane channel for TcpA extrusion	1
Toxin co-regulated pilus biosynthesis protein H, transcriptional activator of ToxT promoter	1
Toxin co-regulated pilus biosynthesis protein I, chemoreceptor, negative regulator of TcpA	1
Toxin co-regulated pilus biosynthesis protein P, transcriptional activator of ToxT promoter	1
Toxin co-regulated pilus biosynthesis protein Q	1
Toxin co-regulated pilus biosynthesis protein R	1
Toxin co-regulated pilus biosynthesis protein T, putative ATP-binding translocase of TcpA	1
Type 4 conjugative transfer system, IncI1 type	3
IncI1 plasmid conjugative transfer protein TraF	1
IncI1 plasmid conjugative transfer protein TraU	1
Shufflon-specific DNA recombinase	1
Type IV pilus	17
3-dehydroquinate synthase (EC 4.2.3.4)	1
Multimodular transpeptidase-transglycosylase (EC 2.4.1.129) (EC 3.4.-.-)	1
Twitching motility protein PilT	1
Two-component sensor PilS	1
Type IV fimbrial assembly, ATPase PilB	1
Type IV fimbrial biogenesis protein FimT	1
Type IV fimbrial biogenesis protein PilV	1
Type IV fimbrial biogenesis protein PilW	1
Type IV fimbrial biogenesis protein PilX	1
Type IV fimbrial biogenesis protein PilY1	1
Type IV pilin PilA	1
Type IV pilus biogenesis protein PilE	1
Type IV pilus biogenesis protein PilM	1
Type IV pilus biogenesis protein PilN	1
Type IV pilus biogenesis protein PilO	1
Type IV pilus biogenesis protein PilP	1
Type IV pilus biogenesis protein PilQ	1
**Protein secretion system, Type II**	**24**
CBSS-562.2.peg.633	5
Dephospho-CoA kinase (EC 2.7.1.24)	1

FIG002842: hypothetical protein	1
FIG003276: zinc-binding protein	1
Mutator mutT protein (7,8-dihydro-8-oxoguanine-triphosphatase) (EC 3.6.1.-)	1
Type IV fimbrial assembly, ATPase PilB	1
General Secretion Pathway	12
General secretion pathway protein B	1
General secretion pathway protein C	1
General secretion pathway protein D	1
General secretion pathway protein E	1
General secretion pathway protein F	1
General secretion pathway protein G	1
General secretion pathway protein I	1
General secretion pathway protein J	1
General secretion pathway protein K	1
General secretion pathway protein L	1
General secretion pathway protein M	1
General secretion pathway protein N	1
Widespread colonization island	7
Flp pilus assembly protein TadD, contains TPR repeat	1
Flp pilus assembly protein, pilin Flp	1
Flp pilus assembly surface protein TadF, ATP/GTP-binding motif	1
Type II/IV secretion system ATP hydrolase TadA/VirB11/CpaF, TadA subfamily	1
Type II/IV secretion system protein TadC, associated with Flp pilus assembly	1
Type II/IV secretion system secretin RcpA/CpaC, associated with Flp pilus assembly	1
Type IV prepilin peptidase TadV/CpaA	1
**Protein secretion system, Type III**	**7**
Type III secretion system	5
Type III secretion bridge between inner and outermembrane lipoprotein (YscJ,HrcJ,EscJ, PscJ)	1
Type III secretion cytoplasmic protein (YscL)	1
Type III secretion inner membrane protein (YscU,SpaS,EscU,HrcU,SsaU, homologous to flagellar export components)	1
Type III secretion low calcium response protein (LcrR)	1
Type III secretion outermembrane pore forming protein (YscC,MxiD,HrcC, InvG)	1
Type III secretion system orphans	2
BarA sensory histidine kinase (= VarS = GacS)	1
BarA-associated response regulator UvrY (= GacA = SirA)	1
**Protein secretion system, Type VI**	**18**
Type VI secretion systems	18

IcmF-related protein	1
Outer membrane protein ImpK/VasF, OmpA/MotB domain	1
Protein ImpG/VasA	1
Sigma-54 dependent transcriptional regulator	1
Type VI secretion lipoprotein/VasD	1
Type VI secretion protein VasI	1
Type VI secretion-related protein VasL	1
Uncharacterized protein ImpA	1
Uncharacterized protein ImpB	1
Uncharacterized protein ImpC	1
Uncharacterized protein ImpD	1
Uncharacterized protein ImpF	1
Uncharacterized protein ImpH/VasB	1
Uncharacterized protein ImpI/VasC	1
Uncharacterized protein ImpJ/VasE	1
Uncharacterized protein similar to VCA0109	1
VgrG protein	1
VgrG-3 protein	1
**Protein secretion system, Type VII (Chaperone/Usher pathway, CU)**	**1**
The fimbrial Stf cluster	1
Uncharacterized protein YadU in stf fimbrial cluster	1
**Protein secretion system, Type VIII (Extracellular nucleation/precipitation pathway, ENP)**	**2**
Curli production	2
Curli production assembly/transport component CsgG	1
Transcriptional regulator CsgD for 2nd curli operon	1
**Protein translocation across cytoplasmic membrane**	**16**
EcsAB transporter affecting expression and secretion of secretory preproteins	1
Foldase protein PrsA precursor (EC 5.2.1.8)	1
ESAT-6 proteins secretion system in Actinobacteria	1
RD1 region associated protein Rv3876	1
ESAT-6 proteins secretion system in Firmicutes	2
ESAT-6/Esx family secreted protein EsxA/YukE	1
Putative toxin component near putative ESAT-related proteins, repetitive	1
HtrA and Sec secretion	8
Preprotein translocase secY subunit (TC 3.A.5.1.1)	1
Preprotein translocase subunit SecE (TC 3.A.5.1.1)	1
Preprotein translocase subunit SecG (TC 3.A.5.1.1)	1

Protein export cytoplasm chaperone protein (SecB, maintains protein to be exported in unfolded state)	1
Protein export cytoplasm protein SecA ATPase RNA helicase (TC 3.A.5.1.1)	1
Protein-export membrane protein SecD (TC 3.A.5.1.1)	1
Protein-export membrane protein SecF (TC 3.A.5.1.1)	1
Serine protease, DegP/HtrA, do-like (EC 3.4.21.-)	1
SecY2-SecA2 Specialized Transport System	1
Accessory secretory protein Asp1	1
Twin-arginine translocation system	3
Twin-arginine translocation protein TatA	1
Twin-arginine translocation protein TatB	1
Twin-arginine translocation protein TatC	1
**Sugar Phosphotransferase Systems, PTS**	**10**
Fructose and Mannose Inducible PTS	2
fructose sensor histidine kinase	1
Phosphoenolpyruvate-protein phosphotransferase of PTS system (EC 2.7.3.9)	1
Galactose-inducible PTS	5
Phosphoenolpyruvate-protein phosphotransferase of PTS system (EC 2.7.3.9)	1
Phosphosugar-binding transcriptional repressor, RpiR family	1
Tagatose 1,6-bisphosphate aldolase (EC 4.1.2.40)	2
Tagatose-6-phosphate kinase (EC 2.7.1.144) / 1-phosphofructokinase (EC 2.7.1.56)	1
Sucrose-specific PTS	3
Phosphoenolpyruvate-protein phosphotransferase of PTS system (EC 2.7.3.9)	1
PTS system, sucrose-specific IIB component (EC 2.7.1.69) / PTS system, sucrose-specific IIC component (EC 2.7.1.69) / PTS system, sucrose-specific IIA component (EC 2.7.1.69)	1
Sucrose operon repressor ScrR, LacI family	1
**Ton and Tol transport systems**	**16**
Ton and Tol transport systems	16
4-hydroxybenzoyl-CoA thioesterase family active site	1
Biopolymer transport protein ExbD/TolR	1
Colicin I receptor precursor	1
Ferric siderophore transport system, periplasmic binding protein TonB	1
Haemin uptake system outer membrane receptor	1
iron-chelator utilization protein	1
Outer membrane receptor for ferric coprogen and ferric-rhodotorulic acid	1
Putative Ton-B dependent hemine receptor	1
Tol biopolymer transport system, TolR protein	1
TolA protein	1
tolB protein precursor, periplasmic protein involved in the tonb-independent uptake of group A colicins	1

TonB-dependent hemin , ferrichrome receptor	1
TonB-dependent receptor	1
TonB-dependent siderophore receptor	1
TPR domain protein, putative component of TonB system	1
Type I secretion outer membrane protein, TolC precursor	1
**Transport of Manganese**	**3**
Transport of Manganese	3
Manganese ABC transporter, inner membrane permease protein SitC	1
Mn-dependent transcriptional regulator MntR	1
Predicted manganese transporter, 11 TMS	1
**Transport of Molybdenum**	**4**
Transport of Molybdenum	4
Molybdate-binding domain of ModE	1
Molybdenum ABC transporter, periplasmic molybdenum-binding protein ModA (TC 3.A.1.8.1)	1
Molybdenum transport ATP-binding protein ModC (TC 3.A.1.8.1)	1
Molybdenum transport system permease protein ModB (TC 3.A.1.8.1)	1
**Transport of Nickel and Cobalt**	**8**
Transport of Nickel and Cobalt	8
Additional component NikL of nickel ECF transporter	1
ATPase component CbiO of energizing module of cobalt ECF transporter	1
HoxN/HupN/NixA family nickel/cobalt transporter	1
Nicel/Cobalt-specific TonB-dependent outer membrane receptor	1
Nickel transport system permease protein NikC (TC 3.A.1.5.3)	1
Nickel transporter UreH	1
Transmembrane component CbiQ of energizing module of cobalt ECF transporter	1
Transmembrane component NikQ of energizing module of nickel ECF transporter	1
**Transport of Zinc**	**5**
Transport of Zinc	5
Zinc ABC transporter, ATP-binding protein ZnuC	1
Zinc ABC transporter, inner membrane permease protein ZnuB	1
Zinc ABC transporter, periplasmic-binding protein ZnuA	1
Zinc uptake regulation protein ZUR	1
Zinc-regulated outer membrane receptor	1
**TRAP transporters**	**3**
A TRAP transporter and a hypothetical	1
TRAP transporter, 4TM/12TM fusion protein, unknown substrate 1	1
TRAP Transporter unknown substrate 9	2

TRAP transporter solute receptor, TAXI family precursor	1
TRAP-type uncharacterized transport system, fused permease component	1
**Tricarboxylate transport system**	**2**
Tricarboxylate transport system	2
Tricarboxylate transport membrane protein TctA	1
Tricarboxylate transport transcriptional regulator TctD	1
**Uni- Sym- and Antiporters**	**10**
Na(+) H(+) antiporter	1
Na+/H+ antiporter NhaD type	1
Proton-dependent Peptide Transporters	4
Di/tripeptide permease DtpA	1
Di/tripeptide permease DtpB	1
Di/tripeptide permease YjdL	1
Di-/tripeptide transporter	1
Sodium Hydrogen Antiporter	5
Na(+) H(+) antiporter subunit A	1
Na(+) H(+) antiporter subunit C	1
Na(+) H(+) antiporter subunit D (TC 2.A.63.1.3)	1
Na(+) H(+) antiporter subunit E	1
Na(+) H(+) antiporter subunit F	1
**Metabolism of Aromatic Compounds**	**62**
**Anaerobic degradation of aromatic compounds**	**2**
Acetophenone carboxylase 1	1
Acetophenone carboxylase subunit Apc3	1
Anaerobic toluene and ethylbenzene degradation	1
Acetophenone carboxylase subunit Apc3	1
**Aromatic Amin Catabolism**	**3**
Aromatic Amin Catabolism	3
4-hydroxyphenylacetate 3-monooxygenase (EC 1.14.13.3)	1
Aldehyde dehydrogenase (EC 1.2.1.3), PaaZ	1
Phenylacetaldehyde dehydrogenase (EC 1.2.1.39)	1
**Benzoate transport and degradation cluster**	**3**
Benzoate transport and degradation cluster	3
Methylglutaconyl-CoA hydratase (EC 4.2.1.18)	1
Regulatory protein of benzoate catabolism	1
Shikimate kinase I (EC 2.7.1.71)	1
**carbazol degradation cluster**	**1**

carbazol degradation cluster	1
2-hydroxy-6-oxo-6-phenylhexa-2,4-dienoate hydrolase (EC 3.7.1.-)	1
**Gentisare degradation**	**3**
Gentisare degradation	3
4-hydroxybenzoate transporter	1
Fumarylacetoacetate hydrolase family protein	1
putative 4-hydroxybenzoyl-CoA thioesterase	1
**Metabolism of central aromatic intermediates**	**24**
4-Hydroxyphenylacetic acid catabolic pathway	3
2,4-dihydroxyhept-2-ene-1,7-dioic acid aldolase (EC 4.1.2.-)	1
4-hydroxyphenylacetate 3-monooxygenase (EC 1.14.13.3)	1
5-carboxymethyl-2-hydroxymuconate delta-isomerase (EC 5.3.3.10)	1
Catechol branch of beta-ketoadipate pathway	4
3-oxoadipate CoA-transferase subunit A (EC 2.8.3.6)	1
3-oxoadipate CoA-transferase subunit B (EC 2.8.3.6)	1
Muconate cycloisomerase (EC 5.5.1.1)	1
Succinyl-CoA:3-ketoacid-coenzyme A transferase subunit B (EC 2.8.3.5)	1
Central meta-cleavage pathway of aromatic compound degradation	4
2-hydroxy-6-oxo-6-phenylhexa-2,4-dienoate hydrolase (EC 3.7.1.-)	1
2-polyprenylphenol hydroxylase and related flavodoxin oxidoreductases	1
5-carboxymethyl-2-hydroxymuconate delta-isomerase (EC 5.3.3.10)	1
Protocatechuate 4,5-dioxygenase beta chain (EC 1.13.11.8)	1
Homogentisate pathway of aromatic compound degradation	3
Aromatic-amino-acid aminotransferase (EC 2.6.1.57)	1
Fumarylacetoacetase (EC 3.7.1.2)	1
Transcriptional regulator, IclR family	1
Protocatechuate branch of beta-ketoadipate pathway	5
3-carboxy-cis,cis-muconate cycloisomerase (EC 5.5.1.2)	1
3-oxoadipate CoA-transferase subunit A (EC 2.8.3.6)	1
3-oxoadipate CoA-transferase subunit B (EC 2.8.3.6)	1
3-oxoadipate enol-lactone hydrolase/4-carboxymuconolactone decarboxylase	1
Succinyl-CoA:3-ketoacid-coenzyme A transferase subunit B (EC 2.8.3.5)	1
Salicylate and gentisate catabolism	5
4-hydroxybenzoate transporter	1
Fumarylacetoacetase (EC 3.7.1.2)	1
Fumarylacetoacetate hydrolase family protein	1
salicylate esterase	1

Salicylate hydroxylase (EC 1.14.13.1)	1
**Peripheral pathways for catabolism of aromatic compounds**	**21**
Benzoate catabolism	1
Muconate cycloisomerase (EC 5.5.1.1)	1
Benzoate degradation	3
benzoate MFS transporter BenK	1
Benzoate transport protein	1
Benzoylformate decarboxylase (EC 4.1.1.7)	1
Biphenyl Degradation	3
2-hydroxy-6-oxo-6-phenylhexa-2,4-dienoate hydrolase (EC 3.7.1.-)	1
2-keto-4-pentenoate hydratase (EC 4.2.1.-)	1
biphenyl-2,3-diol 1,2-dioxygenase III-related protein	1
Chloroaromatic degradation pathway	2
3-oxoadipate CoA-transferase subunit A (EC 2.8.3.6)	1
3-oxoadipate CoA-transferase subunit B (EC 2.8.3.6)	1
Chlorobenzoate degradation	1
Muconate cycloisomerase (EC 5.5.1.1)	1
n-Phenylalkanoic acid degradation	3
3-hydroxyacyl-CoA dehydrogenase (EC 1.1.1.35)	1
Enoyl-CoA hydratase (EC 4.2.1.17)	1
Long-chain-fatty-acid--CoA ligase (EC 6.2.1.3)	1
Phenol hydroxylase	1
Positive regulator of phenol hydroxylase	1
Phenylpropanoid compound degradation	2
4-hydroxybenzoate transporter	1
Protein involved in meta-pathway of phenol degradation	1
p-Hydroxybenzoate degradation	2
4-hydroxybenzoate transporter	1
P-hydroxybenzoate hydroxylase (EC 1.14.13.2)	1
Quinate degradation	1
3-dehydroquinate dehydratase II (EC 4.2.1.10)	1
Salicylate ester degradation	2
salicylate esterase	1
Salicylate hydroxylase (EC 1.14.13.1)	1
**Phenylacetyl-CoA catabolic pathway (core)**	**5**
Phenylacetyl-CoA catabolic pathway (core)	5
3-hydroxyacyl-CoA dehydrogenase PaaC (EC 1.1.1.-)	1

Phenylacetaldehyde dehydrogenase (EC 1.2.1.39)	1
Phenylacetate-CoA oxygenase, PaaJ subunit	1
Phenylacetate-coenzyme A ligase (EC 6.2.1.30) PaaF	1
Phenylacetic acid degradation protein PaaN2, ring-opening aldehyde dehydrogenase (EC 1.2.1.3)	1
**Miscellaneous**	**62**
**Archease**	**1**
Archease	1
Archease	1
**Archease2**	**1**
Archease2	1
Archease	1
**Broadly distributed proteins not in subsystems**	**4**
Broadly distributed proteins not in subsystems	4
UPF0265 protein YeeX	1
YbbM seven transmembrane helix protein	1
YciL protein	1
YrbA protein	1
**Carbonate Biomineralization**	**4**
Carbonate Biomineralization	4
Electron transfer flavoprotein, alpha subunit	1
Enoyl-CoA hydratase (EC 4.2.1.17)	1
Long-chain-fatty-acid--CoA ligase (EC 6.2.1.3)	1
Transcriptional regulator, TetR family	1
**Muconate lactonizing enzyme family**	**2**
Muconate lactonizing enzyme family	2
Muconate cycloisomerase (EC 5.5.1.1)	1
O-succinylbenzoate-CoA synthase (EC 4.2.1.-)	1
**Plant-Prokaryote DOE project**	**28**
At2g33980 At1g28960	3
FIG017823: ATPase, MoxR family	1
Hypothetical nudix hydrolase YeaB	1
tRNA nucleotidyltransferase (EC 2.7.7.21) (EC 2.7.7.25)	1
At3g21300	1
RNA methyltransferase, TrmA family	1
COG2363	8
Cysteine desulfurase (EC 2.8.1.7), IscS subfamily	1
Cysteine desulfurase (EC 2.8.1.7), SufS subfamily	1

Hydroxyethylthiazole kinase (EC 2.7.1.50)	1
Hydroxymethylpyrimidine ABC transporter, substrate-binding component	1
Thiaminase II (EC 3.5.99.2)	1
Thiamine biosynthesis protein thiI	1
Thiamin-phosphate pyrophosphorylase (EC 2.5.1.3)	1
ThiJ/PfpI family protein	1
Conserved gene cluster possibly involved in RNA metabolism	3
Cysteinyl-tRNA synthetase (EC 6.1.1.16)	1
Hypothetical protein DUF901, similar to C-terminal domain of ribosome protection-type Tc-resistance proteins	1
Serine acetyltransferase (EC 2.3.1.30)	1
Iojap	10
Adenylate cyclase (EC 4.6.1.1)	1
COG0536: GTP-binding protein Obg	1
Gamma-glutamyl phosphate reductase (EC 1.2.1.41)	1
Glutamate 5-kinase (EC 2.7.2.11)	1
Inorganic pyrophosphatase (EC 3.6.1.1)	1
LSU ribosomal protein L21p	1
Nicotinamidase (EC 3.5.1.19)	1
Nicotinate-nucleotide adenylyltransferase (EC 2.7.7.18)	1
Phosphatidate cytidylyltransferase (EC 2.7.7.41)	1
tRNA delta(2)-isopentenylpyrophosphate transferase (EC 2.5.1.8)	1
Synechocystis experimental	3
Esterase/lipase, sll0644 homolog	1
prolyl oligopeptidase family protein	1
Serine peptidase (Alpha/beta hydrolase superfamily) fused to N- terminal uncharacterized domain specific to cyanobacteria	1
**YaaA**	**1**
YaaA	1
UPF0246 protein YaaA	1
**YbbK**	**3**
YbbK	3
Putative activity regulator of membrane protease YbbK	1
Putative stomatin/prohibitin-family membrane protease subunit aq_911	1
Putative stomatin/prohibitin-family membrane protease subunit YbbK	1
**ZZ gjo need homes**	**18**
ZZ gjo need homes	18
Lipid A export ATP-binding/permease protein MsbA	1
Mitochondrial processing peptidase-like protein (EC 3.4.24.64)	1

Na+/H+ antiporter	1
Na+/H+ antiporter NhaB	1
Peptidyl-tRNA hydrolase, archaeal type (EC 3.1.1.29)	1
Phosphatidylcholine synthase (EC 2.7.8.24)	1
Pole remodelling regulatory diguanylate cyclase	1
Protein of unknown function DUF81	1
Protein of unknown function YceH	1
Putative heat shock protein YegD	1
Putative membrane protein YfcA	1
Putative permease often clustered with de novo purine synthesis	1
Pyrrolidone-carboxylate peptidase (EC 3.4.19.3)	1
Quinone oxidoreductase (EC 1.6.5.5)	1
Sodium/glutamate symport protein	1
Tyrosine-specific transport protein	1
Uncharacterized ATP-dependent helicase MJ0294	1
Virulence factor MviM	1
**Motility and Chemotaxis**	**85**
**Bacterial Chemotaxis**	**17**
Bacterial Chemotaxis	17
Aerotaxis sensor receptor protein	1
Chemotaxis protein CheC -- inhibitor of MCP methylation	1
Chemotaxis protein CheD	1
Chemotaxis protein CheV (EC 2.7.3.-)	1
Chemotaxis protein CheX	1
Chemotaxis response - phosphatase CheZ	1
Chemotaxis response regulator protein-glutamate methylesterase CheB (EC 3.1.1.61)	1
Dipeptide-binding ABC transporter, periplasmic substrate-binding component (TC 3.A.1.5.2)	1
Flagellar motor switch protein FliM	1
Flagellar motor switch protein FliN	1
Galactose/methyl galactoside ABC transport system, D-galactose-binding periplasmic protein MglB (TC 3.A.1.2.3)	1
Maltose/maltodextrin ABC transporter, substrate binding periplasmic protein MalE	1
Methyl-accepting chemotaxis protein I (serine chemoreceptor protein)	1
Methyl-accepting chemotaxis protein III (ribose and galactose chemoreceptor protein)	1
Methyl-accepting chemotaxis protein IV (dipeptide chemoreceptor protein)	1
Positive regulator of CheA protein activity (CheW)	1
Signal transduction histidine kinase CheA (EC 2.7.3.-)	1
**Flagellar motility in Prokaryota**	**47**

Additional flagellar genes in Vibrionales	2
Flagellar protein FlgO	1
Flagellar protein FlgP	1
Archaeal Flagellum	1
Flagella-related protein FlaI	1
Flagellar motility	12
Chemotaxis protein CheV (EC 2.7.3.-)	1
Flagellar biosynthesis protein FlhA	1
Flagellar biosynthesis protein FlhF	1
Flagellar L-ring protein FlgH	1
Flagellar motor rotation protein MotA	1
Flagellar motor rotation protein MotB	1
Flagellar motor switch protein FliM	1
Flagellar motor switch protein FliN	1
Flagellar synthesis regulator FleN	1
RNA polymerase sigma-54 factor RpoN	1
Rrf2 family transcriptional regulator	1
Signal transduction histidine kinase CheA (EC 2.7.3.-)	1
Flagellum	29
Flagellar basal-body rod protein FlgF	1
Flagellar basal-body rod protein FlgG	1
Flagellar biosynthesis protein FlhA	1
Flagellar biosynthesis protein FlhF	1
Flagellar biosynthesis protein FliC	1
Flagellar biosynthesis protein FliL	1
Flagellar biosynthesis protein FliP	1
Flagellar biosynthesis protein FliQ	1
Flagellar biosynthesis protein FliS	1
Flagellar hook-associated protein FlgK	1
Flagellar hook-associated protein FliD	1
Flagellar hook-basal body complex protein FliE	1
Flagellar L-ring protein FlgH	1
Flagellar motor rotation protein MotA	1
Flagellar motor rotation protein MotB	1
Flagellar motor switch protein FliM	1
Flagellar motor switch protein FliN	1
Flagellar M-ring protein FliF	1

Flagellar P-ring protein FlgI	1
Flagellar protein FlgJ [peptidoglycan hydrolase] (EC 3.2.1.-)	1
Flagellar protein FliJ	1
Flagellar regulatory protein FleQ	1
Flagellar synthesis regulator FleN	1
Flagellin protein FlaA	1
Flagellin protein FlaD	1
Flagellin protein FlaF	1
Flagellin protein FlaG	1
Negative regulator of flagellin synthesis FlgM	1
RNA polymerase sigma-54 factor RpoN	1
Flagellum in Campylobacter	3
Cell division protein FtsI [Peptidoglycan synthetase] (EC 2.4.1.129)	1
Flagellar hook-basal body complex protein FliE	1
Flagellin protein FlaA	1
**Social motility and nonflagellar swimming in bacteria**	**21**
Bacterial motility:Gliding	20
Acetylornithine deacetylase/Succinyl-diaminopimelate desuccinylase and related deacylases	1
Cell division protein FtsX	1
GldJ	1
gliding motility protein GldF	1
gliding motility protein GldG	1
twitching motility protein PilG	1
twitching motility protein PilH	1
twitching motility protein PilJ	1
Twitching motility protein PilT	1
Two-component sensor PilS	1
Type II secretory pathway, ATPase PulE/Tfp pilus assembly pathway, ATPase PilB	1
Type IV fimbrial assembly, ATPase PilB	1
Type IV fimbrial biogenesis protein FimT	1
Type IV fimbrial biogenesis protein PilV	1
Type IV fimbrial biogenesis protein PilW	1
Type IV fimbrial biogenesis protein PilX	1
Type IV fimbrial biogenesis protein PilY1	1
Type IV pilin PilA	1
Type IV pilus biogenesis protein PilE	1
type IV pilus biogenesis protein PilJ	1

Control of Swarming in Vibrio and Shewanella species	1
Sensory box/GGDEF family protein ScrC (involved in swarmer cell regulation)	1
**Nitrogen Metabolism**	**57**
**Allantoin Utilization**	**5**
Allantoin Utilization	5
Allantoate amidohydrolase (EC 3.5.3.9)	1
Allantoicase (EC 3.5.3.4)	1
Allantoinase (EC 3.5.2.5)	1
DNA-binding transcriptional activator of the allD operon	1
Glycerate kinase (EC 2.7.1.31)	1
**Ammonia assimilation**	**11**
Ammonia assimilation	11
[Protein-PII] uridylyltransferase (EC 2.7.7.59)	1
Ammonium transporter	1
Ferredoxin-dependent glutamate synthase (EC 1.4.7.1)	1
Glutamate synthase [NADPH] large chain (EC 1.4.1.13)	1
Glutamate-ammonia-ligase adenylyltransferase (EC 2.7.7.42)	1
Glutamine amidotransferase, class-II	1
Glutamine synthetase type III, GlnN (EC 6.3.1.2)	1
Glutamine synthetase, clostridia type (EC 6.3.1.2)	1
Nitrogen regulation protein NR(I)	1
Nitrogen regulation protein NR(II) (EC 2.7.3.-)	1
Nitrogen regulatory protein P-II	1
**Cyanate hydrolysis**	**1**
Cyanate hydrolysis	1
Carbonic anhydrase (EC 4.2.1.1)	1
**Denitrification**	**7**
Denitrification	7
Nitric oxide reductase activation protein NorD	1
Nitric oxide -responding transcriptional regulator Dnr (Crp/Fnr family)	1
Nitric oxide -responding transcriptional regulator NnrR (Crp/Fnr family)	1
Nitric-oxide reductase (EC 1.7.99.7), quinol-dependent	1
Nitrous oxide reductase maturation periplasmic protein NosX	1
Nitrous oxide reductase maturation protein NosR	1
Nitrous oxide reductase maturation transmembrane protein NosY	1
**Dissimilatory nitrite reductase**	**1**
Dissimilatory nitrite reductase	1

Uroporphyrinogen-III methyltransferase (EC 2.1.1.107)	1
**Nitrate and nitrite ammonification**	**17**
Nitrate and nitrite ammonification	17
Cytochrome c552 precursor (EC 1.7.2.2)	1
Cytochrome c-type heme lyase subunit nrfE, nitrite reductase complex assembly	1
Cytochrome c-type heme lyase subunit nrfG, nitrite reductase complex assembly	1
Cytochrome c-type protein NapC	1
Ferredoxin-type protein NapG (periplasmic nitrate reductase)	1
Nitrate reductase cytochrome c550-type subunit	1
Nitrate/nitrite response regulator protein	1
Nitrate/nitrite transporter	1
Nitrite reductase [NAD(P)H] large subunit (EC 1.7.1.4)	1
Nitrite transporter from formate/nitrite family	1
Periplasmic nitrate reductase component NapE	1
Periplasmic nitrate reductase component NapL	1
Periplasmic nitrate reductase precursor (EC 1.7.99.4)	1
Polyferredoxin NapH (periplasmic nitrate reductase)	1
Respiratory nitrate reductase alpha chain (EC 1.7.99.4)	1
Respiratory nitrate reductase beta chain (EC 1.7.99.4)	1
Respiratory nitrate reductase gamma chain (EC 1.7.99.4)	1
**Nitric oxide synthase**	**1**
Nitric oxide synthase	1
Manganese superoxide dismutase (EC 1.15.1.1)	1
**Nitrogen fixation**	**7**
Nitrogen fixation	7
AnfO protein, required for Mo- and V-independent nitrogenase	1
Iron-sulfur cluster assembly scaffold protein NifU	1
Nitrogenase (vanadium-iron) transcriptional regulator VnfA	1
Nitrogenase FeMo-cofactor carrier protein NifX	1
Nitrogenase FeMo-cofactor scaffold and assembly protein NifE	1
Nitrogenase FeMo-cofactor scaffold and assembly protein NifN	1
Nitrogenase vanadium-cofactor synthesis protein VnfE	1
**Nitrosative stress**	**7**
Nitrosative stress	7
Functional role page for Anaerobic nitric oxide reductase transcription regulator NorR	1
Hcp transcriptional regulator HcpR (Crp/Fnr family)	1
Hydroxylamine reductase (EC 1.7.-.-)	1

NADH oxidoreductase hcr (EC 1.-.-.-)	1
Nitric oxide-dependent regulator DnrN or NorA	1
Nitric-oxide reductase (EC 1.7.99.7), quinol-dependent	1
Nitrite-sensitive transcriptional repressor NsrR	1
**Nucleosides and Nucleotides**	**86**
**Adenosyl nucleosidases**	**2**
Adenosyl nucleosidases	2
hypothetical protein Bcep3774, commonly clustered with carotenoid biosynthesis	1
Purine nucleoside phosphorylase (EC 2.4.2.1)	1
**AMP to 3-phosphoglycerate**	**1**
AMP to 3-phosphoglycerate	1
AMP phosphohydrolase	1
**Hydantoin metabolism**	**5**
Hydantoin metabolism	5
Deacetylases, including yeast histone deacetylase and acetoin utilization protein	1
Hydantoinase	1
N-carbamoyl-L-amino acid hydrolase (EC 3.5.1.87)	1
N-methylhydantoinase A (EC 3.5.2.14)	1
N-methylhydantoinase B (EC 3.5.2.14)	1
**Pseudouridine Metabolism**	**3**
Pseudouridine Metabolism	3
Indigoidine synthase A-like protein, uncharacterized enzyme involved in pigment biosynthesis	1
Pseudouridine kinase (EC 2.7.1.83)	1
Ribosomal small subunit pseudouridine synthase A (EC 4.2.1.70)	1
**Purines**	**42**
A hypothetical coupled to de Novo Purine Biosynthesis	1
Phosphoribosylglycinamide formyltransferase (EC 2.1.2.2)	1
CBSS-314260.3.peg.2133	3
Phosphoribosylaminoimidazole-succinocarboxamide synthase (EC 6.3.2.6)	1
Phosphoribosylformylglycinamidine synthase, PurS subunit (EC 6.3.5.3)	1
Phosphoribosylformylglycinamidine synthase, synthetase subunit (EC 6.3.5.3)	1
De Novo Purine Biosynthesis	9
Phosphoribosylamine--glycine ligase (EC 6.3.4.13)	1
Phosphoribosylaminoimidazole carboxylase ATPase subunit (EC 4.1.1.21)	1
Phosphoribosylaminoimidazole carboxylase catalytic subunit (EC 4.1.1.21)	1
Phosphoribosylaminoimidazole-succinocarboxamide synthase (EC 6.3.2.6)	1
Phosphoribosylformylglycinamidine cyclo-ligase (EC 6.3.3.1)	1

Phosphoribosylformylglycinamidine synthase, PurS subunit (EC 6.3.5.3)	1
Phosphoribosylformylglycinamidine synthase, synthetase subunit (EC 6.3.5.3)	1
Phosphoribosylglycinamide formyltransferase (EC 2.1.2.2)	1
Phosphoribosylglycinamide formyltransferase 2 (EC 2.1.2.-)	1
Purine conversions	19
2',3'-cyclic-nucleotide 2'-phosphodiesterase (EC 3.1.4.16)	1
5'-nucleotidase (EC 3.1.3.5)	1
Adenine deaminase (EC 3.5.4.2)	1
Adenosine deaminase (EC 3.5.4.4)	1
Adenosine kinase (EC 2.7.1.20)	1
Adenylate kinase (EC 2.7.4.3)	1
Adenylosuccinate synthetase (EC 6.3.4.4)	1
Deoxyguanosinetriphosphate triphosphohydrolase (EC 3.1.5.1)	1
GMP reductase (EC 1.7.1.7)	1
GMP synthase [glutamine-hydrolyzing] (EC 6.3.5.2)	1
Guanine deaminase (EC 3.5.4.3)	1
Guanylate kinase (EC 2.7.4.8)	1
Hypoxanthine-guanine phosphoribosyltransferase (EC 2.4.2.8)	1
Inosine-5'-monophosphate dehydrogenase (EC 1.1.1.205)	1
Inosine-guanosine kinase (EC 2.7.1.73)	1
Inosine-uridine preferring nucleoside hydrolase (EC 3.2.2.1)	1
Polyphosphate kinase (EC 2.7.4.1)	1
Purine nucleoside phosphorylase (EC 2.4.2.1)	1
Xanthine-guanine phosphoribosyltransferase (EC 2.4.2.22)	1
Purine nucleotide synthesis regulator	1
Purine nucleotide synthesis repressor	1
Purine Utilization	8
Cytosine/purine/uracil/thiamine/allantoin permease family protein	1
Guanine deaminase (EC 3.5.4.3)	1
Periplasmic aromatic aldehyde oxidoreductase, FAD binding subunit YagS	1
Xanthine and CO dehydrogenases maturation factor, XdhC/CoxF family	1
Xanthine dehydrogenase, FAD binding subunit (EC 1.17.1.4)	1
Xanthine dehydrogenase, iron-sulfur cluster and FAD-binding subunit A (1.17.1.4)	1
Xanthine dehydrogenase, molybdenum binding subunit (EC 1.17.1.4)	1
XdhC protein (assists in molybdopterin insertion into xanthine dehydrogenase)	1
Xanthine Metabolism in Bacteria	1
Nucleoside permease NupC	1

**Pyrimidines**	**26**
De Novo Pyrimidine Synthesis	7
Aspartate carbamoyltransferase regulatory chain (PyrI)	1
Carbamoyl-phosphate synthase large chain (EC 6.3.5.5)	1
Carbamoyl-phosphate synthase small chain (EC 6.3.5.5)	1
Dihydroorotase (EC 3.5.2.3)	1
Dihydroorotate dehydrogenase (EC 1.3.3.1)	1
Dihydroorotate dehydrogenase, catalytic subunit (EC 1.3.3.1)	1
Uracil phosphoribosyltransferase (EC 2.4.2.9)	1
Novel non-oxidative pathway of Uracil catabolism	4
Uracil phosphoribosyltransferase (EC 2.4.2.9)	1
Urea carboxylase (EC 6.3.4.6)	1
Urease alpha subunit (EC 3.5.1.5)	1
Uridine kinase (EC 2.7.1.48)	1
pyrimidine conversions	15
2',3'-cyclic-nucleotide 2'-phosphodiesterase (EC 3.1.4.16)	1
5'-nucleotidase (EC 3.1.3.5)	1
Bis(5'-nucleosyl)-tetraphosphatase (asymmetrical) (EC 3.6.1.17)	1
CTP synthase (EC 6.3.4.2)	1
Cytidine deaminase (EC 3.5.4.5)	1
Cytidylate kinase (EC 2.7.4.14)	1
Cytosine deaminase (EC 3.5.4.1)	1
Deoxycytidine triphosphate deaminase (EC 3.5.4.13)	1
Purine nucleoside phosphorylase (EC 2.4.2.1)	1
Pyrimidine-nucleoside phosphorylase (EC 2.4.2.2)	1
Thioredoxin reductase (EC 1.8.1.9)	1
Thymidylate synthase (EC 2.1.1.45)	1
Uracil phosphoribosyltransferase (EC 2.4.2.9)	1
Uridine kinase (EC 2.7.1.48)	1
Uridine kinase (EC 2.7.1.48) [C1]	1
**Ribonucleotide reduction**	**7**
Ribonucleotide reduction	7
Ribonucleotide reductase of class Ia (aerobic), alpha subunit (EC 1.17.4.1)	1
Ribonucleotide reductase of class Ia (aerobic), beta subunit (EC 1.17.4.1)	1
Ribonucleotide reductase of class Ib (aerobic), beta subunit (EC 1.17.4.1)	1
Ribonucleotide reductase of class III (anaerobic), activating protein (EC 1.97.1.4)	1
Ribonucleotide reductase of class III (anaerobic), large subunit (EC 1.17.4.2)	1

Ribonucleotide reductase transcriptional regulator NrdR	1
Ribonucleotide reduction protein NrdI	1
**Phages, Prophages, Transposable elements, Plasmids**	**64**
**Bacteriophage integration/excision/lysogeny**	**2**
Phage integration and excision	2
Integrase	1
Phage integrase	1
**Bacteriophage structural proteins**	**1**
Phage capsid proteins	1
Phage capsid and scaffold	1
**Experimental**	**2**
Phage functions that need a home	2
Phage DNA invertase	1
Phage replication initiation	1
**Integrons**	**1**
Integrons	1
Integron integrase IntI4	1
**Pathogenicity islands**	**18**
Staphylococcal pathogenicity islands SaPI	5
GMP synthase [glutamine-hydrolyzing] (EC 6.3.5.2)	1
Heat shock protein 60 family chaperone GroEL	1
Methionine ABC transporter substrate-binding protein	1
SSU ribosomal protein S18p	1
tmRNA-binding protein SmpB	1
Vibrio pathogenicity island	13
Lipoprotein, ToxR-activated gene, TagA	1
TCP pilin signal peptidase, TcpA processing	1
Toxin co-regulated pilin A	1
Toxin co-regulated pilus biosynthesis protein C, outer membrane protein	1
Toxin co-regulated pilus biosynthesis protein D	1
Toxin co-regulated pilus biosynthesis protein E, anchors TcpT to membrane	1
Toxin co-regulated pilus biosynthesis protein F, putative outer membrane channel for TcpA extrusion	1
Toxin co-regulated pilus biosynthesis protein H, transcriptional activator of ToxT promoter	1
Toxin co-regulated pilus biosynthesis protein I, chemoreceptor, negative regulator of TcpA	1
Toxin co-regulated pilus biosynthesis protein P, transcriptional activator of ToxT promoter	1
Toxin co-regulated pilus biosynthesis protein Q	1
Toxin co-regulated pilus biosynthesis protein R	1

Toxin co-regulated pilus biosynthesis protein T, putative ATP-binding translocase of TcpA	1
**Phage family-specific subsystems**	**10**
Phage cyanophage	5
Phage protein	1
Phage tail fiber protein	1
Phosphate ABC transporter, periplasmic phosphate-binding protein PstS (TC 3.A.1.7.1)	1
Phosphate starvation-inducible protein PhoH, predicted ATPase	1
S-adenosylmethionine decarboxylase proenzyme (EC 4.1.1.50), prokaryotic class 1B	1
T4-like phage core proteins	4
DNA primase (EC 2.7.7.-)	1
Phage terminase, large subunit	1
Ribonucleotide reductase of class Ia (aerobic), alpha subunit (EC 1.17.4.1)	1
Ribonucleotide reductase of class Ia (aerobic), beta subunit (EC 1.17.4.1)	1
T7-like cyanophage core proteins	1
Transaldolase (EC 2.2.1.2)	1
**Phages, Prophages**	**21**
IbrA and IbrB: co-activators of prophage gene expression	1
Co-activator of prophage gene expression IbrA	1
Phage baseplate proteins	1
Phage baseplate	1
Phage packaging machinery	3
Phage terminase	1
Phage terminase small subunit	1
Phage terminase, large subunit	1
Phage tail fiber proteins	4
Phage long tail fiber proximal subunit	1
Phage tail fiber assembly protein	1
Phage tail fiber protein	1
Phage tail fibers	1
Phage tail proteins	2
Phage minor tail protein	1
Phage tail length tape-measure protein 1	1
Phage tail proteins 2	2
Phage minor tail protein	1
Phage tail length tape-measure protein 1	1
Prophage-encoded Rst operon	2
RstA phage-related replication protein	1

RstR phage-related transcriptional repressor	1
Staphylococcal phi-Mu50B-like prophages	1
Iron-sulfur cluster assembly protein SufB	1
T4-like phages core proteins	5
DNA primase (EC 2.7.7.-)	1
Phage terminase, large subunit	1
Ribonucleotide reductase of class Ia (aerobic), alpha subunit (EC 1.17.4.1)	1
Ribonucleotide reductase of class Ia (aerobic), beta subunit (EC 1.17.4.1)	1
Thymidylate synthase thyX (EC 2.1.1.-)	1
**Plasmid related functions**	**1**
Plasmid-encoded T-DNA transfer	1
Inner membrane protein of type IV secretion of T-DNA complex, VirB6	1
**Transposable elements**	**8**
CBSS-203122.12.peg.188	5
ISPsy4, transposition helper protein	1
Mll9366 protein	1
Plasmid replication protein RepA	1
Predicted nucleotidyltransferases	1
TniA putative transposase	1
Conjugative transposon, Bacteroidales	2
Conjugative transposon protein TraG	1
Conjugative transposon protein TraK	1
Tn552	1
Beta-lactamase (EC 3.5.2.6)	1
**Phosphorus Metabolism**	**47**
**Alkylphosphonate utilization**	**5**
Alkylphosphonate utilization	5
Metal-dependent hydrolase involved in phosphonate metabolism	1
PhnB protein	1
PhnI protein	1
PhnJ protein	1
Protein RcsF	1
**High affinity phosphate transporter and control of PHO regulon**	**5**
High affinity phosphate transporter and control of PHO regulon	5
Phosphate ABC transporter, periplasmic phosphate-binding protein PstS (TC 3.A.1.7.1)	1
Phosphate transport ATP-binding protein PstB (TC 3.A.1.7.1)	1
Phosphate transport system permease protein PstA (TC 3.A.1.7.1)	1

Phosphate transport system permease protein PstC (TC 3.A.1.7.1)	1
Polyphosphate kinase (EC 2.7.4.1)	1
**P uptake (cyanobacteria)**	**6**
P uptake (cyanobacteria)	6
Alkaline phosphatase (EC 3.1.3.1)	1
Phosphate ABC transporter, periplasmic phosphate-binding protein PstS (TC 3.A.1.7.1)	1
Phosphate regulon transcriptional regulatory protein PhoB	1
Phosphate transport ATP-binding protein PstB (TC 3.A.1.7.1)	1
Phosphate transport system permease protein PstA (TC 3.A.1.7.1)	1
Phosphate transport system permease protein PstC (TC 3.A.1.7.1)	1
**Phosphate metabolism**	**24**
Phosphate metabolism	24
1-acyl-sn-glycerol-3-phosphate acyltransferase (EC 2.3.1.51)	1
Alkaline phosphatase (EC 3.1.3.1)	1
Apolipoprotein N-acyltransferase (EC 2.3.1.-)	1
Exopolyphosphatase (EC 3.6.1.11)	1
FIG000233: metal-dependent hydrolase	1
Guanosine-5'-triphosphate,3'-diphosphate pyrophosphatase (EC 3.6.1.40)	1
Inorganic pyrophosphatase (EC 3.6.1.1)	1
Low-affinity inorganic phosphate transporter	1
Magnesium and cobalt efflux protein CorC	1
NAD(P) transhydrogenase subunit beta (EC 1.6.1.2)	1
Phosphate ABC transporter, periplasmic phosphate-binding protein PstS (TC 3.A.1.7.1)	1
Phosphate starvation-inducible ATPase PhoH with RNA binding motif	1
Phosphate starvation-inducible protein PhoH, predicted ATPase	1
Phosphate transport ATP-binding protein PstB (TC 3.A.1.7.1)	1
Phosphate transport regulator (distant homolog of PhoU)	1
Phosphate transport system permease protein PstA (TC 3.A.1.7.1)	1
Phosphate transport system permease protein PstC (TC 3.A.1.7.1)	1
Polyphosphate kinase (EC 2.7.4.1)	1
Predicted ATPase related to phosphate starvation-inducible protein PhoH	1
Probable low-affinity inorganic phosphate transporter	1
Pyrophosphate-energized proton pump (EC 3.6.1.1)	1
response regulator in two-component regulatory system with PhoQ	1
Sodium-dependent phosphate transporter	1
Soluble pyridine nucleotide transhydrogenase (EC 1.6.1.1)	1
**Phosphate-binding DING proteins**	**2**

Phosphate-binding DING proteins	2
Filamentous haemagglutinin family outer membrane protein associated with VreARI signalling system	1
Hemolysin activation/secretion protein associated with VreARI signalling system	1
**Phosphoenolpyruvate phosphomutase**	**1**
Phosphoenolpyruvate phosphomutase	1
2-aminoethylphosphonate:pyruvate aminotransferase (EC 2.6.1.37)	1
**Phosphonate metabolism**	**4**
Phosphonate metabolism	4
2-aminoethylphosphonate ABC transporter permease protein II (TC 3.A.1.9.1)	1
2-aminoethylphosphonate uptake and metabolism regulator	1
2-aminoethylphosphonate:pyruvate aminotransferase (EC 2.6.1.37)	1
Phosphonoacetaldehyde hydrolase (EC 3.11.1.1)	1
**Photosynthesis**	**9**
**Electron transport and photophosphorylation**	**6**
Photosystem I	3
photosystem I assembly related protein Ycf37	1
photosystem I P700 chlorophyll a apoprotein subunit Ib (PsaB)	1
photosystem I subunit X (PsaK, PsaK1)	1
Photosystem II	3
Photosystem II protein PsbN	1
Photosystem II protein PsbV, cytochrome c550	1
Putative chaperon-like protein Ycf39 for quinone binding in Photosystem II	1
**Light-harvesting complexes**	**2**
Phycobilisome	2
Phycobilisome core-membrane linker polypeptide	1
Phycoerythrocyanin beta chain	1
**Proteorhodopsin**	**1**
Proteorhodopsin	1
Phytoene synthase (EC 2.5.1.32)	1
**Potassium metabolism**	**21**
**Glutathione-regulated potassium-efflux system and associated functions**	**3**
Glutathione-regulated potassium-efflux system and associated functions	3
Glutathione-regulated potassium-efflux system ancillary protein KefG	1
Glutathione-regulated potassium-efflux system ATP-binding protein	1
Glutathione-regulated potassium-efflux system protein KefB	1
**Hyperosmotic potassium uptake**	**2**
Hyperosmotic potassium uptake	2

Potassium uptake protein, integral membrane component, KtrA	1
Potassium uptake protein, integral membrane component, KtrB	1
**Potassium homeostasis**	**16**
Potassium homeostasis	16
cAMP-dependent Kef-type K+ transport system	1
FKBP-type peptidyl-prolyl cis-trans isomerase FkpA precursor (EC 5.2.1.8)	1
FKBP-type peptidyl-prolyl cis-trans isomerase SlyD (EC 5.2.1.8)	1
Glutathione-regulated potassium-efflux system ancillary protein KefG	1
Glutathione-regulated potassium-efflux system ATP-binding protein	1
Glutathione-regulated potassium-efflux system protein KefC	1
Osmosensitive K+ channel histidine kinase KdpD (EC 2.7.3.-)	1
Potassium channel protein	1
Potassium uptake protein TrkH	1
Potassium uptake protein, integral membrane component, KtrA	1
Potassium uptake protein, integral membrane component, KtrB	1
POTASSIUM/PROTON ANTIPORTER ROSB	1
Potassium-transporting ATPase B chain (EC 3.6.3.12) (TC 3.A.3.7.1)	1
Potassium-transporting ATPase C chain (EC 3.6.3.12) (TC 3.A.3.7.1)	1
Putative cytoplasmic protein ,probably associated with Glutathione-regulated potassium-efflux	1
putative Glutathione-regulated potassium-efflux system protein KefB	1
**Protein Metabolism**	**212**
**Protein biosynthesis**	**126**
Programmed frameshift	1
Peptide chain release factor 2	1
Ribosome activity modulation	2
Ribosome hibernation protein YhbH	1
Ribosome modulation factor	1
Ribosome biogenesis bacterial	10
16S rRNA processing protein RimM	1
Dimethyladenosine transferase (EC 2.1.1.-)	1
hypothetical protein sometimes fused to ribosomal protein S6 glutaminyl transferase	1
Ribonuclease E (EC 3.1.26.12)	1
Ribosomal large subunit pseudouridine synthase A (EC 4.2.1.70)	1
Ribosomal large subunit pseudouridine synthase C (EC 4.2.1.70)	1
Ribosomal large subunit pseudouridine synthase D (EC 4.2.1.70)	1
Ribosomal protein S6 glutaminyl transferase	1
Similar to ribosomal large subunit pseudouridine synthase D, Bacillus subtilis YjbO type	1

tRNA (Guanine37-N1) -methyltransferase (EC 2.1.1.31)	1
Ribosome LSU bacterial	24
LSU ribosomal protein L11p (L12e)	1
LSU ribosomal protein L13p (L13Ae)	1
LSU ribosomal protein L14p (L23e)	1
LSU ribosomal protein L15p (L27Ae)	1
LSU ribosomal protein L16p (L10e)	1
LSU ribosomal protein L17p	1
LSU ribosomal protein L19p	1
LSU ribosomal protein L1p (L10Ae)	1
LSU ribosomal protein L20p	1
LSU ribosomal protein L21p	1
LSU ribosomal protein L23p (L23Ae)	1
LSU ribosomal protein L25p	1
LSU ribosomal protein L28p	1
LSU ribosomal protein L2p (L8e)	1
LSU ribosomal protein L30p (L7e)	1
LSU ribosomal protein L31p	1
LSU ribosomal protein L32p	1
LSU ribosomal protein L34p	1
LSU ribosomal protein L35p	1
LSU ribosomal protein L3p (L3e)	1
LSU ribosomal protein L4p (L1e)	1
LSU ribosomal protein L6p (L9e)	1
LSU ribosomal protein L7/L12 (P1/P2)	1
LSU ribosomal protein L9p	1
Ribosome LSU eukaryotic and archaeal	6
LSU ribosomal protein L13Ae (L13p)	1
LSU ribosomal protein L19e	1
LSU ribosomal protein L26e (L24p)	1
LSU ribosomal protein L32e	1
LSU ribosomal protein L37Ae	1
LSU ribosomal protein L7e (L30p)	1
Ribosome SSU bacterial	15
SSU ribosomal protein S10p (S20e)	1
SSU ribosomal protein S11p (S14e)	1
SSU ribosomal protein S12p (S23e)	1

SSU ribosomal protein S13p (S18e)	1
SSU ribosomal protein S14p (S29e)	1
SSU ribosomal protein S18p	1
SSU ribosomal protein S19p (S15e)	1
SSU ribosomal protein S1p	1
SSU ribosomal protein S20p	1
SSU ribosomal protein S21p	1
SSU ribosomal protein S3p (S3e)	1
SSU ribosomal protein S4p (S9e)	1
SSU ribosomal protein S6p	1
SSU ribosomal protein S8p (S15Ae)	1
SSU ribosomal protein S9p (S16e)	1
Ribosome SSU eukaryotic and archaeal	3
SSU ribosomal protein S2e (S5p)	1
SSU ribosomal protein S6e	1
SSU ribosomal protein S9e (S4p)	1
Ribosome SSU mitochondrial	1
SSU ribosomal protein S14p (S29e), mitochondrial	1
Translation elongation factor G family	4
Ribosome protection-type tetracycline resistance related proteins, group 2	1
Translation elongation factor G	1
Translation elongation factor G paralog	1
Translation elongation factor G-related protein	1
Translation elongation factors bacterial	8
Translation elongation factor G	1
Translation elongation factor G paralog	1
Translation elongation factor G-related protein	1
Translation elongation factor LepA	1
Translation elongation factor P	1
Translation elongation factor P-related protein	1
Translation elongation factor Ts	1
Translation elongation factor Tu	1
Translation elongation factors eukaryotic and archaeal	3
Eukaryotic peptide chain release factor subunit 1	1
Translation elongation factor 1 alpha-related protein	1
Translation elongation factor P	1
Translation initiation factors bacterial	5

Ribosome-binding factor A	1
Translation initiation factor 1	1
Translation initiation factor 2	1
Translation initiation factor 3	1
Translation initiation factor SUI1-related protein	1
Translation initiation factors eukaryotic and archaeal	3
Eukaryotic translation initiation factor 2 alpha subunit	1
Translation initiation factor 2	1
Translation initiation factor SUI1-related protein	1
Translation termination factors bacterial	9
Methionine aminopeptidase (EC 3.4.11.18)	1
Peptide chain release factor 1	1
Peptide chain release factor 2	1
Peptide chain release factor 3	1
Peptide chain release factor homolog	1
Peptidyl-tRNA hydrolase (EC 3.1.1.29)	1
Protein with similarity to RtcB	1
Ribosome recycling factor	1
tmRNA-binding protein SmpB	1
Trans-translation by stalled ribosomes	1
tmRNA-binding protein SmpB	1
tRNA aminoacylation, Asp and Asn	2
Asparaginyl-tRNA synthetase (EC 6.1.1.22)	1
Aspartyl-tRNA synthetase (EC 6.1.1.12)	1
tRNA aminoacylation, Cys	1
Cysteinyl-tRNA synthetase (EC 6.1.1.16)	1
tRNA aminoacylation, Glu and Gln	2
Glutamyl-tRNA synthetase (EC 6.1.1.17)	1
Glutamyl-tRNA(Gln) synthetase (EC 6.1.1.24)	1
tRNA aminoacylation, Gly	1
Glycyl-tRNA synthetase beta chain (EC 6.1.1.14)	1
tRNA aminoacylation, His	1
Histidyl-tRNA synthetase (EC 6.1.1.21)	1
tRNA aminoacylation, Ile	1
Isoleucyl-tRNA synthetase (EC 6.1.1.5)	1
tRNA aminoacylation, Lys	2
Lysyl-tRNA synthetase (class I) (EC 6.1.1.6)	1

Lysyl-tRNA synthetase (class II) (EC 6.1.1.6), mitochondrial	1
tRNA aminoacylation, Met	2
Methionyl-tRNA synthetase (EC 6.1.1.10)	1
tRNA-binding protein YgjH	1
tRNA aminoacylation, Phe	1
Phenylalanyl-tRNA synthetase beta chain (EC 6.1.1.20)	1
tRNA aminoacylation, Ser	1
Seryl-tRNA synthetase (EC 6.1.1.11)	1
tRNA aminoacylation, Thr	1
Threonyl-tRNA synthetase (EC 6.1.1.3)	1
tRNA aminoacylation, Trp	1
Tryptophanyl-tRNA synthetase (EC 6.1.1.2)	1
tRNA aminoacylation, Val	1
Valyl-tRNA synthetase (EC 6.1.1.9)	1
Universal GTPases	14
50S ribosomal subunit maturation GTPase RbgA (B. subtilis YlqF)	1
COG0536: GTP-binding protein Obg	1
GTPase and tRNA-U34 5-formylation enzyme TrmE	1
GTP-binding and nucleic acid-binding protein YchF	1
GTP-binding protein EngA	1
GTP-binding protein Era	1
GTP-binding protein TypA/BipA	1
GTP-binding protein YqeH, required for biogenesis of 30S ribosome subunit	1
Ribosome small subunit-stimulated GTPase EngC	1
Signal recognition particle receptor protein FtsY (=alpha subunit) (TC 3.A.5.1.1)	1
Translation elongation factor G	1
Translation elongation factor LepA	1
Translation elongation factor Tu	1
Translation initiation factor 2	1
**Protein degradation**	**42**
Aminopeptidases (EC 3.4.11.-)	3
Aminopeptidase S (Leu, Val, Phe, Tyr preference) (EC 3.4.11.24)	1
Membrane alanine aminopeptidase N (EC 3.4.11.2)	1
Peptidase B (EC 3.4.11.23)	1
Dipeptidases (EC 3.4.13.-)	2
Alpha-aspartyl dipeptidase Peptidase E (EC 3.4.13.21)	1
Aminoacyl-histidine dipeptidase (Peptidase D) (EC 3.4.13.3)	1

Metallocarboxypeptidases (EC 3.4.17.-)	2
Muramoyltetrapeptide carboxypeptidase (EC 3.4.17.13)	1
Thermostable carboxypeptidase 1 (EC 3.4.17.19)	1
Metalloendopeptidases (EC 3.4.24.-)	2
Microbial collagenase (EC 3.4.24.3)	1
Microbial collagenase, secreted (EC 3.4.24.3)	1
Omega peptidases (EC 3.4.19.-)	2
Acylamino-acid-releasing enzyme (EC 3.4.19.1)	1
Pyrrolidone-carboxylate peptidase (EC 3.4.19.3)	1
Proteasome archaeal	1
Bacterial proteasome-activating AAA-ATPase (PAN)	1
Proteasome bacterial	5
ATP-dependent Clp protease proteolytic subunit (EC 3.4.21.92)	1
ATP-dependent hsl protease ATP-binding subunit HslU	1
ATP-dependent protease La (EC 3.4.21.53) Type I	1
ATP-dependent protease La (EC 3.4.21.53) Type II	1
Uncharacterized protein, similar to the N-terminal domain of Lon protease	1
Proteasome eukaryotic	3
proteasome regulatory subunit Rpn1	1
proteasome regulatory subunit Rpn10	1
proteasome subunit beta10 (EC 3.4.25.1)	1
Protein degradation	5
Aminopeptidase YpdF (MP-, MA-, MS-, AP-, NP- specific)	1
Dipeptidyl carboxypeptidase Dcp (EC 3.4.15.5)	1
Leucyl/phenylalanyl-tRNA--protein transferase (EC 2.3.2.6)	1
Oligopeptidase A (EC 3.4.24.70)	1
Thimet oligopeptidase (EC 3.4.24.15)	1
Proteolysis in bacteria, ATP-dependent	11
ATPase, AFG1 family	1
ATP-dependent Clp protease adaptor protein ClpS	1
ATP-dependent Clp protease ATP-binding subunit ClpA	1
ATP-dependent Clp protease proteolytic subunit (EC 3.4.21.92)	1
ATP-dependent hsl protease ATP-binding subunit HslU	1
ATP-dependent protease domain protein (EC 3.4.21.-)	1
ATP-dependent protease La (EC 3.4.21.53)	1
ATP-dependent protease La (EC 3.4.21.53) Type I	1
ATP-dependent protease La (EC 3.4.21.53) Type II	1

Outer membrane stress sensor protease DegQ, serine protease	1
Outer membrane stress sensor protease DegS	1
Putative TldE-TldD proteolytic complex	5
FIG138315: Putative alpha helix protein	1
TldD family protein, Actinobacterial subgroup	1
TldD protein, part of proposed TldE/TldD proteolytic complex (PMID 12029038)	1
TldE/PmbA family protein, Actinobacterial subgroup	1
TldE/PmbA protein, part of proposed TldE/TldD proteolytic complex (PMID 12029038)	1
Serine endopeptidase (EC 3.4.21.-)	1
Prolyl endopeptidase (EC 3.4.21.26)	1
**Protein folding**	**20**
GroEL GroES	2
Heat shock protein 60 family chaperone GroEL	1
Heat shock protein 60 family co-chaperone GroES	1
Peptidyl-prolyl cis-trans isomerase	9
FKBP-type peptidyl-prolyl cis-trans isomerase fklB (EC 5.2.1.8)	1
FKBP-type peptidyl-prolyl cis-trans isomerase FkpA precursor (EC 5.2.1.8)	1
FKBP-type peptidyl-prolyl cis-trans isomerase slpA (EC 5.2.1.8)	1
FKBP-type peptidyl-prolyl cis-trans isomerase SlyD (EC 5.2.1.8)	1
Foldase protein PrsA precursor (EC 5.2.1.8)	1
Peptidyl-prolyl cis-trans isomerase ppiA precursor (EC 5.2.1.8)	1
Peptidyl-prolyl cis-trans isomerase ppiC (EC 5.2.1.8)	1
Peptidyl-prolyl cis-trans isomerase ppiD (EC 5.2.1.8)	1
Survival protein SurA precursor (Peptidyl-prolyl cis-trans isomerase SurA) (EC 5.2.1.8)	1
Periplasmic disulfide interchange	4
Cytochrome c-type biogenesis protein CcdA (DsbD analog)	1
Cytochrome c-type biogenesis protein CcmG/DsbE, thiol:disulfide oxidoreductase	1
Cytochrome c-type biogenesis protein DsbD, protein-disulfide reductase (EC 1.8.1.8)	1
Periplasmic thiol:disulfide interchange protein DsbA	1
Protein chaperones	5
Chaperone protein DnaK	1
Chaperone protein HscB	1
Chaperone-modulator protein CbpM	1
Heat shock protein GrpE	1
HspR, transcriptional repressor of DnaK operon	1
**Protein processing and modification**	**19**
Lipoprotein Biosynthesis	3

Apolipoprotein N-acyltransferase (EC 2.3.1.-)	1
Lipoprotein signal peptidase (EC 3.4.23.36)	1
Prolipoprotein diacylglyceryl transferase (EC 2.4.99.-)	1
N-linked Glycosylation in Bacteria	6
4-keto-6-deoxy-N-Acetyl-D-hexosaminyl-(Lipid carrier) aminotransferase	1
Alpha-1,4-N-acetylgalactosamine transferase PglH (EC 2.4.1.-)	1
Lipid carrier : UDP-N-acetylgalactosaminyltransferase (EC 2.4.1.-)	1
Oligosaccharyltransferase PglB (EC 2.4.1.119)	1
UDP-glucose 4-epimerase (EC 5.1.3.2)	1
UDP-N-acetylglucosamine 4,6-dehydratase (EC 4.2.1.-)	1
Peptide methionine sulfoxide reductase	1
Peptide methionine sulfoxide reductase MsrB (EC 1.8.4.12)	1
Protein Acetylation and Deacetylation in Bacteria	3
Acetate permease ActP (cation/acetate symporter)	1
Acetyl-coenzyme A synthetase (EC 6.2.1.1)	1
Protein acetyltransferase	1
Ribosomal protein S12p Asp methylthiotransferase	4
MiaB family protein, possibly involved in tRNA or rRNA modification	1
Ribosomal protein S12p Asp88 (E. coli) methylthiotransferase	1
SSU ribosomal protein S12p (S23e)	1
tRNA-i(6)A37 methylthiotransferase	1
Signal peptidase	2
Lipoprotein signal peptidase (EC 3.4.23.36)	1
Signal peptidase I (EC 3.4.21.89)	1
**Selenoproteins**	**5**
Glycine reductase, sarcosine reductase and betaine reductase	2
Thioredoxin	1
Thioredoxin reductase (EC 1.8.1.9)	1
Selenocysteine metabolism	2
L-seryl-tRNA(Sec) selenium transferase (EC 2.9.1.1)	1
Selenocysteine-specific translation elongation factor	1
Selenoprotein O	1
Selenoprotein O and cysteine-containing homologs	1
**Regulation and Cell signaling**	**110**
**cAMP signaling in bacteria**	**10**
cAMP signaling in bacteria	10
3',5'-cyclic-nucleotide phosphodiesterase (EC 3.1.4.17)	1

Adenylate cyclase (EC 4.6.1.1)	1
ATP-dependent Clp protease proteolytic subunit (EC 3.4.21.92)	1
cAMP-binding proteins - catabolite gene activator and regulatory subunit of cAMP-dependent protein kinases	1
cAMP-dependent Kef-type K+ transport system	1
Cyclic AMP receptor protein	1
Heme-regulated cyclic AMP phosphodiesterase (EC 3.1.4.-)	1
Hydrolase, alpha/beta fold family functionally coupled to Phosphoribulokinase	1
Predicted signal-transduction protein containing cAMP-binding and CBS domains	1
Prophage Clp protease-like protein	1
**Cell envelope-associated LytR-CpsA-Psr transcriptional attenuators**	**2**
Cell envelope-associated LytR-CpsA-Psr transcriptional attenuators	2
Cell envelope-associated transcriptional attenuator LytR-CpsA-Psr, subfamily F2 (as in PMID19099556)	1
Cell envelope-associated transcriptional attenuator LytR-CpsA-Psr, subfamily M (as in PMID19099556)	1
**CytR regulation**	**1**
CytR regulation	1
Cyclic AMP receptor protein	1
**DNA-binding regulatory proteins, strays**	**11**
DNA-binding regulatory proteins, strays	11
Alkanesulfonate utilization operon LysR-family regulator Cbl	1
Aromatic hydrocarbon utilization transcriptional regulator CatR (LysR family)	1
Cys regulon transcriptional activator CysB	1
GltC, transcription activator of glutamate synthase operon	1
Hydrogen peroxide-inducible genes activator	1
LysR family transcriptional regulator near succinyl-CoA:3-ketoacid-coenzyme A transferase	1
LysR family transcriptional regulator PA2877	1
LysR family transcriptional regulator PA3398	1
LysR family transcriptional regulator STM2281	1
LysR family transcriptional regulator STM3121	1
LysR family transcriptional regulator YbhD	1
**Global Two-component Regulator PrrBA in Proteobacteria**	**1**
Global Two-component Regulator PrrBA in Proteobacteria	1
Sensor histidine kinase PrrB (RegB) (EC 2.7.3.-)	1
**Orphan regulatory proteins**	**15**
Orphan regulatory proteins	15
Copper-sensing two-component system response regulator CpxR	1
Copper-sensing two-component system response regulator CusR	1
DNA transformation protein TfoX	1

Glycine cleavage system transcriptional activator	1
Glycine cleavage system transcriptional activator GcvA	1
Putative sensor-like histidine kinase YfhK	1
Putative sensory histidine kinase YfhA	1
Putative two-component response regulator and GGDEF family protein YeaJ	1
Sensor kinase CitA, DpiB (EC 2.7.3.-)	1
Sensor protein basS/pmrB (EC 2.7.3.-)	1
Sensory histidine kinase in two-component regulatory system with RstA	1
Sensory histidine kinase QseC	1
Transcriptional regulatory protein CitB, DpiA	1
Transcriptional regulatory protein RstA	1
Two-component system response regulator QseB	1
**Oxygen and light sensor PpaA-PpsR**	**1**
Oxygen and light sensor PpaA-PpsR	1
Phytochrome, two-component sensor histidine kinase (EC 2.7.3.-)	1
**Programmed Cell Death and Toxin-antitoxin Systems**	**16**
MazEF toxin-antitoxing (programmed cell death) system	1
Programmed cell death toxin YdcE	1
Murein hydrolase regulation and cell death	8
Autolysis histidine kinase LytS	1
Cytidine deaminase (EC 3.5.4.5)	1
Holin-like protein CidA	1
LrgA-associated membrane protein LrgB	1
LysR family regulatory protein CidR	1
Preprotein translocase subunit SecG (TC 3.A.5.1.1)	1
S-layer protein Sap	1
tRNA-dihydrouridine synthase C (EC 1.-.-.-)	1
Phd-Doc, YdcE-YdcD toxin-antitoxin (programmed cell death) systems	3
Death on curing protein, Doc toxin	1
Prevent host death protein, Phd antitoxin	1
Programmed cell death toxin YdcE	1
Toxin-antitoxin systems (other than RelBE and MazEF)	4
DNA-damage-inducible protein J	1
HigA protein (antitoxin to HigB)	1
ParD protein (antitoxin to ParE)	1
ParE toxin protein	1
**Proteolytic pathway**	**1**

Coagulation cascade	1
Fibrinogen alpha chain	1
**Pseudomonas quinolone signal PQS**	**1**
Pseudomonas quinolone signal PQS	1
BarA-associated response regulator UvrY (= GacA = SirA)	1
**Quorum sensing and biofilm formation**	**18**
Autoinducer 2 (AI-2) transport and processing (lsrACDBFGE operon)	5
Autoinducer 2 (AI-2) ABC transport system, fused AI2 transporter subunits and ATP-binding component	1
Autoinducer 2 (AI-2) kinase LsrK (EC 2.7.1.-)	1
Autoinducer 2 sensor kinase/phosphatase LuxQ (EC 2.7.3.-) (EC 3.1.3.-)	1
Autoinducer 2-binding periplasmic protein LuxP precursor	1
LsrR, transcriptional repressor of lsr operon	1
Biofilm Adhesin Biosynthesis	1
Biofilm PGA synthesis deacetylase PgaB (EC 3.-)	1
Biofilm formation in Staphylococcus	1
RNA polymerase sigma factor SigB	1
Quorum sensing regulation in Pseudomonas	1
BarA-associated response regulator UvrY (= GacA = SirA)	1
Quorum Sensing: Autoinducer-2 Synthesis	1
S-adenosylmethionine synthetase (EC 2.5.1.6)	1
Quorum-sensing in Vibrio	9
8-amino-7-oxononanoate synthase (EC 2.3.1.47) / CqsA	1
Autoinducer 2 sensor kinase/phosphatase LuxQ (EC 2.7.3.-) (EC 3.1.3.-)	1
Autoinducer 2-binding periplasmic protein LuxP precursor	1
N-(3-hydroxybutanoyl)-L- homoserine lactone synthase LuxM	1
Phosphorelay protein LuxU	1
Quorum-sensing regulator of virulence HapR	1
Regulatory protein LuxO	1
Sensor histidine kinase CqsS	1
S-ribosylhomocysteine lyase (EC 4.4.1.21) / Autoinducer-2 production protein LuxS	1
**Rcs phosphorelay signal transduction pathway**	**1**
Rcs phosphorelay signal transduction pathway	1
Protein RcsF	1
**Regulation of virulence**	**6**
A conserved operon linked to TyrR and possibly involved in virulence	1
Membrane protein YcjF	1
Streptococcal Mga Regulon	2

Immunogenic secreted protein	1
Two-component system histidine kinase	1
VieSAB signal transduction system of Vibrio	3
Response regulator VieA	1
Response regulator VieB	1
Sensory box sensor histidine kinase/response regulator VieS	1
**Sex pheromones in Enterococcus faecalis and other Firmicutes**	**3**
Sex pheromones in Enterococcus faecalis and other Firmicutes	3
Lipoprotein signal peptidase (EC 3.4.23.36)	1
Pheromone response surface protein PrgC	1
Putative pheromone cAM373 precursor lipoprotein CamS	1
**Stringent Response, (p)ppGpp metabolism**	**2**
Stringent Response, (p)ppGpp metabolism	2
D-tyrosyl-tRNA(Tyr) deacylase	1
Guanosine-5'-triphosphate,3'-diphosphate pyrophosphatase (EC 3.6.1.40)	1
**The Chv regulatory system of Alphaproteobacteria**	**2**
The Chv regulatory system of Alphaproteobacteria	2
Phosphocarrier protein, nitrogen regulation associated	1
Sensor histidine kinase ChvG (EC 2.7.3.-)	1
**Trans-envelope signaling system VreARI in Pseudomonas**	**2**
Trans-envelope signaling system VreARI in Pseudomonas	2
Filamentous haemagglutinin family outer membrane protein associated with VreARI signalling system	1
Hemolysin activation/secretion protein associated with VreARI signalling system	1
**Two-component regulatory systems in Campylobacter**	**3**
Two-component regulatory systems in Campylobacter	3
Positive regulator of CheA protein activity (CheW)	1
Signal transduction histidine kinase CheA (EC 2.7.3.-)	1
Two-component system histidine kinase	1
**Two-component sensor regulator linked to Carbon Starvation Protein A**	**3**
Two-component sensor regulator linked to Carbon Starvation Protein A	3
Autolysin sensor kinase (EC 2.7.3.-)	1
Carbon starvation protein A	1
FIG001014_Response regulator of the LytR/AlgR family	1
**Zinc regulated enzymes**	**11**
Zinc regulated enzymes	11
C4-type zinc finger protein, DksA/TraR family	1
Carbonic anhydrase (EC 4.2.1.1)	1

Cysteinyl-tRNA synthetase (EC 6.1.1.16)	1
Dihydroorotase (EC 3.5.2.3)	1
GTP cyclohydrolase I (EC 3.5.4.16) type 1	1
GTP cyclohydrolase I (EC 3.5.4.16) type 2	1
N-acetylmuramoyl-L-alanine amidase (EC 3.5.1.28)	1
Porphobilinogen synthase (EC 4.2.1.24)	1
Putative metal chaperone, involved in Zn homeostasis, GTPase of COG0523 family	1
Queuosine biosynthesis QueD, PTPS-I	1
Zinc uptake regulation protein ZUR	1
**Respiration**	**186**
**ATP synthases**	**10**
F0F1-type ATP synthase	8
ATP synthase A chain (EC 3.6.3.14)	1
ATP synthase alpha chain (EC 3.6.3.14)	1
ATP synthase B chain (EC 3.6.3.14)	1
ATP synthase beta chain (EC 3.6.3.14)	1
ATP synthase C chain (EC 3.6.3.14)	1
ATP synthase delta chain (EC 3.6.3.14)	1
ATP synthase epsilon chain (EC 3.6.3.14)	1
ATP synthase protein I	1
V-Type ATP synthase	2
V-type ATP synthase subunit A (EC 3.6.3.14)	1
V-type ATP synthase subunit I (EC 3.6.3.14)	1
**Biogenesis of cbb3-type cytochrome c oxidases**	**2**
Biogenesis of cbb3-type cytochrome c oxidases	2
Putative analog of CcoH, COG3198	1
Type cbb3 cytochrome oxidase biogenesis protein CcoS, involved in heme b insertion	1
**Biogenesis of c-type cytochromes**	**15**
Biogenesis of c-type cytochromes	15
ABC transporter involved in cytochrome c biogenesis, CcmB subunit	1
Ccs1/ResB-related putative cytochrome C-type biogenesis protein	1
Cytochrome c heme lyase subunit CcmF	1
Cytochrome c heme lyase subunit CcmH	1
Cytochrome c-type biogenesis protein CcdA (DsbD analog)	1
Cytochrome c-type biogenesis protein CcmD, interacts with CcmCE	1
Cytochrome c-type biogenesis protein CcmE, heme chaperone	1
Cytochrome c-type biogenesis protein CcmG/DsbE, thiol:disulfide oxidoreductase	1

Cytochrome c-type biogenesis protein Ccs1/ResB	1
Cytochrome c-type biogenesis protein CcsA/ResC	1
Cytochrome c-type biogenesis protein DsbD, protein-disulfide reductase (EC 1.8.1.8)	1
Cytochrome c-type biogenesis protein ResA	1
Periplasmic thiol:disulfide interchange protein DsbA	1
Putative cytochrome C-type biogenesis protein	1
Thiol:disulfide oxidoreductase related to ResA	1
**Biogenesis of cytochrome c oxidases**	**6**
Biogenesis of cytochrome c oxidases	6
Copper metallochaperone, bacterial analog of Cox17 protein	1
Cytochrome oxidase biogenesis protein Sco1/SenC/PrrC, putative copper metallochaperone	1
Cytochrome oxidase biogenesis protein Surf1, facilitates heme A insertion	1
Ferredoxin--NADP(+) reductase (EC 1.18.1.2)	1
Heme A synthase, cytochrome oxidase biogenesis protein Cox15-CtaA	1
Heme O synthase, protoheme IX farnesyltransferase (EC 2.5.1.-) COX10-CtaB	1
**Carbon monoxide dehydrogenase maturation factors**	**3**
Carbon monoxide dehydrogenase maturation factors	3
carbon monoxide dehydrogenase D protein	1
carbon monoxide dehydrogenase E protein	1
Carbon monoxide dehydrogenase F protein	1
**Carbon monoxide induced hydrogenase**	**2**
Carbon monoxide induced hydrogenase	2
Acetyl-CoA synthase corrinoid iron-sulfur protein, large subunit	1
Carbon monoxide dehydrogenase CooS subunit (EC 1.2.99.2)	1
**Electron accepting reactions**	**51**
Anaerobic respiratory reductases	14
Anaerobic dehydrogenases, typically selenocysteine-containing	1
Anaerobic dimethyl sulfoxide reductase chain A (EC 1.8.99.-)	1
Anaerobic dimethyl sulfoxide reductase chain C (EC 1.8.99.-)	1
Arsenate reductase (EC 1.20.4.1)	1
CoB--CoM heterodisulfide reductase subunit A (EC 1.8.98.1)	1
Dissimilatory sulfite reductase (desulfoviridin), alpha and beta subunits	1
Electron transfer flavoprotein-ubiquinone oxidoreductase (EC 1.5.5.1)	1
Ferric reductase (1.6.99.14)	1
Flavodoxin reductases (ferredoxin-NADPH reductases) family 1	1
Formate dehydrogenase -O, gamma subunit (EC 1.2.1.2)	1
Heterodisulfide reductase, cytochrome reductase subunit	1

heterodisulfide reductase, iron-sulfur binding subunit, putative	1
Polysulfide reductase, subunit B, putative	1
polysulfide reductase, subunit C	1
Cytochrome c oxidases d@O copy	7
Cytochrome d ubiquinol oxidase subunit II (EC 1.10.3.-)	1
Cytochrome O ubiquinol oxidase subunit II (EC 1.10.3.-)	1
putative Cytochrome bd2, subunit I	1
putative Cytochrome bd2, subunit II	1
Transport ATP-binding protein CydC	1
Transport ATP-binding protein CydCD	1
Transport ATP-binding protein CydD	1
Fumarate respiration cluster	2
C4-dicarboxylate transporter DcuB	1
Fumarate respiration transcriptional regulator DcuR	1
Terminal cytochrome C oxidases	5
Cytochrome c oxidase polypeptide I (EC 1.9.3.1)	1
Cytochrome c oxidase polypeptide II (EC 1.9.3.1)	1
Cytochrome c oxidase subunit CcoO (EC 1.9.3.1)	1
Cytochrome c oxidase subunit CcoQ (EC 1.9.3.1)	1
Type cbb3 cytochrome oxidase biogenesis protein CcoS, involved in heme b insertion	1
Terminal cytochrome d ubiquinol oxidases	6
Cytochrome d ubiquinol oxidase subunit II (EC 1.10.3.-)	1
putative Cytochrome bd2, subunit I	1
putative Cytochrome bd2, subunit II	1
Transport ATP-binding protein CydC	1
Transport ATP-binding protein CydCD	1
Transport ATP-binding protein CydD	1
Terminal cytochrome O ubiquinol oxidase	1
Cytochrome O ubiquinol oxidase subunit II (EC 1.10.3.-)	1
Terminal cytochrome oxidases	7
Cytochrome d ubiquinol oxidase subunit II (EC 1.10.3.-)	1
Cytochrome O ubiquinol oxidase subunit II (EC 1.10.3.-)	1
putative Cytochrome bd2, subunit I	1
putative Cytochrome bd2, subunit II	1
Transport ATP-binding protein CydC	1
Transport ATP-binding protein CydCD	1
Transport ATP-binding protein CydD	1

trimethylamine N-oxide (TMAO) reductase	8
Biotin sulfoxide reductase (EC 1.-.-.-)	1
Cytochrome c-type protein NapC	1
Cytochrome c-type protein TorC	1
Cytochrome c-type protein TorY	1
Hypothetical iron-sulfur cluster binding protein YccM	1
Periplasmic protein torT precursor	1
Sensor protein torS (EC 2.7.3.-)	1
Trimethylamine-N-oxide reductase (EC 1.6.6.9)	1
Ubiquinone Menaquinone-cytochrome c reductase complexes	1
ubiquinol cytochrome C oxidoreductase, cytochrome C1 subunit	1
**Electron donating reactions**	**69**
CO Dehydrogenase	6
carbon monoxide dehydrogenase D protein	1
carbon monoxide dehydrogenase E protein	1
Carbon monoxide dehydrogenase F protein	1
Carbon monoxide dehydrogenase large chain (EC 1.2.99.2)	1
Carbon monoxide dehydrogenase large chain (EC 1.2.99.2) parolog without usual motifs	1
CO dehydrogenases maturation factor, CoxF family	1
Coenzyme F420 hydrogenase	1
Coenzyme F420 hydrogenase maturation protease (EC 3.4.24.-)	1
Energy-conserving hydrogenase (ferredoxin)	1
Energy-conserving hydrogenase (ferredoxin), subunit A	1
Formate dehydrogenase	1
Formate dehydrogenase beta subunit (EC 1.2.1.2)	1
H2:CoM-S-S-HTP oxidoreductase	2
CoB--CoM heterodisulfide reductase subunit A (EC 1.8.98.1)	1
CoB--CoM-reducing hydrogenase (Cys) gamma subunit	1
Hydrogenases	7
[Ni/Fe] hydrogenase, group 1, small subunit	1
Coenzyme F420 hydrogenase maturation protease (EC 3.4.24.-)	1
NAD-reducing hydrogenase subunit HoxH (EC 1.12.1.2)	1
Ni,Fe-hydrogenase I cytochrome b subunit	1
Ni,Fe-hydrogenase III large subunit	1
Ni/Fe-hydrogenase 2 B-type cytochrome subunit	1
Quinone-reactive Ni/Fe-hydrogenase small chain precursor (EC 1.12.5.1)	1
Membrane-bound Ni, Fe-hydrogenase	2

Hydrogenase maturation protease (EC 3.4.24.-)	1
Ni,Fe-hydrogenase I cytochrome b subunit	1
Na(+)-translocating NADH-quinone oxidoreductase and rnf-like group of electron transport complexes	9
Electron transport complex protein RnfB	1
Electron transport complex protein RnfC	1
Electron transport complex protein RnfD	1
Electron transport complex protein RnfE	1
Electron transport complex protein RnfG	1
Na(+)-translocating NADH-quinone reductase subunit A (EC 1.6.5.-)	1
Na(+)-translocating NADH-quinone reductase subunit B (EC 1.6.5.-)	1
Na(+)-translocating NADH-quinone reductase subunit C (EC 1.6.5.-)	1
Probable exported or periplasmic protein in ApbE locus	1
NiFe hydrogenase maturation	4
[NiFe] hydrogenase metallocenter assembly protein HypC	1
[NiFe] hydrogenase metallocenter assembly protein HypD	1
[NiFe] hydrogenase nickel incorporation-associated protein HypB	1
Energy-conserving hydrogenase (ferredoxin), subunit A	1
Respiratory Complex I	12
NADH dehydrogenase I subunit 4, Involved in photosystem-1 cyclic electron flow	1
NADH dehydrogenase subunit 4L	1
NADH dehydrogenase, subunit 5	1
NADH-ubiquinone oxidoreductase chain C (EC 1.6.5.3)	1
NADH-ubiquinone oxidoreductase chain D (EC 1.6.5.3)	1
NADH-ubiquinone oxidoreductase chain F (EC 1.6.5.3)	1
NADH-ubiquinone oxidoreductase chain G (EC 1.6.5.3)	1
NADH-ubiquinone oxidoreductase chain H (EC 1.6.5.3)	1
NADH-ubiquinone oxidoreductase chain I (EC 1.6.5.3)	1
NADH-ubiquinone oxidoreductase chain J (EC 1.6.5.3)	1
NADH-ubiquinone oxidoreductase chain K (EC 1.6.5.3)	1
NADH-ubiquinone oxidoreductase chain L (EC 1.6.5.3)	1
Respiratory dehydrogenases 1	17
Aerobic glycerol-3-phosphate dehydrogenase (EC 1.1.5.3)	1
Anaerobic glycerol-3-phosphate dehydrogenase subunit A (EC 1.1.5.3)	1
Anaerobic glycerol-3-phosphate dehydrogenase subunit B (EC 1.1.5.3)	1
Anaerobic glycerol-3-phosphate dehydrogenase subunit C (EC 1.1.5.3)	1
D-amino acid dehydrogenase small subunit (EC 1.4.99.1)	1
D-Lactate dehydrogenase (EC 1.1.2.5)	1

Glucose dehydrogenase, PQQ-dependent (EC 1.1.5.2)	1
Glucose-methanol-choline (GMC) oxidoreductase:NAD binding site	1
Glycerol dehydrogenase (EC 1.1.1.6)	1
Glycerol-1-phosphate dehydrogenase [NAD(P)] (EC 1.1.1.261)	1
Glycerol-3-phosphate dehydrogenase (EC 1.1.5.3)	1
L-lactate dehydrogenase (EC 1.1.2.3)	1
Methanol dehydrogenase large subunit protein (EC 1.1.99.8)	1
Methylamine dehydrogenase heavy chain precursor (EC 1.4.99.3)	1
NADH dehydrogenase (EC 1.6.99.3)	1
Proline dehydrogenase (EC 1.5.99.8) (Proline oxidase)	1
putative Fe-S, FMN containing oxidoreductase	1
Succinate dehydrogenase	7
Fumarate reductase flavoprotein subunit (EC 1.3.99.1)	1
Fumarate reductase subunit C	1
Fumarate reductase subunit D	1
Succinate dehydrogenase cytochrome b-556 subunit	1
Succinate dehydrogenase flavoprotein subunit (EC 1.3.99.1)	1
Succinate dehydrogenase hydrophobic membrane anchor protein	1
Succinate dehydrogenase iron-sulfur protein (EC 1.3.99.1)	1
**Formate hydrogenase**	**12**
Formate hydrogenase	12
Formate dehydrogenase chain D (EC 1.2.1.2)	1
formate dehydrogenase formation protein FdhE	1
Formate dehydrogenase O putative subunit	1
Formate dehydrogenase -O, gamma subunit (EC 1.2.1.2)	1
Formate dehydrogenase-O, major subunit (EC 1.2.1.2)	1
Formate hydrogenlyase subunit 5	1
Formate hydrogenlyase subunit 7	1
Formate hydrogenlyase transcriptional activator	1
Hydrogenase-4 component C (EC 1.-.-.-)	1
Hydrogenase-4 component F (EC 1.-.-.-)	1
NAD-dependent formate dehydrogenase delta subunit	1
Putative formate dehydrogenase-specific chaperone	1
**Methanogenesis strays**	**2**
Methanogenesis strays	2
Uncharacterized protein MJ0065	1
UPF0288 protein MJ1412	1

**Quinone oxidoreductase family**	**2**
Quinone oxidoreductase family	2
Putative oxidoreductase SMc00968	1
Quinone oxidoreductase (EC 1.6.5.5)	1
**Sodium Ion-Coupled Energetics**	**2**
Na+ translocating decarboxylases and related biotin-dependent enzymes	2
Oxaloacetate decarboxylase alpha chain (EC 4.1.1.3)	1
Oxaloacetate decarboxylase gamma chain (EC 4.1.1.3)	1
**Soluble cytochromes and functionally related electron carriers**	**10**
Soluble cytochromes and functionally related electron carriers	10
Cytochrome c4	1
Cytochrome c551/c552	1
Cytochrome c552 precursor (EC 1.7.2.2)	1
Cytochrome c553	1
Cytochrome C553 (soluble cytochrome f)	1
Ferredoxin	1
Photosystem II protein PsbV, cytochrome c550	1
Putative diheme cytochrome c-553	1
soluble [2Fe-2S] ferredoxin	1
Soluble cytochrome b562	1
**RNA Metabolism**	**232**
**RNA processing and modification**	**204**
16S rRNA modification within P site of ribosome	5
2-dehydropantoate 2-reductase (EC 1.1.1.169)	1
Cell division protein FtsI [Peptidoglycan synthetase] (EC 2.4.1.129)	1
Cell division protein FtsL	1
Penicillin-binding protein 2 (PBP-2)	1
rRNA small subunit methyltransferase H	1
ATP-dependent RNA helicases, bacterial	10
ATP-dependent RNA helicase DbpA	1
ATP-dependent RNA helicase NGO0650	1
ATP-dependent RNA helicase RhlE	1
ATP-dependent RNA helicase SrmB	1
ATP-dependent RNA helicase VC1407	1
ATP-dependent RNA helicase VCA0061	1
ATP-dependent RNA helicase VCA0990	1
ATP-dependent RNA helicase YfmL	1

ATP-dependent RNA helicase YxiN	1
Cold-shock DEAD-box protein A	1
COG1901	2
tRNA (Uracil54-C5-)-methyltransferase (EC 2.1.1.35)	1
tRNA pseudouridine synthase B (EC 4.2.1.70)	1
Methylthiotransferases	3
MiaB family protein, possibly involved in tRNA or rRNA modification	1
Ribosomal protein S12p Asp88 (E. coli) methylthiotransferase	1
tRNA-i(6)A37 methylthiotransferase	1
mnm5U34 biosynthesis bacteria	9
5-methylaminomethyl-2-thiouridine-forming enzyme mnmC	1
Cysteine desulfurase (EC 2.8.1.7), IscS subfamily	1
Cysteine desulfurase (EC 2.8.1.7), SufS subfamily	1
GTPase and tRNA-U34 5-formylation enzyme TrmE	1
tRNA (5-methylaminomethyl-2-thiouridylate)-methyltransferase (EC 2.1.1.61)	1
tRNA 5-methylaminomethyl-2-thiouridine synthase TusA	1
tRNA 5-methylaminomethyl-2-thiouridine synthase TusB	1
tRNA 5-methylaminomethyl-2-thiouridine synthase TusC	1
tRNA uridine 5-carboxymethylaminomethyl modification enzyme GidA	1
Polyadenylation bacterial	4
Poly(A) polymerase (EC 2.7.7.19)	1
Polyribonucleotide nucleotidyltransferase (EC 2.7.7.8)	1
RNA-binding protein Hfq	1
tRNA nucleotidyltransferase (EC 2.7.7.21) (EC 2.7.7.25)	1
Queuosine-Archaeosine Biosynthesis	14
glutamyl-Q-tRNA synthetase	1
GTP cyclohydrolase I (EC 3.5.4.16) type 1	1
GTP cyclohydrolase I (EC 3.5.4.16) type 2	1
Inosine-uridine preferring nucleoside hydrolase (EC 3.2.2.1)	1
NADPH dependent preQ0 reductase	1
Peptidyl-prolyl cis-trans isomerase (EC 5.2.1.8)	1
Putative preQ0 transporter	1
Queuosine Biosynthesis QueC ATPase	1
Queuosine biosynthesis QueD, PTPS-I	1
Queuosine Biosynthesis QueE Radical SAM	1
S-adenosylmethionine:tRNA ribosyltransferase-isomerase (EC 5.-.-.-)	1
Substrate-specific component QueT (COG4708) of predicted queuosine-regulated ECF transporter	1

Substrate-specific component STY3230 of queuosine-regulated ECF transporter	1
tRNA-guanine transglycosylase (EC 2.4.2.29)	1
Ribonuclease H	4
hypothetical protein ssl1918	1
Ribonuclease HI (EC 3.1.26.4)	1
Ribonuclease HI, Vibrio paralog	1
Ribonuclease HI-related protein	1
Ribonuclease P archaeal and eukaryal	1
Ribonuclease P protein component 4 (EC 3.1.26.5)	1
RNA 3'-terminal phosphate cyclase	3
Protein RtcB	1
Protein with similarity to RtcB	1
Transcriptional regulatory protein RtcR	1
RNA processing and degradation, bacterial	7
3'-to-5' exoribonuclease RNase R	1
3'-to-5' oligoribonuclease (orn)	1
Cytoplasmic axial filament protein CafA and Ribonuclease G (EC 3.1.4.-)	1
Exoribonuclease II (EC 3.1.13.1)	1
FIG146085: 3'-to-5' oligoribonuclease A, Bacillus type	1
Ribonuclease E (EC 3.1.26.12)	1
Ribonuclease III (EC 3.1.26.3)	1
RNA pseudouridine syntheses	9
Ribosomal large subunit pseudouridine synthase A (EC 4.2.1.70)	1
Ribosomal large subunit pseudouridine synthase C (EC 4.2.1.70)	1
Ribosomal large subunit pseudouridine synthase D (EC 4.2.1.70)	1
Ribosomal large subunit pseudouridine synthase E (EC 4.2.1.70)	1
Ribosomal large subunit pseudouridine synthase F (EC 4.2.1.70)	1
Ribosomal small subunit pseudouridine synthase A (EC 4.2.1.70)	1
tRNA pseudouridine synthase A (EC 4.2.1.70)	1
tRNA pseudouridine synthase B (EC 4.2.1.70)	1
tRNA pseudouridine synthase C (EC 4.2.1.70)	1
rRNA modification Archaea	2
Dimethyladenosine transferase (EC 2.1.1.-)	1
tRNA:Cm32/Um32 methyltransferase	1
rRNA modification Bacteria	17
23S rRNA (guanine-N-2-) -methyltransferase rlmG (EC 2.1.1.-)	1
23S rRNA (guanosine-2'-O-) -methyltransferase rlmB (EC 2.1.1.-)	1

23S rRNA (Uracil-5-) -methyltransferase rumB (EC 2.1.1.-)	1
Dimethyladenosine transferase (EC 2.1.1.-)	1
Ribosomal large subunit pseudouridine synthase A (EC 4.2.1.70)	1
Ribosomal large subunit pseudouridine synthase C (EC 4.2.1.70)	1
Ribosomal large subunit pseudouridine synthase D (EC 4.2.1.70)	1
Ribosomal large subunit pseudouridine synthase F (EC 4.2.1.70)	1
Ribosomal RNA large subunit methyltransferase A (EC 2.1.1.51)	1
Ribosomal RNA large subunit methyltransferase F (EC 2.1.1.51)	1
Ribosomal RNA small subunit methyltransferase B (EC 2.1.1.-)	1
Ribosomal RNA small subunit methyltransferase C (EC 2.1.1.52)	1
Ribosomal RNA small subunit methyltransferase E (EC 2.1.1.-)	1
Ribosomal RNA small subunit methyltransferase F (EC 2.1.1.-)	1
Ribosomal small subunit pseudouridine synthase A (EC 4.2.1.70)	1
RNA binding methyltransferase FtsJ like	1
tRNA:m(5)U-54 MTase gid	1
tRNA modification Archaea	20
5-carboxymethyl uridine and 5-carboxymethyl 2-thiouridine methyltransferase	1
Archease	1
Asparaginyl-tRNA synthetase (EC 6.1.1.22)	1
COG1355, Predicted dioxygenase	1
Cysteine desulfurase (EC 2.8.1.7), IscS subfamily	1
Cysteine desulfurase (EC 2.8.1.7), SufS subfamily	1
GTP-binding protein Era	1
Iron-sulfur cluster assembly scaffold protein IscU	1
Protein RtcB	1
PUA-PAPS reductase like fusion	1
Thiamine biosynthesis protein thiI	1
tRNA (Guanine37-N1) -methyltransferase (EC 2.1.1.31)	1
tRNA (Uracil54-C5-)-methyltransferase (EC 2.1.1.35)	1
tRNA dihydrouridine synthase B (EC 1.-.-.-)	1
tRNA pseudouridine 13 synthase (EC 4.2.1.-)	1
tRNA pseudouridine synthase A (EC 4.2.1.70)	1
tRNA(Cytosine32)-2-thiocytidine synthetase	1
tRNA:Cm32/Um32 methyltransferase	1
tRNA-i(6)A37 methylthiotransferase	1
YrdC/Sua5 family protein, required for threonylcarbamoyladenosine (t(6)A) formation in tRNA	1
tRNA modification Bacteria	55

5-methylaminomethyl-2-thiouridine-forming enzyme mnmC	1
Believed to be involved in assembly of Fe-S clusters	1
Chaperone protein HscB	1
COG0613, Predicted metal-dependent phosphoesterases (PHP family)	1
Cysteine desulfurase (EC 2.8.1.7), IscS subfamily	1
Cysteine desulfurase (EC 2.8.1.7), SufS subfamily	1
Cytidine deaminase (EC 3.5.4.5)	1
FIG004453: protein YceG like	1
FIG137478: Hypothetical protein	1
glutamyl-Q-tRNA synthetase	1
GTP cyclohydrolase I (EC 3.5.4.16) type 1	1
GTPase and tRNA-U34 5-formylation enzyme TrmE	1
Iojap protein	1
Iron binding protein IscA for iron-sulfur cluster assembly	1
Iron-sulfur cluster assembly protein SufB	1
Iron-sulfur cluster assembly protein SufD	1
Iron-sulfur cluster assembly scaffold protein IscU	1
NADPH dependent preQ0 reductase	1
Putative tRNA-m1A22 methylase	1
Queuosine Biosynthesis QueC ATPase	1
Queuosine biosynthesis QueD, PTPS-I	1
Queuosine Biosynthesis QueE Radical SAM	1
Rhodanese-related sulfurtransferases	1
Ribosomal large subunit pseudouridine synthase A (EC 4.2.1.70)	1
RNA binding methyltransferase FtsJ like	1
S-adenosylmethionine:tRNA ribosyltransferase-isomerase (EC 5.-.-.-)	1
Thiamine biosynthesis protein thiI	1
tRNA (5-methoxyuridine) 34 synthase	1
tRNA (5-methylaminomethyl-2-thiouridylate)-methyltransferase (EC 2.1.1.61)	1
tRNA (cytosine34-2'-O-)-methyltransferase (EC 2.1.1.-)	1
tRNA (Guanine37-N1) -methyltransferase (EC 2.1.1.31)	1
tRNA (guanine46-N7-)-methyltransferase (EC 2.1.1.33)	1
tRNA (Guanosine18-2'-O-) -methyltransferase (EC 2.1.1.34)	1
tRNA (Uracil54-C5-)-methyltransferase (EC 2.1.1.35)	1
tRNA (uridine-5-oxyacetic acid methyl ester) 34 synthase	1
tRNA 5-methylaminomethyl-2-thiouridine synthase TusA	1
tRNA 5-methylaminomethyl-2-thiouridine synthase TusB	1

tRNA 5-methylaminomethyl-2-thiouridine synthase TusC	1
tRNA delta(2)-isopentenylpyrophosphate transferase (EC 2.5.1.8)	1
tRNA dihydrouridine synthase A (EC 1.-.-.-)	1
tRNA dihydrouridine synthase B (EC 1.-.-.-)	1
tRNA pseudouridine 13 synthase (EC 4.2.1.-)	1
tRNA pseudouridine synthase A (EC 4.2.1.70)	1
tRNA pseudouridine synthase B (EC 4.2.1.70)	1
tRNA pseudouridine synthase C (EC 4.2.1.70)	1
tRNA uridine 5-carboxymethylaminomethyl modification enzyme GidA	1
tRNA(Cytosine32)-2-thiocytidine synthetase	1
tRNA(Ile)-lysidine synthetase	1
tRNA-(ms[2]io[6]A)-hydroxylase (EC 1.-.-.-)	1
tRNA:Cm32/Um32 methyltransferase	1
tRNA:m(5)U-54 MTase gid	1
tRNA-dihydrouridine synthase C (EC 1.-.-.-)	1
tRNA-guanine transglycosylase (EC 2.4.2.29)	1
tRNA-i(6)A37 methylthiotransferase	1
YrdC/Sua5 family protein, required for threonylcarbamoyladenosine (t(6)A) formation in tRNA	1
tRNA modification yeast cytoplasmic	8
Sua5 YciO YrdC YwlC family protein	1
tRNA (Guanine37-N1) -methyltransferase (EC 2.1.1.31)	1
tRNA (Guanosine18-2'-O-) -methyltransferase (EC 2.1.1.34)	1
tRNA (Uracil54-C5-)-methyltransferase (EC 2.1.1.35)	1
tRNA delta(2)-isopentenylpyrophosphate transferase (EC 2.5.1.8)	1
tRNA N2,N2-dimethyl(Guanine26-N2)-methyltransferase (EC 2.1.1.32)	1
tRNA pseudouridine 13 synthase (EC 4.2.1.-)	1
tRNA pseudouridine synthase A (EC 4.2.1.70)	1
tRNA modification yeast mitochondrial	9
GTPase and tRNA-U34 5-formylation enzyme TrmE	1
Sua5 YciO YrdC YwlC family protein	1
tRNA (5-methylaminomethyl-2-thiouridylate)-methyltransferase (EC 2.1.1.61)	1
tRNA (Guanine37-N1) -methyltransferase (EC 2.1.1.31)	1
tRNA (Uracil54-C5-)-methyltransferase (EC 2.1.1.35)	1
tRNA delta(2)-isopentenylpyrophosphate transferase (EC 2.5.1.8)	1
tRNA N2,N2-dimethyl(Guanine26-N2)-methyltransferase (EC 2.1.1.32)	1
tRNA pseudouridine synthase A (EC 4.2.1.70)	1
tRNA uridine 5-carboxymethylaminomethyl modification enzyme GidA	1

tRNA nucleotidyltransferase	2
tRNA nucleotidyltransferase (EC 2.7.7.21) (EC 2.7.7.25)	1
tRNA nucleotidyltransferase related protein MMP0420	1
tRNA processing	12
5-carboxymethyl uridine and 5-carboxymethyl 2-thiouridine methyltransferase	1
Ribonuclease D (EC 3.1.26.3)	1
Ribonuclease P protein component (EC 3.1.26.5)	1
Ribonuclease PH (EC 2.7.7.56)	1
Ribonuclease T (EC 3.1.13.-)	1
Ribonuclease Z (EC 3.1.26.11)	1
tRNA delta(2)-isopentenylpyrophosphate transferase (EC 2.5.1.8)	1
tRNA pseudouridine 13 synthase (EC 4.2.1.-)	1
tRNA pseudouridine synthase A (EC 4.2.1.70)	1
tRNA pseudouridine synthase B (EC 4.2.1.70)	1
tRNA(Ile)-lysidine synthetase	1
tRNA-i(6)A37 methylthiotransferase	1
tRNA splicing	1
RNA:NAD 2'-phosphotransferase	1
Wyeosine-MimG Biosynthesis	3
Iron-sulfur cluster assembly scaffold protein IscU	1
Thioredoxin reductase (EC 1.8.1.9)	1
tRNA (Guanine37-N1) -methyltransferase (EC 2.1.1.31)	1
YrdC-YciO	4
COG0613, Predicted metal-dependent phosphoesterases (PHP family)	1
YciO family	1
YgjD/Kae1/Qri7 family, required for threonylcarbamoyladenosine (t(6)A) formation in tRNA	1
YrdC/Sua5 family protein, required for threonylcarbamoyladenosine (t(6)A) formation in tRNA	1
**Transcription**	**28**
RNA polymerase archaeal	2
DNA-directed RNA polymerase subunit A' (EC 2.7.7.6)	1
DNA-directed RNA polymerase subunit B'' (EC 2.7.7.6)	1
RNA polymerase archaeal initiation factors	1
Transcription initiation factor B	1
RNA polymerase bacterial	2
DNA-directed RNA polymerase beta subunit (EC 2.7.7.6)	1
DNA-directed RNA polymerase beta' subunit (EC 2.7.7.6)	1
RNA polymerase III	1

DNA-directed RNA polymerase III largest subunit (EC 2.7.7.6)	1
Rrf2 family transcriptional regulators	4
Nitrite-sensitive transcriptional repressor NsrR	1
Predicted transcriptional regulator of cysteine synthase, Rrf2 family	1
Rrf2 family transcriptional regulator	1
Rrf2 family transcriptional regulator, group III	1
Transcription factors bacterial	9
Regulator of nucleoside diphosphate kinase	1
Rho-specific inhibitor of transcription termination (YaeO)	1
Transcription antitermination protein NusG	1
Transcription elongation factor GreA	1
Transcription elongation factor GreB	1
Transcription termination factor Rho	1
Transcription termination protein NusB	1
Transcriptional activator RfaH	1
Transcription-repair coupling factor	1
Transcription initiation, bacterial sigma factors	9
RNA polymerase sigma factor RpoE	1
RNA polymerase sigma factor RpoH	1
RNA polymerase sigma factor RpoS	1
RNA polymerase sigma factor SigB	1
RNA polymerase sigma factor SigW	1
RNA polymerase sigma-54 factor RpoN	1
RNA polymerase sigma-70 factor	1
Serine protease precursor MucD/AlgY associated with sigma factor RpoE	1
Sigma factor RpoE negative regulatory protein RseA	1
**Secondary Metabolism**	**13**
**Bacterial cytostatics, differentiation factors and antibiotics**	**3**
Paerucumarin Biosynthesis	2
HTH-type transcriptional regulator PtxR	1
PvcB protein, related to amino acid oxidizing enzymes	1
Phenazine biosynthesis	1
Phenazine biosynthesis protein PhzF	1
**Biologically active compounds in metazoan cell defence and differentiation**	**2**
Steroid sulfates	2
Arylsulfatase (EC 3.1.6.1)	1
Steryl-sulfatase precursor (EC 3.1.6.2)	1

**Biosynthesis of phenylpropanoids**	**2**
Apigenin derivatives	1
Isoflavone reductase homolog P3 (EC 1.3.1.-)	1
Phytoalexin biosynthesis	1
Isoflavone reductase homolog P3 (EC 1.3.1.-)	1
**Plant Hormones**	**6**
Auxin biosynthesis	5
Anthranilate phosphoribosyltransferase (EC 2.4.2.18)	1
Aromatic-L-amino-acid decarboxylase (EC 4.1.1.28)	1
Phosphoribosylanthranilate isomerase (EC 5.3.1.24)	1
Tryptophan synthase alpha chain (EC 4.2.1.20)	1
Tryptophan synthase beta chain (EC 4.2.1.20)	1
Auxin degradation	1
Peroxidase (EC 1.11.1.7)	1
**Stress Response**	**177**
**Acid stress**	**3**
Acid resistance mechanisms	1
Probable glutamate/gamma-aminobutyrate antiporter	1
Glutamate transporter involved in acid tolerance in Streptococcus	2
Glutamate transport ATP-binding protein	1
Glutamate transport membrane-spanning protein	1
**Bacterial hemoglobins**	**2**
Bacterial hemoglobins	2
diguanylate cyclase/phosphodiesterase (GGDEF & EAL domains) with PAS/PAC sensor(s)	1
Hemoglobin-like protein HbO	1
**Carbon Starvation**	**6**
Carbon Starvation	6
Carbon starvation protein A	1
Carbon starvation protein A paralog	1
Carbon storage regulator	1
Starvation lipoprotein Slp paralog	1
Starvation sensing protein RspA	1
Stringent starvation protein B	1
**Cold shock**	**5**
Cold shock, CspA family of proteins	5
Cold shock protein CspA	1
Cold shock protein CspC	1

Cold shock protein CspD	1
Cold shock protein CspE	1
Cold shock protein CspG	1
**Commensurate regulon activation**	**1**
Commensurate regulon activation	1
Right origin-binding protein	1
**Detoxification**	**22**
D-tyrosyl-tRNA(Tyr) deacylase	1
D-tyrosyl-tRNA(Tyr) deacylase	1
Housecleaning nucleoside triphosphate pyrophosphatases	6
5-nucleotidase SurE (EC 3.1.3.5)	1
5'-nucleotidase YjjG (EC 3.1.3.5)	1
Cof protein, HD superfamily hydrolase	1
Deoxyuridine 5'-triphosphate nucleotidohydrolase (EC 3.6.1.23)	1
Nucleoside 5-triphosphatase RdgB (dHAPTP, dITP, XTP-specific) (EC 3.6.1.15)	1
Nucleotidase YfbR, HD superfamily	1
Nucleoside triphosphate pyrophosphohydrolase MazG	1
MazG-related protein	1
Nudix proteins (nucleoside triphosphate hydrolases)	8
Adenosine (5')-pentaphospho-(5'')-adenosine pyrophosphohydrolase (EC 3.6.1.-)	1
ADP compounds hydrolase NudE (EC 3.6.1.-)	1
ADP-ribose pyrophosphatase (EC 3.6.1.13)	1
ADP-ribose pyrophosphatase, mitochondrial precursor (EC 3.6.1.13)	1
Deoxyuridine 5'-triphosphate nucleotidohydrolase (EC 3.6.1.23)	1
Hypothetical nudix hydrolase YeaB	1
Mutator mutT protein (7,8-dihydro-8-oxoguanine-triphosphatase) (EC 3.6.1.-)	1
Nudix hydrolase family protein YffH	1
Tellurite resistance: Chromosomal determinants	2
FIG005189: putative transferase clustered with tellurite resistance proteins TehA/TehB	1
Tellurite resistance protein TehB	1
Uptake of selenate and selenite	4
DedA protein	1
Sulfate and thiosulfate import ATP-binding protein CysA (EC 3.6.3.25)	1
Various polyols ABC transporter, periplasmic substrate-binding protein	1
Various polyols ABC transporter, permease component 2	1
**Dimethylarginine metabolism**	**1**
Dimethylarginine metabolism	1

Ornithine aminotransferase (EC 2.6.1.13)	1
**Flavohaemoglobin**	**1**
Flavohaemoglobin	1
Nitric-oxide reductase (EC 1.7.99.7), quinol-dependent	1
**Heat shock**	**16**
Heat shock dnaK gene cluster extended	16
Chaperone protein DnaK	1
DNA replication intiation control protein YabA	1
FIG001341: Probable Fe(2+)-trafficking protein YggX	1
FIG009886: phosphoesterase	1
Heat shock protein GrpE	1
Heat-inducible transcription repressor HrcA	1
HspR, transcriptional repressor of DnaK operon	1
Hypothetical radical SAM family enzyme, NOT coproporphyrinogen III oxidase, oxygen-independent	1
MiaB family protein, possibly involved in tRNA or rRNA modification	1
Nucleoside 5-triphosphatase RdgB (dHAPTP, dITP, XTP-specific) (EC 3.6.1.15)	1
Putative coproporphyrinogen III oxidase of BS HemN-type, oxygen-independent (EC 1.3.99.22), in heat shock gene cluster	1
Ribonuclease PH (EC 2.7.7.56)	1
Ribosomal RNA small subunit methyltransferase E (EC 2.1.1.-)	1
RNA polymerase sigma factor RpoH	1
tmRNA-binding protein SmpB	1
Translation elongation factor LepA	1
**Hfl operon**	**3**
Hfl operon	3
HflC protein	1
HflK protein	1
RNA-binding protein Hfq	1
**Osmotic stress**	**29**
Choline and Betaine Uptake and Betaine Biosynthesis	14
Betaine aldehyde dehydrogenase (EC 1.2.1.8)	1
Choline binding protein A	1
Choline dehydrogenase (EC 1.1.99.1)	1
Glycine betaine ABC transport system permease protein	1
Glycine betaine ABC transport system, ATP-binding protein OpuAA (EC 3.6.3.32)	1
Glycine betaine ABC transport system, permease/glycine betaine-binding protein OpuABC	1
HTH-type transcriptional regulator BetI	1
L-proline glycine betaine ABC transport system permease protein ProV (TC 3.A.1.12.1)	1

L-proline glycine betaine ABC transport system permease protein ProW (TC 3.A.1.12.1)	1
L-proline glycine betaine binding ABC transporter protein ProX (TC 3.A.1.12.1)	1
Osmotically activated L-carnitine/choline ABC transporter, substrate-binding protein OpuCC	1
Sarcosine oxidase alpha subunit (EC 1.5.3.1)	1
Sarcosine oxidase beta subunit (EC 1.5.3.1)	1
Sarcosine oxidase delta subunit (EC 1.5.3.1)	1
Ectoine biosynthesis and regulation	3
L-2,4-diaminobutyric acid acetyltransferase (EC 2.3.1.-)	1
L-ectoine synthase (EC 4.2.1.-)	1
Putative regulatory protein associated with the ectoine operon	1
Osmoprotectant ABC transporter YehZYXW of Enterobacteriales	2
Osmoprotectant ABC transporter inner membrane protein YehW	1
Osmoprotectant ABC transporter permease protein YehY	1
Osmoregulation	2
Glycerol uptake facilitator protein	1
Outer membrane protein A precursor	1
Osmotic stress cluster	1
Heat shock (predicted periplasmic) protein YciM, precursor	1
Synthesis of osmoregulated periplasmic glucans	7
Beta-(1-->2)glucan export ATP-binding/permease protein NdvA (EC 3.6.3.42)	1
Cyclic beta-1,2-glucan modification transmembrane protein	1
Glucans biosynthesis glucosyltransferase H (EC 2.4.1.-)	1
Glucans biosynthesis protein C (EC 2.1.-.-)	1
Glucans biosynthesis protein G precursor	1
OpgC protein	1
Phosphoglycerol transferase I (EC 2.7.8.20)	1
**Oxidative stress**	**64**
CoA disulfide thiol-disulfide redox system	1
CoA-disulfide reductase (EC 1.8.1.14)	1
Glutaredoxins	6
Glutaredoxin	1
Glutaredoxin 1	1
Glutaredoxin 2	1
Glutaredoxin 3	1
Glutaredoxin 3 (Grx3)	1
Glutaredoxin-related protein	1
Glutathione analogs: mycothiol	2

Glycosyltransferase MshA involved in mycothiol biosynthesis (EC 2.4.1.-)	1
N-acetyl-1-D-myo-inosityl-2-amino-2-deoxy-alpha-D-glucopyranoside deacetylase MshB	1
Glutathione: Biosynthesis and gamma-glutamyl cycle	4
Gamma-glutamyltranspeptidase (EC 2.3.2.2)	1
Glutamate--cysteine ligase (EC 6.3.2.2)	1
Glutathione biosynthesis bifunctional protein gshF (EC 6.3.2.2)(EC 6.3.2.3)	1
Similar to 5-oxoprolinase (EC 3.5.2.9) and Methylhydantoinases A, B (EC 3.5.2.14), contradiction in experimental data (see Annotation)	1
Glutathione: Non-redox reactions	6
FIG005121: SAM-dependent methyltransferase (EC 2.1.1.-)	1
Glutathione S-transferase family protein	1
Glutathione S-transferase, omega (EC 2.5.1.18)	1
Glutathione S-transferase, unnamed subgroup (EC 2.5.1.18)	1
SAM-dependent methyltransferase 2, in cluster with Hydroxyacylglutathione hydrolase (EC 3.1.2.6)	1
Uncharacterized glutathione S-transferase-like protein	1
Glutathione: Redox cycle	7
Glutaredoxin	1
Glutaredoxin 1	1
Glutaredoxin 2	1
Glutaredoxin 3	1
Glutaredoxin 3 (Grx3)	1
Glutathione peroxidase (EC 1.11.1.9)	1
Glutathione reductase (EC 1.8.1.7)	1
NADPH:quinone oxidoreductase 2	3
NADPH:quinone oxidoreductase 2	1
Redox-sensing transcriptional regulator QorR	1
Redox-sensing transcriptional regulator QorR, putative	1
Oxidative stress	19
bacteriophytochrome heme oxygenase BphO	1
Catalase (EC 1.11.1.6)	1
Ferric uptake regulation protein FUR	1
Fumarate and nitrate reduction regulatory protein	1
Heme oxygenase HemO, associated with heme uptake	1
Hydrogen peroxide-inducible genes activator	1
Manganese superoxide dismutase (EC 1.15.1.1)	1
Nitrite-sensitive transcriptional repressor NsrR	1
Organic hydroperoxide resistance protein	1
Organic hydroperoxide resistance transcriptional regulator	1

Paraquat-inducible protein A	1
Paraquat-inducible protein B	1
Peroxidase (EC 1.11.1.7)	1
Peroxide stress regulator PerR, FUR family	1
Phytochrome, two-component sensor histidine kinase (EC 2.7.3.-)	1
Redox-sensitive transcriptional activator SoxR	1
Rubrerythrin	1
Superoxide dismutase [Fe] (EC 1.15.1.1)	1
Zinc uptake regulation protein ZUR	1
Protection from Reactive Oxygen Species	5
Catalase (EC 1.11.1.6)	1
Cytochrome c551 peroxidase (EC 1.11.1.5)	1
Manganese superoxide dismutase (EC 1.15.1.1)	1
Peroxidase (EC 1.11.1.7)	1
Superoxide dismutase [Fe] (EC 1.15.1.1)	1
Redox-dependent regulation of nucleus processes	4
NAD-dependent glyceraldehyde-3-phosphate dehydrogenase (EC 1.2.1.12)	1
NADPH-dependent glyceraldehyde-3-phosphate dehydrogenase (EC 1.2.1.13)	1
Nicotinamidase (EC 3.5.1.19)	1
Nicotinate phosphoribosyltransferase (EC 2.4.2.11)	1
Regulation of Oxidative Stress Response	5
Aerobic respiration control protein arcA	1
Aerobic respiration control sensor protein arcB (EC 2.7.3.-)	1
Peroxidase (EC 1.11.1.7)	1
Redox-sensitive transcriptional activator SoxR	1
Superoxide dismutase [Fe] (EC 1.15.1.1)	1
Rubrerythrin	2
Rubredoxin-NAD(+) reductase (EC 1.18.1.1)	1
Rubrerythrin	1
**Periplasmic Stress**	**6**
Periplasmic Stress Response	6
HtrA protease/chaperone protein	1
Outer membrane protein H precursor	1
Outer membrane stress sensor protease DegQ, serine protease	1
Outer membrane stress sensor protease DegS	1
Sigma factor RpoE negative regulatory protein RseA	1
Survival protein SurA precursor (Peptidyl-prolyl cis-trans isomerase SurA) (EC 5.2.1.8)	1

**Phage shock protein (psp) operon**	**4**
Phage shock protein (psp) operon	4
Phage shock protein A	1
Phage shock protein B	1
Phage shock protein C	1
Phage shock protein E	1
**SigmaB stress responce regulation**	**6**
SigmaB stress responce regulation	6
Putative SigmaB asociated two-component system sensor protein	1
RNA polymerase sigma factor SigB	1
RsbR, positive regulator of sigma-B	1
RsbS, negative regulator of sigma-B	1
Serine phosphatase RsbU, regulator of sigma subunit	1
Serine-protein kinase rsbW (EC 2.7.11.1)	1
**Sugar-phosphate stress regulation**	**1**
Sugar-phosphate stress regulation	1
SgrR, sugar-phosphate stress, transcriptional activator of SgrS small RNA	1
**Universal stress protein family**	**7**
Universal stress protein family	7
Universal stress protein A	1
Universal stress protein B	1
Universal stress protein E	1
Universal stress protein family	1
Universal stress protein family 1	1
Universal stress protein family 4	1
Universal stress protein family COG0589	1
**Sulfur Metabolism**	**43**
**Galactosylceramide and Sulfatide metabolism**	**4**
Galactosylceramide and Sulfatide metabolism	4
Alpha-galactosidase (EC 3.2.1.22)	1
Arylsulfatase (EC 3.1.6.1)	1
Beta-galactosidase (EC 3.2.1.23)	1
Neuraminidase NanP	1
**Inorganic sulfur assimilation**	**17**
Inorganic Sulfur Assimilation	17
4Fe-4S ferredoxin, iron-sulfur binding	1
ABC-type probable sulfate transporter, periplasmic binding protein	1

Ferredoxin	1
Ferredoxin--NADP(+) reductase (EC 1.18.1.2)	1
Phosphoadenylyl-sulfate reductase [thioredoxin] (EC 1.8.4.8)	1
Putative sulfate permease	1
Sulfate adenylyltransferase subunit 1 (EC 2.7.7.4)	1
Sulfate adenylyltransferase subunit 2 (EC 2.7.7.4)	1
Sulfate and thiosulfate binding protein CysP	1
Sulfate and thiosulfate import ATP-binding protein CysA (EC 3.6.3.25)	1
Sulfate permease, Pit-type	1
Sulfate transport system permease protein CysT	1
Sulfate transport system permease protein CysW	1
Sulfate transporter, CysZ-type	1
Sulfate-binding protein Sbp	1
Sulfite reductase [NADPH] flavoprotein alpha-component (EC 1.8.1.2)	1
Sulfite reductase [NADPH] hemoprotein beta-component (EC 1.8.1.2)	1
**Organic sulfur assimilation**	**11**
Alkanesulfonate assimilation	4
ABC-type nitrate/sulfonate/bicarbonate transport system, permease component	1
Alkanesulfonate monooxygenase (EC 1.14.14.5)	1
Alkanesulfonates transport system permease protein	1
Arylsulfatase (EC 3.1.6.1)	1
Alkanesulfonates Utilization	2
Alkanesulfonate monooxygenase (EC 1.14.14.5)	1
Alkanesulfonates transport system permease protein	1
L-Cystine Uptake and Metabolism	1
Cystathionine gamma-synthase (EC 2.5.1.48)	1
Taurine Utilization	1
Taurine transport system permease protein TauC	1
Utilization of glutathione as a sulphur source	3
Gamma-glutamyltranspeptidase (EC 2.3.2.2)	1
Putative glutathione transporter, permease component	1
Putative gluthatione transporter,solute-binding component	1
**Release of Dimethyl Sulfide (DMS) from Dimethylsulfoniopropionate (DMSP)**	**1**
Release of Dimethyl Sulfide (DMS) from Dimethylsulfoniopropionate (DMSP)	1
Dimethylsulfoniopropionate (DMSP) acyl CoA transferase DddD	1
**Sulfate reduction-associated complexes**	**2**
Sulfate reduction-associated complexes	2

Sulfite reduction-associated complex DsrMKJOP protein DsrK (=HmeD)	1
tRNA 2-thiouridine synthesizing protein E (EC 2.8.1.-)	1
**Sulfur oxidation**	**3**
Sulfur oxidation	3
Cytochrome c-type biogenesis protein CcdA (DsbD analog)	1
Lipocalin-related protein and Bos/Can/Equ allergen	1
Sulfite dehydrogenase cytochrome subunit SoxD	1
**Thioredoxin-disulfide reductase**	**5**
Thioredoxin-disulfide reductase	5
Alkyl hydroperoxide reductase protein F (EC 1.6.4.-)	1
Alkylhydroperoxidase protein D	1
Hydrogen peroxide-inducible genes activator	1
Thiol peroxidase, Tpx-type (EC 1.11.1.15)	1
Thioredoxin reductase (EC 1.8.1.9)	1
**Virulence, Disease and Defense**	**156**
**Adhesion**	**10**
Accessory colonization factor	4
Accessory colonization factor AcfA	1
Accessory colonization factor AcfB	1
Accessory colonization factor AcfC	1
Accessory colonization factor AcfD precursor	1
Adhesins in Staphylococcus	3
Extracellular ECM and plasma binding protein Emp	1
Predicted cell-wall-anchored protein SasA (LPXTG motif)	1
Predicted cell-wall-anchored protein SasF (LPXAG motif)	1
Mediator of hyperadherence YidE in Enterobacteria and its conserved region	1
16 kDa heat shock protein A	1
Streptococcus pyogenes recombinatorial zone	1
Fibronectin-binding protein	1
Type 1 pili (mannose-sensitive fimbriae)	1
type 1 fimbriae anchoring protein FimD	1
**Bacterial cyanide production and tolerance mechanisms**	**3**
Bacterial cyanide production and tolerance mechanisms	3
Carbonic anhydrase (EC 4.2.1.1)	1
formate dehydrogenase formation protein FdhE	1
Thiosulfate sulfurtransferase, rhodanese (EC 2.8.1.1)	1
**Bacteriocins, ribosomally synthesized antibacterial peptides**	**14**

Bacitracin Stress Response	4
ABC transporter permease protein YvcS	1
Bacitracin export permease protein BceB	1
Protein LiaI	1
Two-component response regulator BceR	1
Bacteriocin-like peptides Blp	1
Lactacin F ABC transporter permease component	1
Colicin V and Bacteriocin Production Cluster	6
Acetyl-coenzyme A carboxyl transferase beta chain (EC 6.4.1.2)	1
Bacteriocin production protein	1
Colicin V production protein	1
DedA protein	1
DedD protein	1
tRNA pseudouridine synthase A (EC 4.2.1.70)	1
Marinocine, a broad-spectrum antibacterial protein	1
Dehydrogenase flavoprotein LodB	1
Tolerance to colicin E2	2
Two-component response regulator CreB	1
Two-component response regulator CreC	1
**C jejuni colonization of chick caeca**	**9**
C jejuni colonization of chick caeca	9
4-keto-6-deoxy-N-Acetyl-D-hexosaminyl-(Lipid carrier) aminotransferase	1
Alpha-1,4-N-acetylgalactosamine transferase PglH (EC 2.4.1.-)	1
Flagellar hook-associated protein FlgK	1
Flagellar motor rotation protein MotA	1
Flagellar motor rotation protein MotB	1
Phosphate acetyltransferase (EC 2.3.1.8)	1
RNA polymerase sigma-54 factor RpoN	1
Rrf2 family transcriptional regulator	1
UDP-N-acetylglucosamine 4,6-dehydratase (EC 4.2.1.-)	1
**Detection**	**5**
MLST	5
Carbamate kinase (EC 2.7.2.2)	1
Glycerol kinase (EC 2.7.1.30)	1
Guanylate kinase (EC 2.7.4.8)	1
Phosphate acetyltransferase (EC 2.3.1.8)	1
Triosephosphate isomerase (EC 5.3.1.1)	1

**Fimbriae of the Chaperone/Usher Assembly Pathway**	**3**
α-Fimbriae	1
Alpha-fimbriae tip adhesin	1
σ-Fimbriae	2
Sigma-fimbriae uncharacterized paralogous subunit	1
Sigma-fimbriae usher protein	1
**Invasion and intracellular resistance**	**9**
Cytolysin and Lipase operon in Vibrio	3
Cytolysin and hemolysin, HlyA, Pore-forming toxin	1
Lipase activator protein, Lipase-specific foldase	1
Metalloprotease, putative zinc-binding domain	1
Gram-Positive Extracellular Nucleases	2
DNA-entry nuclease (Competence-specific nuclease) (EC 3.1.30.-)	1
Streptodornase D	1
Listeria surface proteins: Internalin-like proteins	3
Internalin A (LPXTG motif)	1
internalin, putative (LPXTG motif)	1
Internalin-like protein (LPXTG motif) Lmo2026 homolog	1
Listeria surface proteins: LPXTG motif	1
Putative peptidoglycan bound protein (LPXTG motif) Lmo0160 homolog	1
**Resistance to antibiotics and toxic compounds**	**78**
Aminoglycoside adenylyltransferases	1
Spectinomycin 9-O-adenylyltransferase	1
Arsenic resistance	4
Arsenate reductase (EC 1.20.4.1)	1
Arsenic efflux pump protein	1
Arsenical pump-driving ATPase (EC 3.6.3.16)	1
Arsenical-resistance protein ACR3	1
Beta-lactamase	6
Beta-lactamase	1
Beta-lactamase (Cephalosporinase) (EC 3.5.2.6)	1
Beta-lactamase (EC 3.5.2.6)	1
Beta-lactamase class A	1
Beta-lactamase class C and other penicillin binding proteins	1
Metal-dependent hydrolases of the beta-lactamase superfamily III	1
BlaR1 Family Regulatory Sensor-transducer Disambiguation	3
Beta-lactamase (EC 3.5.2.6)	1

Beta-lactamase class A	1
Copper-translocating P-type ATPase (EC 3.6.3.4)	1
Cobalt-zinc-cadmium resistance	9
Cobalt/zinc/cadmium efflux RND transporter, membrane fusion protein, CzcB family	1
Cobalt-zinc-cadmium resistance protein	1
Cobalt-zinc-cadmium resistance protein CzcD	1
Copper-sensing two-component system response regulator CusR	1
DNA-binding heavy metal response regulator	1
Heavy metal RND efflux outer membrane protein, CzcC family	1
Probable Co/Zn/Cd efflux system membrane fusion protein	1
Transcriptional regulator, MerR family	1
Zinc transporter ZitB	1
Copper homeostasis	11
CopG protein	1
Copper resistance protein B	1
Copper resistance protein D	1
Copper tolerance protein	1
Copper-sensing two-component system response regulator CusR	1
Copper-translocating P-type ATPase (EC 3.6.3.4)	1
Cu(I)-responsive transcriptional regulator	1
Cytochrome c heme lyase subunit CcmF	1
Cytochrome c heme lyase subunit CcmH	1
Multicopper oxidase	1
Sensor protein copS (EC 2.7.3.-)	1
Copper homeostasis: copper tolerance	3
Cytoplasmic copper homeostasis protein cutC	1
Magnesium and cobalt efflux protein CorC	1
Membrane protein, suppressor for copper-sensitivity ScsB	1
Erythromycin resistance	1
Dimethyladenosine transferase (EC 2.1.1.-)	1
Mercuric reductase	1
Mercuric ion reductase (EC 1.16.1.1)	1
Mercury resistance operon	1
Mercuric ion reductase (EC 1.16.1.1)	1
Methicillin resistance in Staphylococci	6
D-alanine--poly(phosphoribitol) ligase subunit 1 (EC 6.1.1.13)	1
D-alanyl transfer protein DltB	1

Poly(glycerophosphate chain) D-alanine transfer protein DltD	1
RNA polymerase sigma factor SigB	1
UDP-N-acetylmuramoylalanyl-D-glutamate--2,6-diaminopimelate ligase (EC 6.3.2.13)	1
Undecaprenyl-phosphate N-acetylglucosaminyl 1-phosphate transferase (EC 2.7.8.-)	1
Multidrug efflux pump in Campylobacter jejuni (CmeABC operon)	3
RND efflux system, inner membrane transporter CmeB	1
RND efflux system, membrane fusion protein CmeA	1
RND efflux system, outer membrane lipoprotein CmeC	1
Multidrug Resistance Efflux Pumps	12
Acriflavin resistance protein	1
Macrolide-specific efflux protein MacA	1
Membrane fusion protein of RND family multidrug efflux pump	1
Multidrug efflux pump component MtrF	1
Multidrug-efflux transporter, major facilitator superfamily (MFS) (TC 2.A.1)	1
RND efflux system, inner membrane transporter CmeB	1
RND efflux system, membrane fusion protein CmeA	1
RND efflux system, outer membrane lipoprotein CmeC	1
RND efflux system, outer membrane lipoprotein, NodT family	1
Transcription regulator of multidrug efflux pump operon, TetR (AcrR) family	1
Transcription repressor of multidrug efflux pump acrAB operon, TetR (AcrR) family	1
Type I secretion outer membrane protein, TolC precursor	1
Multidrug Resistance Operon mdtRP of Bacillus	1
Multidrug efflux transporter MdtP	1
Multidrug Resistance, 2-protein version Found in Gram-positive bacteria	1
Membrane component of multidrug resistance system	1
Multidrug Resistance, Tripartite Systems Found in Gram Negative Bacteria	3
Inner membrane component of tripartite multidrug resistance system	1
Membrane fusion component of tripartite multidrug resistance system	1
Outer membrane component of tripartite multidrug resistance system	1
Resistance to fluoroquinolones	3
DNA gyrase subunit A (EC 5.99.1.3)	1
DNA gyrase subunit B (EC 5.99.1.3)	1
Topoisomerase IV subunit A (EC 5.99.1.-)	1
Resistance to Vancomycin	1
Vancomycin response regulator VanR	1
Streptococcus pneumoniae Vancomycin Tolerance Locus	1
Sensor histidine kinase VncS	1

Tetracycline resistance, ribosome protection type	2
Ribosome protection-type tetracycline resistance related proteins, group 2	1
Translation elongation factor G	1
The mdtABCD multidrug resistance cluster	3
Multidrug transporter MdtB	1
Multidrug transporter MdtD	1
Sensory histidine kinase BaeS	1
Zinc resistance	2
Response regulator of zinc sigma-54-dependent two-component system	1
Sensor protein of zinc sigma-54-dependent two-component system	1
**Streptococcus agalactiae virulome**	**1**
Streptococcus agalactiae virulome	1
Tyrosine-protein kinase transmembrane modulator EpsC	1
**Streptococcus pyogenes Virulome**	**4**
Streptococcus pyogenes Virulome	4
D-alanine--poly(phosphoribitol) ligase subunit 1 (EC 6.1.1.13)	1
Fibronectin-binding protein	1
Hyaluronate lyase precursor (EC 4.2.2.1)	1
Streptodornase D	1
**Toxins and superantigens**	**7**
Cholera toxin	5
Enterotoxin, A subunit (NAD(+)--diphthamide ADP- ribosyltransferase) (EC 2.4.2.36)	1
Enterotoxin, B subunit	1
Transcriptional activator ToxR	1
Transmembrane regulatory protein ToxS	1
Zona occludens toxin	1
Prophage-encoded Exotoxins	1
Streptodornase D	1
Streptolysin S Biosynthesis and Transport	1
Streptolysin S export transmembrane permease (SagH)	1
**Type III, Type IV, Type VI, ESAT secretion systems**	**13**
Type 4 secretion and conjugative transfer	13
ATPase provides energy for both assembly of type IV secretion complex and secretion of T-DNA complex (VirB4)	1
Conjugative transfer protein PSLT087	1
Conjugative transfer protein TrbB	1
Conjugative transfer protein TrbD	1
Conjugative transfer protein TrbI	1

Conjugative transfer protein TrbL	1
IncF plasmid conjugative transfer DNA-nicking and unwinding protein TraI	1
IncF plasmid conjugative transfer pilus assembly protein TraE	1
IncF plasmid conjugative transfer pilus assembly protein TraH	1
IncF plasmid conjugative transfer protein TraD	1
IncF plasmid conjugative transfer protein TrbC	1
IncQ plasmid conjugative transfer DNA nicking endonuclease TraR (pTi VirD2 homolog)	1
Integral inner membrane protein of type IV secretion complex (VirB6)	1
**Total**	**3877**
