# Supplementary material for: Genome-Wide Study of the Defective Sucrose Fermenter Strain of Vibrio cholerae from the Latin American Cholera Epidemic
Source: PLoS One. 2012 May 25;7(5):e37283. doi: 10.1371/journal.pone.0037283 (PMC3360680; doi:10.1371/journal.pone.0037283)
Supplement: Table S4 — Predicted functions of the hypothetical proteins of the IEC224 V. cholerae strain. (PDF) [file pone.0037283.s006.pdf]

Genome-Wide Study of the Defective Sucrose Fermenter Strain of *Vibrio cholerae* from the Latin American Cholera Epidemic

(Garza DR, Thompson CC, Loureiro ECB, Dutilh BE, Inada DT, et al.)

Table S4

Predicted Functions of the Hypothetical Proteins of the IEC224 *Vibrio cholerae* Strain

**Amino Acids and Derivatives**	**83**
**Alanine, serine, and glycine**	**6**
Glycine and Serine Utilization	4
D-3-phosphoglycerate dehydrogenase (EC 1.1.1.95)	1
D-serine/D-alanine/glycine transporter	1
Glycerate kinase (EC 2.7.1.31)	1

Glycine dehydrogenase [decarboxylating] (glycine cleavage system P protein) (EC 1.4.4.2)	1
Glycine cleavage system	1
Glycine dehydrogenase [decarboxylating] (glycine cleavage system P protein) (EC 1.4.4.2)	1
Serine Biosynthesis	1
D-3-phosphoglycerate dehydrogenase (EC 1.1.1.95)	1
**Arginine; urea cycle, polyamines**	**10**
Arginine and Ornithine Degradation	2
Ornithine cyclodeaminase (EC 4.3.1.12)	1
Succinylglutamic semialdehyde dehydrogenase (EC 1.2.1.71)	1
Arginine Biosynthesis extended	2
Acetylornithine deacetylase (EC 3.5.1.16)	1
N-succinyl-L,L-diaminopimelate desuccinylase (EC 3.5.1.18)	1
Polyamine Metabolism	2
Spermidine Putrescine ABC transporter permease component PotB (TC 3.A.1.11.1)	1
Spermidine Putrescine ABC transporter permease component potC (TC_3.A.1.11.1)	1
Putrescine utilization pathways	1
Glycine/D-amino acid oxidase (deaminating) in putrescine utilization cluster	1
Urea decomposition	2
Urea carboxylase-related ABC transporter, permease protein	1
Urease accessory protein UreE	1
Urease subunits	1
Urease accessory protein UreE	1
**Aromatic amino acids and derivatives**	**7**
Chorismate Synthesis	2
5-Enolpyruvylshikimate-3-phosphate synthase (EC 2.5.1.19)	1
Shikimate kinase I (EC 2.7.1.71)	1
Chorismate: Intermediate for synthesis of PAPA antibiotics, PABA, anthranilate, 3-hydroxyanthranilate and more.	1
Anthranilate synthase, aminase component (EC 4.1.3.27)	1

Common Pathway For Synthesis of Aromatic Compounds (DAHP synthase to chorismate)	2
5-Enolpyruvylshikimate-3-phosphate synthase (EC 2.5.1.19)	1
Shikimate kinase I (EC 2.7.1.71)	1
Tryptophan synthesis	2
Anthranilate synthase, aminase component (EC 4.1.3.27)	1
Tryptophan synthase alpha chain (EC 4.2.1.20)	1
**Branched-chain amino acids**	**19**
Branched chain amino acid degradation regulons	3
Dihydrolipoamide acyltransferase component of branched-chain alpha-keto acid dehydrogenase complex (EC 2.3.1.168)	1
Electron transfer flavoprotein, alpha subunit	1
Electron transfer flavoprotein-ubiquinone oxidoreductase (EC 1.5.5.1)	1
Branched-Chain Amino Acid Biosynthesis	3
Acetolactate synthase large subunit (EC 2.2.1.6)	1
Dihydroxy-acid dehydratase (EC 4.2.1.9)	1
Threonine dehydratase (EC 4.3.1.19)	1
HMG-CoA	2
Dihydrolipoamide acyltransferase component of branched-chain alpha-keto acid dehydrogenase complex (EC 2.3.1.168)	1
Hydroxymethylglutaryl-CoA reductase (EC 1.1.1.34)	1
Isoleucine degradation	4
Acyl-CoA dehydrogenase, short-chain specific (EC 1.3.99.2)	1
Butyrate kinase (EC 2.7.2.7)	1
Dihydrolipoamide acyltransferase component of branched-chain alpha-keto acid dehydrogenase complex (EC 2.3.1.168)	1
Enoyl-CoA hydratase (EC 4.2.1.17)	1
Ketoisovalerate oxidoreductase	1
Acetyl-coenzyme A synthetase (EC 6.2.1.1)	1
Leucine Biosynthesis	1
2-isopropylmalate synthase (EC 2.3.3.13)	1
Leucine Degradation and HMG-CoA Metabolism	2

Dihydrolipoamide acyltransferase component of branched-chain alpha-keto acid dehydrogenase complex (EC 2.3.1.168)	1
Hydroxymethylglutaryl-CoA reductase (EC 1.1.1.34)	1
Valine degradation	3
Butyrate kinase (EC 2.7.2.7)	1
Dihydrolipoamide acyltransferase component of branched-chain alpha-keto acid dehydrogenase complex (EC 2.3.1.168)	1
Enoyl-CoA hydratase (EC 4.2.1.17)	1
**Glutamine, glutamate, aspartate, asparagine; ammonia assimilation**	**11**
Glutamate and Aspartate uptake in Bacteria	1
Glutamate Aspartate transport system permease protein GltK (TC 3.A.1.3.4)	1
Glutamate dehydrogenases	1
NAD-specific glutamate dehydrogenase (EC 1.4.1.2)	1
Glutamine synthetases	1
Glutamine synthetase type I (EC 6.3.1.2)	1
Glutamine, Glutamate, Aspartate and Asparagine Biosynthesis	7
Aspartate aminotransferase (EC 2.6.1.1)	1
Aspartate racemase (EC 5.1.1.13)	1
Ferredoxin-dependent glutamate synthase (EC 1.4.7.1)	1
Glutamate synthase [NADPH] large chain (EC 1.4.1.13)	1
Glutamate synthase [NADPH] small chain (EC 1.4.1.13)	1
Glutamine synthetase type I (EC 6.3.1.2)	1
NAD-specific glutamate dehydrogenase (EC 1.4.1.2)	1
Poly-gamma-glutamate biosynthesis	1
Gamma-glutamyltranspeptidase (EC 2.3.2.2)	1
**Histidine Metabolism**	**4**
Histidine Biosynthesis	3
ATP phosphoribosyltransferase catalytic subunit (EC 2.4.2.17)	1
Histidinol dehydrogenase (EC 1.1.1.23)	1
Imidazole glycerol phosphate synthase cyclase subunit (EC 4.1.3.-)	1

Histidine Degradation	1
Histidine ammonia-lyase (EC 4.3.1.3)	1
**Lysine, threonine, methionine, and cysteine**	**23**
Cysteine Biosynthesis	1
Serine acetyltransferase (EC 2.3.1.30)	1
Lysine Biosynthesis DAP Pathway	2
N-acetyl-L,L-diaminopimelate deacetylase (EC 3.5.1.47)	1
N-succinyl-L,L-diaminopimelate desuccinylase (EC 3.5.1.18)	1
Lysine degradation	2
5-aminopentanamidase (EC 3.5.1.30)	1
Lysine 2,3-aminomutase (EC 5.4.3.2)	1
Lysine fermentation	3
Butyrate-acetoacetate CoA-transferase subunit B (EC 2.8.3.9)	1
Electron transfer flavoprotein, alpha subunit	1
Lysine 2,3-aminomutase (EC 5.4.3.2)	1
Methionine Biosynthesis	7
5,10-methylenetetrahydrofolate reductase (EC 1.5.1.20)	1
Adenosylhomocysteinase (EC 3.3.1.1)	1
Cystathionine beta-lyase (EC 4.4.1.8)	1
Homoserine O-acetyltransferase (EC 2.3.1.31)	1
Methionine ABC transporter ATP-binding protein	1
S-adenosylmethionine synthetase (EC 2.5.1.6)	1
Serine acetyltransferase (EC 2.3.1.30)	1
Methionine Degradation	4
Adenosylhomocysteinase (EC 3.3.1.1)	1
Methionine ABC transporter ATP-binding protein	1
Pyruvate-flavodoxin oxidoreductase (EC 1.2.7.-)	1
S-adenosylmethionine synthetase (EC 2.5.1.6)	1

Methionine Salvage	1
5-methylthioribose kinase (EC 2.7.1.100)	1
Threonine and Homoserine Biosynthesis	2
Aspartate aminotransferase (EC 2.6.1.1)	1
Threonine synthase (EC 4.2.3.1)	1
Threonine degradation	1
Threonine dehydratase (EC 4.3.1.19)	1
**Proline and 4-hydroxyproline**	**3**
A Hypothetical Protein Related to Proline Metabolism	1
Pyrroline-5-carboxylate reductase (EC 1.5.1.2)	1
Proline Synthesis	1
Pyrroline-5-carboxylate reductase (EC 1.5.1.2)	1
Proline, 4-hydroxyproline uptake and utilization	1
4-hydroxyproline epimerase (EC 5.1.1.8)	1
**Carbohydrates**	**150**
**Aminosugars**	**5**
Chitin and N-acetylglucosamine utilization	4
Beta-hexosaminidase (EC 3.2.1.52)	1
Chitinase (EC 3.2.1.14)	1
N-Acetyl-D-glucosamine ABC transport system, permease protein 1	1
N-acetylglucosamine-6-phosphate deacetylase (EC 3.5.1.25)	1
N-Acetyl-Galactosamine and Galactosamine Utilization	1
Beta-hexosaminidase (EC 3.2.1.52)	1
**Carbon storage regulator**	**2**
Flagellar hook-associated protein FlgK	1
Flagellar hook-associated protein FlgK	1
Flagellar hook-associated protein FlgL	1
Flagellar hook-associated protein FlgL	1

**Central carbohydrate metabolism**	**40**
Dehydrogenase complexes	1
Dihydrolipoamide acyltransferase component of branched-chain alpha-keto acid dehydrogenase complex (EC 2.3.1.168)	1
Dihydroxyacetone kinases	1
Dihydroxyacetone kinase, ATP-dependent (EC 2.7.1.29)	1
Entner-Doudoroff Pathway	6
6-phosphogluconolactonase (EC 3.1.1.31)	1
Aldehyde dehydrogenase (EC 1.2.1.3)	1
NAD-dependent glyceraldehyde-3-phosphate dehydrogenase (EC 1.2.1.12)	1
Phosphoglycerate mutase (EC 5.4.2.1)	1
Polyphosphate glucokinase (EC 2.7.1.63)	1
Pyruvate kinase (EC 2.7.1.40)	1
Ethylmalonyl-CoA pathway of C2 assimilation	1
Methylsuccinyl-CoA dehydrogenase, predicted by (Erb et al, 2007)	1
Ethylmalonyl-CoA pathway of C2 assimilation, GJO	2
Malate synthase (EC 2.3.3.9)	1
Methylsuccinyl-CoA dehydrogenase, predicted by (Erb et al, 2007)	1
Glycolate, glyoxylate interconversions	2
Glycolate dehydrogenase (EC 1.1.99.14), subunit GlcD	1
Phosphoglycolate phosphatase (EC 3.1.3.18)	1
Glycolysis and Gluconeogenesis	5
NAD-dependent glyceraldehyde-3-phosphate dehydrogenase (EC 1.2.1.12)	1
Phosphoglycerate mutase (EC 5.4.2.1)	1
Polyphosphate glucokinase (EC 2.7.1.63)	1
Pyruvate kinase (EC 2.7.1.40)	1
Pyruvate,phosphate dikinase (EC 2.7.9.1)	1
Glycolysis and Gluconeogenesis, including Archaeal enzymes	3
Phosphoglycerate mutase (EC 5.4.2.1)	1

Pyruvate kinase (EC 2.7.1.40)	1
Pyruvate,phosphate dikinase (EC 2.7.9.1)	1
Glyoxylate bypass	2
Citrate synthase (si) (EC 2.3.3.1)	1
Malate synthase (EC 2.3.3.9)	1
Methylglyoxal Metabolism	2
Aldehyde dehydrogenase (EC 1.2.1.3)	1
Lactoylglutathione lyase (EC 4.4.1.5)	1
Pentose phosphate pathway	5
6-phosphogluconate dehydrogenase, decarboxylating (EC 1.1.1.44)	1
6-phosphogluconolactonase (EC 3.1.1.31)	1
Ribose-phosphate pyrophosphokinase (EC 2.7.6.1)	1
Transaldolase (EC 2.2.1.2)	1
Transketolase (EC 2.2.1.1)	1
Pyruvate Alanine Serine Interconversions	2
Alanine dehydrogenase (EC 1.4.1.1)	1
D-serine/D-alanine/glycine transporter	1
Pyruvate metabolism I: anaplerotic reactions, PEP	3
Oxaloacetate decarboxylase beta chain (EC 4.1.1.3)	1
Pyruvate kinase (EC 2.7.1.40)	1
Pyruvate,phosphate dikinase (EC 2.7.9.1)	1
Pyruvate metabolism II: acetyl-CoA, acetogenesis from pyruvate	3
Acetyl-coenzyme A synthetase (EC 6.2.1.1)	1
Aldehyde dehydrogenase (EC 1.2.1.3)	1
Pyruvate oxidase [ubiquinone, cytochrome] (EC 1.2.2.2)	1
Pyruvate:ferredoxin oxidoreductase	1
Pyruvate-flavodoxin oxidoreductase (EC 1.2.7.-)	1
TCA Cycle	1

Citrate synthase (si) (EC 2.3.3.1)	1
**CO_2 fixation**	**9**
Calvin-Benson cycle	3
NAD-dependent glyceraldehyde-3-phosphate dehydrogenase (EC 1.2.1.12)	1
Phosphoribulokinase (EC 2.7.1.19)	1
Transketolase (EC 2.2.1.1)	1
Photorespiration (oxidative C2 cycle)	6
Catalase (EC 1.11.1.6)	1
Glycerate kinase (EC 2.7.1.31)	1
Glycine dehydrogenase [decarboxylating] (glycine cleavage system P protein) (EC 1.4.4.2)	1
Glycolate dehydrogenase (EC 1.1.99.14), subunit GlcD	1
Malate synthase (EC 2.3.3.9)	1
Phosphoglycolate phosphatase (EC 3.1.3.18)	1
**Di- and oligosaccharides**	**19**
Beta-Glucoside Metabolism	2
6-phospho-beta-glucosidase (EC 3.2.1.86)	1
Beta-glucosidase (EC 3.2.1.21)	1
Fructooligosaccharides(FOS) and Raffinose Utilization	3
Alpha-galactosidase (EC 3.2.1.22)	1
Beta-glucosidase (EC 3.2.1.21)	1
Sucrose-6-phosphate hydrolase (EC 3.2.1.26)	1
Lactose and Galactose Uptake and Utilization	5
Alpha-galactosidase (EC 3.2.1.22)	1
Beta-galactosidase (EC 3.2.1.23)	1
Galactokinase (EC 2.7.1.6)	1
Galactose/methyl galactoside ABC transport system, ATP-binding protein MglA (EC 3.6.3.17)	1
UDP-glucose 4-epimerase (EC 5.1.3.2)	1
Lactose utilization	1

Beta-galactosidase (EC 3.2.1.23)	1
Maltose and Maltodextrin Utilization	3
4-alpha-glucanotransferase (amylomaltase) (EC 2.4.1.25)	1
Alpha-amylase (EC 3.2.1.1)	1
Maltose/maltodextrin ABC transporter, permease protein MalG	1
Melibiose Utilization	1
Alpha-galactosidase (EC 3.2.1.22)	1
Sucrose utilization	1
Sucrose-6-phosphate hydrolase (EC 3.2.1.26)	1
Trehalose Biosynthesis	3
Alpha-amylase (EC 3.2.1.1)	1
Glycogen debranching enzyme (EC 3.2.1.-)	1
Trehalose-6-phosphate phosphatase (EC 3.1.3.12)	1
**Fermentation**	**15**
Acetoin, butanediol metabolism	3
Acetolactate synthase large subunit (EC 2.2.1.6)	1
Acetolactate synthase, catabolic (EC 2.2.1.6)	1
Transcriptional activator of acetoin dehydrogenase operon AcoR	1
Acetone Butanol Ethanol Synthesis	2
Alcohol dehydrogenase (EC 1.1.1.1)	1
Electron transfer flavoprotein, alpha subunit	1
Acetyl-CoA fermentation to Butyrate	5
Butyrate kinase (EC 2.7.2.7)	1
Butyrate-acetoacetate CoA-transferase subunit B (EC 2.8.3.9)	1
Electron transfer flavoprotein, alpha subunit	1
Electron transfer flavoprotein-ubiquinone oxidoreductase (EC 1.5.5.1)	1
Enoyl-CoA hydratase (EC 4.2.1.17)	1
Butanol Biosynthesis	2

Enoyl-CoA hydratase (EC 4.2.1.17)	1
Pyruvate formate-lyase (EC 2.3.1.54)	1
Fermentations: Mixed acid	3
Alcohol dehydrogenase (EC 1.1.1.1)	1
Pyruvate formate-lyase (EC 2.3.1.54)	1
Sugar/maltose fermentation stimulation protein homolog	1
**Lacto-N-Biose I and Galacto-N-Biose Metabolic Pathway**	**1**
UDP-glucose 4-epimerase (EC 5.1.3.2)	1
UDP-glucose 4-epimerase (EC 5.1.3.2)	1
**Monosaccharides**	**9**
Deoxyribose and Deoxynucleoside Catabolism	1
Putative deoxyribonuclease YjjV	1
D-galactarate, D-glucarate and D-glycerate catabolism	2
Glucarate dehydratase (EC 4.2.1.40)	1
Glycerate kinase (EC 2.7.1.31)	1
D-Galacturonate and D-Glucuronate Utilization	1
D-mannonate oxidoreductase (EC 1.1.1.57)	1
Fructose utilization	3
Phosphotransferase system, phosphocarrier protein HPr	1
Transaldolase (EC 2.2.1.2)	1
Transcriptional repressor of the fructose operon, DeoR family	1
Unknown pentose utilization	1
Transketolase (EC 2.2.1.1)	1
Xylose utilization	1
Xylulose kinase (EC 2.7.1.17)	1
**One-carbon Metabolism**	**5**
One-carbon metabolism by tetrahydropterines	1
5,10-methylenetetrahydrofolate reductase (EC 1.5.1.20)	1

Serine-glyoxylate cycle	4
5,10-methylenetetrahydrofolate reductase (EC 1.5.1.20)	1
Citrate synthase (si) (EC 2.3.3.1)	1
Glycerate kinase (EC 2.7.1.31)	1
Methylmalonyl-CoA mutase (EC 5.4.99.2)	1
**Organic acids**	**8**
Alpha-acetolactate operon	1
Acetolactate synthase, catabolic (EC 2.2.1.6)	1
Glycerate metabolism	2
Glycerate kinase (EC 2.7.1.31)	1
Pyruvate kinase (EC 2.7.1.40)	1
Lactate utilization	1
Predicted D-lactate dehydrogenase, Fe-S protein, FAD/FMN-containing	1
Methylcitrate cycle	1
Methylisocitrate lyase (EC 4.1.3.30)	1
Propionate-CoA to Succinate Module	1
Methylisocitrate lyase (EC 4.1.3.30)	1
Propionyl-CoA to Succinyl-CoA Module	1
Methylmalonyl-CoA mutase (EC 5.4.99.2)	1
Tricarballylate Utilization	1
TcuB: works with TcuA to oxidize tricarballylate to cis-aconitate	1
**Polysaccharides**	**4**
Glycogen metabolism	4
4-alpha-glucanotransferase (amylomaltase) (EC 2.4.1.25)	1
Glucose-1-phosphate adenylyltransferase (EC 2.7.7.27)	1
Glycogen debranching enzyme (EC 3.2.1.-)	1
Glycogen synthase, ADP-glucose transglucosylase (EC 2.4.1.21)	1
**Sugar alcohols**	**12**

Di-Inositol-Phosphate biosynthesis	1
Inositol-1-monophosphatase (EC 3.1.3.25)	1
Glycerol and Glycerol-3-phosphate Uptake and Utilization	5
Aerobic glycerol-3-phosphate dehydrogenase (EC 1.1.5.3)	1
Glycerol-3-phosphate ABC transporter, periplasmic glycerol-3-phosphate-binding protein (TC 3.A.1.1.3)	1
Glycerol-3-phosphate ABC transporter, permease protein UgpE (TC 3.A.1.1.3)	1
Glycerol-3-phosphate dehydrogenase (EC 1.1.5.3)	1
Glycerol-3-phosphate dehydrogenase [NAD(P)+] (EC 1.1.1.94)	1
Glycerol fermenation to 1,3-propanediol	1
Cob(I)alamin adenosyltransferase PduO (EC 2.5.1.17)	1
Inositol catabolism	1
Myo-inositol 2-dehydrogenase (EC 1.1.1.18)	1
Inositol utilization	2
Inositol-1-monophosphatase (EC 3.1.3.25)	1
Myo-inositol 2-dehydrogenase (EC 1.1.1.18)	1
Propanediol utilization	1
Cob(I)alamin adenosyltransferase PduO (EC 2.5.1.17)	1
Ribitol, Xylitol, Arabitol, Mannitol and Sorbitol utilization	1
Xylulose kinase (EC 2.7.1.17)	1
**Sugar utilization in Thermotogales**	**19**
6-phosphogluconate dehydrogenase, decarboxylating (EC 1.1.1.44)	1
6-phosphogluconate dehydrogenase, decarboxylating (EC 1.1.1.44)	1
Alpha-galactosidase (EC 3.2.1.22)	1
Alpha-galactosidase (EC 3.2.1.22)	1
Beta-galactosidase (EC 3.2.1.23)	1
Beta-galactosidase (EC 3.2.1.23)	1
Beta-glucosidase (EC 3.2.1.21)	1
Beta-glucosidase (EC 3.2.1.21)	1

Beta-hexosaminidase (EC 3.2.1.52)	1
Beta-hexosaminidase (EC 3.2.1.52)	1
D-mannonate oxidoreductase (EC 1.1.1.57)	1
D-mannonate oxidoreductase (EC 1.1.1.57)	1
Galactokinase (EC 2.7.1.6)	1
Galactokinase (EC 2.7.1.6)	1
Glycerol-3-phosphate ABC transporter, periplasmic glycerol-3-phosphate-binding protein (TC 3.A.1.1.3)	1
Glycerol-3-phosphate ABC transporter, periplasmic glycerol-3-phosphate-binding protein (TC 3.A.1.1.3)	1
Glycerol-3-phosphate ABC transporter, permease protein UgpE (TC 3.A.1.1.3)	1
Glycerol-3-phosphate ABC transporter, permease protein UgpE (TC 3.A.1.1.3)	1
Glycerol-3-phosphate dehydrogenase (EC 1.1.5.3)	1
Glycerol-3-phosphate dehydrogenase (EC 1.1.5.3)	1
N-acetylglucosamine-6-phosphate deacetylase (EC 3.5.1.25)	1
N-acetylglucosamine-6-phosphate deacetylase (EC 3.5.1.25)	1
NAD-dependent glyceraldehyde-3-phosphate dehydrogenase (EC 1.2.1.12)	1
NAD-dependent glyceraldehyde-3-phosphate dehydrogenase (EC 1.2.1.12)	1
Phosphoglycerate mutase (EC 5.4.2.1)	1
Phosphoglycerate mutase (EC 5.4.2.1)	1
Phosphotransferase system, phosphocarrier protein HPr	1
Phosphotransferase system, phosphocarrier protein HPr	1
Pyruvate kinase (EC 2.7.1.40)	1
Pyruvate kinase (EC 2.7.1.40)	1
Transaldolase (EC 2.2.1.2)	1
Transaldolase (EC 2.2.1.2)	1
Transketolase (EC 2.2.1.1)	1
Transketolase (EC 2.2.1.1)	1
UDP-glucose 4-epimerase (EC 5.1.3.2)	1
UDP-glucose 4-epimerase (EC 5.1.3.2)	1

Xylulose kinase (EC 2.7.1.17)	1
Xylulose kinase (EC 2.7.1.17)	1
**Unknown carbohydrate utilization (cluster Ydj)**	**1**
Peptide methionine sulfoxide reductase MsrB (EC 1.8.4.12)	1
Peptide methionine sulfoxide reductase MsrB (EC 1.8.4.12)	1
**VC0266**	**1**
Hypothetical protein VC0266 (sugar utilization related?)	1
Hypothetical protein VC0266 (sugar utilization related?)	1
**Cell Division and Cell Cycle**	**12**
**Bacterial Cytoskeleton**	**7**
Cell division protein FtsA	1
Cell division protein FtsA	1
Cell division protein FtsI [Peptidoglycan synthetase] (EC 2.4.1.129)	1
Cell division protein FtsI [Peptidoglycan synthetase] (EC 2.4.1.129)	1
Cell division protein FtsK	1
Cell division protein FtsK	1
Cell division protein FtsW	1
Cell division protein FtsW	1
Rod shape-determining protein MreB	1
Rod shape-determining protein MreB	1
Rod shape-determining protein MreD	1
Rod shape-determining protein MreD	1
Septum formation protein Maf	1
Septum formation protein Maf	1
**Macromolecular synthesis operon**	**2**
Carbamoyl-phosphate synthase large chain (EC 6.3.5.5)	1
Carbamoyl-phosphate synthase large chain (EC 6.3.5.5)	1
Transamidase GatB domain protein	1

Transamidase GatB domain protein	1
**Two cell division clusters relating to chromosome partitioning**	**2**
Chromosome partition protein smc	1
Chromosome partition protein smc	1
Signal recognition particle, subunit Ffh SRP54 (TC 3.A.5.1.1)	1
Signal recognition particle, subunit Ffh SRP54 (TC 3.A.5.1.1)	1
**YgjD and YeaZ**	**1**
Inactive homolog of metal-dependent proteases, putative molecular chaperone	1
Inactive homolog of metal-dependent proteases, putative molecular chaperone	1
**Cell Wall and Capsule**	**65**
**Capsular and extracellular polysacchrides**	**27**
Alginate metabolism	1
Probable poly(beta-D-mannuronate) O-acetylase (EC 2.3.1.-)	1
Capsular heptose biosynthesis	1
GDP-L-fucose synthetase (EC 1.1.1.271)	1
Capsular Polysaccharides Biosynthesis and Assembly	3
Capsular polysaccharide export system protein KpsC	1
Oligosaccharide repeat unit polymerase Wzy	1
Tyrosine-protein kinase Wzc (EC 2.7.10.2)	1
CMP-N-acetylneuraminate Biosynthesis	2
N-Acetylneuraminate cytidylyltransferase (EC 2.7.7.43)	1
UDP-N-acetylglucosamine 2-epimerase (EC 5.1.3.14)	1
Colanic acid biosynthesis	2
GDP-L-fucose synthetase (EC 1.1.1.271)	1
Tyrosine-protein kinase Wzc (EC 2.7.10.2)	1
Exopolysaccharide Biosynthesis	2
Capsular polysaccharide synthesis enzyme CpsD, exopolysaccharide synthesis	1
Glycosyl transferase, group 2 family protein	1

Legionaminic Acid Biosynthesis	1
UDP-N-acetylglucosamine 2-epimerase (EC 5.1.3.14)	1
Rhamnose containing glycans	2
Capsular polysaccharide export system protein KpsC	1
UDP-glucose 4-epimerase (EC 5.1.3.2)	1
Sialic Acid Metabolism	3
N-acetylglucosamine-6-phosphate deacetylase (EC 3.5.1.25)	1
N-Acetylneuraminate cytidylyltransferase (EC 2.7.7.43)	1
UDP-N-acetylglucosamine 2-epimerase (EC 5.1.3.14)	1
Vibrio Polysaccharide (VPS) Biosynthesis	10
Capsular polysaccharide synthesis enzyme CpsD, exopolysaccharide synthesis	1
Hypothetical protein RbmB	1
Hypothetical protein RbmF	1
Hypothetical protein VpsF	1
Hypothetical protein VpsJ	1
Hypothetical protein VpsP	1
Hypothetical protein VpsQ	1
N-acetylmannosaminyltransferase (EC 2.4.1.187)	1
Serine acetyltransferase (EC 2.3.1.30)	1
UDP-N-acetylglucosamine 2-epimerase (EC 5.1.3.14)	1
**Cell wall of Mycobacteria**	**3**
linker unit-arabinogalactan synthesis	1
UDP-glucose 4-epimerase (EC 5.1.3.2)	1
mycolic acid synthesis	2
3-oxoacyl-[acyl-carrier protein] reductase (EC 1.1.1.100)	1
Malonyl CoA-acyl carrier protein transacylase (EC 2.3.1.39)	1
**Gram-Negative cell wall components**	**16**
Inner membrane protein YhjD and conserved cluster involved in LPS biosynthesis	1

Uncharacterized protein YhjG	1
KDO2-Lipid A biosynthesis	7
3-deoxy-manno-octulosonate cytidylyltransferase (EC 2.7.7.38)	1
Acyl-[acyl-carrier-protein]--UDP-N-acetylglucosamine O-acyltransferase (EC 2.3.1.129)	1
Lipid A biosynthesis lauroyl acyltransferase (EC 2.3.1.-)	1
Lipid A export ATP-binding/permease protein MsbA (EC 3.6.3.25)	1
Lipid-A-disaccharide synthase (EC 2.4.1.182)	1
Oligosaccharide repeat unit polymerase Wzy	1
Predicted hydrolase of the metallo-beta-lactamase superfamily, clustered with KDO2-Lipid A biosynthesis genes	1
Lipid A modifications	1
Phosphoethanolamine transferase EptA specific for the 1 phosphate group of core-lipid A	1
Lipopolysaccharide assembly	2
Lipoprotein releasing system ATP-binding protein LolD	1
Lipoprotein releasing system transmembrane protein LolE	1
Lipopolysaccharide-related cluster in Alphaproteobacteria	2
Lipid A export ATP-binding/permease protein MsbA (EC 3.6.3.25)	1
TldE/PmbA protein, part of proposed TldE/TldD proteolytic complex (PMID 12029038)	1
Lipoprotein sorting system	2
Lipoprotein releasing system ATP-binding protein LolD	1
Lipoprotein releasing system transmembrane protein LolE	1
LOS core oligosaccharide biosynthesis	1
Lipopolysaccharide core biosynthesis protein RfaY	1
**Gram-Positive cell wall components**	**2**
Teichoic and lipoteichoic acids biosynthesis	2
N-acetylmannosaminyltransferase (EC 2.4.1.187)	1
Undecaprenyl-phosphate N-acetylglucosaminyl 1-phosphate transferase (EC 2.7.8.-)	1
**Murein Hydrolases**	**3**
D-alanyl-D-alanine carboxypeptidase (EC 3.4.16.4)	1

D-alanyl-D-alanine carboxypeptidase (EC 3.4.16.4)	1
Membrane-bound lytic murein transglycosylase B precursor (EC 3.2.1.-)	1
Membrane-bound lytic murein transglycosylase B precursor (EC 3.2.1.-)	1
N-acetylmuramoyl-L-alanine amidase (EC 3.5.1.28)	1
N-acetylmuramoyl-L-alanine amidase (EC 3.5.1.28)	1
**Peptidoglycan Biosynthesis**	**8**
Cell division protein FtsI [Peptidoglycan synthetase] (EC 2.4.1.129)	1
Cell division protein FtsI [Peptidoglycan synthetase] (EC 2.4.1.129)	1
D-alanine--D-alanine ligase (EC 6.3.2.4)	1
D-alanine--D-alanine ligase (EC 6.3.2.4)	1
D-alanyl-D-alanine carboxypeptidase (EC 3.4.16.4)	1
D-alanyl-D-alanine carboxypeptidase (EC 3.4.16.4)	1
Glutamine synthetase type I (EC 6.3.1.2)	1
Glutamine synthetase type I (EC 6.3.1.2)	1
Membrane-bound lytic murein transglycosylase B precursor (EC 3.2.1.-)	1
Membrane-bound lytic murein transglycosylase B precursor (EC 3.2.1.-)	1
Multimodular transpeptidase-transglycosylase (EC 2.4.1.129) (EC 3.4.-.-)	1
Multimodular transpeptidase-transglycosylase (EC 2.4.1.129) (EC 3.4.-.-)	1
Penicillin-binding protein 2 (PBP-2)	1
Penicillin-binding protein 2 (PBP-2)	1
Phospho-N-acetylmuramoyl-pentapeptide-transferase (EC 2.7.8.13)	1
Phospho-N-acetylmuramoyl-pentapeptide-transferase (EC 2.7.8.13)	1
**Peptidoglycan biosynthesis--gjo**	**1**
D-alanine--D-alanine ligase (EC 6.3.2.4)	1
D-alanine--D-alanine ligase (EC 6.3.2.4)	1
**Recycling of Peptidoglycan Amino Acids**	**1**
N-acetylmuramoyl-L-alanine amidase (EC 3.5.1.28)	1
N-acetylmuramoyl-L-alanine amidase (EC 3.5.1.28)	1

**UDP-N-acetylmuramate from Fructose-6-phosphate Biosynthesis**	**1**
N-acetylglucosamine-6-phosphate deacetylase (EC 3.5.1.25)	1
N-acetylglucosamine-6-phosphate deacetylase (EC 3.5.1.25)	1
**YjeE**	**3**
ATPase YjeE, predicted to have essential role in cell wall biosynthesis	1
ATPase YjeE, predicted to have essential role in cell wall biosynthesis	1
COG3178: Predicted phosphotransferase related to Ser/Thr protein kinases	1
COG3178: Predicted phosphotransferase related to Ser/Thr protein kinases	1
Inactive homolog of metal-dependent proteases, putative molecular chaperone	1
Inactive homolog of metal-dependent proteases, putative molecular chaperone	1
**Cofactors, Vitamins, Prosthetic Groups, Pigments**	**65**
**Biotin**	**2**
Biotin biosynthesis	2
Biotin synthase (EC 2.8.1.6)	1
Long-chain-fatty-acid--CoA ligase (EC 6.2.1.3)	1
**Coenzyme A**	**1**
Coenzyme A Biosynthesis	1
Pantothenate:Na+ symporter (TC 2.A.21.1.1)	1
**Folate and pterines**	**26**
5-FCL-like protein	3
5,10-methylenetetrahydrofolate reductase (EC 1.5.1.20)	1
Alcohol dehydrogenase (EC 1.1.1.1)	1
Dihydrofolate reductase (EC 1.5.1.3)	1
Folate Biosynthesis	4
Dihydrofolate reductase (EC 1.5.1.3)	1
Dihydropteroate synthase (EC 2.5.1.15)	1
Nucleoside triphosphate pyrophosphohydrolase MazG	1
Transaldolase (EC 2.2.1.2)	1

Molybdenum cofactor biosynthesis	4
Molybdenum cofactor biosynthesis protein MoaB	1
Molybdenum transport system permease protein ModB (TC 3.A.1.8.1)	1
Molybdopterin biosynthesis enzyme	1
Molybdopterin biosynthesis protein MoeA	1
Pterin metabolism 3	1
Dihydropteroate synthase (EC 2.5.1.15)	1
YgfZ	12
5,10-methylenetetrahydrofolate reductase (EC 1.5.1.20)	1
Adenosylcobinamide-phosphate synthase	1
Biotin synthase (EC 2.8.1.6)	1
Coproporphyrinogen III oxidase, oxygen-independent (EC 1.3.99.22)	1
Dihydroorotase (EC 3.5.2.3)	1
Dihydropteroate synthase (EC 2.5.1.15)	1
Glutamate synthase [NADPH] small chain (EC 1.4.1.13)	1
Orotate phosphoribosyltransferase (EC 2.4.2.10)	1
Phosphoribosylformylglycinamidine cyclo-ligase (EC 6.3.3.1)	1
Ribose-phosphate pyrophosphokinase (EC 2.7.6.1)	1
Thiazole biosynthesis protein ThiH	1
YgfY COG2938	1
YgfZ-Iron	2
Cytochrome c heme lyase subunit CcmF	1
Dihydroorotase (EC 3.5.2.3)	1
**Lipoic acid**	**1**
Lipoic acid metabolism	1
Lipoate-protein ligase A	1
**Molybdopterin cytosine dinucleotide**	**1**
Molybdopterin biosynthesis enzyme	1

Molybdopterin biosynthesis enzyme	1
**NAD and NADP**	**1**
NAD and NADP cofactor biosynthesis global	1
ADP-ribose pyrophosphatase (EC 3.6.1.13)	1
**Pyridoxine**	**3**
Pyridoxin (Vitamin B6) Biosynthesis	3
1-deoxy-D-xylulose 5-phosphate synthase (EC 2.2.1.7)	1
D-3-phosphoglycerate dehydrogenase (EC 1.1.1.95)	1
NAD-dependent glyceraldehyde-3-phosphate dehydrogenase (EC 1.2.1.12)	1
**Quinone cofactors**	**2**
Menaquinone and Phylloquinone Biosynthesis	1
Naphthoate synthase (EC 4.1.3.36)	1
Ubiquinone Biosynthesis	1
3-demethylubiquinone-9 3-methyltransferase (EC 2.1.1.64)	1
**Tetrapyrroles**	**28**
Chlorophyll Biosynthesis	1
Light-independent protochlorophyllide reductase subunit N (EC 1.18.-.-)	1
Cobalamin synthesis	4
Adenosylcobinamide-phosphate synthase	1
Cobalt-precorrin-6 synthase, anaerobic	1
Cobyric acid synthase	1
Cobyrinic acid A,C-diamide synthase	1
Coenzyme B12 biosynthesis	6
Adenosylcobinamide-phosphate synthase	1
Cob(I)alamin adenosyltransferase PduO (EC 2.5.1.17)	1
Cobalt-precorrin-6 synthase, anaerobic	1
Cobyric acid synthase	1
Cobyrinic acid A,C-diamide synthase	1

Uroporphyrinogen-III methyltransferase (EC 2.1.1.107)	1
CPO analysis	6
Coproporphyrinogen III oxidase, oxygen-independent (EC 1.3.99.22)	1
Fe-S protein, homolog of lactate dehydrogenase SO1521	1
Glutamate-1-semialdehyde aminotransferase (EC 5.4.3.8)	1
Hypothetical protein, ydbT homolog	1
Porphobilinogen synthase (EC 4.2.1.24)	1
Uroporphyrinogen-III methyltransferase (EC 2.1.1.107)	1
Experimental tye	7
Coproporphyrinogen III oxidase, oxygen-independent (EC 1.3.99.22)	1
Cytochrome c-type biogenesis protein CcdA (DsbD analog)	1
Cytochrome c-type biogenesis protein CcsA/ResC	1
Glutamate-1-semialdehyde aminotransferase (EC 5.4.3.8)	1
Hypothetical protein, ydbT homolog	1
Porphobilinogen synthase (EC 4.2.1.24)	1
Uroporphyrinogen-III methyltransferase (EC 2.1.1.107)	1
Heme and Siroheme Biosynthesis	4
Coproporphyrinogen III oxidase, oxygen-independent (EC 1.3.99.22)	1
Glutamate-1-semialdehyde aminotransferase (EC 5.4.3.8)	1
Porphobilinogen synthase (EC 4.2.1.24)	1
Uroporphyrinogen-III methyltransferase (EC 2.1.1.107)	1
**DNA Metabolism**	**48**
**CRISPs**	**2**
CRISPRs	2
CRISPR-associated protein Cas1	1
CRISPR-associated protein Cas2	1
**DNA repair**	**22**
2-phosphoglycolate salvage	1

Phosphoglycolate phosphatase (EC 3.1.3.18)	1
DNA Repair Base Excision	5
DNA polymerase I (EC 2.7.7.7)	1
DNA-3-methyladenine glycosylase (EC 3.2.2.20)	1
Formamidopyrimidine-DNA glycosylase (EC 3.2.2.23)	1
Single-stranded-DNA-specific exonuclease RecJ (EC 3.1.-.-)	1
Uracil-DNA glycosylase, family 1	1
DNA repair, bacterial	5
A/G-specific adenine glycosylase (EC 3.2.2.-)	1
DNA polymerase IV (EC 2.7.7.7)	1
Exodeoxyribonuclease VII large subunit (EC 3.1.11.6)	1
Exonuclease SbcC	1
Exonuclease SbcD	1
DNA repair, bacterial MutL-MutS system	1
DNA mismatch repair protein MutL	1
DNA repair, bacterial photolyase	1
Deoxyribodipyrimidine photolyase (EC 4.1.99.3)	1
DNA repair, bacterial RecBCD pathway	2
ATP-dependent DNA helicase SCO5183	1
Exodeoxyribonuclease V beta chain (EC 3.1.11.5)	1
DNA repair, bacterial RecFOR pathway	4
ATP-dependent DNA helicase RecQ	1
DNA recombination and repair protein RecO	1
Recombination protein RecR	1
Single-stranded-DNA-specific exonuclease RecJ (EC 3.1.-.-)	1
DNA repair, bacterial UvrD and related helicases	2
ATP-dependent DNA helicase UvrD/PcrA	1
DNA helicase IV	1

Uracil-DNA glycosylase	1
Uracil-DNA glycosylase, family 1	1
**DNA replication**	**17**
DNA replication, archaeal	1
Archaeal DNA polymerase I (EC 2.7.7.7)	1
DNA topoisomerases, Type II, ATP-dependent	3
DNA gyrase subunit A (EC 5.99.1.3)	1
DNA gyrase subunit B (EC 5.99.1.3)	1
Topoisomerase IV subunit A (EC 5.99.1.-)	1
DNA-replication	11
ATP-dependent DNA helicase RecQ	1
DNA polymerase I (EC 2.7.7.7)	1
DNA polymerase III alpha subunit (EC 2.7.7.7)	1
DNA polymerase III beta subunit (EC 2.7.7.7)	1
DNA polymerase III subunits gamma and tau (EC 2.7.7.7)	1
Exodeoxyribonuclease V beta chain (EC 3.1.11.5)	1
Helicase PriA essential for oriC/DnaA-independent DNA replication	1
Recombination protein RecR	1
Replicative DNA helicase (EC 3.6.1.-)	1
Single-stranded-DNA-specific exonuclease RecJ (EC 3.1.-.-)	1
Transcription-repair coupling factor	1
Plasmid replication	2
Plasmid replication protein RepA	1
Plasmid replication protein RepB	1
**DNA uptake, competence**	**4**
DNA processing cluster	3
DNA polymerase III subunits gamma and tau (EC 2.7.7.7)	1
FIG000557: hypothetical protein co-occurring with RecR	1

Recombination protein RecR	1
Gram Positive Competence	1
Competence protein CoiA	1
**Restriction-Modification System**	**1**
Type I restriction-modification system, specificity subunit S (EC 3.1.21.3)	1
Type I restriction-modification system, specificity subunit S (EC 3.1.21.3)	1
**Type I Restriction-Modification**	**1**
Type I restriction-modification system, specificity subunit S (EC 3.1.21.3)	1
Type I restriction-modification system, specificity subunit S (EC 3.1.21.3)	1
**YcfH**	**1**
Putative deoxyribonuclease YjjV	1
Putative deoxyribonuclease YjjV	1
**Dormancy and Sporulation**	**3**
**Persister Cells**	**1**
HipA protein	1
HipA protein	1
**Sporulation Cluster**	**2**
Nucleoside triphosphate pyrophosphohydrolase MazG	1
Nucleoside triphosphate pyrophosphohydrolase MazG	1
Transcription-repair coupling factor	1
Transcription-repair coupling factor	1
**Fatty Acids, Lipids, and Isoprenoids**	**30**
**Fatty acids**	**7**
Fatty Acid Biosynthesis FASII	3
3-oxoacyl-[acyl-carrier protein] reductase (EC 1.1.1.100)	1
Enoyl-[acyl-carrier-protein] reductase [NADPH] (EC 1.3.1.10)	1
Malonyl CoA-acyl carrier protein transacylase (EC 2.3.1.39)	1
Fatty acid degradation regulons	2

Enoyl-CoA hydratase (EC 4.2.1.17)	1
Long-chain-fatty-acid--CoA ligase (EC 6.2.1.3)	1
Polyunsaturated Fatty Acids synthesis	1
omega-3 polyunsaturated fatty acid synthase subunit, PfaA	1
Unsaturated Fatty Acid Metabolism	1
Long-chain-fatty-acid--CoA ligase (EC 6.2.1.3)	1
**Isoprenoids**	**13**
Archaeal lipids	1
Hydroxymethylglutaryl-CoA reductase (EC 1.1.1.34)	1
Isoprenoid Biosynthesis	5
1-deoxy-D-xylulose 5-phosphate reductoisomerase (EC 1.1.1.267)	1
1-deoxy-D-xylulose 5-phosphate synthase (EC 2.2.1.7)	1
4-hydroxy-3-methylbut-2-enyl diphosphate reductase (EC 1.17.1.2)	1
Hydroxymethylglutaryl-CoA reductase (EC 1.1.1.34)	1
Undecaprenyl pyrophosphate synthetase (EC 2.5.1.31)	1
Mevalonate Branch of Isoprenoid Biosynthesis	1
Hydroxymethylglutaryl-CoA reductase (EC 1.1.1.34)	1
Nonmevalonate Branch of Isoprenoid Biosynthesis	3
1-deoxy-D-xylulose 5-phosphate reductoisomerase (EC 1.1.1.267)	1
1-deoxy-D-xylulose 5-phosphate synthase (EC 2.2.1.7)	1
4-hydroxy-3-methylbut-2-enyl diphosphate reductase (EC 1.17.1.2)	1
Polyprenyl Diphosphate Biosynthesis	1
Undecaprenyl pyrophosphate synthetase (EC 2.5.1.31)	1
polyprenyl synthesis	2
1-deoxy-D-xylulose 5-phosphate reductoisomerase (EC 1.1.1.267)	1
4-hydroxy-3-methylbut-2-enyl diphosphate reductase (EC 1.17.1.2)	1
**Phospholipids**	**8**
Glycerolipid and Glycerophospholipid Metabolism in Bacteria	8

1-acyl-sn-glycerol-3-phosphate acyltransferase (EC 2.3.1.51)	1
Aerobic glycerol-3-phosphate dehydrogenase (EC 1.1.5.3)	1
Alcohol dehydrogenase (EC 1.1.1.1)	1
Aldehyde dehydrogenase (EC 1.2.1.3)	1
Glycerate kinase (EC 2.7.1.31)	1
Glycerol-3-phosphate dehydrogenase (EC 1.1.5.3)	1
Glycerol-3-phosphate dehydrogenase [NAD(P)+] (EC 1.1.1.94)	1
Phosphatidate cytidylyltransferase (EC 2.7.7.41)	1
**Polyhydroxybutyrate metabolism**	**2**
Butyrate-acetoacetate CoA-transferase subunit B (EC 2.8.3.9)	1
Butyrate-acetoacetate CoA-transferase subunit B (EC 2.8.3.9)	1
Enoyl-CoA hydratase (EC 4.2.1.17)	1
Enoyl-CoA hydratase (EC 4.2.1.17)	1
**Iron acquisition and metabolism**	**16**
**Campylobacter Iron Metabolism**	**2**
Ferric siderophore transport system, periplasmic binding protein TonB	1
Ferric siderophore transport system, periplasmic binding protein TonB	1
Ferrous iron transport protein B	1
Ferrous iron transport protein B	1
**Heme, hemin uptake and utilization systems in GramPositives**	**1**
Hypothetical protein DUF454	1
Hypothetical protein DUF454	1
**Hemin transport system**	**4**
ABC-type hemin transport system, ATPase component	1
ABC-type hemin transport system, ATPase component	1
Ferric siderophore transport system, periplasmic binding protein TonB	1
Ferric siderophore transport system, periplasmic binding protein TonB	1
Outer membrane receptor proteins, mostly Fe transport	1

Outer membrane receptor proteins, mostly Fe transport	1
TonB-dependent hemin , ferrichrome receptor	1
TonB-dependent hemin , ferrichrome receptor	1
**Iron acquisition in Vibrio**	**4**
Ferric hydroxamate ABC transporter (TC 3.A.1.14.3), permease component FhuB	1
Ferric hydroxamate ABC transporter (TC 3.A.1.14.3), permease component FhuB	1
Ferrous iron transport protein B	1
Ferrous iron transport protein B	1
Hypothetical protein colocalized with Enterobactin receptor VctA	1
Hypothetical protein colocalized with Enterobactin receptor VctA	1
TonB-dependent receptor	1
TonB-dependent receptor	1
**Siderophores**	**4**
Siderophore Aerobactin	1
Ferric hydroxamate ABC transporter (TC 3.A.1.14.3), permease component FhuB	1
Siderophore assembly kit	3
ABC-type hemin transport system, ATPase component	1
Ferric hydroxamate ABC transporter (TC 3.A.1.14.3), permease component FhuB	1
TonB-dependent hemin , ferrichrome receptor	1
**Transport of Iron**	**1**
Ferrous iron transport protein B	1
Ferrous iron transport protein B	1
**Membrane Transport**	**33**
**ABC transporters**	**7**
ABC transporter branched-chain amino acid (TC 3.A.1.4.1)	2
Branched-chain amino acid ABC transporter, amino acid-binding protein (TC 3.A.1.4.1)	1
High-affinity branched-chain amino acid transport system permease protein LivH (TC 3.A.1.4.1)	1
ABC transporter dipeptide (TC 3.A.1.5.2)	2

Dipeptide transport system permease protein DppC (TC 3.A.1.5.2)	1
Dipeptide-binding ABC transporter, periplasmic substrate-binding component (TC 3.A.1.5.2)	1
ABC transporter oligopeptide (TC 3.A.1.5.1)	3
Oligopeptide ABC transporter, periplasmic oligopeptide-binding protein OppA (TC 3.A.1.5.1)	1
Oligopeptide transport ATP-binding protein OppD (TC 3.A.1.5.1)	1
Oligopeptide transport system permease protein OppC (TC 3.A.1.5.1)	1
**Choline Transport**	**1**
Sodium-Choline Symporter	1
Sodium-Choline Symporter	1
**ECF class transporters**	**1**
ATPase component STY3232 of energizing module of queuosine-regulated ECF transporter	1
ATPase component STY3232 of energizing module of queuosine-regulated ECF transporter	1
**Folate transporters**	**1**
Dihydrofolate synthase (EC 6.3.2.12) / Folylpolyglutamate synthase (EC 6.3.2.17)	1
Dihydrofolate synthase (EC 6.3.2.12) / Folylpolyglutamate synthase (EC 6.3.2.17)	1
**Protein and nucleoprotein secretion system, Type IV**	**2**
Type IV pilus	2
Multimodular transpeptidase-transglycosylase (EC 2.4.1.129) (EC 3.4.-.-)	1
Type IV fimbrial assembly, ATPase PilB	1
**Protein secretion system, Type II**	**4**
CBSS-562.2.peg.633	2
FIG002842: hypothetical protein	1
Type IV fimbrial assembly, ATPase PilB	1
General Secretion Pathway	1
General secretion pathway protein F	1
Widespread colonization island	1
Type II/IV secretion system ATPase TadZ/CpaE, associated with Flp pilus assembly	1
**Protein secretion system, Type III**	**1**

Type III secretion system	1
Type III secretion cytoplasmic protein (YscL)	1
**Protein secretion system, Type VI**	**3**
Type VI secretion systems	3
ClpB protein	1
Uncharacterized protein ImpF	1
VgrG protein	1
**Protein translocation across cytoplasmic membrane**	**2**
HtrA and Sec secretion	2
Protein export cytoplasm protein SecA ATPase RNA helicase (TC 3.A.5.1.1)	1
Protein-export membrane protein SecF (TC 3.A.5.1.1)	1
**Sugar Phosphotransferase Systems, PTS**	**1**
Sucrose-specific PTS	1
Sucrose-6-phosphate hydrolase (EC 3.2.1.26)	1
**Ton and Tol transport systems**	**7**
Colicin I receptor precursor	1
Colicin I receptor precursor	1
Ferric siderophore transport system, periplasmic binding protein TonB	1
Ferric siderophore transport system, periplasmic binding protein TonB	1
MotA/TolQ/ExbB proton channel family protein	1
MotA/TolQ/ExbB proton channel family protein	1
Outer membrane lipoprotein omp16 precursor	1
Outer membrane lipoprotein omp16 precursor	1
TonB-dependent hemin , ferrichrome receptor	1
TonB-dependent hemin , ferrichrome receptor	1
TonB-dependent receptor	1
TonB-dependent receptor	1
Type I secretion outer membrane protein, TolC precursor	1

Type I secretion outer membrane protein, TolC precursor	1
**Transport of Manganese**	**1**
Manganese ABC transporter, inner membrane permease protein SitC	1
Manganese ABC transporter, inner membrane permease protein SitC	1
**Transport of Molybdenum**	**1**
Molybdenum transport system permease protein ModB (TC 3.A.1.8.1)	1
Molybdenum transport system permease protein ModB (TC 3.A.1.8.1)	1
**Transport of Zinc**	**1**
Zinc ABC transporter, ATP-binding protein ZnuC	1
Zinc ABC transporter, ATP-binding protein ZnuC	1
**Metabolism of Aromatic Compounds**	**11**
**Benzoate transport and degradation cluster**	**2**
Benzoate transport, inner-membrane translocator precursor	1
Benzoate transport, inner-membrane translocator precursor	1
Shikimate kinase I (EC 2.7.1.71)	1
Shikimate kinase I (EC 2.7.1.71)	1
**Metabolism of central aromatic intermediates**	**4**
Catechol branch of beta-ketoadipate pathway	1
Beta-ketoadipate enol-lactone hydrolase (EC 3.1.1.24)	1
Homogentisate pathway of aromatic compound degradation	1
Transcriptional regulator, IclR family	1
Protocatechuate branch of beta-ketoadipate pathway	1
Beta-ketoadipate enol-lactone hydrolase (EC 3.1.1.24)	1
Salicylate and gentisate catabolism	1
Salicylate hydroxylase (EC 1.14.13.1)	1
**Peripheral pathways for catabolism of aromatic compounds**	**4**
Chloroaromatic degradation pathway	1
Beta-ketoadipate enol-lactone hydrolase (EC 3.1.1.24)	1

n-Phenylalkanoic acid degradation	2
Enoyl-CoA hydratase (EC 4.2.1.17)	1
Long-chain-fatty-acid--CoA ligase (EC 6.2.1.3)	1
Salicylate ester degradation	1
Salicylate hydroxylase (EC 1.14.13.1)	1
**Phenylacetyl-CoA catabolic pathway (core)**	**1**
Phenylacetic acid degradation protein PaaN2, ring-opening aldehyde dehydrogenase (EC 1.2.1.3)	1
Phenylacetic acid degradation protein PaaN2, ring-opening aldehyde dehydrogenase (EC 1.2.1.3)	1
**Miscellaneous**	**17**
**Broadly distributed proteins not in subsystems**	**2**
YbbL ABC transporter ATP-binding protein	1
YbbL ABC transporter ATP-binding protein	1
YciL protein	1
YciL protein	1
**Carbonate Biomineralization**	**4**
Electron transfer flavoprotein, alpha subunit	1
Electron transfer flavoprotein, alpha subunit	1
Enoyl-CoA hydratase (EC 4.2.1.17)	1
Enoyl-CoA hydratase (EC 4.2.1.17)	1
Long-chain-fatty-acid--CoA ligase (EC 6.2.1.3)	1
Long-chain-fatty-acid--CoA ligase (EC 6.2.1.3)	1
Transcriptional regulator, TetR family	1
Transcriptional regulator, TetR family	1
**Plant-Prokaryote DOE project**	**8**
At2g33980 At1g28960	2
FIG017823: ATPase, MoxR family	1
tRNA nucleotidyltransferase (EC 2.7.7.21) (EC 2.7.7.25)	1
COG2363	2

Hydroxymethylpyrimidine ABC transporter, substrate-binding component	1
Thiazole biosynthesis protein ThiG	1
Conserved gene cluster possibly involved in RNA metabolism	1
Serine acetyltransferase (EC 2.3.1.30)	1
Iojap	3
Adenylate cyclase (EC 4.6.1.1)	1
Orotate phosphoribosyltransferase (EC 2.4.2.10)	1
Phosphatidate cytidylyltransferase (EC 2.7.7.41)	1
**ZZ gjo need homes**	**3**
Lipid A export ATP-binding/permease protein MsbA	1
Lipid A export ATP-binding/permease protein MsbA	1
Na+/H+ antiporter NhaB	1
Na+/H+ antiporter NhaB	1
Sodium/glutamate symport protein	1
Sodium/glutamate symport protein	1
**Motility and Chemotaxis**	**23**
**Bacterial Chemotaxis**	**2**
Dipeptide-binding ABC transporter, periplasmic substrate-binding component (TC 3.A.1.5.2)	1
Dipeptide-binding ABC transporter, periplasmic substrate-binding component (TC 3.A.1.5.2)	1
Methyl-accepting chemotaxis protein I (serine chemoreceptor protein)	1
Methyl-accepting chemotaxis protein I (serine chemoreceptor protein)	1
**Flagellar motility in Prokaryota**	**15**
Archaeal Flagellum	1
Flagella-related protein FlaI	1
Flagellar motility	5
Flagellar biosynthesis protein FlhA	1
Flagellar biosynthesis protein FlhF	1
Flagellar motor rotation protein MotA	1

Flagellar motor rotation protein MotB	1
Predicted signal transduction protein	1
Flagellum	8
Flagellar biosynthesis protein FlhA	1
Flagellar biosynthesis protein FlhF	1
Flagellar hook-associated protein FlgK	1
Flagellar hook-associated protein FlgL	1
Flagellar hook-length control protein FliK	1
Flagellar motor rotation protein MotA	1
Flagellar motor rotation protein MotB	1
Flagellar sensor histidine kinase FleS	1
Flagellum in Campylobacter	1
Cell division protein FtsI [Peptidoglycan synthetase] (EC 2.4.1.129)	1
**Social motility and nonflagellar swimming in bacteria**	**6**
Bacterial motility:Gliding	6
Cell division protein FtsX	1
GldJ	1
twitching motility protein PilH	1
Type IV fimbrial assembly, ATPase PilB	1
type IV pili signal transduction protein PilI	1
type IV pilus biogenesis protein PilJ	1
**Nitrogen Metabolism**	**17**
**Allantoin Utilization**	**1**
Glycerate kinase (EC 2.7.1.31)	1
Glycerate kinase (EC 2.7.1.31)	1
**Ammonia assimilation**	**5**
Ferredoxin-dependent glutamate synthase (EC 1.4.7.1)	1
Ferredoxin-dependent glutamate synthase (EC 1.4.7.1)	1

Glutamate synthase [NADPH] large chain (EC 1.4.1.13)	1
Glutamate synthase [NADPH] large chain (EC 1.4.1.13)	1
Glutamate synthase [NADPH] small chain (EC 1.4.1.13)	1
Glutamate synthase [NADPH] small chain (EC 1.4.1.13)	1
Glutamine synthetase type I (EC 6.3.1.2)	1
Glutamine synthetase type I (EC 6.3.1.2)	1
Nitrogen regulation protein NR(II) (EC 2.7.3.-)	1
Nitrogen regulation protein NR(II) (EC 2.7.3.-)	1
**Denitrification**	**3**
Nitric oxide -responding transcriptional regulator Dnr (Crp/Fnr family)	1
Nitric oxide -responding transcriptional regulator Dnr (Crp/Fnr family)	1
Nitric oxide -responding transcriptional regulator NnrR (Crp/Fnr family)	1
Nitric oxide -responding transcriptional regulator NnrR (Crp/Fnr family)	1
NnrS protein involved in response to NO	1
NnrS protein involved in response to NO	1
**Dissimilatory nitrite reductase**	**1**
Uroporphyrinogen-III methyltransferase (EC 2.1.1.107)	1
Uroporphyrinogen-III methyltransferase (EC 2.1.1.107)	1
**Nitrate and nitrite ammonification**	**4**
Assimilatory nitrate reductase large subunit (EC:1.7.99.4)	1
Assimilatory nitrate reductase large subunit (EC:1.7.99.4)	1
Nitrate ABC transporter, nitrate-binding protein	1
Nitrate ABC transporter, nitrate-binding protein	1
Nitrite reductase [NAD(P)H] large subunit (EC 1.7.1.4)	1
Nitrite reductase [NAD(P)H] large subunit (EC 1.7.1.4)	1
Response regulator NasT	1
Response regulator NasT	1
**Nitric oxide synthase**	**1**

putative cytochrome P450 hydroxylase	1
putative cytochrome P450 hydroxylase	1
**Nitrogen fixation**	**1**
AnfO protein, required for Mo- and V-independent nitrogenase	1
AnfO protein, required for Mo- and V-independent nitrogenase	1
**Nitrosative stress**	**1**
NnrS protein involved in response to NO	1
NnrS protein involved in response to NO	1
**Nucleosides and Nucleotides**	**16**
**Purines**	**6**
De Novo Purine Biosynthesis	2
Phosphoribosylformylglycinamidine cyclo-ligase (EC 6.3.3.1)	1
Ribose-phosphate pyrophosphokinase (EC 2.7.6.1)	1
Purine conversions	2
Inosine-5'-monophosphate dehydrogenase (EC 1.1.1.205)	1
Polyphosphate kinase (EC 2.7.4.1)	1
Purine Utilization	2
Cytosine/purine/uracil/thiamine/allantoin permease family protein	1
Xanthine dehydrogenase, molybdenum binding subunit (EC 1.17.1.4)	1
**Pyrimidines**	**7**
De Novo Pyrimidine Synthesis	3
Carbamoyl-phosphate synthase large chain (EC 6.3.5.5)	1
Dihydroorotase (EC 3.5.2.3)	1
Orotate phosphoribosyltransferase (EC 2.4.2.10)	1
Novel non-oxidative pathway of Uracil catabolism	1
Uridine kinase (EC 2.7.1.48)	1
pyrimidine conversions	3
CTP synthase (EC 6.3.4.2)	1

Thioredoxin reductase (EC 1.8.1.9)	1
Uridine kinase (EC 2.7.1.48)	1
**Ribonucleotide reduction**	**3**
Ribonucleotide reductase of class II (coenzyme B12-dependent) (EC 1.17.4.1)	1
Ribonucleotide reductase of class II (coenzyme B12-dependent) (EC 1.17.4.1)	1
Ribonucleotide reductase of class III (anaerobic), large subunit (EC 1.17.4.2)	1
Ribonucleotide reductase of class III (anaerobic), large subunit (EC 1.17.4.2)	1
Ribonucleotide reductase transcriptional regulator NrdR	1
Ribonucleotide reductase transcriptional regulator NrdR	1
**Phages, Prophages, Transposable elements, Plasmids**	**12**
**Pathogenicity islands**	**1**
Staphylococcal pathogenicity islands SaPI	1
Heat shock protein 60 family chaperone GroEL	1
**Phage family-specific subsystems**	**5**
Phage cyanophage	3
Phage protein	1
Phage tail fiber protein	1
Phosphate ABC transporter, periplasmic phosphate-binding protein PstS (TC 3.A.1.7.1)	1
T7-like cyanophage core proteins	2
Ribonucleotide reductase of class II (coenzyme B12-dependent) (EC 1.17.4.1)	1
Transaldolase (EC 2.2.1.2)	1
**Phages, Prophages**	**4**
IbrA and IbrB: co-activators of prophage gene expression	1
Co-activator of prophage gene expression IbrA	1
Phage tail fiber proteins	1
Phage tail fiber protein	1
Phage tail proteins	1
Phage tail protein	1

Phage tail proteins 2	1
Phage tape measure	1
**Plasmid related functions**	**1**
Plasmid-encoded T-DNA transfer	1
Inner membrane protein of type IV secretion of T-DNA complex, VirB6	1
**Transposable elements**	**1**
CBSS-203122.12.peg.188	1
Plasmid replication protein RepA	1
**Phosphorus Metabolism**	**16**
**High affinity phosphate transporter and control of PHO regulon**	**4**
Phosphate ABC transporter, periplasmic phosphate-binding protein PstS (TC 3.A.1.7.1)	1
Phosphate ABC transporter, periplasmic phosphate-binding protein PstS (TC 3.A.1.7.1)	1
Phosphate regulon transcriptional regulatory protein PhoB (SphR)	1
Phosphate regulon transcriptional regulatory protein PhoB (SphR)	1
Phosphate transport system permease protein PstA (TC 3.A.1.7.1)	1
Phosphate transport system permease protein PstA (TC 3.A.1.7.1)	1
Polyphosphate kinase (EC 2.7.4.1)	1
Polyphosphate kinase (EC 2.7.4.1)	1
**P uptake (cyanobacteria)**	**2**
Phosphate ABC transporter, periplasmic phosphate-binding protein PstS (TC 3.A.1.7.1)	1
Phosphate ABC transporter, periplasmic phosphate-binding protein PstS (TC 3.A.1.7.1)	1
Phosphate transport system permease protein PstA (TC 3.A.1.7.1)	1
Phosphate transport system permease protein PstA (TC 3.A.1.7.1)	1
**Phosphate metabolism**	**10**
1-acyl-sn-glycerol-3-phosphate acyltransferase (EC 2.3.1.51)	1
1-acyl-sn-glycerol-3-phosphate acyltransferase (EC 2.3.1.51)	1
Low-affinity inorganic phosphate transporter	1
Low-affinity inorganic phosphate transporter	1

Manganese-dependent inorganic pyrophosphatase (EC 3.6.1.1)	1
Manganese-dependent inorganic pyrophosphatase (EC 3.6.1.1)	1
Phosphate ABC transporter, periplasmic phosphate-binding protein PstS (TC 3.A.1.7.1)	1
Phosphate ABC transporter, periplasmic phosphate-binding protein PstS (TC 3.A.1.7.1)	1
Phosphate regulon transcriptional regulatory protein PhoB (SphR)	1
Phosphate regulon transcriptional regulatory protein PhoB (SphR)	1
Phosphate transport system permease protein PstA (TC 3.A.1.7.1)	1
Phosphate transport system permease protein PstA (TC 3.A.1.7.1)	1
Polyphosphate kinase (EC 2.7.4.1)	1
Polyphosphate kinase (EC 2.7.4.1)	1
Probable low-affinity inorganic phosphate transporter	1
Probable low-affinity inorganic phosphate transporter	1
Pyrophosphate-energized proton pump (EC 3.6.1.1)	1
Pyrophosphate-energized proton pump (EC 3.6.1.1)	1
Sodium-dependent phosphate transporter	1
Sodium-dependent phosphate transporter	1
**Photosynthesis**	**1**
**Light-harvesting complexes**	**1**
Phycobilisome	1
Phycoerythrocyanin beta chain	1
**Potassium metabolism**	**5**
**Glutathione-regulated potassium-efflux system and associated functions**	**1**
Trk system potassium uptake protein TrkA	1
Trk system potassium uptake protein TrkA	1
**Potassium homeostasis**	**4**
Potassium uptake protein TrkH	1
Potassium uptake protein TrkH	1
Potassium-transporting ATPase B chain (EC 3.6.3.12) (TC 3.A.3.7.1)	1

Potassium-transporting ATPase B chain (EC 3.6.3.12) (TC 3.A.3.7.1)	1
putative Glutathione-regulated potassium-efflux system protein KefB	1
putative Glutathione-regulated potassium-efflux system protein KefB	1
Trk system potassium uptake protein TrkA	1
Trk system potassium uptake protein TrkA	1
**Protein Metabolism**	**43**
**Protein biosynthesis**	**19**
Ribosome biogenesis bacterial	4
hypothetical protein sometimes fused to ribosomal protein S6 glutaminyl transferase	1
Inactive homolog of metal-dependent proteases, putative molecular chaperone	1
Ribosomal large subunit pseudouridine synthase A (EC 4.2.1.70)	1
Ribosomal large subunit pseudouridine synthase C (EC 4.2.1.70)	1
Ribosome LSU bacterial	2
LSU ribosomal protein L23p (L23Ae)	1
LSU ribosomal protein L25p	1
Ribosome SSU bacterial	3
SSU ribosomal protein S12p (S23e)	1
SSU ribosomal protein S1p	1
SSU ribosomal protein S7p (S5e)	1
Translation termination factors bacterial	1
Peptide chain release factor 3	1
tRNA aminoacylation, Arg	1
Arginyl-tRNA synthetase (EC 6.1.1.19)	1
tRNA aminoacylation, Gly	1
Glycyl-tRNA synthetase beta chain (EC 6.1.1.14)	1
tRNA aminoacylation, Leu	1
Leucyl-tRNA synthetase (EC 6.1.1.4)	1
tRNA aminoacylation, Phe	1

Phenylalanyl-tRNA synthetase beta chain (EC 6.1.1.20)	1
Universal GTPases	5
GTPase and tRNA-U34 5-formylation enzyme TrmE	1
GTP-binding protein EngA	1
GTP-binding protein HflX	1
Probable GTPase related to EngC	1
Signal recognition particle, subunit Ffh SRP54 (TC 3.A.5.1.1)	1
**Protein degradation**	**13**
Aminopeptidases (EC 3.4.11.-)	2
Membrane alanine aminopeptidase N (EC 3.4.11.2)	1
Xaa-Pro aminopeptidase (EC 3.4.11.9)	1
Metallocarboxypeptidases (EC 3.4.17.-)	1
D-alanyl-D-alanine carboxypeptidase (EC 3.4.16.4)	1
Proteasome bacterial	2
ATP-dependent protease La (EC 3.4.21.53) Type I	1
ATP-dependent protease La (EC 3.4.21.53) Type II	1
Protein degradation	1
Leucyl/phenylalanyl-tRNA--protein transferase (EC 2.3.2.6)	1
Proteolysis in bacteria, ATP-dependent	5
ATP-dependent Clp protease ATP-binding subunit ClpA	1
ATP-dependent protease La (EC 3.4.21.53)	1
ATP-dependent protease La (EC 3.4.21.53) Type I	1
ATP-dependent protease La (EC 3.4.21.53) Type II	1
ClpB protein	1
Putative TldE-TldD proteolytic complex	2
TldE/PmbA family protein, Actinobacterial subgroup	1
TldE/PmbA protein, part of proposed TldE/TldD proteolytic complex (PMID 12029038)	1
**Protein folding**	**5**

GroEL GroES	1
Heat shock protein 60 family chaperone GroEL	1
Peptidyl-prolyl cis-trans isomerase	1
Peptidyl-prolyl cis-trans isomerase ppiD (EC 5.2.1.8)	1
Periplasmic disulfide interchange	2
Cytochrome c-type biogenesis protein CcdA (DsbD analog)	1
Cytochrome c-type biogenesis protein DsbD, protein-disulfide reductase (EC 1.8.1.8)	1
Protein chaperones	1
ClpB protein	1
**Protein processing and modification**	**5**
N-linked Glycosylation in Bacteria	1
UDP-glucose 4-epimerase (EC 5.1.3.2)	1
Peptide methionine sulfoxide reductase	1
Peptide methionine sulfoxide reductase MsrB (EC 1.8.4.12)	1
Protein Acetylation and Deacetylation in Bacteria	1
Acetyl-coenzyme A synthetase (EC 6.2.1.1)	1
Ribosomal protein S12p Asp methylthiotransferase	1
SSU ribosomal protein S12p (S23e)	1
Signal peptidase	1
Signal peptidase I (EC 3.4.21.89)	1
**Selenoproteins**	**1**
Glycine reductase, sarcosine reductase and betaine reductase	1
Thioredoxin reductase (EC 1.8.1.9)	1
**Regulation and Cell signaling**	**13**
**cAMP signaling in bacteria**	**3**
Adenylate cyclase (EC 4.6.1.1)	1
Adenylate cyclase (EC 4.6.1.1)	1
cAMP-binding proteins - catabolite gene activator and regulatory subunit of cAMP-dependent protein kinases	1

cAMP-binding proteins - catabolite gene activator and regulatory subunit of cAMP-dependent protein kinases	1
Predicted signal-transduction protein containing cAMP-binding and CBS domains	1
Predicted signal-transduction protein containing cAMP-binding and CBS domains	1
**Cell envelope-associated LytR-CpsA-Psr transcriptional attenuators**	**1**
Cell envelope-associated transcriptional attenuator LytR-CpsA-Psr, subfamily F2 (as in PMID19099556)	1
Cell envelope-associated transcriptional attenuator LytR-CpsA-Psr, subfamily F2 (as in PMID19099556)	1
**Oxygen and light sensor PpaA-PpsR**	**1**
Phytochrome, two-component sensor histidine kinase (EC 2.7.3.-)	1
Phytochrome, two-component sensor histidine kinase (EC 2.7.3.-)	1
**Programmed Cell Death and Toxin-antitoxin Systems**	**2**
Murein hydrolase regulation and cell death	1
Antiholin-like protein LrgA	1
Phd-Doc, YdcE-YdcD toxin-antitoxin (programmed cell death) systems	1
Death on curing protein, Doc toxin	1
**Quorum sensing and biofilm formation**	**2**
Biofilm Adhesin Biosynthesis	1
Biofilm PGA synthesis deacetylase PgaB (EC 3.-)	1
Quorum Sensing: Autoinducer-2 Synthesis	1
S-adenosylmethionine synthetase (EC 2.5.1.6)	1
**Sex pheromones in Enterococcus faecalis and other Firmicutes**	**1**
Oligopeptide ABC transporter, periplasmic oligopeptide-binding protein OppA (TC 3.A.1.5.1)	1
Oligopeptide ABC transporter, periplasmic oligopeptide-binding protein OppA (TC 3.A.1.5.1)	1
**Zinc regulated enzymes**	**3**
Dihydroorotase (EC 3.5.2.3)	1
Dihydroorotase (EC 3.5.2.3)	1
N-acetylmuramoyl-L-alanine amidase (EC 3.5.1.28)	1
N-acetylmuramoyl-L-alanine amidase (EC 3.5.1.28)	1
Porphobilinogen synthase (EC 4.2.1.24)	1

Porphobilinogen synthase (EC 4.2.1.24)	1
**Respiration**	**21**
**ATP synthases**	**2**
F0F1-type ATP synthase	2
ATP synthase A chain (EC 3.6.3.14)	1
ATP synthase delta chain (EC 3.6.3.14)	1
**Biogenesis of c-type cytochromes**	**4**
Cytochrome c heme lyase subunit CcmF	1
Cytochrome c heme lyase subunit CcmF	1
Cytochrome c-type biogenesis protein CcdA (DsbD analog)	1
Cytochrome c-type biogenesis protein CcdA (DsbD analog)	1
Cytochrome c-type biogenesis protein CcsA/ResC	1
Cytochrome c-type biogenesis protein CcsA/ResC	1
Cytochrome c-type biogenesis protein DsbD, protein-disulfide reductase (EC 1.8.1.8)	1
Cytochrome c-type biogenesis protein DsbD, protein-disulfide reductase (EC 1.8.1.8)	1
**Electron accepting reactions**	**5**
Anaerobic respiratory reductases	2
Anaerobic dehydrogenases, typically selenocysteine-containing	1
Electron transfer flavoprotein-ubiquinone oxidoreductase (EC 1.5.5.1)	1
Cytochrome c oxidases d@O copy	1
Transport ATP-binding protein CydC	1
Terminal cytochrome d ubiquinol oxidases	1
Transport ATP-binding protein CydC	1
Terminal cytochrome oxidases	1
Transport ATP-binding protein CydC	1
**Electron donating reactions**	**6**
Na(+)-translocating NADH-quinone oxidoreductase and rnf-like group of electron transport complexes	2
Electron transport complex protein RnfB	1

Na(+)-translocating NADH-quinone reductase subunit A (EC 1.6.5.-)	1
Respiratory Complex I	2
NADH-ubiquinone oxidoreductase chain E (EC 1.6.5.3)	1
NADH-ubiquinone oxidoreductase chain N (EC 1.6.5.3)	1
Respiratory dehydrogenases 1	2
Aerobic glycerol-3-phosphate dehydrogenase (EC 1.1.5.3)	1
Glycerol-3-phosphate dehydrogenase (EC 1.1.5.3)	1
**Formate hydrogenase**	**1**
formate dehydrogenase formation protein FdhE	1
formate dehydrogenase formation protein FdhE	1
**Sodium Ion-Coupled Energetics**	**1**
Na+ translocating decarboxylases and related biotin-dependent enzymes	1
Oxaloacetate decarboxylase beta chain (EC 4.1.1.3)	1
**Soluble cytochromes and functionally related electron carriers**	**2**
Cytochrome c551/c552	1
Cytochrome c551/c552	1
soluble [2Fe-2S] ferredoxin	1
soluble [2Fe-2S] ferredoxin	1
**RNA Metabolism**	**45**
**RNA processing and modification**	**40**
16S rRNA modification within P site of ribosome	2
Cell division protein FtsI [Peptidoglycan synthetase] (EC 2.4.1.129)	1
Penicillin-binding protein 2 (PBP-2)	1
ATP-dependent RNA helicases, bacterial	1
ATP-dependent RNA helicase Bcep18194_A5658	1
mcm5s2U biosynthesis in tRNA	1
histone acetyltransferase, ELP3 family	1
mnm5U34 biosynthesis bacteria	2

GTPase and tRNA-U34 5-formylation enzyme TrmE	1
tRNA 5-methylaminomethyl-2-thiouridine synthase TusA	1
Polyadenylation bacterial	2
Polyribonucleotide nucleotidyltransferase (EC 2.7.7.8)	1
tRNA nucleotidyltransferase (EC 2.7.7.21) (EC 2.7.7.25)	1
Queuosine-Archaeosine Biosynthesis	4
glutamyl-Q-tRNA synthetase	1
Peptidyl-prolyl cis-trans isomerase (EC 5.2.1.8)	1
Permease of the drug/metabolite transporter (DMT) superfamily	1
S-adenosylmethionine:tRNA ribosyltransferase-isomerase (EC 5.-.-.-)	1
Ribonuclease H	1
hypothetical protein ssl1918	1
RNA processing and degradation, bacterial	3
3'-to-5' exoribonuclease RNase R	1
Exoribonuclease II (EC 3.1.13.1)	1
Ribonuclease E inhibitor RraA	1
RNA pseudouridine syntheses	3
Ribosomal large subunit pseudouridine synthase A (EC 4.2.1.70)	1
Ribosomal large subunit pseudouridine synthase C (EC 4.2.1.70)	1
tRNA pseudouridine synthase A (EC 4.2.1.70)	1
rRNA modification Bacteria	2
Ribosomal large subunit pseudouridine synthase A (EC 4.2.1.70)	1
Ribosomal large subunit pseudouridine synthase C (EC 4.2.1.70)	1
tRNA modification Archaea	1
tRNA pseudouridine synthase A (EC 4.2.1.70)	1
tRNA modification Bacteria	7
glutamyl-Q-tRNA synthetase	1
GTPase and tRNA-U34 5-formylation enzyme TrmE	1

Ribosomal large subunit pseudouridine synthase A (EC 4.2.1.70)	1
S-adenosylmethionine:tRNA ribosyltransferase-isomerase (EC 5.-.-.-)	1
tRNA 5-methylaminomethyl-2-thiouridine synthase TusA	1
tRNA pseudouridine synthase A (EC 4.2.1.70)	1
tRNA(Ile)-lysidine synthetase	1
tRNA modification yeast cytoplasmic	3
histone acetyltransferase, ELP3 family	1
tRNA N2,N2-dimethyl(Guanine26-N2)-methyltransferase (EC 2.1.1.32)	1
tRNA pseudouridine synthase A (EC 4.2.1.70)	1
tRNA modification yeast mitochondrial	3
GTPase and tRNA-U34 5-formylation enzyme TrmE	1
tRNA N2,N2-dimethyl(Guanine26-N2)-methyltransferase (EC 2.1.1.32)	1
tRNA pseudouridine synthase A (EC 4.2.1.70)	1
tRNA nucleotidyltransferase	1
tRNA nucleotidyltransferase (EC 2.7.7.21) (EC 2.7.7.25)	1
tRNA processing	2
tRNA pseudouridine synthase A (EC 4.2.1.70)	1
tRNA(Ile)-lysidine synthetase	1
Wyeosine-MimG Biosynthesis	1
Thioredoxin reductase (EC 1.8.1.9)	1
YrdC-YciO	1
Inactive homolog of metal-dependent proteases, putative molecular chaperone	1
**Transcription**	**5**
RNA polymerase bacterial	2
DNA-directed RNA polymerase beta subunit (EC 2.7.7.6)	1
DNA-directed RNA polymerase beta' subunit (EC 2.7.7.6)	1
Transcription factors bacterial	2
Transcription elongation factor GreB	1

Transcription-repair coupling factor	1
Transcription initiation, bacterial sigma factors	1
RNA polymerase sigma factor RpoH-related protein	1
**Secondary Metabolism**	**3**
**Bacterial cytostatics, differentiation factors and antibiotics**	**1**
Paerucumarin Biosynthesis	1
PvcA protein, related to known isonitrile synthases	1
**Biologically active compounds in metazoan cell defence and differentiation**	**1**
Steroid sulfates	1
Arylsulfatase (EC 3.1.6.1)	1
**Plant Hormones**	**1**
Auxin biosynthesis	1
Tryptophan synthase alpha chain (EC 4.2.1.20)	1
**Stress Response**	**22**
**Bacterial hemoglobins**	**1**
diguanylate cyclase/phosphodiesterase (GGDEF & EAL domains) with PAS/PAC sensor(s)	1
diguanylate cyclase/phosphodiesterase (GGDEF & EAL domains) with PAS/PAC sensor(s)	1
**Detoxification**	**4**
Housecleaning nucleoside triphosphate pyrophosphatases	1
5'-nucleotidase YjjG (EC 3.1.3.5)	1
Nucleoside triphosphate pyrophosphohydrolase MazG	1
Nucleoside triphosphate pyrophosphohydrolase MazG	1
Nudix proteins (nucleoside triphosphate hydrolases)	2
Adenosine (5')-pentaphospho-(5'')-adenosine pyrophosphohydrolase (EC 3.6.1.-)	1
ADP-ribose pyrophosphatase (EC 3.6.1.13)	1
**Hfl operon**	**1**
GTP-binding protein HflX	1
GTP-binding protein HflX	1

**Osmotic stress**	**3**
Choline and Betaine Uptake and Betaine Biosynthesis	1
High-affinity choline uptake protein BetT	1
Synthesis of osmoregulated periplasmic glucans	2
Cyclic beta-1,2-glucan synthase (EC 2.4.1.-)	1
Glucans biosynthesis protein G precursor	1
**Oxidative stress**	**11**
Glutathione: Biosynthesis and gamma-glutamyl cycle	1
Gamma-glutamyltranspeptidase (EC 2.3.2.2)	1
Glutathione: Non-redox reactions	1
Lactoylglutathione lyase (EC 4.4.1.5)	1
Oxidative stress	6
Catalase (EC 1.11.1.6)	1
NnrS protein involved in response to NO	1
Organic hydroperoxide resistance transcriptional regulator	1
Paraquat-inducible protein B	1
Peroxide stress regulator PerR, FUR family	1
Phytochrome, two-component sensor histidine kinase (EC 2.7.3.-)	1
Protection from Reactive Oxygen Species	2
Catalase (EC 1.11.1.6)	1
Cytochrome c551 peroxidase (EC 1.11.1.5)	1
Redox-dependent regulation of nucleus processes	1
NAD-dependent glyceraldehyde-3-phosphate dehydrogenase (EC 1.2.1.12)	1
**SigmaB stress responce regulation**	**1**
Serine phosphatase RsbU, regulator of sigma subunit	1
Serine phosphatase RsbU, regulator of sigma subunit	1
**Universal stress protein family**	**1**
Universal stress protein E	1

Universal stress protein E	1
**Sulfur Metabolism**	**12**
**Galactosylceramide and Sulfatide metabolism**	**3**
Alpha-galactosidase (EC 3.2.1.22)	1
Alpha-galactosidase (EC 3.2.1.22)	1
Arylsulfatase (EC 3.1.6.1)	1
Arylsulfatase (EC 3.1.6.1)	1
Beta-galactosidase (EC 3.2.1.23)	1
Beta-galactosidase (EC 3.2.1.23)	1
**Inorganic sulfur assimilation**	**1**
Inorganic Sulfur Assimilation	1
3'(2'),5'-bisphosphate nucleotidase (EC 3.1.3.7)	1
**Organic sulfur assimilation**	**6**
Alkanesulfonate assimilation	2
Alkanesulfonates transport system permease protein	1
Arylsulfatase (EC 3.1.6.1)	1
Alkanesulfonates Utilization	1
Alkanesulfonates transport system permease protein	1
L-Cystine Uptake and Metabolism	1
Cystathionine beta-lyase (EC 4.4.1.8)	1
Taurine Utilization	1
Taurine transport system permease protein TauC	1
Utilization of glutathione as a sulphur source	1
Gamma-glutamyltranspeptidase (EC 2.3.2.2)	1
**Sulfur oxidation**	**1**
Cytochrome c-type biogenesis protein CcdA (DsbD analog)	1
Cytochrome c-type biogenesis protein CcdA (DsbD analog)	1
**Thioredoxin-disulfide reductase**	**1**

Thioredoxin reductase (EC 1.8.1.9)	1
Thioredoxin reductase (EC 1.8.1.9)	1
**Virulence, Disease and Defense**	**34**
**Bacterial cyanide production and tolerance mechanisms**	**1**
formate dehydrogenase formation protein FdhE	1
formate dehydrogenase formation protein FdhE	1
**Bacteriocins, ribosomally synthesized antibacterial peptides**	**1**
Colicin V and Bacteriocin Production Cluster	1
tRNA pseudouridine synthase A (EC 4.2.1.70)	1
**C jejuni colonization of chick caeca**	**5**
Branched-chain amino acid ABC transporter, amino acid-binding protein (TC 3.A.1.4.1)	1
Branched-chain amino acid ABC transporter, amino acid-binding protein (TC 3.A.1.4.1)	1
Flagellar hook-associated protein FlgK	1
Flagellar hook-associated protein FlgK	1
Flagellar motor rotation protein MotA	1
Flagellar motor rotation protein MotA	1
Flagellar motor rotation protein MotB	1
Flagellar motor rotation protein MotB	1
Predicted signal transduction protein	1
Predicted signal transduction protein	1
**Fimbriae of the Chaperone/Usher Assembly Pathway**	**1**
σ-Fimbriae	1
Sigma-fimbriae usher protein	1
**Invasion and intracellular resistance**	**1**
Listeria surface proteins: Internalin-like proteins	1
internalin, putative	1
**Resistance to antibiotics and toxic compounds**	**21**
Aminoglycoside adenylyltransferases	1

Spectinomycin 9-O-adenylyltransferase	1
Arsenic resistance	1
Arsenic efflux pump protein	1
Cobalt-zinc-cadmium resistance	4
Cobalt-zinc-cadmium resistance protein CzcD	1
Heavy metal RND efflux outer membrane protein, CzcC family	1
Probable Co/Zn/Cd efflux system membrane fusion protein	1
Transcriptional regulator, MerR family	1
Copper homeostasis	2
Cytochrome c heme lyase subunit CcmF	1
Multicopper oxidase	1
Copper homeostasis: copper tolerance	1
Membrane protein, suppressor for copper-sensitivity ScsB	1
Methicillin resistance in Staphylococci	1
Undecaprenyl-phosphate N-acetylglucosaminyl 1-phosphate transferase (EC 2.7.8.-)	1
Multidrug efflux pump in Campylobacter jejuni (CmeABC operon)	2
RND efflux system, inner membrane transporter CmeB	1
RND efflux system, outer membrane lipoprotein CmeC	1
Multidrug Resistance Efflux Pumps	4
Multi antimicrobial extrusion protein (Na(+)/drug antiporter), MATE family of MDR efflux pumps	1
RND efflux system, inner membrane transporter CmeB	1
RND efflux system, outer membrane lipoprotein CmeC	1
Type I secretion outer membrane protein, TolC precursor	1
Multidrug Resistance, Tripartite Systems Found in Gram Negative Bacteria	1
Outer membrane component of tripartite multidrug resistance system	1
Resistance to fluoroquinolones	3
DNA gyrase subunit A (EC 5.99.1.3)	1
DNA gyrase subunit B (EC 5.99.1.3)	1

Topoisomerase IV subunit A (EC 5.99.1.-)	1
Zinc resistance	1
Sensor protein of zinc sigma-54-dependent two-component system	1
**Streptococcus agalactiae virulome**	**1**
UDP-N-acetylglucosamine 2-epimerase (EC 5.1.3.14)	1
UDP-N-acetylglucosamine 2-epimerase (EC 5.1.3.14)	1
**Type III, Type IV, Type VI, ESAT secretion systems**	**3**
Type 4 secretion and conjugative transfer	3
Conjugative transfer protein TrbB	1
Conjugative transfer protein TrbI	1
IncF plasmid conjugative transfer DNA-nicking and unwinding protein TraI	1
**Total**	**816**
