# Supplementary material for: Genome-Wide Study of the Defective Sucrose Fermenter Strain of Vibrio cholerae from the Latin American Cholera Epidemic
Source: PLoS One. 2012 May 25;7(5):e37283. doi: 10.1371/journal.pone.0037283 (PMC3360680; doi:10.1371/journal.pone.0037283)
Supplement: Table S5 — Comparison of the allelic composition between the strains IEC224, INDRE 91/11, MO10, and N16961. (PDF) [file pone.0037283.s007.pdf]

Genome-Wide Study of the Defective Sucrose Fermenter Strain of *Vibrio cholerae* from the Latin American Cholera Epidemic

(Garza DR, Thompson CC, Loureiro ECB, Dutilh BE, Inada DT, et al.)

Table S5

Comparison of the Allelic Composition between the Strains IEC224, INDRE 91/1, M010, and N16961

Chromosome I				
Locus (N16961)	IEC224	INDRE 91/1	M010	N16961
VC0030	A	A	B	B
VC0035	A	A	B	B
VC0165	A	A	A	B
VC0183	A	A1	A	B
VC0185	A	A	B	B
VC0258	A	A1	X	B
VC0276	A	A	B	B
VC0280	A	A	B	B

Chromosome II				
Locus (N16961)	IEC224	INDRE 91/1	M010	N16961
VCA0020	A	A	B	B
VCA0027	A	B	B	B
VCA0034	A	A	B	B
VCA0064	A	A	B	B
VCA0076	A	A	A	B
VCA0097	A	B	B	B
VCA0103	A	A	B	B
VCA0118	A	B	B	B

VC0329	A	A	B	B
VC0340	A	A	A	B
VC0342	A	A	B	B
VC0343	A	A	A	B
VC0398	A	A	A	B
VC0415	A	B	B	B
VC0489	A	A1	B	B
VC0518	A	X	A	B
VC0525	A	A	B	B
VC0543	A	A	A	B
VC0620	A	A	B	B
VC0624	A	A	A	B
VC0653	A	A	A	B
VC0674	A	A	B	B
VC0695	A	A	B	B
VC0722	A	A	B	B
VC0723	A	B	B	B
VC0752	A	A	B	B
VC0784	A	B	B	B
VC0798	A	A	A	B
VC0799	A	B	B	B
VC0837	A	A	B	B
VC0933	A	A	A	B
VC0947	A	A	A	B
VC1047	A	A	B	B
VC1076	A	A	A	B
VC1113	A	A	A	B

VCA0121	A	B	B	B
VCA0129	A	A	A	B
VCA0137	A	A	A	B
VCA0146	A	A	A	B
VCA0155	A	B	B	B
VCA0157	A	B	B	B
VCA0193	A	B	B	B
VCA0239	A	A	B	B
VCA0246	A	A	B	B
VCA0250	A	A	B	B
VCA0317	A	A	B	B
VCA0368	A	A	A	B
VCA0388	A	X	B	B
VCA0413	A	B	X	B
VCA0433	A	B	X	B
VCA0436	A	B	B1	B
VCA0535	A	A	B	B
VCA0546	A	A	B	B
VCA0557	A	A	B	B
VCA0578	A	A1	B	B
VCA0629	A	A	B	B
VCA0684	A	A	B	B
VCA0818	A	A	A	B
VCA0861	A	A	A	B
VCA0931	A	A	B	B
VCA0944	A	A	A	B
VCA1043	A	A	A	B

VC1145	A	A	B	B
VC1165	A	A	A	B
VC1188	A	A	B	B
VC1261	A	A	A	B
VC1295	A	A	B	B
VC1374	A	A	B	B
VC1382	A	B	B	B
VC1451	A	X	A	B
VC1454	A	B	B	B
VC1462	A	A	B	B
VC1463	A	A	B	B
VC1482	A	A	A	B
VC1486	A	A	A	B
VC1585	A	A	B	B
VC1634	A	B	B	B
VC1645	A	A	A	B
VC1650	A	A	B	B
VC1653	A	A	B	B
VC1771	A	A	X	B
VC1817	A	A	B	B
VC1849	A	X	B	B
VC1850	A	X	A	B
VC1887	A	A	B	B
VC1898	A	A	B	B
VC1925	A	A	A	B
VC1967	A	A	B	B
VC1971	A	A	A	B

VCA1055	A	A	A	B
VCA1095	A	A	B	B

**VC2001**	A	A	A	B
**VC2027**	A	X	A	B
**VC2173**	A	A	A	B
**VC2191**	A	A	B	B
**VC2208**	A	A	B	B
**VC2224**	A	A	A	B
**VC2225**	A	A	A	B
**VC2231**	A	B	B	B
**VC2237**	A	B	B	B
**VC2282**	A	A	B	B
**VC2295**	A	B	B	B
**VC2342**	A	A	A	B
**VC2387**	A	B	B	B
**VC2416**	A	A	A	B
**VC2472**	A	A	B	B
**VC2497**	A	X	A	B
**VC2498**	A	X	B	B
**VC2556**	A	B	B	B
**VC2614**	A	B	B	B
**VC2618**	A	A	B	B
**VC2671**	A	A	B	B
**VC2676**	A	A	B	B
**VC2710**	A	A	A	B

Allele comparison of the strains IEC224 and N16961 that presented small polymorphisms (up to five mutations or indels) with the strains INDRE 91/1 from Mexico (isolated in 1991), and M010 from India (0139). IEC224 alleles are represented as an **A** and colored red, while the N169961 alleles are represented as a **B** and blue color. Pink **A1** are the alleles that match the IEC224 allele in comparison to the N16961, but contain unique differences, and the light blue **B1** are alleles that match the N16961 allele in comparison to the IEC224 allele, but contain unique features. Black **X** are genes that are absent from the strains genome annotation.

Unique SNP Profile and Mobile Genomic Regions that the Latin American Epidemic Lineage of *V. cholerae* from the Prototypical O1 El Tor Strain

	27804	34254	185099	281442	286337	364550	376379	VSPII	558833	656622	720770	743406	743698	750723	805327	899563	899712
N16961	C	C	G	C	C	C	C		G	G	C	A	C	T	C	A	C
A5_ANGOLA_1989	C	T	T	T	T	C	T		A	G	T	G	A	T	T	A	T
INDRE 91/1_MEXICO_1991	T	T	T	T	T	A	T		A	A	T	G	A	A	T	G	T
15 Latin American Strains (ENA)	T	T	T	T	T	A	T		A	A	T	G	A	A	T	G	T
IEC224_BRAZIL_1994	T	T	T	T	T	A	T		A	A	T	G	A	A	T	G	T

	1262261	1466928	1571665	1571677	1571683	L. A. EP. PHAGE	1989285	2033964	2034684	2437098	2437224	2489368	2515595	2683691	2787100
N16961	C	A	C	A	T		G	C	C	G	C	G	T	G	G
A5_ANGOLA_1989	T	T	T	G	A		A	T	C	A	T	A	T	T	A
INDRE 91/1_MEXICO_1991	T	T	N	N	N		A	T	T	A	T	A	C	T	A
15 Latin American Strains (ENA)	T	T	T	G	A		A	T	T	A	T	A	C	T	A
IEC224_BRAZIL_1994	T	T	T	G	A		A	T	T	A	T	A	C	T	A

	2843849	2879782	2986236	3032674	3072280	3222072	3227892	3241941	3247022	3455480	3479479	3527832	3581904	3684727	3845699	3851113	3998819	4012838
N16961	G	A	C	G	G	A	G	C	C	A	A	G	G	A	C	C	G	C
A5_ANGOLA_1989	A	G	T	A	T	G	G	T	C	G	G	A	G	A	T	G	A	T
INDRE 91/1_MEXICO_1991	A	G	N	A	T	G	A	T	T	G	G	A	A	C	T	G	A	T
15 Latin American Strains (ENA)	A	G	T	A	T	G	A	T	T	G	G	A	A	C	T	G	A	T
IEC224_BRAZIL_1994	A	G	T	A	T	G	A	T	T	G	G	A	A	C	T	G	A	T

Table shows a comparison of single nucleotide polymorphisms (SNPs) that distinguish the Latin American epidemic strain of *V. cholerae* from other El Tor lineages. These SNPs were identified in a study by Mutreja et al. (2011) (see reference bellow) that compared the genomes of 136 V. cholerae O1 El Tor strains, out of which 15 were from Latin America. The putative ancestor strain from Angola (A5) also shares most of these SNPs. The INDRE 91/11 has absent genes because of its incomplete annotation. The VSP II and the Latin American epidemic phage (see main text) are also shown to be shared by the Latin American strains and the putative Angolan ancestor (purple). The lowest line represents the IEC224 genome that is shown to share both the SNP profile and the mobile regions with the Latin American strains.

Reference of the 15 Latin American epidemic *V. cholerae* strains from the comparison above:

Mutreja A, Kim D, Thomson N, Connor T, Lee JH, *et al.* (2011). Evidence for several waves of global transmission in the seventh cholera pandemic. Nature, 000, 1-5.
