# Supplementary figures and images for: Genome-Wide Study of the Defective Sucrose Fermenter Strain of Vibrio cholerae from the Latin American Cholera Epidemic
Source: PLoS One. 2012 May 25;7(5):e37283. doi: 10.1371/journal.pone.0037283 (PMC3360680; doi:10.1371/journal.pone.0037283)

Figure S1

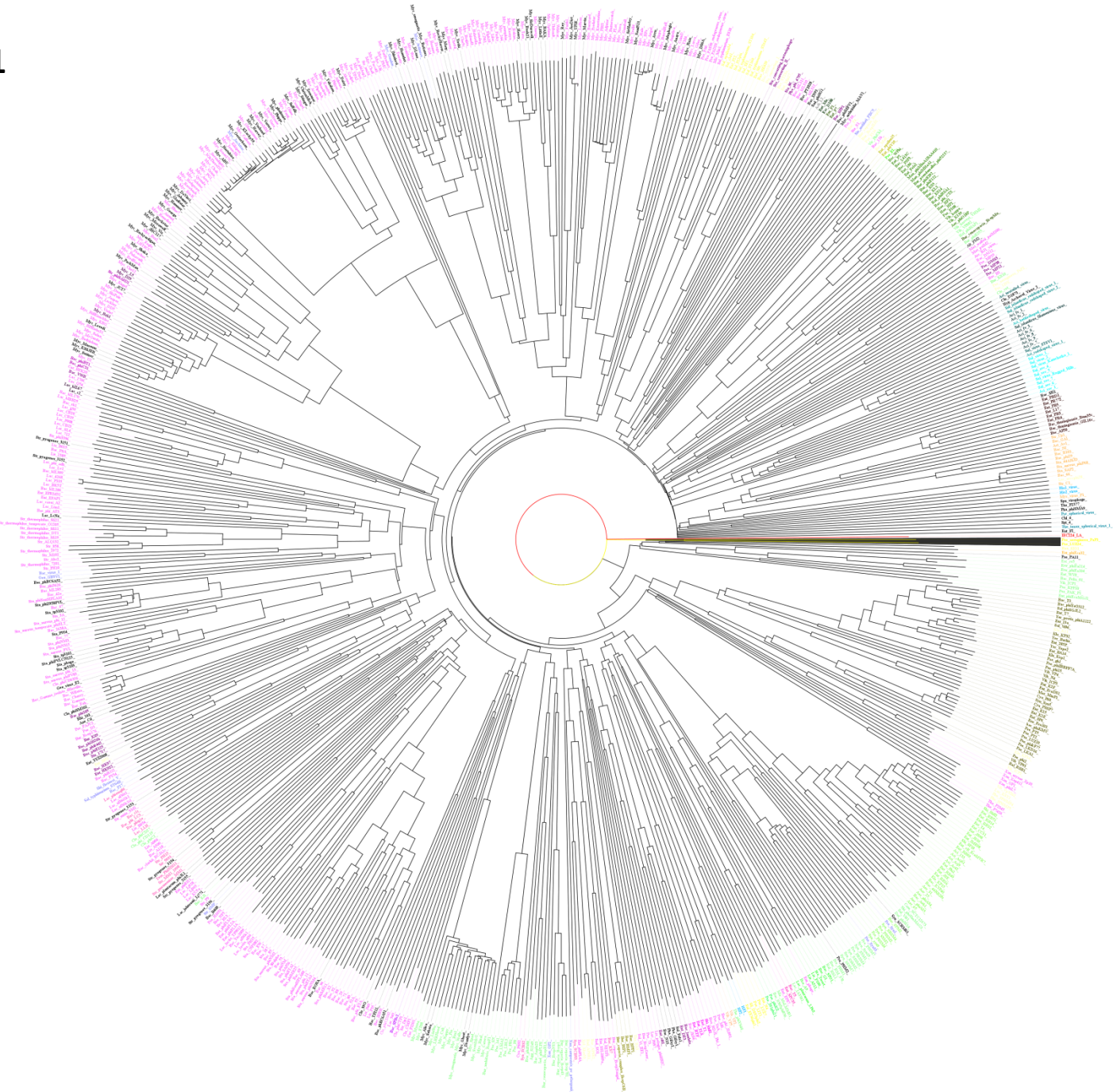

Supplement: Figure S1 — Phage Proteomic Tree with the IEC224's phage highlighted. (PDF) [file pone.0037283.s008.pdf]
